# Identification of potential auxin response candidate genes for soybean rapid canopy coverage through comparative evolution and expression analysis

**DOI:** 10.3389/fpls.2024.1463438

**Published:** 2024-10-03

**Authors:** Deisiany Ferreira Neres, Joseph S. Taylor, John A. Bryant, Bastiaan O. R. Bargmann, R. Clay Wright

**Affiliations:** ^1^ Biological Systems Engineering, Virginia Polytechnic Institute and State University, Blacksburg, VA, United States; ^2^ Translational Plant Science Center, Virginia Polytechnic Institute and State University, Blacksburg, VA, United States; ^3^ School of Plant and Environmental Sciences, Virginia Polytechnic Institute and State University, Blacksburg, VA, United States

**Keywords:** auxin, crop trait improvement, canopy development, phylogenetics, comparative evolutionary analysis, pleiotropy, soybeans (Glycine max)

## Abstract

**Introduction:**

Throughout domestication, crop plants have gone through strong genetic bottlenecks, dramatically reducing the genetic diversity in today’s available germplasm. This has also reduced the diversity in traits necessary for breeders to develop improved varieties. Many strategies have been developed to improve both genetic and trait diversity in crops, from backcrossing with wild relatives, to chemical/radiation mutagenesis, to genetic engineering. However, even with recent advances in genetic engineering we still face the rate limiting step of identifying which genes and mutations we should target to generate diversity in specific traits.

**Methods:**

Here, we apply a comparative evolutionary approach, pairing phylogenetic and expression analyses to identify potential candidate genes for diversifying soybean (Glycine max) canopy cover development via the nuclear auxin signaling gene families, while minimizing pleiotropic effects in other tissues. In soybean, rapid canopy cover development is correlated with yield and also suppresses weeds in organic cultivation.

**Results and discussion:**

We identified genes most specifically expressed during early canopy development from the TIR1/AFB auxin receptor, Aux/IAA auxin co-receptor, and ARF auxin response factor gene families in soybean, using principal component analysis. We defined Arabidopsis thaliana and model legume species orthologs for each soybean gene in these families allowing us to speculate potential soybean phenotypes based on well-characterized mutants in these model species. In future work, we aim to connect genetic and functional diversity in these candidate genes with phenotypic diversity in planta allowing for improvements in soybean rapid canopy cover, yield, and weed suppression. Further development of this and similar algorithms for defining and quantifying tissue- and phenotype-specificity in gene expression may allow expansion of diversity in valuable phenotypes in important crops.

## Introduction

Genetic diversity stands as a significant bottleneck in the improvement of long-cultivated varieties of crops. This limitation stems from the reduced genetic diversity resulting from the lengthy domestication process, often involving a limited number of cultivars evolving from a small pool of accessions (Rani et al., 2023). Such diminished genetic diversity presents a formidable challenge for future trait development, especially in the context of climate change and the imperative to dramatically increase crop production to meet the demands of Earth’s growing population (FAO, 2018; van Dijk et al., 2021).

Soybean [*Glycine max* (L.) Merr.] is among the most cultivated crops worldwide and serves as an example of a crop affected by reduced genetic diversity resulting from a lengthy domestication process (Rani et al., 2023). Soybeans are a chief source of plant-based protein and are commonly used in animal feed, dairy, fuel, and oil production. While significant efforts have been directed towards enhancing soybean production, particularly in developing high-yielding varieties to meet escalating demand for soybean-based products in both traditional and organic agriculture, other crucial traits may have been overlooked. Specifically, demand for organic soy products has increased in recent decades, but weed suppression remains a significant challenge for organic producers (Horn and Burnside, 1985; Wang et al., 2014; Leinonen et al., 2019; Lusk, 2022). Crop-weed interference studies highlight the importance of weeds in agriculture as soybean yield loss can reach up to 90% if necessary management practices aren’t in place. Soybean grain yield is impacted by weed species and density (Horn and Burnside, 1985; Silva et al., 2009).

Rapid canopy cover development, or rapid canopy closure, (RCC) is a highly valuable trait for soybean, as it is both strongly associated with yield and enables early-season weed suppression by outcompeting and shading weeds (Peters et al., 1965; Horn and Burnside, 1985; Xavier et al., 2017). Several studies have found that RCC has a positive effect on soybean yields. For example, Xavier et al., (2017) investigated the genetic architecture of RCC and found that this trait is associated with higher grain yields in soybeans (r = 0.87). Additionally, Peters et al., (1965) observed that RCC reduced weed biomass and increased soybean yields in a row spacing experiment. These findings suggest that RCC is an important trait for improving soybean yields and may be particularly advantageous in organic production where weed competition is a challenge.

RCC is primarily related to plant aerial architecture, which encompasses a range of structures including hypocotyl, cotyledon, apical and axillary meristems, and leaves. By providing greater available leaf area sooner after planting, plants with improved RCC can increase solar radiation interception, which is crucial for photosynthesis and ultimately dictates crop growth and yield (Stewart et al., 2003; Edwards and Purcell, 2005; Hatfield and Dold, 2019). Additionally, increased radiation interception by the desired crop plant will shade weeds, potentially inhibiting their germination and growth. Soybean plants that exhibit RCC also benefit from improved water-use efficiency by minimizing water evaporation and enhancing soil moisture retention (Peters et al., 1965). RCC can help increase yields and improve weed management, making this an important area of research for improving sustainability of agricultural systems. Despite the potential advantages of RCC, few causal mechanisms for this developmental trait have been explored (Clark et al., 2022).

Soybean Genome-Wide Association Studies (GWAS) have shown that auxin is important in early establishment of canopy cover (Xavier et al., 2017; Kaler et al., 2018; Li and Chen, 2023). For instance, among seven SNPs significantly associated with RCC, two are found in a locus that contains three auxin related genes (Xavier et al., 2017). Additionally, Kaler et al., (2018) identified 92 RCC-correlated SNPs, at least two of which are directly auxin related, and several more which are auxin responsive. Li and Chen, (2023) hypothesized that a soybean orthology of *AtARF7* is involved in RCC. Therefore, auxin related genes are a potential RCC breeding target and worth exploring further.

Auxin is a phytohormone involved in numerous aspects of plant growth and development, including response to biotic and abiotic stresses (Padmanabhan et al., 2005; Sun et al., 2016), root and seed development, apical dominance (Tatematsu et al., 2004; Prigge et al., 2020), leaf longevity and expansion, and plant architecture (Davies, 1995; Lim et al., 2010). The auxin signaling pathway comprises three main gene families that act in concert to modulate transcription of numerous response genes. When auxin levels in a plant cell are low, transcriptional repressors AUXIN/INDOLE-3-ACETIC ACID INDUCIBLE (Aux/IAA) are bound to transcription factors AUXIN RESPONSE FACTOR (ARF) proteins repressing auxin-responsive gene expression through Aux/IAA interaction with TOPLESS/TOPLESS-RELATED (TPL/TPR) co-repressor proteins (Abel and Theologis, 1996; Tiwari et al., 2001; Overvoorde et al., 2005; Weijers et al., 2005; Szemenyei et al., 2008). When auxin accumulates, it acts as a molecular glue increasing the affinity between the members of the TRANSPORT INHIBITOR RESPONSE 1/AUXIN SIGNALING F-BOX (TIR1/AFB) auxin receptors and Aux/IAA repressors, which form auxin co-receptor complexes (Tan et al., 2007). Ultimately, as most TIR1/AFB proteins associate with SKP1-CULLIN-F-box ubiquitin ligase complexes, the Aux/IAAs bound to these complexes are subjected to polyubiquitination, targeting them for proteolysis through the 26S proteasome. Degradation of the Aux/IAAs leads to de-repression of activator ARFs and expression of auxin responsive genes (Gray et al., 2001; Ramos et al., 2001; Zenser et al., 2001; Chapman and Estelle, 2009). Additionally, ARF family proteins which repress instead of activate transcription modulate the strength and specificity of auxin responsive gene expression through several potential mechanisms (Cancé et al., 2022). Based on the expression of a network of upstream transcription factors, the interplay between these three auxin gene families varies in a time- and tissue-dependent manner and is responsible for orchestrating different plant fate and agronomic traits (Cancé et al., 2022).

Auxin signaling genes in soybean have previously been associated with root nodulation and development, as well as flowering (Sun et al., 2016; Cai et al., 2017; Li and Chen, 2023). Moreover, auxin genes have been linked to shoot height in soybean plants, such as up-regulation of *GmIAA9* and *GmIAA29* leading to internode elongation, *GmARF9* promoting first pod height, and a dwarf phenotype being associated with *GmIAA27* (Jiang et al., 2018; Su et al., 2022; Zhang et al., 2022). Tuning the function of auxin signaling components in *Arabidopsis thaliana* has generated predictable alterations in root and shoot growth (Guseman et al., 2015; Moss et al., 2015; Wright et al., 2017; Khakhar et al., 2018). However, perhaps due to the complexity of the auxin signaling network, its interaction with other signaling pathways, and its pleiotropic nature (Davies, 1995; Swarup et al., 2002; Vernoux et al., 2011; Calderón Villalobos et al., 2012; Lavy and Estelle, 2016; Prigge et al., 2020), identification of candidate genes in the auxin signaling pathway governing soybean aerial architecture is still a limiting step to rationally tuning soybean RCC.

Auxin signaling genes are known to play many important roles in *Arabidopsis* apical meristem development that point to auxin’s involvement in RCC. Perhaps the most notable are the *ARF1/2* and *ARF3/4* clades of repressor ARFs. Mutants in *ARF2* have enlarged rosette leaves and seeds as well as elongated hypocotyls, but at the cost of reduced fertility (Okushima et al., 2005a). *arf1/arf2* double mutants have even stronger developmental phenotypes (Okushima et al., 2005b). Variants affecting *ARF3/ETTIN* yield pleiotropic effects on leaf and flower development as well as abnormal phyllotaxy (Nishimura et al., 2005; Pekker et al., 2005). *ARF3* and *ARF4* are regulated by trans-acting siRNAs which, when disrupted, lead to changes in the progressive leaf shape from round, flat juvenile leaves to oblong, downward curling (epinastic) adult leaves, also known as heteroblasty (Hunter et al., 2006). Consistent with this association between relief of auxin response gene repression and increased growth of aerial tissues, mutants in the activators *ARF6* and *ARF8* result in dwarfing of aerial tissues (Nagpal et al., 2005; Okushima et al., 2005b). These ARFs are similarly small-RNA-regulated, in this case by miRNA167. Additionally, *arf7/arf19* double mutant plants are of small stature, but also have several detrimental root phenotypes, exhibiting pleiotropy (Okushima et al., 2005b).

In *Arabidopsis*, auxin perception via TIR1/AFB–auxin–Aux/IAA interaction is less clearly associated with RCC related traits than some *arf* phenotypes noted above. *TIR1/AFB* and *Aux/IAA* mutants are more commonly associated with root traits, such as formation of lateral roots, but still there is evidence for their regulation of apical dominance and meristematic tissues, as well as leaf longevity and other above-ground developmental processes (Dharmasiri et al., 2005; Parry et al., 2009; Lim et al., 2010; Salehin et al., 2015). The six TIR1/AFB auxin receptor F-box genes have overlapping functions and are expressed and accumulated in growing organs related to RCC in Arabidopsis, such as shoot apical meristem (SAM) and leaf primordia (Parry et al., 2009). Mutants in two or more of the TIR1/AFB genes of Arabidopsis become increasingly dwarfed (Prigge et al., 2020). In the *Arabidopsis* Aux/IAA family there is more evidence for tissue-specificity in expression and function (Overvoorde et al., 2005). For instance, IAA3 preferentially regulates ARF7 and ARF19 during root development, whereas during leaf expansion and hypocotyl tropic responses these same ARFs are modulated by IAA19 and IAA28 (Wilmoth et al., 2005). However, loss-of-function mutants in *Aux/IAA* genes show subtle or no phenotypes, likely due to redundancy or compensation within this large gene family (Nagpal et al., 2000; Tian et al., 2002; Overvoorde et al., 2005).

We propose to leverage the existing knowledge of auxin signaling and associated traits in *Arabidopsis* and other model species as a foundation for trait engineering in soybean. Through transcriptomic analysis we identify the auxin-signaling genes which are most specifically expressed in tissues involved in early canopy development, RCC-specific genes. Using a Bayesian phylogenetics approach we identified orthology groups of auxin signaling genes, and examined comparative evolutionary evidence that these RCC-specific genes shape the aerial architecture of soybeans. As soybeans have undergone several additional genome duplications relative to *Arabidopsis*, we expect that some soybean paralogs will exhibit more tissue specificity in expression and less pleiotropy. A similar approach to ours was used to examine the evolutionary developmental relationships between *Arabidopsis* and *Zea mays* auxin signaling components (Matthes et al., 2019).

This method allowed us to identify which of the numerous orthologues of the auxin gene families in soybeans are likely involved in RCC and are minimally expressed in other tissues. We identified orthologous groups of *TIR1/AFB*, *Aux/IAA*, and *ARF* genes from *Arabidopsis* and several *Fabaceae* species, and defined an ortholog-based naming system for these *Glycine max* genes to help facilitate evolutionary comparisons. We then performed an expression analysis based on our hypothesis that these auxin signaling genes that are highly and specifically expressed in apical tissues and early development will have the greatest effect on RCC development (RCC-related tissues). Auxin signaling genes are responsible for many developmental processes in plants, thus engineering RCC through manipulation of auxin signaling may result in pleiotropic effects on plant growth and development. To avoid this pitfall, we propose to target genes associated specifically with RCC tissues, as identified via principal component analysis. As a result of phylogenetic and transcriptomic analyses, we identified several candidate auxin-signaling genes that potentially affect RCC. Several soybean orthologs of *Arabidopsis ARF2, ARF8*, and *ARF9* were found to be expressed with high specificity in RCC-related tissues. We also identified a selection of *Aux/IAA* and *TIR1/AFB* candidate genes. *Aux/IAA* genes have the highest tissue specificity in soybeans of the auxin signaling gene families, corroborating the existing body of literature of spatial expression analysis in other species. These findings suggest promising RCC candidate genes for further exploration at both the molecular and organismal levels. Future experiments will be necessary to assess the phenotypic variation in RCC, yield, and other traits associated with allelic variation in these candidate genes. Additionally, further development of this and other algorithms for identifying candidate genes involved in valuable phenotypic traits that may be paired with gene editing techniques to rationally increase phenotypic diversity and accelerate crop breeding.

## Methods

### Sequence collection

Seven *A. thaliana* AFB, twenty-three ARF, and twenty-nine Aux/IAA amino acid sequences from (Hamm et al., 2019) were used in sequence retrieval through the BSgenome.Athaliana.TAIR.TAIR9 and r1001genomes R packages (Hamm et al., 2019; R Core Team, 2023). The peptide sequences were used in the initial Basic Local Alignment Search Tool (BLAST) against the *Glycine max* (assembly Wm82.a4.v1), *Glycine soja* (assembly Gsoja_v1_1), *Medicago truncatula* (assembly Mtruncatula_Mt4_0v1), *Lotus japonicus* (assembly Ljaponicus_Lj1_0v1), as well as *Arabidopsis thaliana* (assembly Athaliana_Araport11) genome databases in Phytozome V13 (Goodstein et al., 2012). Peptide sequences with an E-value less than or equal to 1E–50 were used for analysis. ARF and Aux/IAA sequences were manually separated in some cases according to their length, and the presence of a “QVVGWPPv/i” canonical *Aux/IAA* degron or B3 ARF DNA binding domain. Retrieved peptide sequences in the ARF search containing less than 400 base pairs (bp) or containing auxin canonical degron were removed from the fasta file.

### Sequence alignment

Amino acid (AA) sequence alignment was performed using the function AlignSeqs from DECIPHER 2.24.0 R package (Wright, 2015) following default parameters. The alignment was built according to a similarity tree based on pairwise distinction of shared AA sequences. Two iterations followed the three built in which sequences were re-aligned to the three until convergence was reached. Finally, there was a refinement step in which portions of the alignment were re-aligned to the remnant of the alignment where two alignments were generated and the one that reached convergence with the best sum-of-pairs score was kept (Wright, 2015). Ultimately, we accounted for low information portions of the alignment due to highly variable and/or gap regions by applying the MaskAlignment function in order to remove those regions. All settings, except for windowSize equals to 6 in MaskAlignment, followed default recommendations.

### Phylogenetic analysis

A nexus file was written from masked sequences using write.nexus.data function from ape’s R package version 5.6-2 (Maddison et al., 1997) for building the phylogeny trees. We then built phylogenies on MrBayes v3.2 software (Ronquist et al., 2012) based on the provided peptide sequences in the nexus file of the homologous proteins of AtAFB, AtARF, and AtAux/IAA family members for *G. max*, *G. soja*, *M. truncatula*, and *L. japonicus*. We defined AtCOI1, AtARF17, and AtIAA33 as outgroups to build AFB, ARF, and IAA Bayesian Markov chain Monte Carlo (MCMC) phylogenies, respectively. The likelihood model was defined using lset command with nucleotide substitution model set to protein and rates considered a gamma distribution. The prior probability for the evolutionary model was defined using a fixed protein model (Jones model) and the proposal probability set to zero. The posterior probabilities of the phylogenetic trees were calculated based on the MCMC parameters: for TIR1/AFBs we used a critical value for topological convergence diagnostic of 0.01, 6 chains, and the Markov chain was sampled at every 100 cycles, additionally one quarter of the total samples were discarded when convergence diagnostic was calculated. To explore the possible model parameter space more efficiently and enable model convergence the following parameters were changed for ARF and Aux/IAA phylogenies. For the ARFs, the sample frequency was increased to 10000 cycles, number of runs set to 2, minimum frequency partition set to 0.05 and number of chains equal to 8. Finally, for Aux/IAAs we followed the same parameters as for ARFs, except that we increased the number of chains to 12. Ultimately, phylogeny visualizations and annotations were drawn using ggtree and ggplot2 R packages (Wickham, 2016). Ortholog analysis based on the resulting phylogenies was used to assign *A. thaliana* ortholog names for each *G. max* gene. **Supplementary Table S1** provides a correspondence table of ortholog names to Wm82.a4.v1 gene IDs.

### Expression analysis data

RNA-seq raw data from NCBI project PRJNA241144, containing eleven soybean tissues, were downloaded from http://www.ncbi.nlm.nih.gov/sra/?term=SRP040057 and three other tissues were downloaded from Soybase (https://soybase.org/soyseq/tables_lists/index.php). Soybean open flower (OF), inflorescence before and after meiosis (IBM and IAM), callus, hypocotyl, cotyledon, root tip, axillary meristem (AM), as well as shoot apical meristem at 6, 17, and 38 days (SAM6D, SAM17D, and SAM38D) were obtained from NCBI, whereas root, young leaf and nodule tissues raw data in Soybase. RNA-seq data was used for tissue-specific analysis using Galaxy (https://usegalaxy.org/) *Salmon quant* tool. The raw gene expression counts were normalized to Transcripts Per Million (TPM), allowing the comparison of gene expression between samples. The normalized data from Salmon quant was then plotted in R using the pheatmap function from ggtree version 3.4.1 (Yu, 2022). The heatmap was built using median expression across all tissues equal or greater than 2, and normalized gene expression. Normalization was performed according to the following formula:


z=(x−µ)/σ

where, *z* is the standard score, *x* is the observed gene expression, μ is the mean gene expression, and *σ* is standard deviation of gene expression.

To further analyze and identify which genes contribute to rapid canopy cover related tissues we used the principal component analysis (PCA) unsupervised method. PCA of the gene expression were calculated using the *prcomp* function of the stats R package version 4.2.C (R Core Team, 2023). Parameters used for *prcomp* included *center* and *scale* set to *true*, meaning that the result is a correlation-based PCA. PCA retrieves loading segments through the linear combination of the gene expression values, thus reducing the dimensions of the data set. Additionally, PCA can be used to identify similarities and dissimilarities between genes and their contribution to each principal component and its respective loadings or eigenvectors. The dataset used in the PCA were all transcripts for which the median expression value for the fourteen tissues in question were equal or greater than 2, resulting in a total of 133 transcripts well expressed. PCA presented in this study show the predicted genes for the fourteen tissues, yet we show in supplemental data a PCA pertinent to the seven tissues important in aerial growth. PCA plots were built using the *ggbiplot* function of the ggbiplot R package version 0.55 (Vu and Friendly, 2024). Parameters used for *ggbiplot* included an ellipse set to *true* and *ellipse.prob* set to 70% confidence interval. Ellipses were used as a visual representation of gene expression (data points) dispersion within each group in the PCA. Additionally, ellipses are drawn based on the covariance structure of gene expression for each group, meaning that size and orientation of the ellipses are determined by the covariance matrix.

To further validate our analysis and provide additional information, we also calculated the tissue specificity index τ (tau) (Yanai et al., 2005; Kryuchkova-Mostacci and Robinson-Rechavi, 2017), using normalized gene expression data of the 133 transcripts:


$$\tau =\frac{\sum_{i=1}^{n}\left(1-\hat{x_{i}}\right)}{n\;-1};\;\hat{x_{i}}=\frac{x_{i}}{max_{1\leq i\leq n}\left(x_{i}\right)}$$

Here, *n* represents the number of tissues, $\hat{x_{i}}$ is the normalized expression of a gene in tissue *i* relative to the maximal expression values across all *i* tissues, and $x_{i}$ is the expression of a gene for each individual tissue, *i*.

Finally, we also tested for differences between gene family groups using the Kruskal-Wallis test with the R function *kruskal.test*. We tested the following hypotheses:

- H_0_: The three families are equal in terms of tau.
- H_1_: At least one gene family is different from the other two families in terms of tau.

Based on the Kruskal-Wallis test results, we conducted further analysis using pairwise *post hoc* tests to identify which groups were significantly different from one another. Specifically, we used the Wilcoxon test (with the *pairwise.wilcox.test* function) and the Dunn test (using the *ggbetweenstats* function from the ggstatsplot package (Patil, 2021).

## Results

### Phylogenetic analysis of TIR1/AFB co-receptors and proposed orthology based on *A. thaliana* classification

To facilitate discussion of comparative evolutionary and developmental roles of the nuclear auxin signaling gene families, we propose a nomenclature for *G. max* auxin signaling genes using a comparative phylogenetic approach with *A. thaliana*. This nomenclature aims to enhance the prediction of gene and protein functions in *G. max* by leveraging the extensive gene function knowledge available in *A. thaliana*. Several evolutionary and developmental comparative studies have shown that genes that share sequence similarity, and therefore fall within the same clade in a phylogeny, are broadly predicted to have similar function (Zhou et al., 2013; Hyung et al., 2014; Husbands et al., 2015; Damodharan et al., 2018; Zhang et al., 2021). Although comparative approaches have been used to identify and predict *G. max* gene function, little is known about the role of auxin signaling in *G. max* aerial architecture. In *A. thaliana* several auxin signaling genes are associated with unique aerial phenotypes of their mutants (Dharmasiri et al., 2005; Parry et al., 2009; Zhou et al., 2013; Husbands et al., 2015; Damodharan et al., 2018; Zhang et al., 2021). Therefore, we have assigned ortholog names for each *G. max* auxin signaling gene based on their phylogenetic placement in clades with *A. thaliana* genes, e.g. *GmTIR1/AFB1_A* is more closely related to (shares more sequence similarity with) *AtTIR1* and *AtAFB1* than other members of the *A. thaliana TIR1/AFB* family.

**Figure 1:**
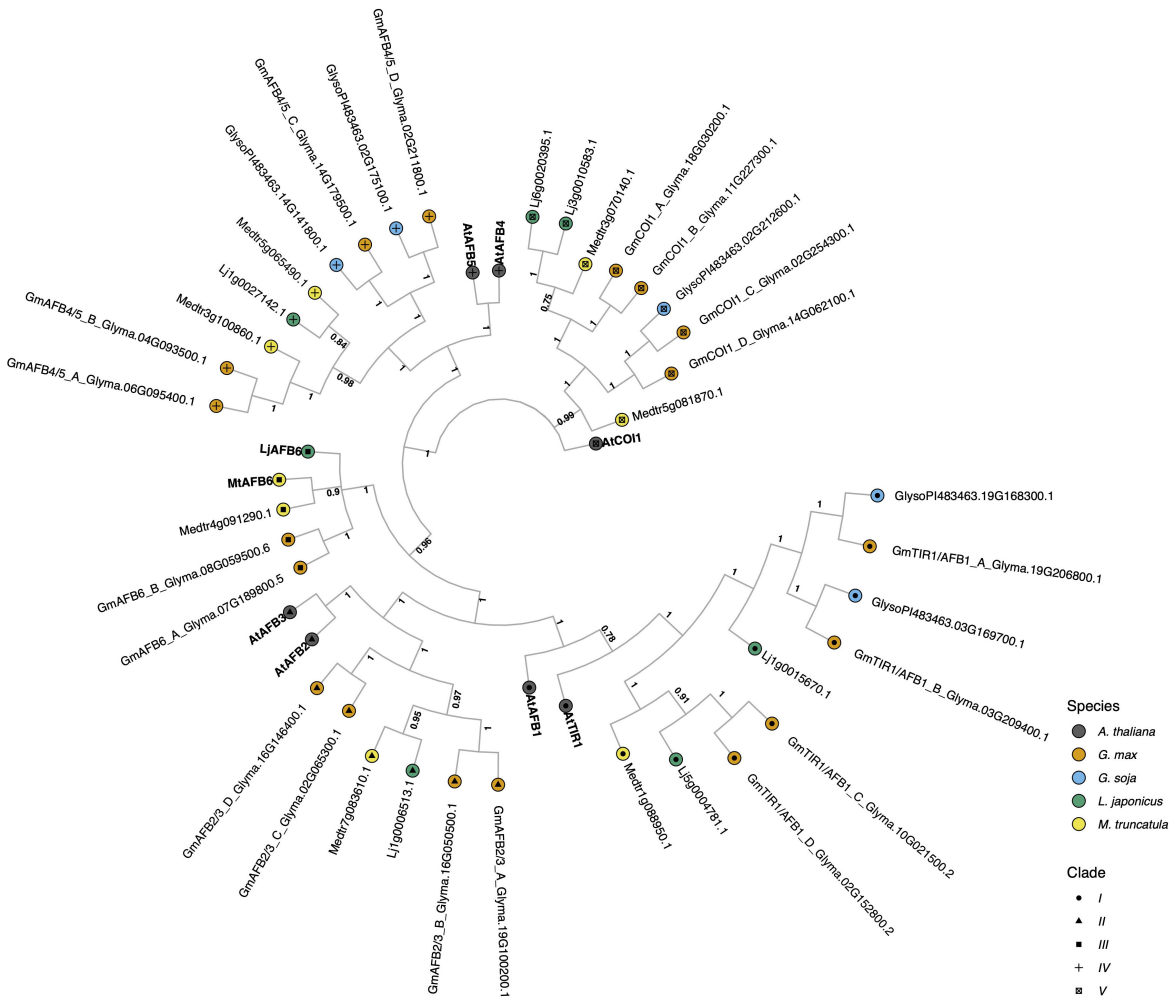
The evolutionary relationships between *G. max* TIR1/AFB proteins, and *A. thaliana* and other legume species orthologs. The historical relationship was inferred using MrBayes (Ronquist et al., 2012). The optimal tree was drawn according to the posterior probability of the evolutionary distances. The posterior probability of each node is labeled. Each tip is colored according to species, with *A. thaliana* in black, *G. max* in orange, *G. soja* in light blue, *L. japonicus* in green, and *M. truncatula* in yellow. *A. thaliana* gene symbols are displayed in bold to better visualize clade separation. Clades are also defined by the symbols inside the tips, with clade I as a circle, clade II as a triangle, clade III as a square, clade IV is a ‘+’ sign, and clade V as a boxed x. *G. max* genes were named according to their orthology to *A. thaliana and* gene ID.

**Figure 2:**
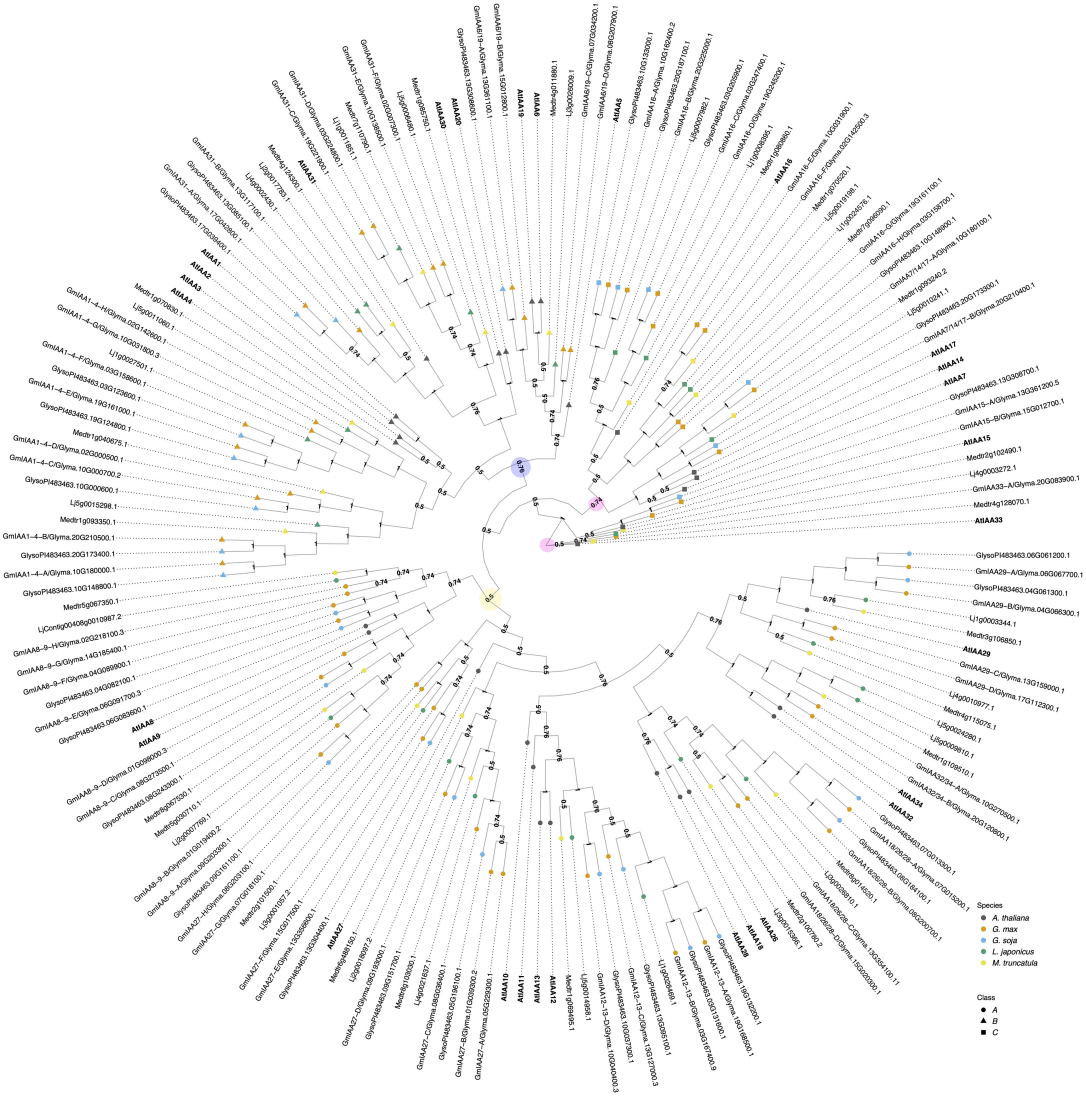
The evolutionary relationships between *G. max* Aux/IAA proteins, *A. thaliana* and other legume species orthologs. The historical relationship was inferred using the MrBayes (Ronquist et al., 2012). The optimal tree is drawn according to the posterior probability of the evolutionary distances. The posterior probability of each node is labeled. Each tip is colored according to species, with *A. thaliana* in black, *G. max* in orange, *G. soja* in light blue, *L. japonicus* in green, and *M. truncatula* in yellow. *A. thaliana* gene symbols are displayed in bold to better visualize assigned orthology. Aux/IAAs co-receptors are divided here into three classes: class A, represented as a circle; Class B, represented as a triangle, and class C, represented as a square. *G. max genes* were named according to both their orthology to *A. thaliana and* gene ID. Nodes are labeled with their supporting probabilities in the center. There are three defined clades, each highlighted with a colored circle. Clade I is marked in yellow, Clade II in blue, and Clade III in light pink. The light pink circles indicate the start of the root and the second defining point, showing that the remaining groups of genes belong to Clade III.

**Figure 3:**
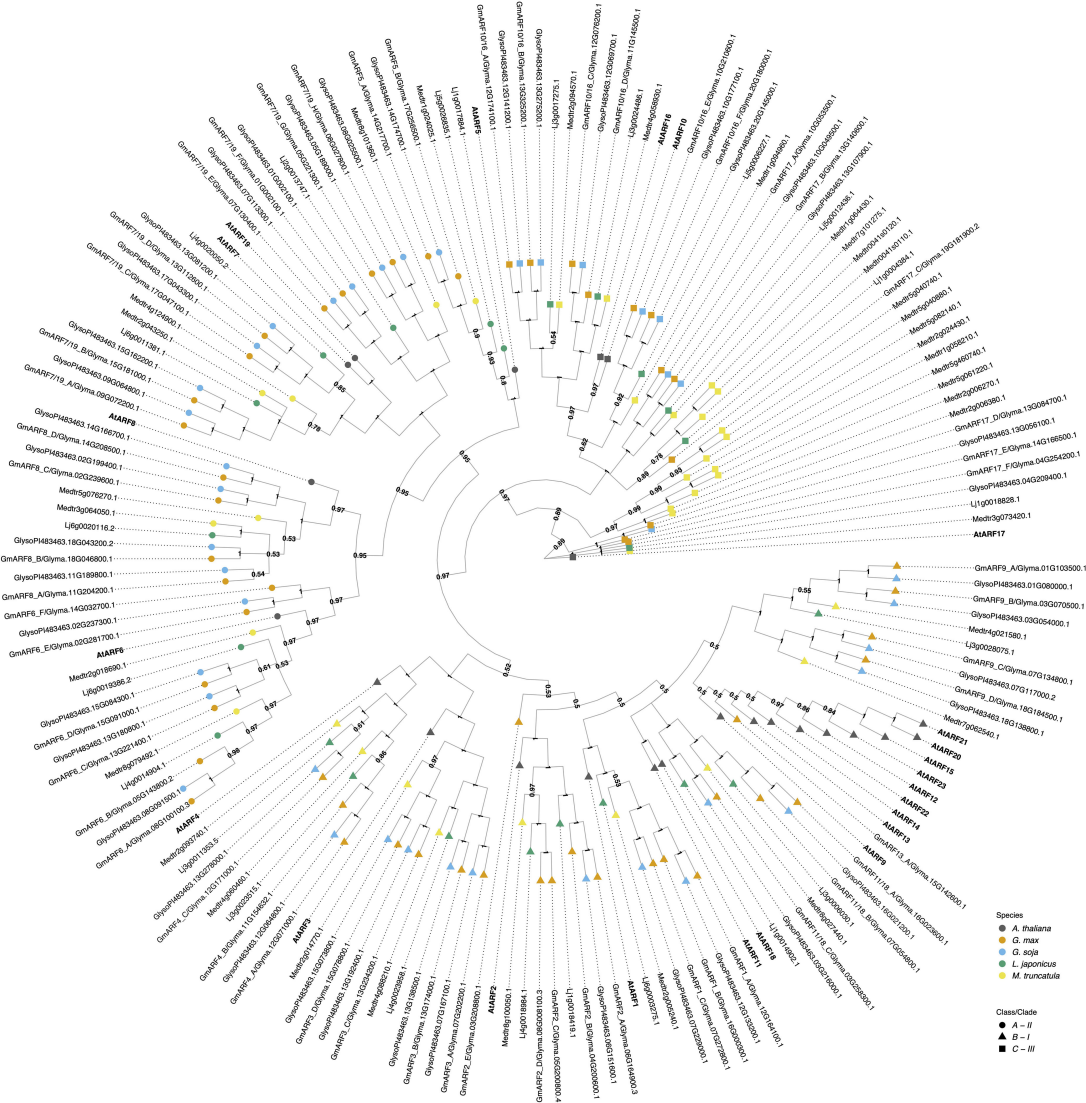
The evolutionary relationships between *G. max* ARFs proteins, *A. thaliana* and other legume species orthologs. The historical relationship was inferred using Bayesian Inference (Ronquist et al., 2012). The optimal tree is drawn according to the posterior probability of the evolutionary distances. The posterior probability of each node is labeled. Each tip is colored according to species, with *A. thaliana* in dark grey, *G. max* in orange, *G. soja* in light blue, *L. japonicus* in green, and *M. truncatula* in yellow. *A. thaliana* gene symbols are displayed in bold to better visualize assigned orthology. ARFs are divided here into three classes/clades (Ulmasov et al., 1999; Finet et al., 2013): Class A - II, represented as a circle; Class B - I, represented as a triangle, and class C - III, represented as a square. *G. max* orthologs were named according to both their orthology to *A. thaliana* and gene ID.

*G. max* has undergone two whole-genome duplications. The first corresponds to the early legume-duplication, which occurred approximately 59 million years (Myr) ago. The second duplication is Glycine-specific and happened around 13 Myr ago (Schmutz et al., 2010). As a result, *G. max* typically possesses more than one copy of each *A. thaliana* ortholog (**Figures 1**–**3**). Because of this, we have assigned letters to differentiate between *G. max* orthologs for each clade. Additionally, to strengthen our classifications of these *G. max* gene families and provide some additional context of these clades in the *Fabaceae*, we have also included other well studied legume species *G. soja*, *M. truncatula*, and *L. japonicus*.

The fourteen *G. max* TIR1/AFB auxin receptors identified were grouped into five main clades. Clade I, which has the least similarity to the COI1 (Coronatine Insensitive 1) jasmonate receptor outgroup, comprises *A. thaliana* TIR1-like proteins, with a posterior probability of 0.78 (the lowest probability found in the resulting tree) to its *G. soja*, *M. truncatula*, *L. japonicus*, and *G. max* orthologs. This clade consists of four *G. max* proteins *GmTIR1/AFB1_A–D*, of which only two (*GmTIR1/AFB1_A* and B) have a *G. soja* sister taxa, the other sister taxa were lost in *G. soja* (**Figure 1**). Interestingly this A/B subclade contains a *L. japonicus* taxa but is missing a *M. truncatula* taxa, suggesting a complex pattern of recent gene loss events in this clade. Clade II, consists of AFB2/AFB3-like proteins and also contains four *G. max* representatives but does not contain any *G. soja* and only one representative each from *M. truncatula* and *L. japonicus* (**Figure 1**). Clade III, is comprised by the AFB6-like proteins, Medtr8g098695.2 and Lj4g0012889.1 defined in (Rogato et al., 2021) and, according to (Parry et al., 2009) this clade was lost during the evolution of the Brassicaceae (*A. thaliana* family) and Poaceae families. We identified two *G. max* AFB6 orthologs. Interestingly, *G. soja*, *G. max*’s wild relative, lacks orthologs for both clades II and III, whereas *M. truncatula* and *L. japonicus* are both represented. Clade IV, AFB4/AFB5-like proteins, contains four *G. max* proteins, of which again only two have a corresponding *G. soja* sister as with TIR1/AFB1-like clade I. Lastly, clade V, comprises COI1-like F-box proteins, with four *G. max* homologs and only one representative sister in *G. soja*. The difference in number of genes between cultivated soybean, *G. max*, and its wild relative, *G. soja* is also in accordance with comparative genomics published data in which cultivated soybean has many unique genes that are unavailable in its wild relative (Joshi et al., 2013 and references therein). All the *G. max* orthologs here identified contain all the necessary functional domains to perceive auxin and associate with the SCF E3 ligase complex necessary for the turnover of Aux/IAA transcriptional repressors and auxin-mediated transcriptional response (**Supplementary Figure S4**; **Appendix A in Supplementary Data Sheet 2**).

### Phylogenetic analysis of Aux/IAA co-receptors and proposed orthology based on *A. thaliana* classification

Relative to the TIR1/AFB family, both the Aux/IAA and ARF families are far more numerous in all plants. Additionally, both have been more broadly studied in previous phylogenetic analyses. However, the names assigned to *G. max* genes/proteins are often not defined by orthology, making comparative inference difficult. To facilitate such analysis here and in the future, we have assigned *G. max* gene names for these families according to their orthology to *A. thaliana* (**Supplementary Table S1**).

The *G. max Aux/IAA* gene family contains sixty-one members. According to our inferred phylogeny, they are classified into three main groups: I, II, and III. This structure differs from the two main clades observed by (Hamm et al., 2019), while largely maintaining the same branch sister structure. Additionally, they are classified into three previously defined classes: A, B, and C. Classes A and B are paraphyletic when aligning *A. thaliana* genes alone and have conserved structural domains such as PB1, EAR motif, and degron domains. In contrast, class C genes lack one or more of these functional domains in their protein-coding sequences (Remington et al., 2004; Hamm et al., 2019) (**Figure 2**; **Appendix B in Supplementary Data Sheet 2**).

Clade I is composed of orthologs of AtIAA8/9/10/11/12/13/18/26/27/28/32/34, with at least two representatives in *G. max.* In contrast, its wild relative, *G. soja*, has one representative sister for each *GmIAA10-13* but lacks at least one sister in the remaining *G*. *max* orthologous groups. AtIAA8/9/27 belong to class A, AtIAA10/11/12/13/28 to class B, and AtIAA32/34 to class C (**Figure 2**).

Clade II, which consists of orthologs of AtIAA1/2/3/4/5/6/19/20/30/31, is distributed similarly to Clade I. However, all orthologous groups are missing at least one sister in *G. soja.* AtIAA1/2/3/4/5/6/19 are classified as class A Aux/IAAs, AtIAA20/30 belong to class B, and AtIAA31 belongs to class C. Classes A and B are structured within the same clade as observed by (Hamm et al., 2019). AtIAA31, along with AtIAA20/30 previously mentioned as part of Clade I, are clustered in our results within Clade II (**Figure 2**). This clustering shows slight differences compared to (Hamm et al., 2019) and (Remington et al., 2004). These differences are likely due to the complexity of our multi-species phylogeny.

Clade III is composed by AtIAA7/14/15/16/17/33 orthologs (**Figure 2**). AtIAA33 was defined as the root of our phylogeny. AtIAA7/14/15/16/17 belongs to class A, and AtIAA33 to class C. There are two *G. max* orthologs of AtIAA7/14/17, and each of them has an ortholog in G. soja. In contrast, orthologs of AtIAA15 and 33, have only one *G. max* ortholog. The *GmIAA15* has one G. soja ortholog, but *GmIAA33* does not have a *G. soja* ortholog. Although most species do have a representative, due to its paleopolyploidy, *G. max* has the most abundant number of Aux/IAAs when compared to other species analyzed here. In particular, the IAA16 orthology group contains eight *G. max* orthologs in two subclades of four, one sharing a more recent common ancestor with AtIAA16.

### Phylogenetic analysis of ARFs transcriptional factors and proposed orthology based on *A. thaliana* classification

The *ARF* gene family in *G. max* includes fifty-five members which can be separated into three functional classes, based on previous work with the Arabidopsis (Ulmasov et al., 1999; Tiwari et al., 2003; Finet et al., 2013; Le et al., 2016). Class A ARFs are likely transcriptional activators and orthologous to AtARF5/6/7/8/19 (**Figure 3**). The protein sequences of activator ARFs contain a glutamine-rich middle region that is associated with their transcriptional activation properties (**Appendix C**). Class A activator ARFs can be found under clade II.

Class B ARFs are traditionally defined as transcriptional repressors having a serine-rich middle region and comprise a large clade containing AtARF1/2/3/4/9/11/12/13/14/15/18/20/21/22/23. They are found under clade I. Finally, class C, also traditionally classified as transcriptional repressors, contains AtARF10/16/17 and is nearest the root of the tree (**Figure 3**) (Ulmasov et al., 1999; Finet et al., 2013). This is likely as the split between class C ARFs and A/B ARFs existed before the evolution of land plants (Ulmasov et al., 1999; Finet et al., 2013; Flores-Sandoval et al., 2018; Mutte et al., 2018). Class C repressor ARFs can be found under clade III.

As a result of *G. max* paleopolyploidy the majority of orthology groups have at least three *G. max* genes per corresponding *A. thaliana* ortholog (**Figure 3**). Additionally, all orthologs of ARF3, 8, 9, 10, 16, 7, and 19 *G. max* proteins have *G. soja* sisters, whereas the others lack at least one sister taxa in the wild relative. Interestingly, *M. truncatula* has ten orthologs of the class C AtARF17, which is the outgroup in our analysis. Five of these orthologs are on chromosome 5, suggesting this expansion is perhaps due to tandem duplications. Similarly in *A. thaliana* ARF9, 12, 13, 14, 15, 20, 21, 22, and 23 likely resulted from tandem duplication events, and the legumes have many fewer genes in this clade, following the more typical pattern of whole genome duplication events (Remington et al., 2004).

### Expression analysis and identification of auxin signaling targets for RCC development in *G. max*

**Figure 4:**
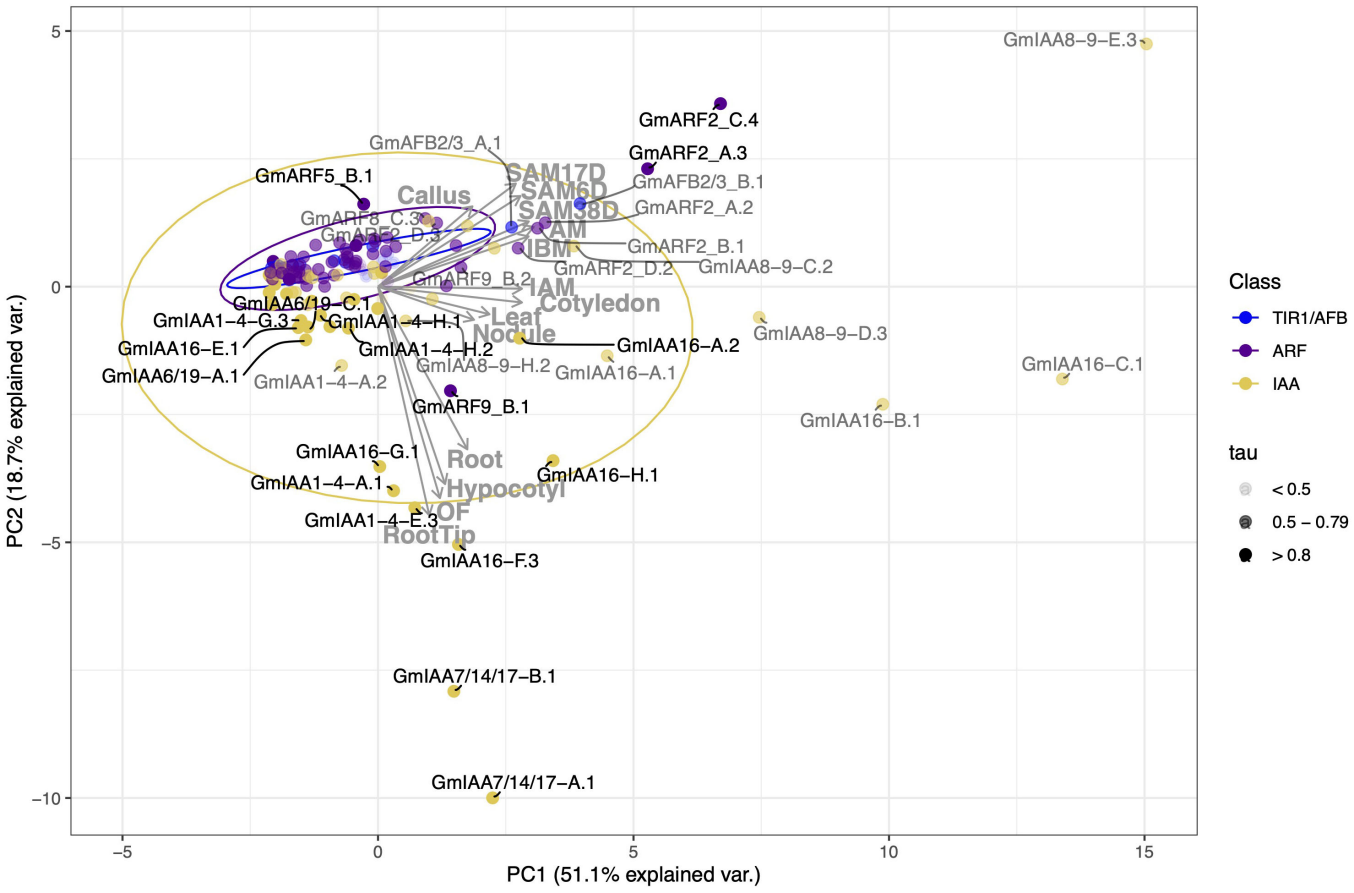
Correlation-based Principal Component Analysis (PCA): biplot of gene transcript expression and explanatory tissues involved in plant aerial architecture as eigenvectors (grey arrows, n = 14). Principal components 1 and 2 account for 69.8% of the total inertia. Ellipses are used here as a visual representation of dispersion of data points within each group (TIR1/AFB, ARF, and Aux/IAA (IAA)) with a 70% confidence interval. TIR1/AFB genes are colored cyan, ARF genes are colored purple, and Aux/IAA (IAA) genes are colored yellow. Some labels are connected to their respective points with curved solid lines. Color opacity of both data points and labels are determined by the gene tissue specificity index (tau), with tau < 0.5 represented in light grey, tau between 0.5 and 0.79 appearing as grey, and those with tau > 0.8 in black. Genes clustering together inside the ellipses, and/or having smaller tau values, are hypothesized to have more pleiotropic effects on plant growth and development. Conversely, genes associated with a specific RCC tissue (genes that fall along an eigenvector, outside of the respectively colored ellipse, and/or having intermediate to high tau) are hypothesized to have narrower effects and be more amenable to engineering RCC traits through gene editing.

First, we present our expression analysis using principal component analysis (PCA) with gene names presented as ortholog names from our phylogenetic analysis, below. Tissue-level gene expression data was retrieved from both (Wang et al., 2014; van Dijk et al., 2021) and SoyBase (further defined in the “Expression analysis data” section in methodology). A total of 133 of the 221 total transcripts for the combined TIR1/AFB, Aux/IAA, and ARF gene families displayed median expression equal to or greater than 2 TPM across all tissues (including open flower (OF), inflorescence before and after meiosis (IBM and IAM), callus, hypocotyl, cotyledon, root tip, axillary meristem (AM), shoot apical meristem at 6, 17, and 38 days (SAM6D, SAM17D, and SAM38D), root, young leaf and nodule tissues). The 133 transcripts were further evaluated through PCA (using the R built in function *prcomp* from stats package) and tissue specificity index analysis (tau) (Yanai et al., 2005) in order to identify which auxin regulatory genes are most specifically associated with certain RCC tissues. The first two principal components (PCs) account for 51.1% and 18.7%, respectively, of the total variation in gene expression (**Figure 4**). Therefore, the two-dimensional scatter-plot of the 133 given transcripts shown in **Figure 4** represents 69.8% of total variation.

Correlation between gene expression patterns in different tissues can be qualitatively assessed based on the angle and distance between their eigenvectors (grey arrows in **Figure 4**). Expression levels across meristematic tissues were strongly correlated and primary contributors to PC1 (**Figure 4**). Gene expression levels in leaf and cotyledon were strongly correlated and contributed weakly to PC2 and PC1 as denoted by its smaller eigenvectors. Expression levels in hypocotyl, root, and open flower tissues were also positively correlated with one another and contribute more to PC2. The relationship between hypocotyl and meristematic tissues ranges from no correlation to a weak negative correlation, probably due to tissue specificity of the Aux/IAAs. We observe a 90-degree angle between hypocotyl and axillary meristem (AM) tissues, representing no correlation between gene expression in these tissues.

To assess the variance and outliers in expression for each of these gene families, we drew ellipses representing 70% confidence intervals, assuming a Student’s T-distribution, in each PC for each gene family (in yellow, purple, and blue). Genes within these ellipses show weaker and perhaps less tissue-specificity in their expression. Variation in gene expression specificity across different gene families can be inferred from the area and shape of their respective ellipses. Our observations reveal that Aux/IAAs (yellow) exhibit a larger ellipse with approximately equal width along both PC1 and PC2 axes. This configuration signifies higher variability in expression between tissues within the Aux/IAA gene family. In contrast, ARFs (purple) and TIR1/AFBs (blue) display smaller, more elongated ellipses, particularly towards meristematic tissues. This suggests that these genes are less variable in expression and possibly share more functional overlap.

Genes clustered within ellipses, and close to the origin are more likely to display pleiotropic effects. This is suggested by the ellipses’ overlap in PCA space, as overlapping ellipses imply a high similarity between the expression of these genes and the tissues under analysis. Additionally, genes positioned closer to the origin make minor contributions to the variance explained by a principal component. While this could result from low expression levels, which reduces their impact on the analysis, it is not always the case. For example, *GmIAA7/14/17-A.1* has a median expression of 7.48 TPM and is highly expressed in root (94 TPM), root tip (214 TPM), hypocotyl (350 TPM), and open flower (536 TPM) but shows comparatively low expression in other tissues (30 TPM or less). Similarly, *GmARF9-B.2* has a median expression of 35 TPM and is located closer to the origin compared to ARF9-B.1, which has a median expression of 11 TPM. Thus, their proximity to the origin does not imply lower average expression levels.

**Figure 5:**
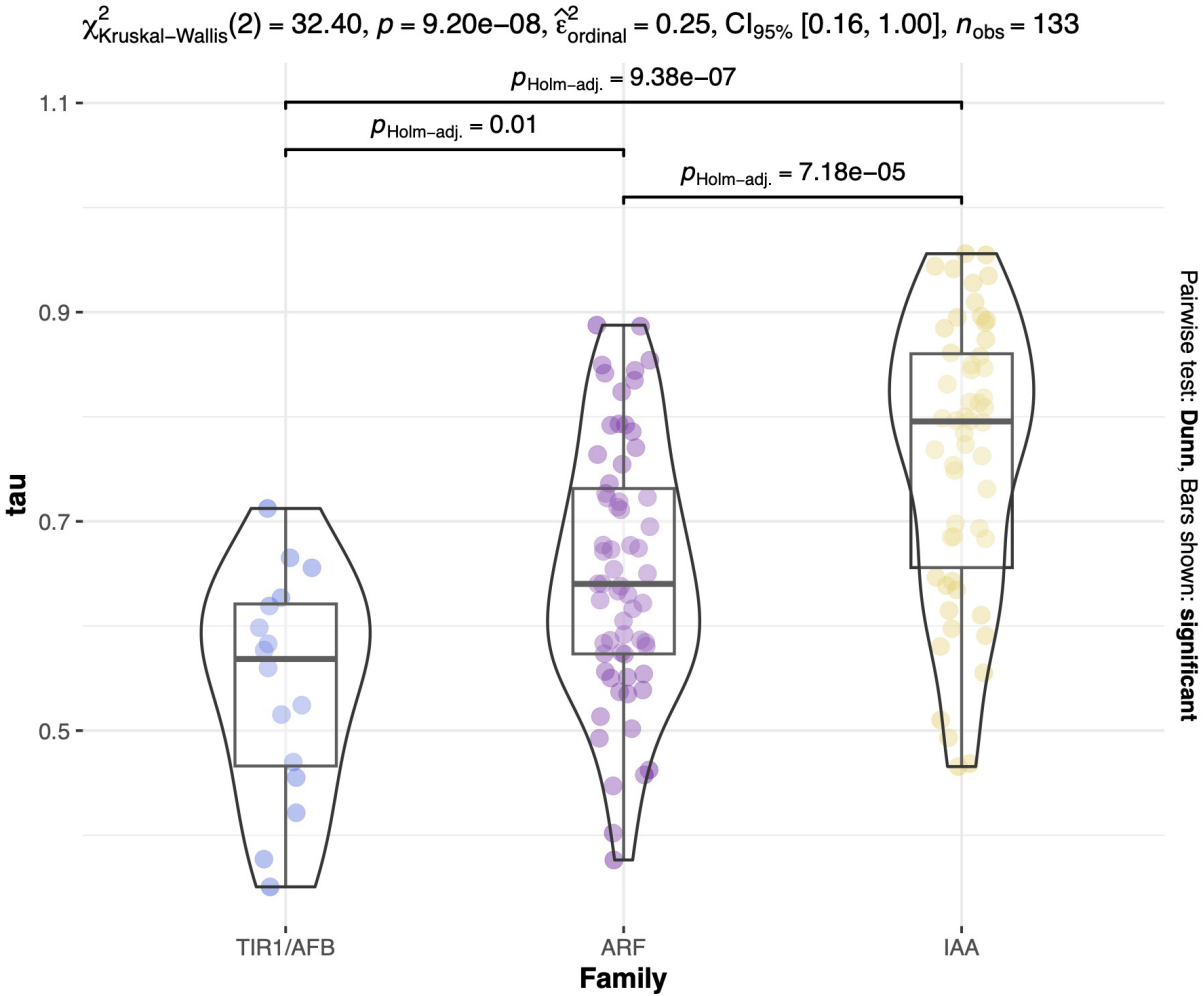
Distribution of tau values across auxin regulatory gene families. Kruskal-Wallis test of the 133 transcripts. The violin boxplots show the data distribution for TIR1/AFB represented in light blue, ARF in purple, and IAA in yellow. χ^2Kruskal-Wallis^(2) = 32.40, *p* = 9.20e^-08^, effect size = 0.25, and CI_95%_[0.18, 1.00]. *Post-hoc* test results between each gene family are indicated above each group with brackets. Tau values are displayed in the y-axis.

To further substantiate these observations, we examined the tissue specificity index, tau, a metric for evaluating tissue specificity in gene expression. This index ranges from 0 to 1, where 0 denotes no specificity and 1 indicates high specificity of a gene to a particular tissue (Yanai et al., 2005; Kryuchkova-Mostacci and Robinson-Rechavi, 2017). Similarly to the PCA results, we observed higher tau values for IAA transcripts, indicating greater specificity for these auxin repressors, followed by ARF and TIR1/AFB transcripts (**Figures 4**, **5**; **Supplementary Table S2**). The tau index median differs between groups based on the nonparametric Kruskal-Wallis test, which yielded a χ^2^ value of 32.4, a p-value of 9.20e-8, an effect size *w* of 0.25, and a 95% confidence interval of [0.16, 1.00] for the 133 observed transcripts. The Dunn *post hoc* test results indicate that the median tau index for IAAs differs significantly from those of ARFs and TIR1/AFB gene families (**Figure 5**). These findings align with the existing body of literature as well as with our PCA results, underscoring the idea that the regulation of auxin response may be tissue-dependent, primarily influenced by Aux/IAA repressor proteins.

Tissue specificity can also be qualitatively assessed based on the placement of genes with respect to the PCA eigenvectors and tau index. We defined genes falling outside the ellipses and having tau ≥ 0.8 as strongly associated with one or more tissues, as indicated by the eigenvectors of tissue expression also shown on the PC biplots. It is important to note that intermediate expression can also be a result of strong association in several tissues of interest and was oftentimes considered in a case-by-case basis depending on their position in PCA and what tissues impacted the overall index (refer to **Supplementary Table S2**).

**Figure 6:**
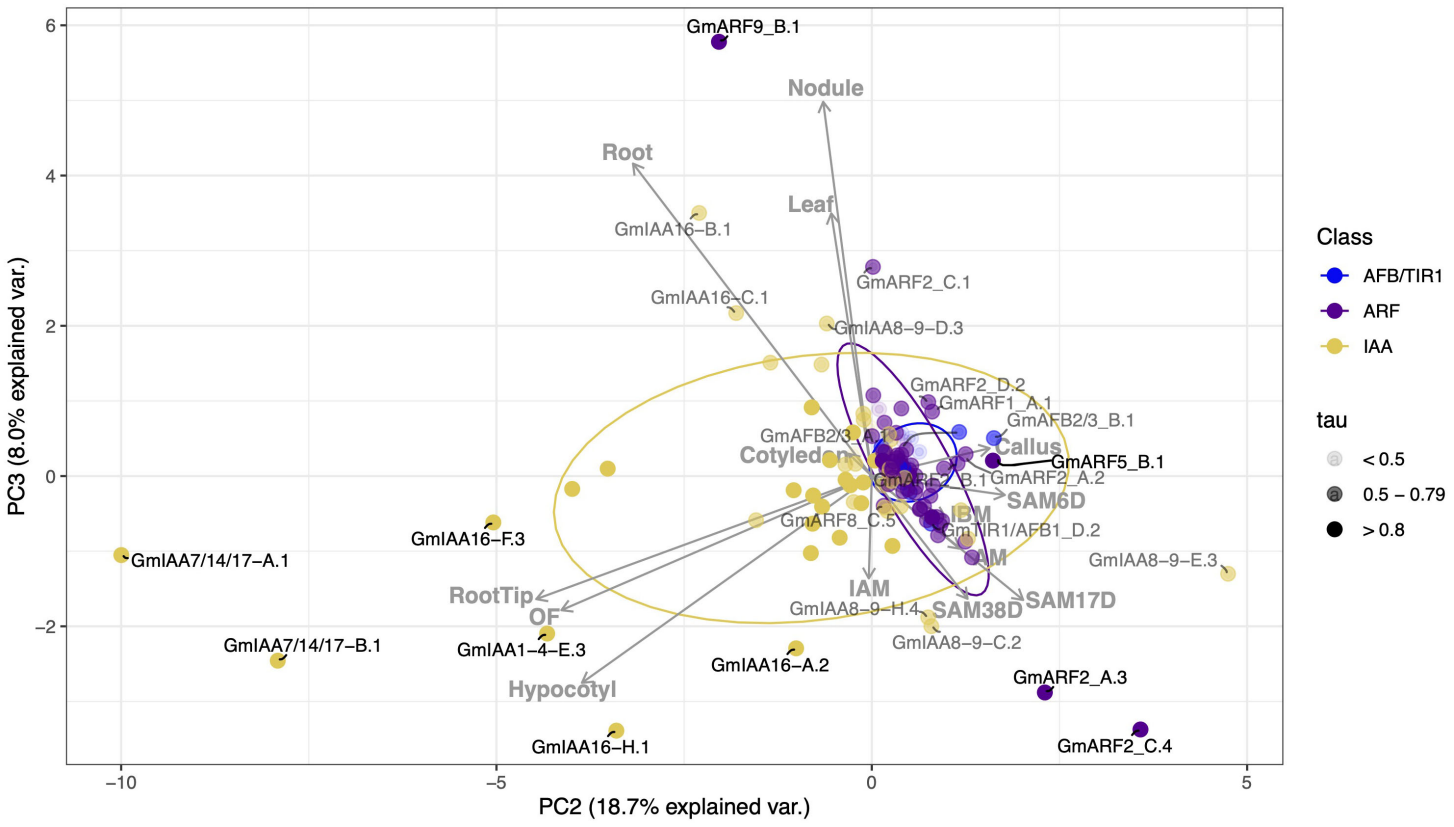
Correlation-based Principal Component Analysis (PCA): biplot of gene transcript expression and explanatory tissues involved in plant growth as eigenvectors (grey arrows), n = 14). Principal components 2 and 3 account for 26.7% of the total inertia. Both data points and label are also determined by the gene tissue specificity index (tau), with tau < 0.5 represented in light grey, tau between 0.5 and 0.79 appearing as grey, and those with tau > 0.8 in black.

Auxin regulatory genes associated with meristematic tissues were *GmARF2_A.3* (tau value of 0.82)*, GmARF2_C.4* (tau of 0.84), *GmARF8_C.3* (tau of 0.71)*, GmARF9_B.2* (tau of 0.55)*, GmARF11/18_A.2* (tau of 0.72), *GmARF11/18_B.1* (tau of 0.63), *GmARF11/18_B.4* (tau of 0.79), *IAA8-9_D.3* (tau of 0.64)*, GmIAA8-9_E.3* (tau of 0.76), *GmIAA16_B.1* (tau of 0.69), and *GmIAA16_C.1* (tau of 0.51) (**Figure 4**; **Supplementary Table S2**). There were four Aux/IAAs (*GmIAA7/14/17_A.1* (tau of 0.89)*, GmIAA7/14/17_B.1* (tau of 0.93)*, GmIAA16_G.1* (tau of 0.94)*, GmIAA16_H.1* (tau of 0.87)), three ARFs (*GmARF2_C.1* (tau of 0.55)*, GmARF8_C.1* (tau of 0.49), and *GmARF11/18_C.1* (tau of 0.69)), associated with at least one of the following hypocotyl, leaf, and cotyledon RCC-related tissues (**Figures 4**, **6**; **Supplementary Figure S1**). *IAA8-9_D.3, IAA16_C.1*, and *ARF2_C1* are examples of transcripts that display intermediate tau values, yet influence development in either or both, leaf, cotyledon and meristematic tissues. These transcripts fall between their respective tissue eigenvectors and can be found outside their respective ellipses. *GmARF11/18_A.2* and its paralogs are also examples of transcripts with intermediate tau values that influence one or more of leaf, cotyledon and/or meristematic tissues (**Supplementary Figure S1**; **Supplementary Table S2**). However, due to intermediate contributions to uncorrelated tissues, they are likely to fall close to the origin of our PCA, except for *GmARF11/18_A.2* observed in **Supplementary Figure S1**. Transcripts mentioned above were observed outside of the 70% confidence ellipses not only in the PC1 and PC2 biplot, but also in the PC2 and PC3 biplot (35% (n=7, tissues related to RCC) and 26.7% (n=14, all tissues) explained variance) as they provide additional information of possible candidate gene contributions to these aerial tissues (**Supplementary Figure S1**, **Figure 6**, respectively).

We observed fewer candidates from the *TIR1/AFB* family outside the corresponding ellipses, and tau values ranged from 0.3 to 0.72 (**Figure 4**; **Supplementary Table S2**). This aligns with their documented overlapping expression and function in Arabidopsis (Prigge et al., 2020). Despite somewhat overlapping expression across all tissues, *GmAFB2/3*_A.1 (tau of 0.59), *GmAFB2/3_B.1* (tau of 0.58), and *GmAFB2/3*_C.1 (tau of 0.61), displayed maximal component values ranging from 0.52 to 0.63 in AM, and 0.80 to 1 in SAM, have greater specificity to shoot apical meristem tissues than *GmTIR1/AFB1* orthologs. *GmTIR1/AFB1_A.1* (tau of 0.47), *GmTIR1/AFB1_B.1* (tau of 0.38), *GmTIR1/AFB1_C.1* (tau of 0.66), *GmTIR1/AFB1_C.2* (tau of 0.42), *GmTIR1/AFB1_C.3* (tau of 0.67), and *GmTIR1/AFB1_D.2* (tau of 0.55), displayed maximal component values ranged from 0.50 to 1 in AM, and 0.32 to 1 in SAM, however it shares relatively high and overlapping expression across other tissues when compared to *GmAFB2/3* paralogs (**Figure 4**; **Supplementary Figure S2**; **Figure 6**; **Supplementary Table S2**). *GmTIR1/AFB1* paralogs are also key regulators of leaf tissue, with component values ranging from as low as 0.16 to a maximum of 1. Although we observe paralogs of *GmAFB2/3* and *GmTIR1/AFB1* (**Figures 4**, **6**; **Supplementary Figure S2**) outside their corresponding ellipses, it remains unclear which receptors would be the best candidate genes due to their low tissue specificity (**Supplementary Table S2**) and likely pleiotropic nature. One possibility is that these auxin receptors may function in concert to control Aux/IAA levels freeing ARFs to mediate auxin signaling responsiveness. This idea is rather speculatory as to this point only receptor oligomerization to the SCF and dimerization of TIR1 receptors have been proposed (Dezfulian et al., 2016; Prigge et al., 2020). Further phenotypic characterization of mutants in these receptors is needed to understand how their extensive overlap in tissue expression in both soybean and Arabidopsis influences overall auxin signaling.

We have also compared our principal component analysis results using only the subset of 7 tissues that make up vegetative plant aerial architecture. We observe that excluding root, callus, and later developmental tissues did not alter the genes we found associated with RCC-related tissues. However, this exclusion did affect our ability to distinguish between genes that impact the tissues of interest and those that contribute to the aforementioned tissues (**Supplementary Figure S2**; **Supplementary Table S2**). Alternatively, we can delve into principal components that explain smaller variations that provide discrimination in the correlation between root, nodule, and hypocotyl tissues (**Figure 6**; **Supplementary Figure S3**). This provides further differentiation of the variance not explained by genes closer to the origin of PC1 and PC2, such as *GmTIR1/AFB1_D.2*, which show overlapping expression across various tissues (tau = 0.55) but are also significant contributors to meristems (with component values of AM = 0.65, SAM6D = 0.86, SAM17D = 1, SAM38D = 0.5). *GmTIR1/AFB1_C.2* is another example with an extremely low tau (0.21) due its broad and overlapping expression across tissues, but that also shows strong relationship with leaf (Rani et al., 2023), and meristems (AM = 0.75, SAM17D = 0.85, SAM38D = 0.59) (**Supplementary Figure S3**). This allows us to narrow down targets that might otherwise be considered only as broadly expressed and not contributors to the variance explained in PC1 and PC2.

## Discussion

Although the auxin signaling pathway is well studied and known to play an important role in plant growth, development, and architecture in *A. thaliana*, relatively little is known about auxin’s roles in legumes (Salehin et al., 2015; Li and Chen, 2023) outside root and nodule development and shoot height/dwarfing (Breakspear et al., 2014; Wang et al., 2015; Cai et al., 2017; Nadzieja et al., 2018; Schiessl et al., 2019; Wang et al., 2019; Gao, 2021; Rogato et al., 2021; Goto et al., 2022; Li and Chen, 2023). The extensive auxin regulatory gene families work together in a tissue-dependent manner (Piya et al., 2014) conducting shoot development and rosette area (Vernoux et al., 2011; Salehin et al., 2015; Prigge et al., 2020), organ primordia, as well as cell fate (Parry et al., 2009; Salehin et al., 2015; Rogato et al., 2021) in plants. Therefore, auxin’s mechanism of regulation is a great candidate for plant breeding programs (Li and Chen, 2023). However, what novel complexity and/or functional redundancy contained in each of these gene families remains a largely open question, especially outside of *A. thaliana*.

We have identified 14 *TIR1/AFB, 4 COI1-like F-box*, 55 *ARF*, and 61 *Aux/IAA* gene family members in *G. max* based on their similarity and evolutionary history relative to *A. thaliana* genes. The evolutionary history of the TIR1/AFB proteins exhibited 5 clades in for the *G. max* genes which were clustered equivalently to results reported for most *A. thaliana*, *L. japonicus* and *M. truncatula* in previous literature (Dharmasiri et al., 2005; Parry et al., 2009; Shen et al., 2015; Hamm et al., 2019; Rogato et al., 2021). To our knowledge, phylogenetic analysis of these TIR1/AFB auxin receptors found in the *G. max* genome has not yet been explored. We identified 61 *Aux/IAA* genes in *G. max*, which largely followed the clade structure of previous analyses (Remington et al., 2004; Liu et al., 2021; Ali et al., 2022). Similarly, we identified 55 *ARFs* in the *G. max* genome which also fell into orthology groups largely as expected based on previous analyses (Le et al., 2016). Our orthology naming convention, which has not to our knowledge been established before for soybean, facilitated comparative evolutionary analysis of these families with that of *A. thaliana*, providing some additional support for the genes we have identified as RCC-specific in our expression analysis.

Auxin transcriptional responses are governed by the degradation of *Aux/IAAs* in an auxin-dependent manner through interaction with *SCF^TIR1/AFB^* ubiquitin ligases and proteasomal degradation that releases class A activator *ARFs* from *Aux/IAAs* repression (Gray et al., 2001; Ramos et al., 2001; Zenser et al., 2001; Chapman and Estelle, 2009). Subsequently, different *TIR1/AFB-Aux/IAA-ARF* modules have been shown to regulate plant growth and development in a tissue-specific fashion in *A. thaliana* (Tatematsu et al., 2004; Vernoux et al., 2011; Piya et al., 2014; Krogan and Berleth, 2015; Galvan-Ampudia et al., 2020). We expected that the larger gene families in soybean would likely have more tissue specificity among members than *A. thaliana* and would therefore be less likely to result in pleiotropic phenotypes if mutated. In our expression analysis PC biplots, the proximity of *ARFs* and *TIR1/AFBs* to the origin along with smaller tau values (**Figure 4**) suggests their overlap in overall expression and involvement in diverse tissue processes. However, we did identify several members of these families with moderate to high tissue specificity. The *Aux/IAAs* exhibited greater dispersion and higher median tau values than the other families, implying greater tissue specificity, and possibly distinct auxin responses in different tissues. These differences in tau values are significant between groups (**Figure 5**), highlighting the overlap in the expression of auxin receptors and transcription factors compared to auxin repressors. This outcome is in-line with the expectation that larger gene families have a higher propensity for genetic drift and sub- and/or neo-functionalization (Birchler and Yang, 2022). However, much work remains in ascribing specific biological functions to modules composed of these auxin signaling gene families (Ori, 2019). Below, we discuss the functions and phenotypes associated with *A. thaliana* and legume orthologues of the *G. max* auxin signaling genes identified as potentially affecting RCC development.

### TIR1/AFB receptor genes important in G. max aerial architecture

In *A. thaliana*, the *TIR1/AFB* family members *TIR1*, *AFB1*, *AFB2* and *AFB3* are all shown to be expressed in rosette leaves and meristematic regions, however expression of *AFB2 and AFB3* transcript were the highest observed (Dharmasiri et al., 2005; Vernoux et al., 2011; Prigge et al., 2020). Additionally, AtAFB4 levels were nearly negligible whereas AtAFB5 is involved in Aux/IAAs turnover in shoot apical meristems (Vernoux et al., 2011). AFB5 orthologues in *A. thaliana* and *Pisum sativum* are involved in shoot branching and height (Prigge et al., 2016; Ligerot et al., 2017; Prigge et al., 2020). In *G. max*, *GmTIR1/AFB1*, *GmAFB2/3*, and *GmAFB4/5* orthologs are highly expressed in meristematic regions and leaves (**Supplementary Figure S5**; **Supplementary Table S2**). High maximal component values of *GmAFB4/5* orthologs are observed in meristems. However, these orthologs exhibit significant redundancy in expression domains (**Supplementary Table S2**; **Figures 4**, **6**). While additional research is required to comprehend the distinct roles of *GmTIR1/AFB1* and *GmAFB2/3* in soybean shoot architecture, speculation based on both PCA and tau values suggests that *GmAFB2/3* may be more conducive to engineering compared to *GmTIR1/AFB1*, due to higher tissue specificity specially in shoot apical meristem. *GmTIR1/AFB1* is expressed later in developmental stages and flower tissue, which may introduce undesirable effects on yield (**Figure 4**, **Supplementary Figure S2**; **Supplementary Table S2**). Given the extensive overlap in expression, we speculate the possibility of these receptors working cooperatively to regulate auxin response. This idea comes from known importance of ARFs and Aux/IAAs dimerization and oligomerization is modulating auxin signaling (Korasick et al., 2014). It may be possible that auxin receptors also dimerize or even form heterodimers to effectively control auxin responses.

### Aux/IAAs repressors important in *G. max* aerial architecture

As the largest gene family in the auxin signaling pathway, the Aux/IAAs contain the highest number of orthologs for each *A. thaliana* representative. We observed 10 soybean orthologs of *AtIAA16*, several of them highly and specifically expressed in hypocotyl, leaf, cotyledon, axillary and shoot apical meristems (**Figure 4**). Korasick et al. (Korasick et al., 2014) demonstrated that overexpression of the *Atiaa16-1* gain-of-function mutant stunts vegetative growth, which can then be rescued by knocking out a binding face of the Atiaa16-1 PB1 domain. Rinaldi et al. (Rinaldi et al., 2012) noted a dominant trait in *Atiaa16* gain-of-function mutants, which led to limited vegetative growth in adult plants. The high expression of soybean *AtIAA16* orthologs in hypocotyl, leaf, cotyledon, axillary and shoot apical meristems may regulate apical growth. In addition, *AtIAA16* is predicted to interact with *AtARF8*, which is also closely related to *ARF6* and therefore likely to share similar interaction patterns (Piya et al., 2014). Orthologs of *AtARF6* and *AtARF8* were also expressed highly in canopy cover associated soybean tissues, such as *GmARF6_C.2*, *GmARF8_A.1*, *GmARF8_A.2*, *GmARF8_C.3*, *GmARF8_C.5* (**Figures 4**, **6**; **Supplementary Figures S1**, **S6**). Thus, we postulate that At*IAA16* orthologs may also play a role in soybean auxin signaling and the associated phenotypes (**Figure 4**; **Supplementary Figure S1**; **Supplementary Table S2**). Examples of these *AtIAA16* orthologs are: *GmIAA16-A.1*, *GmIAA16-A.2, GmIAA16-A.3, GmIAA16-C.1*. Importantly, smaller PC values do not signify the absence of these genes’ influence on a phenotype. Particularly for auxin regulatory genes, which form complex dominance relationships have been previously described to form heterodimers and oligomerize, playing a crucial role in driving plant phenotype (Vernoux et al., 2011; Shen et al., 2015). Similarly, At*IAA7/14/17* orthologs are closely related to *AtIAA16* and interact with activator *ARFs* (Korasick et al., 2014; Piya et al., 2014). Several soybean orthologs of *AtIAA7/14/17* are predicted to be important in hypocotyl development in soybean based on their high expression levels (**Figure 4**). *AtIAA17* and *AtARF1* are predicted to affect hypocotyl development in *A. thaliana* (Piya et al., 2014). Similarly, we observe that soybean orthologs, such as *GmIAA7/14/17_A.1* and *GmIAA7/14/17_B.1*, are highly expressed in hypocotyl development in soybean. Although we did not identify *GmIAA29* as RCC specific through PCA, likely due to its high expression in many tissues, its upregulation has been associated with internode elongation in soybean. Notably, one ortholog, *GmIAA29_D.1*, has an intermediate tau of 0.72, with contributions to hypocotyl and open flower (maximum component values of 0.99 and 1, respectively). Additionally, it displays low to intermediate contributions, ranging from 0.34 to 0.72, across meristematic tissues. While this supports our algorithm for predicting pleiotropy, further development of this approach to the rational identification of candidate genes is still needed.

*Atiaa28* gain-of-function mutants show a strong phenotype for reducing apical dominance and plant size in *A. thaliana* (Rogg et al., 2001). Similarly, gain-of-function mutants of *Gmiaa27* are known to influence apical dominance and branching in soybean plants (Su et al., 2022). While we did not identify any orthologs of *AtIAA27* as strongly RCC related, again probably due to its redundancy in expression across several of the tissues analyzed, we observe that *GmIAA27-C.1*, and *GmIAA27-C.2* are highly expressed in meristems and hypocotyl (**Supplementary Figure S5**; **Supplementary Table S2**). Notably, there were seven *GmIAA27* orthologs, one with low tau (<0.5), four with intermediate tau (0.5 - 0.79), and two with high tau (>0.8). However, even orthologs with higher tau values, such as *GmIAA27-D.1* and *GmIAA27-F.1*, exhibit redundant expression across tissues that are not correlated with each other, resulting in their placement near the origin in our PCA.

*SlIAA19* has been linked to multiple auxin signaling processes, such as apical dominance (Sun et al., 2013). It is possible that orthologs of these genes may serve as interesting targets as well. AtIAA26 (PAP1) in *A. thaliana* has also been linked to apical dominance due to loss of the trait after RNA silencing (Padmanabhan et al., 2005). Our expression analysis and heatmap (**Supplementary Figure S5**; **Supplementary Table S2**) show that soybean orthologs of *AtIAA18/26/28* are highly expressed in RCC related tissues. For instance, *GmIAA18/26/28_C.8* is strongly expressed in leaf tissue and *GmIAA18/26/28_C.6/C.11/D.1* in the shoot apical meristem. However, they also exhibit overlapping expression across at least one or more tissues such as IAM, IBM, and OF tissues and have intermediate tau values (0.5 - 0.79), thus placing them closer to the origin in the PCA. This underscores the importance of using tau for enhanced discrimination of tissue specificity in conjunction with PCA. It also highlights the need for developing better algorithms to handle ubiquitously expressed and potentially highly pleiotropic genes, as their discrimination remains imperfect and requires significant follow-up analyses as demonstrated here to further narrow down specific candidates. Moreover, intermediate expression profiles may contain important information about tissue-specific enhancement and suppression, as described by Yanai et al (Yanai et al., 2005). Perhaps binning by some summary statistic of phenotype-relevant tissue expression values prior to PCA and tau calculation would identify some additional candidates. Increasing sample size of transcriptome data in a spatial-temporal contest is also needed in order to precisely train new models and increase our power in identifying candidate genes.

Interestingly, most orthologs mentioned above are phylogenetically-related, reaffirming what was discussed by Piya et al. (Piya et al., 2014) that closely related proteins are prone to display similar modes of action. Most ARF family members can form complexes with most Aux/IAA family members interchangeably (Guilfoyle et al., 1998; Vernoux et al., 2011; Piya et al., 2014). The numerous paralogs stemming from soybean whole-genome duplication could also potentially play a role in interactions between and within ARFs, Aux/IAAs, as well as TIR1/AFBs (Shen et al., 2015). These interactions could potentially impact auxin response in different ways, perhaps even facilitating adaptation of these crop through neofunctionalization of these paralogs. Negative feedback in the greater auxin signaling network also prevents quantification of molecular function in planta. However, further investigation of transcriptome and molecular data on the interaction of these paralogs are needed to clarify the mechanism of specific ARF and Aux/IAA family members on transcriptional dynamics. Due to these confounding factors, exploring many Aux/IAA and ARF orthologs will be crucial for finding combinations that can be harnessed for rational engineering of plant growth.

### ARFs transcription factors important in *G. max* aerial architecture

*AtARF2, AtARF8*, and *AtARF9* orthologs stand out in our analyses as being highly and specifically expressed in RCC tissues in *G. max*. As stated above *GmARF2_A.3/C.4* and *GmARF9_B.2* were associated with meristematic tissues. *ARF8_C.3* also contributes to meristematic tissues, however with intermediate expression in IBM tissue. *ARF8_C.5* plays a major role in hypocotyl development, but due to its marginal intermediate (0.4 maximum component) contributions to several tissues it can only be further distinguished by its tissue specificity analysis and PC explaining smaller variances (**Supplementary Table S2**; **Figure 6**). *ARF8_C.1*, and *ARF2_C.1* are strongly associated with hypocotyl, leaf and cotyledon tissues. Described below, many of our findings are corroborated by existing literature by means of phenotypic and genomic analyses in *Arabidopsis, G. max*, and other related species. However, our analysis suggests that seemingly redundant *ARF* paralogs may have also evolved unique roles in *G. max.* Additionally, despite extensive genetic analyses of the *ARF* family (Okushima et al., 2005b), the delineation of *ARF* functionality in the SAM has been primarily limited to *ARF5*, while the involvement of other *ARF* genes is mostly supported by indirect evidence (Hardtke et al., 2004; Mallory et al., 2005). *AtARF2*, a class B ARF, is thought to serve as a negative regulator of cell proliferation and enlargement. In seedlings, *HOOKLESS1 (HLS1)* negatively regulates AtARF2 protein accumulation in the presence of ethylene, acting as a bridge between ethylene and auxin signaling. Ultimately, *AtARF2* plays a key role in apical hook formation (Li et al., 2004), supporting our data that suggest the association of *GmARF2_C.1* with hypocotyl tissues. Furthermore, *AtARF2* orthologs serve as a regulator of leaf senescence in both *G. max* and *Arabidopsis* (Lim et al., 2010; La et al., 2022). Mutations in *AtARF2* result in delayed leaf senescence by reducing the repression of auxin signaling and increasing auxin sensitivity (Lim et al., 2010). Mutations in *AtARF2*, akin to loss-of-function achieved through gene silencing, lead to elongated hypocotyls, darker green rosette leaves, and enlarged cotyledons, but do not impact global expression of auxin regulated genes in *A. thaliana* seedlings (Okushima et al., 2005a). *GmARF2_A.2/A.3* expression is upregulated in shaded *G. max* plants, contributing to leaf enlargement inhibition (Wu et al., 2017). While we do not observe high expression of *GmARF2_A.2/A.3* in leaf (**Supplementary Table S2**), that could be due to differences in experimental conditions through which samples analyzed here were collected. Expanding on transcriptome data from different environmental conditions could further help us to better understand the role of each of these orthologues. *AtARF2* has also been cited as playing a role in the SAM. For example, in Arabidopsis, SAM cells are maintained during embryogenesis by down-regulating AtARF2 activity (Roodbarkelari et al., 2015). This corresponds with the SAM tissue association of *GmARF2_A.2/A.3/*B.1/C.4/D.2 in our PC analysis.

*AtARF6/8* orthologs were the only class A ARFs that were strongly associated with any of the tissues in our analysis (**Supplementary Figures S1**, **S6**; **Supplementary Table S2**). Regulated by photoreceptors, CRY1 and phyB in *A. thaliana*, *AtARF6* and *AtARF8* in turn are associated with regulation of hypocotyl elongation under blue and red light. *AtARF8/ARF6* double null mutants also have reduced responses to environmental conditions. Far red light and elevated temperature exposure stunt hypocotyl elongation (Mao et al., 2020). AtARF8, in conjunction with AtARF6, indirectly mediates the expression of a key brassinosteroid biosynthetic enzyme in *A. thaliana*, which ultimately directs proximodistal cell expansion (Xiong et al., 2021). Leaf shape is primarily determined by proximodistal growth. Brassinosteroids also promote cell wall loosening which has been shown in simulations to lead to cell and organ growth, and thus modulate leaf roundness (Xiong et al., 2021). AtARF8 operates redundantly with AtARF6 to repress phloem proliferation and induce cambium senescence during the xylem expansion phase in the hypocotyl by interacting with DELLA proteins from the gibberellin signaling pathway. *AtARF8* and *AtARF6* also play essential roles in cambium establishment and maintenance (Ben-Targem et al., 2021). In *M. truncatula*, the *AtARF8* ortholog exhibits slightly elevated expression in the petiole and stem, but not the leaf (Liu et al., 2021). Similarly, our analysis found expression of *GmARF8_C.1* specific to hypocotyl, leaf and cotyledon tissues. We also discovered a strong association of *GmARF8_C.3* with the SAM, along with *GmARF6_C.2* and *GmARF8_A.1* with the meristem and leaf, respectively (**Supplementary Figure S6**; **Supplementary Table S2**). *AtARF6* and *AtARF8* are typically cited together due to their redundant expression domain and functionality, which is observed to some extent in our analysis of *G. max*.

One ortholog of *AtARF9*, *GmARF9_B.2*, another class B repressor, stood out in our analysis as potentially playing a role in shoot architecture. *GmARF9*, here *GmARF9_C.1* variant, is associated with promoting first pod height (Jiang et al., 2018). In *M. truncatula*, *MtARF9* has elevated expression levels in the leaves, shoots, and petioles among other tissues in the roots and seeds (Liu et al., 2021). There is otherwise a notable lack of literature that draws any meaningful connection between *AtARF9* orthologs and shoot architecture. Nonetheless, our PC analysis suggests that several of the *GmARF9* paralogs may serve distinct roles in the SAM and AM tissues. Importantly, they also exhibit marginal effects in inflorescence, with intermediate tissue contribution values often lower than those observed for the meristems. However (Vernoux et al., 2011), found that *AtARF9* has a fairly weak homogenous expression pattern in the *Arabidopsis* SAM, postulating that *AtARF9* likely does not play a significant role in that tissue at the time point examined. This discrepancy between the literature and our results could be explained by divergent roles of gene paralogs. Our analysis may also point to previously unknown functions of *GmARF9_B.2* and its role in regulating shoot architecture.

The above *AtARF2*, *AtARF8*, and *AtARF9* orthologs in *G. max* may serve as key targets in future studies exploring the developmental regulation of RCC. Further investigation is needed to clarify the roles of specific ARF-mediated transcriptional dynamics which is further confounded by the complex network of interactions with the large family of Aux/IAA proteins which play a key role in modulating unique transcriptional responses.

In conclusion, the findings presented in this study pinpoint potential auxin candidate genes that hold promise for improving RCC development in soybean. Specifically, diversifying the function of genes involved in early apical dominance such as those regulating meristematic tissues and hypocotyl, may enhance rapid canopy development. While we are enthusiastic about these results, we recognize the constraints of our analysis, primarily stemming from the limited available data. Increased resolution of single cell analysis or smaller bulk tissues as well as across developmental time would improve the conclusions of our analysis. It is clear from our results that the combined PCA and tau approach should be considered before drawing any conclusions as distance from the origin of PCA biplots and tau are not correlated. Thus, the addition of better suited datasets as well as development of a new machine learning model for predicting candidate genes may fast-forward this process. Additionally, synthetic biology approaches for functional characterization of soybean RCC-related TIR1/AFB-Aux/IAA-ARFs modules could be used to finely tune auxin responses and gain deeper insights into its intricate interaction network. Subsequently, functional variation in the candidate genes identified here could be studied *in planta* to the correlation of phenotypes with function in tissue specific auxin signaling modules.

## Data Availability

Publicly available datasets were analyzed in this study. This data can be found here: https://github.com/PlantSynBioLab/SoyARC_manuscript_public/releases/tag/Auxin.
