# Supplementary material for: Identification of potential auxin response candidate genes for soybean rapid canopy coverage through comparative evolution and expression analysis
Source: Front Plant Sci. 2024 Oct 3;15:1463438. doi: 10.3389/fpls.2024.1463438 (PMC11484095; doi:10.3389/fpls.2024.1463438)
Supplement: Supplementary file 1 [file DataSheet1.zip › AppendixA_AFB_myXStringSet_full_Alignment.pdf]

```
                                        20        40        60        80       100
Glyma.19G206800.1   -MQRMAYT---------------------------------FSFP----------------------------EEVLEHVFSFIWNERDRNAISLVCKSW   38
Glyma.03G209400.1   -MQKMAYT---------------------------------FSFP----------------------------EEVLEHVFSFIWSERDRNAISLVCKSW   38
     Lj1g0015670.1   -MKRMVCS-----------------------------------FP----------------------------EEVLEHVFSFIQVDTDRNAISLVCKSW   36
Glyma.10G021500.2   MRPRVNYS-----------------------------------FP----------------------------EEVLEHVFSFIECDKDRGSISLVCKSW   37
Glyma.02G152800.2   MRPRVAYS-----------------------------------FP----------------------------EEVLEHVFSFIECDKDRGSISLVCKSW   37
     Lj5g0004781.1   -MQRVAFS-----------------------------------LP----------------------------EEVLEHVFSFIDNDKDRSSISLVCRSW   36
   AT3G62980/TIR1   MQKRIALS-----------------------------------FP----------------------------EEVLEHVFSFIQLDKDRNSVSLVCKSW   37
   AT4G03190/AFB1   ----MGLR-----------------------------------FP----------------------------PKVLEHILSFIDSNEDRNSVSLVCKSW   33
Glyma.19G100200.1   ---MMNY------------------------------------FP----------------------------DEVIEHIFDYVVSHSDRNALSLVCKSW   33
Glyma.16G050500.1   ---MMNY------------------------------------FP----------------------------DEVIEHIFDYVVSHSDRNALSLVCKSW   33
     Lj1g0006513.1   ---MMNY------------------------------------FP----------------------------DEVIEHVFDYVVSHRDRNALSLVCKSW   33
Glyma.02G065300.1   ----MNF------------------------------------FP----------------------------DEVIEHIFDYVTSQRDRNDLSLVCKNW   32
Glyma.16G146400.1   ----MNC------------------------------------FP----------------------------DEVIGHIFGCVTSQRDRNAVSLVCKNW   32
   AT3G26810/AFB2   ----MNY------------------------------------FP----------------------------DEVIEHVFDFVTSHKDRNAISLVCKSW   32
   AT1G12820/AFB3   ----MNY------------------------------------FP----------------------------DEVIEHVFDFVASHKDRNSISLVCKSW   32
Glyma.07G189800.5   ----MECR-----------RKKENQKSN-------------STFP----------------------------DEVLERILGMLKSRKDKSTVSLVCKEW   44
Glyma.08G059500.6   ---MMECR-----------RKKENQNPN-------------STFP----------------------------DEVLERISGMLKSRKDKSTVSLVCKEW   45
Lj4g0012889.1/AFB6   ----MECRI----------RKKESPEPNILN--------QASPFP----------------------------DEVLERVLGMVKSRKDRSSVSLVCKEW   50
Glyma.06G095400.1   ----MKQI-----------------------------------------------DDGDDEQRSLSPLPEQVLENVLESVLHFLTSRRDRNAASLVCKSW   49
Glyma.04G093500.1   ----MNQI-----------------------------------------------DDG----RTLSPLPDQVLENVLESVLHFLTSRRDRNAASLVCKSW   45
     Lj1g0027142.1   ----MRDN--HPPPPTSDDDHRSSSLPDLARGGQITDSSSSKTRPGPGSSSSSLPQPGS-SSELQAQFPDQVLENVLENVLHFLSSRRDRNAASLVCRSW   93
Glyma.14G179500.1   ----MREK-ENHPSTNSEDDHRSS-LLDLAR-AEITESSNSKTR-----TCGSGLGSGSGTSEPQAPFPDQVLENVLENVLHFLSSRRDRNAASLVCRSW   88
Glyma.02G211800.1   ----MRDKSESHPSTNSEDDHRSSPLLDLAC-AEITESSNSKTR-----TCGPFPGSG--PSEPQAPFPDQVLENVLENVLHFLPSRRDRNAASLVCRSW   88
   AT5G49980/AFB5   ----MTQD---RSEMSEDDDDQQSPPLDLPS-TAIADPCSSSSSP----------NKSRNCISNSQTFPDHVLENVLENVLQFLDSRCDRNAASLVCKSW   82
   AT4G24390/AFB4   ----MTEE---DSSAKMSEDVEKYLNLNPPC-SSSSSSSSAATFT----------NKSRNFKSSPPPCPDHVLENVLENVLQFLTSRCDRNAVSLVCRSW   82

        Consensus   MXXXXXX++++++++++++++++++++++++-+++++++++++++----++++++++++++++++++++++++XXVXEXXXXXXXXXXDXXXXSLVCXXW   34

                                       120       140       160       180       200
Glyma.19G206800.1   YEIERWCRRKVFVGNCYAVSPLMVVKRFPEVRSIALKGKPHFADFNLVPDGWGGYVCPWIAAMARAFP-CLEEIRLKRMVITDESLELIAKSFKNFKVLV   137
Glyma.03G209400.1   YEIERWCRRKVFVGNCYAVSPLMVIKRFPELRSIALKGKPHFADFNLVPEGWGGYVCPWIAAMARAFP-CLEEIRLKRMVITDESLELIAKSFKNFKVLV   137
     Lj1g0015670.1   YEIERWCRRKVFVGNCYAVSPMIVIKRFPEVRSIALKGKPHFADFNLVPEGWGGYVCTWIAAMSRAFP-WLEEIRLKRMVISDESLELIAKSFKNFKVLV   135
Glyma.10G021500.2   YEIERWCRRRVFVGNCYAVSPATVVNRFPKVRSIAIKGKPHFADFNLVPEGWGAYVGPWIKAMAAAYP-WLQEIRLKRMVISDECLELIAKSFKNFQVLV   136
Glyma.02G152800.2   YEIERWCRRRVFVGNCYAVSPATVVNRFPKVRSIAIKGKPHFADFNLVPEGWGAYVGPWIKAMAAAYP-WLQEIRLKRMVIADECLELIAKSFKNFQVLV   136
     Lj5g0004781.1   YEIERCCRRNVFVGNCYAVSPEMVVKRFPRVRSVTLKGKPHFADFNLVPDGWGGYVSPWIKAMAAACP-SLLEIRLKRMVVTDDCLDLIAKSFKNFRVLV   135
   AT3G62980/TIR1   YEIERWCRRKVFIGNCYAVSPATVIRRFPKVRSVELKGKPHFADFNLVPDGWGGYVYPWIEAMSSSYT-WLEEIRLKRMVVTDDCLELIAKSFKNFKVLV   136
   AT4G03190/AFB1   FETERKTRKRVFVGNCYAVSPAAVTRRFPEMRSLTLKGKPHFADYNLVPDGWGGYAWPWIEAMAAKSS-SLEEIRMKRMVVTDECLEKIAASFKDFKVLV   132
Glyma.19G100200.1   YRIERCTRQRVFIGNCYSITPERLIQRFPGLKSLTLKGKPHFADFSLVPYDWGGFVHPWVEALAKSRV-GLEELRLKRMVVSDESLELLSRSFTHFKSLV   132
Glyma.16G050500.1   YRIERCTRQRVFIGNCYSITPERLIQRFPGLKSLTLKGKPHFADFSLVPYDWGGFVHPWIEALAKNKV-GLEELRLKRMVVSDESLELLSRSFTHFKSLV   132
     Lj1g0006513.1   YRIEKSTRKRVFIGNCYSISPERLIQRFPSLRSLTLKGKPHFADFSLVPLDWGGFVYPWIEALAKNKV-GLEELRLKRMVVSDESLELLSRSFVNFKSLV   132
Glyma.02G065300.1   HRLERCSRKSLFIGNCYSISPERVIERFPELKSLTLKGKPHFADFNLVPHGWGGFVSPWIEAMVKSRV-DLEELRLKRMVVTDKSLELLSRSFMNFKSLV   131
Glyma.16G146400.1   HRLERCCRKSLFIGNCYTISPERVIERFPELRSLTLKGKPHFPYFSLVPSGWGGFVAPWIEALARSRV-DLEELRLKRMVVSDESLELLSRSFVNFKSLV   131
   AT3G26810/AFB2   YKIERYSRQKVFIGNCYAINPERLLRRFPCLKSLTLKGKPHFADFNLVPHEWGGFVLPWIEALARSRV-GLEELRLKRMVVTDESLELLSRSFVNFKSLV   131
   AT1G12820/AFB3   HKIERFSRKEVFIGNCYAINPERLIRRFPCLKSLTLKGKPHFADFNLVPHEWGGFVHPWIEALARSRV-GLEELRLKRMVVTDESLDLLSRSFANFKSLV   131
Glyma.07G189800.5   FNAERWSRRSVFIGNCYSVSPEILTRRFPNIRSVTLKGKPRFSDFNLVPANWGADIHSWLVVFAEKYP-WLEELRLKRMTVTDESLEFLALKFPNFKALS   143
Glyma.08G059500.6   YNAERWSRRSVFIGNCYSVSPEILTRRFPNIRSVTLKGKPRFSDFNLVPANWGADIHSWLVVFAGKYP-WLEELRLKRMTVTDESLEFLALQFPNFKALS   144
Lj4g0012889.1/AFB6   YNAERWSRRSVFIGNCYSVSPEILTRRFPNIRSVTLKGKPRFSDFNLVPANWGADIRSWLVVFADKYP-LLEELRLKRMTVSDESLEFLALSFPYFKALS   149
Glyma.06G095400.1   YRAEALTRPDLFIGNCYAVSPRRATARFPRVRSLTIKGKPRFADFDLMPLNWGAHFTPWATALSQSYPSSLNKLHLKRMSLTDHDLILLSHSFPSFQDLV   149
Glyma.04G093500.1   YRAEALTRTELFIGNCYAVSPRRATGRFPRVRSVTIKGKPRFADFDLMPLNWGAHFTPWVTALSQSYHSSLNKLHLKRMSLTDHDLTLLSHSLPSFQDLL   145
     Lj1g0027142.1   CRAEALTRSELFIGNCYAVSPRRATARFSRVRSVTVKGKPRFADFDLMPDDWGAHFAPWATALAQAYP-WLEKIHLKRMSVTDEDLAVVADSFSGFRELV   192
Glyma.14G179500.1   YRAEALTRSELFIGNCYALSPTRATARFTRARSVTVKGKPRFADFDLMPADWGAHFSPWASALSQAYP-WLEKLHLKRMLLTDADLALIADSFAAFRELV   187
Glyma.02G211800.1   YRAEALTRSELFIGNCYALSPTRATARFTRVMSVTVKGKPRFADFDLMPPDWGAHFGPWASALAQAYP-WLEKLHLKRMLVTDADLALIADSFAGFRELV   187
   AT5G49980/AFB5   WRVEALTRSEVFIGNCYALSPARLTQRFKRVRSLVLKGKPRFADFNLMPPDWGANFAPWVSTMAQAYP-CLEKVDLKRMFVTDDDLALLADSFPGFKELI   181
   AT4G24390/AFB4   YRVEAQTRLEVFIGNCYSLSPARLIHRFKRVRSLVLKGKPRFADFNLMPPNWGAQFSPWVAATAKAYP-WLEKVHLKRMFVTDDDLALLAESFPGFKELT   181

        Consensus   XXXEXXXRXXXFXGNCYXXXPXXXXXRFXXXXSXXXKGKPXFXDFXLXPXXWGXXXXXWXXXXXXXXX+XLXXXXLKRMXXXDXXLXXXXXXFXXFXXLX   133

                                       220       240       260       280       300
Glyma.19G206800.1   LTSCEGFTTDGLAAIAANCRNLRELDLQESEVE--EDLSGHWLSHFPDS--YTSLVSLNISCLNNEVSLSALERLLGRCPNLRTLRLNRAVPLDRLPNLL   233
Glyma.03G209400.1   LTSCEGFTADGLTAIASNCRNLRELDLQESEV---EDLSGHWLSHFPDS--YTSLVSLNISCLNHEVSLSALERLLGRCRNLRTLRLNRAVPLDRLPNLL   232
     Lj1g0015670.1   LTSCEGFTTDGLAAIAANCRNLRELDLQESEV---EDLSGHWLSHFPDS--YTSLVSLNISCLSNEVSLSALERLLGRCPNMKTLRLNRAVPLDRLPNLL   230
Glyma.10G021500.2   LTSCEGFTTDGLAAIAANCRNLRELELRESEV---DDICGHWLSHFPDS--YTSLVSLNISCLGNEVNLSALERLVSRCPNLQTLRLNRAVPLDRLANLL   231
Glyma.02G152800.2   LTSCEGFTTDGLAAIAANCRNLRELELRESEV---DDICGHWLSHFPDS--YTSLVSLNISCLGNEVNLSALERLVSRCPNLQTLRLNRAVPLDRLATLL   231
     Lj5g0004781.1   LISCEGFTTHGLAAIAANCRNLRELDLRESEV---EDICGHWLSHFPDS--YNSLESLNISCLSNEVNLPALERLVSRCPNLQTLRLNRAVPLDRLTNLL   230
   AT3G62980/TIR1   LSSCEGFSTDGLAAIAATCRNLKELDLRESDV---DDVSGHWLSHFPDT--YTSLVSLNISCLASEVSFSALERLVTRCPNLKSLKLNRAVPLEKLATLL   231
   AT4G03190/AFB1   LTSCEGFSTDGIAAIAATCRNLRVLELRECIV---EDLGGDWLSYFPES--STSLVSLDFSCLDSEVKISDLERLVSRSPNLKSLKLNPAVTLDGLVSLL   227
Glyma.19G100200.1   LVSCEGFSTDGLAAIAANCRFLRELDLQENEV---EDHKGQWLSCFPDN--CTSLVSLNFACLKGEVSLGALERFVARSPNLKSLKLNRSVPVDALQRIM   227
Glyma.16G050500.1   LVSCEGFSTDGLAALAANCRFLRELDLQENEV---EDHKGQWLSCFPDN--CTSLVSLNFACLKGEVSLGALERLVARSPYLKSLKLNRSVPFDALQRIM   227
     Lj1g0006513.1   LVSCEGFTTDGLAAVAANCRFLRELDLQENEV---EDQKGQWLSCFPDS--CTSLVSLNFACLKGDVNLGALERLVARSPNLKSLRLNRSVPIEALQRIL   227
Glyma.02G065300.1   LVSCEGFTTDGLAAIAANCRFLKELDLQENEV---DDHRGQWLSCFPDC--CTSLVSLNFACLKGQINLGALERLVARSPNLKSLRLNHTVPLNALQRIL   226
Glyma.16G146400.1   LVRCEGFTTEGLAAIAANCRFLKDLDLHENVV---TDLKGQWLSCFPDC--CTSLVSLNFACLKGQINAGDLERLVARSPNLKSLRLNHTVPLSALQRIL   226
   AT3G26810/AFB2   LVSCEGFTTDGLASIAANCRHLRDLDLQENEI---DDHRGQWLSCFPDT--CTTLVTLNFACLEGETNLVALERLVARSPNLKSLKLNRAVPLDALARLM   226
   AT1G12820/AFB3   LVSCEGFTTDGLASIAANCRHLRELDLQENEI---DDHRGQWLNCFPDS--CTTLMSLNFACLKGETNVAALERLVARSPNLKSLKLNRAVPLDALARLM   226
Glyma.07G189800.5   LLSCDGFSTDGLASIATNCKNLTELDIQENGI---EDKSGNWLSCFPDS--FTSLEVLNFANLHNDVNFDALEKLVSRCKSLKTLKVNKSVTLEQLQRLI   238
Glyma.08G059500.6   LLSCDGFSTDGLASIATNCKNLTELDIQENGI---EDKSGNWLGCFPDS--FTSLEVLNFANLHNDVNFDALEKLVCRCKSLKTLKVNKSVTLEQLQRLL   239
Lj4g0012889.1/AFB6   LLSCDGFSTDGLAAIATSCKNLTELDIQENGI---DDKSGNWLSCFPES--FTSLEVLNFANLPNDVNSDALEKLVSRCKSLKTLKVNRSVTLEQLQRLL   244
Glyma.06G095400.1   LTCCEGFGTTGLAALTSNCRLLRVLELVECVVEV-GDEEMDWISCFPESDAQTHLESLVFDCVECPVNFDALERLVARSPLLRKLRLNRYVSMSQLHRLM   248
Glyma.04G093500.1   LTCCEGFGTTALAALASNCRLLRVLELVECVVEV-GDEELDWISCFPEIDAQTYLESLVFDCVECPINLEALERLVARSPSLRKLRLNRYVSMSQLHRLM   244
     Lj1g0027142.1   LVCCEGFGTRGLAAVASKCRLLRVLEVVESLVEPVSEDEVDWVSCFPET--QTHLESLVFDCVEYPVNFEALEGLVARSPGLKKLRLNRFVSMPQLYRLM   290
Glyma.14G179500.1   LVCCEGFGTPGLAVVVSKCRLLRVLELVESEV--EDDEEVDWISCFPES--QTNLESLVFDCVDVPINFEALEGLVARSPRLKKLRLNRYVSMAELYRLL   283
Glyma.02G211800.1   LVCCEGFGTPGLAAVASKCRLLRVLELVESVVEVEDDEEVDWISCFPET--QTNMESLVFDCVEVPINFEALEGLVARSPRLKKLRLNQFVSMAQLYRLL   285
   AT5G49980/AFB5   LVCCEGFGTSGISIVANKCRKLKVLDLIESEV---TDDEVDWISCFPED--VTCLESLAFDCVEAPINFKALEGLVARSPFLKKLRLNRFVSLVELHRLL   276
   AT4G24390/AFB4   LVCCEGFGTSGIAIVANKCRQLKVLDLMESEV---TDDELDWISCFPEG--ETHLESLSFDCVESPINFKALEELVVRSPFLKKLRTNRFVSLEELHRLM   276

        Consensus   LXXCXGFXTXGXXXXXXXCXXLXXLXXXEXXX+++XDXXXXWXXXFPXX++XTXLXXLXXXXXXXXXXXXXLEXLXXRXXXLXXLXXNXXVXXXXLXXXX   228

                                       320       340       360       380       400
Glyma.19G206800.1   LQCPQLVELGTGVYST-EM-------RPEVFSNLEAAFSGCKQLKSLSGFWDVLPSYLPAVYPICSRLTSLNLSYA-IIQSSDLIKLISQCPNLLRLWVL   324
Glyma.03G209400.1   LRCPQLVELGTGVYST-EM-------RPEVFSNLEAAFSGCKQLKSLSGFWDVLPSYLPAVYPICSRLTSLNLSYA-IIQSSDLIKLISQCPNLLRLWVL   323
     Lj1g0015670.1   RQCPQLVELGTGVYSA-EM-------RPDVFSNLAAAFSGCKQLKSLSGFWDVLPSYLPAVYPVCSGLTSLNLSYA-TVQSSDLVKLISQCPSLQRLWVL   321
Glyma.10G021500.2   RGAPQLVELGTGTYTT-EM-------RPEVFTNLAEAFSGCKQLKGLSGFWDVLPSYLPAVYPICSNLTSLNLSYA-TIQSPDLIKLVGQCESLQRLWVL   322
Glyma.02G152800.2   RGAPQLVELGTGAYTT-EM-------RPEVFTNLAEAFSGCKQLKGLSGFWDVLPSYLPAVYPICSNLTSLNLSYA-TIQSPDLIKLVGQCESLQRLWVL   322
     Lj5g0004781.1   RGAPQLVELGTGAYTA-EM-------RPEVLANLTEAFSGCKQLKGLSGFWDVLPSYLPAVYPVCSGLTSLNLSYA-TIQSPDHIKLVSQCGSLQRLWVL   321
   AT3G62980/TIR1   QRAPQLEELGTGGYTA-EV-------RPDVYSGLSVALSGCKELRCLSGFWDAVPAYLPAVYSVCSRLTTLNLSYA-TVQSYDLVKLLCQCPKLQRLWVL   322
   AT4G03190/AFB1   RCAPQLTELGTGSFAA-QI-------KPEAFSKLSEAFSNCKQLQSLSGLWDVLPEYLPALYSVCPGLTSLNLSYA-TVRMPDLVELLRRCSKLQKLWVM   318
```

```
       Glyma.19G100200.1   MRAPQLSDLGIGSLVH-DP-------ESEAYIKLKNTILKCKSITSLSGFLEVAPHCLAAIYPICPNLTSLNLSYAAGIQGSALVKLIHHCVKLQRLWIM   319
       Glyma.16G050500.1   MRAPQLSDLGIGSFVH-DP-------ESEAYIKLKNTILKRKSITSLSGFLEVAPHCLAAIYPICPNLTSLNLSYAAGIQGSDLIKLIRHCVKLQRLLIM   319
           Lj1g0006513.1   MQAPQLADLGIGSFVH-NP-------FSDAFSKLKNTILKSKSITSLSGFLEVGPFCLPAMYPICRNLTALNLSYAAGIHGNELIKLIYHCGKLQRLWIM   319
       Glyma.02G065300.1   RRAPQIVDLGIGSFIP-DP-------NSNVFIKLMNTIIECKSITSLSGFFEVTPRCLPAIYPVCMNLTAMNLSYAAGIQSRELIKLICRCGKLQRLWIM   318
       Glyma.16G146400.1   MQAPQLVDLGIGSFVF-DP-------RSEVYNNMKNAILKCMSITSLSGFFWVYPHCLSALYPVCMNLTTLNLRFAAGIQNTELIKLICCCGKLQRLSIM   318
         AT3G26810/AFB2   ACAPQIVDLGVGSYEN-DP-------DSESYLKLMAVIKKCTSLRSLSGFLEAAPHCLSAFHPICHNLTSLNLSYAAEIHGSHLIKLIQHCKKLQRLWIL   318
         AT1G12820/AFB3   SCAPQLVDLGVGSYEN-EP-------DPESFAKLMTAIKKYTSLRSLSGFLEVAPLCLPAFYPICQNLISLNLSYAAEIQGNHLIKLIQLCKRLQRLWIL   318
       Glyma.07G189800.5   VHVPQLGELGTGSFSQ-EL-------TSQQCSDLESALKNCKNLHTLSGLWVATAQYLPVLYSACTNLTFLNFSYA-PLDSDGLTKLLVHCPKLQRLWVV   329
       Glyma.08G059500.6   VHVPQLGELGTGSFSQ-EL-------TAQQCSDLESAFKNCRNLHTLSGLWVATAQYLPVLYSACTNLTFLNFSYA-PLDSDGLAKLLVHCPKLQRIWVV   330
  Lj4g0012889.1/AFB6   VRAPQLCELGSGSFSEDDL-------TAQQYSEIESAFNNCKNLHTLSGLWDPAEQYLPLLYPACTNLTFFNLSYA-PLDGDDLAKLLVHCPNLRRLWVV   336
       Glyma.06G095400.1   HRAPQLTHLGTGSFSASEL---------DQELDFASAFASCKSLVCLSGFREFWADYLPAIYPACANLISLNFSFA-DISADQLKSVIRHCHKLQTFWVL   338
       Glyma.04G093500.1   HRAPQLTHLGTGSFSASEL---------DQELDFASAFAACKSLVCLSGFREIWPDYLPAIYPACANLISLNFSYA-DISADQLISVIRHCHKLQTFWVL   334
           Lj1g0027142.1   LRAPQLTHLGTGSFSDSEA--------LEQEPDYASAFAACRSLVCLSGFREIWANYLPAILPVCANLTSLNLSYA-DVNADQLRSVICHCRKLQILWAL   381
       Glyma.14G179500.1   LRAPQLTHLGTGSFSATEAGAVGDQEPDYQEPDYAAAFEACRSLVCLSGFREIWADYLPAIYPVCTNLTSLNLSYA-DVNTDQLKSVIRHCHKLQIFWVL   382
       Glyma.02G211800.1   LRAPQLTHLGTGSFSATEAGVVG-----DQEPDYAAAFEACRSLVCLSGFREIWADYLPAIYPVCANLTSLNLSYA-DINTDQLKSVICHCHKLQIFWVL   379
         AT5G49980/AFB5   LGAPQLTSLGTGSFSHDEE------PQSEQEPDYAAAFRACKSVVCLSGFRELMPEYLPAIFPVCANLTSLNFSYA-NISPDMFKPIILNCHKLQVFWAL   369
         AT4G24390/AFB4   VRAPQLTSLGTGSFSPDNV------PQGEQQPDYAAAFRACKSIVCLSGFREFRPEYLLAISSVCANLTSLNFSYA-NISPHMLKPIISNCHNIRVFWAL   369

              Consensus   XXXPQXXXLGXGXXXX+XX++++--+++XXXXXXXXXXXXXXXXXXLSGXXXXXXXXLXXXXXXCXXLXXXNXSXA+XXXXXXXXXXXXXCXXLXXXXXX   317

                                          420                 440                 460                 480                 500
                           .........|.........|.........|.........|.........|.........|.........|.........|.........|.........|

       Glyma.19G206800.1   DYIEDAGLYALAASCKDLRELRVFPSDPFGL-EPNVALTEQGLVSVSEGCPKLQSVLYFCRQMSNAALHTIARNRTNLTRFRLCIIEPRTPDYLTHEPLD   423
       Glyma.03G209400.1   DYIEDAGLYALAASCKDLRELRVFPSEPFGL-EPNVSLTEQGLVSVSEGCPKLQSVLYFCRQMSNAALHTIARNRPNLTRFRLCIIEPRTPDYLTLEPLD   422
           Lj1g0015670.1   DFIEDAGLDVLAASCKDLRELRVFPSDPFGF-EPNVALTERGLVSVSEGCPKLQSVLYFCRQMSNAALHTIARNRPNFTCFRLCIIEPQAPDYLTLQPLD   420
       Glyma.10G021500.2   DYIEDAGLEVIAASCKDLRELRVFPSDPFGL-EPNVALTEQGLVSVSEGCTKLQSVLYFCRQMTNSALDTIARNRPNMTRFRLCIIEPQAPDHLTHQPLD   421
       Glyma.02G152800.2   DYIEDAGLEVIAASCKDLRELRVFPSDPFGL-EPNVALTEQGLVSVSEGCTKLQSVLYFCRQMSNAALDTIARSRPNMTRFRLCIIEPRAPDYLTHQPLD   421
           Lj5g0004781.1   DYIEDAGLDVLAASCKDLRELRVFPSDPFGL-EPNVALTEEGLISVSEGCPKLQSVLYFCRQMSNAALNTIAQNRPNMTRFRLCIIEPRTPDYLTHQPLD   420
         AT3G62980/TIR1   DYIEDAGLEVLASTCKDLRELRVFPSEPFVM-EPNVALTEQGLVSVSMGCPKLESVLYFCRQMTNAALITIARNRPNMTRFRLCIIEPKAPDYLTLEPLD   421
         AT4G03190/AFB1   DLIEDKGLEAVASYCKELRELRVFPSEPDLD-ATNIPLTEQGLVFVSKGCRKLESVLYFCVQFTNAALFTIARKRPNLKCFRLCVIEPFAPDYKTNEPLD   417
       Glyma.19G100200.1   DCIGDKGLGVVATTCKDLQELRVFPSVPFG---DPAAVTEKGLVAISMGCPKLHSLLYFCHQMTNAALITVAKNCPNFIRFRLCILDATKPDPDTMQPLD   416
       Glyma.16G050500.1   DCIGDKGLDVVATSCKDLQELRVFPSVPFG---NPAAVTEKGLVAISMGCPKLHSLLYFCHQMTNAALITVAKNCPNFIRFRLCILDATKPDPDTMQPLD   416
           Lj1g0006513.1   DCIGDKGLGIVASTCKELQELRVFPSAPFG---NPAAVTEKGLVAISAGCRKLHSLLYFCHQMTNAALITVAKNCPNFIRFRLCILDATKPDPDTMQPLD   416
       Glyma.02G065300.1   DCIGDFGLHVVASTCKDLQELRVFPSVRVGR-NDPAGVTEKGLVAISMGCPKLHSLLYFCQQMTNAALITVAKNCPNFIRFRLCILDPTKPDPDTVQPLD   417
       Glyma.16G146400.1   DCIGDNGLGVVAATCKDLQELRVFPVVRVGG-NGPTRVTEKGLVAISMGCPELHSLLYFCQQMTNAALITVAKNCPNFIRFRLCILDPTKPDPDTMQPLN   417
         AT3G26810/AFB2   DSIGDKGLEVVASTCKELQELRVFPSDLLG--GGNTAVTEEGLVAISAGCPKLHSILYFCQQMTNAALVTVAKNCPNFIRFRLCILEPNKPDHVTSQPLD   416
         AT1G12820/AFB3   DSIGDKGLAVVAATCKELQELRVFPSDVHGEEDNNASVTEVGLVAISAGCPKLHSILYFCKQMTNAALIAVAKNCPNFIRFRLCILEPHKPDHITFQSLD   418
       Glyma.07G189800.5   DTVEDKGLEAVGSHCPLLEELRVFPADPFDE-GIVHGVTESGFIAVSQGCPRLHYVLYFCRQMTNAAVATVVQNCPDFTHFRLCIMHPGQLDYLTQESMD   428
       Glyma.08G059500.6   DTVEDKGLEAVGSHCPLLEELRVFPADPFDE-GIVHGVTESGFIAVSQGCPRLHYVLYFCRQMTNAAVATVVQNCPDFTHFRLCIMHPGQPDYLTQESMD   429
  Lj4g0012889.1/AFB6   DTVEDKGLEAVGSHCPLLEELRVFPADPFEDEAIGHGVTESGFVALSRGCPRLHYVLYFCRQMTNAAVATVVQNCPDFTHFRLCIMNPGQPDYLTHEPMD   436
       Glyma.06G095400.1   DTICDEGLQAVAETCKDLRELRVFPVNTRE--EIEGPVSEVGFEAISRGCRKLQSILFFCQRMTNAAVVAMSNNCPDLVVFRLCIIGQYRPDPVTLEPMD   436
       Glyma.04G093500.1   DTICDEGLQAVAETCKDLRELRVFPVNTRE--EIEGPVSEVGFEAISRGCRKLQSILFFCQRMTNAAVVAMSNNCPDLVVFRLCIIGRYRPDPETLEPMD   432
           Lj1g0027142.1   DSIGDEGLQAVATTCHDLRELRVFPVDARE--ESEGPVSEVGFEAISKGCRKLESILFFCQRMTNAAVVAMSKNCPDLLVFRLCIIGRYRPDAVTQEPMD   479
       Glyma.14G179500.1   DSIRDEGLQAVAATCKDLRELRVFPVDARE--ETDGPVSEVGFEAISQGCRKLESILFFTQRMTNAAVVAMSKNCPDLVVFRLCIIGRYRPDPVTEEPMD   480
       Glyma.02G211800.1   DSIRDEGLQAVAATCKDLRELRVFPMDARE--ETDGPVSEVGFEAISQGCRKLESILFLCQRMTNAAVVAMSKNCPDLVVFRLCIIGRYRPDPVTQEPMD   477
         AT5G49980/AFB5   DSICDEGLQAVAATCKELRELRIFPFDPRE--DSEGPVSELGLQAISEGCRKLESILYFCQRMTNAAVIAMSENCPELTVFRLCIMGRHRPDHVTGKPMD   467
         AT4G24390/AFB4   DSIRDEGLQAVAATCKELRELRIFPFDPRE--DSEGPVSGVGLQAISEGCRKLESILYFCQNMTNGAVTAMSENCPQLTVFRLCIMGRHRPDHVTGKPMD   467

              Consensus   DXXXDXGLXXXXXXCXXLXELRXFPXXXXX+++XXXXXXEXGXXXXSXGCXXLXXXLXFCXXMXNXAXXXXXXXXPXXXXFRLCIXXXXXPDXXTXXXXD   414

                                          520                 540                 560                 580                 600
                           .........|.........|.........|.........|.........|.........|.........|.........|.........|.........|

       Glyma.19G206800.1   SGFGAIVEQCKDLQRLSLSGLLTDRVFEYIGTCGKKLEMLSVAFAGDSDLGLHHVLSGCDNLRKLEIRDCPFGDKALLANAEKLETMRSLWMSSCSVSYG   523
       Glyma.03G209400.1   SGFGAIVEQCKDLQRLSLSGLLTDRVFEYIGTYAKKLEMLSVAFAGDSDLGLHHVLSGCDNLRKLEIRDCPFGDKALLANAEKLETMRSLWMSSCSVSYG   522
           Lj1g0015670.1   SGFGAIVEHCKGLQRLSLSGLLTDRVFEYIGTYGKKLEMLSLAFAGDSDLGLHHVLSGCDNLKKLEIRDCPFGDKALLANAAKLETMRSLWMSSCSVSYG   520
       Glyma.10G021500.2   AGFGAIVEHCKDLQRLSLSGLLTDRVFEYIGTYGKKLEMLSVAFAGDSDLGLHHVLSGCDNLRKLEIRDCPFGDKALLANAAKLETMRSLWMSSCLVSYG   521
       Glyma.02G152800.2   AGFGAIVEHCKDLQRLSLSGLLTDRVFEYIGTYGKKLEMLSVAFAGDSDLGLHHVLSGCDNLRKLEIRDCPFGDKALLANAAKLETMRSLWMSSCLVSYG   521
           Lj5g0004781.1   AGFGAIVQHCKNLQRLSLSGLLTDRVFEYIGTYAKKLEMLSVAFAGDSDLGLHHVLSGCDNLRKLEIRDCPFGDKALLANAAKLETMRSLWMSSCLVSYG   520
         AT3G62980/TIR1   IGFGAIVEHCKDLRRLSLSGLLTDKVFEYIGTYAKKMEMLSVAFAGDSDLGMHHVLSGCDSLRKLEIRDCPFGDKALLANASKLETMRSLWMSSCSVSFG   521
         AT4G03190/AFB1   KGFKAIAEGCRDLRRLSVSGLLSDKAFKYIGKHAKKVRMLSIAFAGDSDLMLHHLLSGCESLKKLEIRDCPFGDTALLEHAAKLETMRSLWMSSCFVSFG   517
       Glyma.19G100200.1   EGFGAIVQSCRRLRRLSLSGKLTDQVFLYIGMYAEKLEMLSIAFAGDGDKGMLYVLNGCKKLRKLEIRDCPFGDMALLTDVGKYETMRSLWMSSCEVTVG   516
       Glyma.16G050500.1   EGFGAIVQSCRRLRRLSLSGQLTDQVFLYIGMYAEKLEMLSIAFAGESDKGMLYVLNGCKKLRKLEIRDCPFGNVALLTDVGKYETMRSLWMSSCEVTVG   516
           Lj1g0006513.1   EGFGAIVQSCKRLRRLSLSGQLTDQVFLYIGMYAEQLEMLSIAFAGESDKGMQYVLNGCKKLRKLEIRDCPFGNMALLADIGKYETMRSLWMSSCEVTVG   516
       Glyma.02G065300.1   EGFGAIVQSCKQLRRLSLSGQLTDQVFLYIGVYAEQLEMLSIAFAGESDKAMLYVLNGCKKLRKLEIRDSPFGDSALLMDVGKYETMRSLWMSSCDVTIG   517
       Glyma.16G146400.1   EGFGAIVQSCKQLRRLSLSGQLTDQVFLYIGMYAEQLEMLSVAFAGESDKAMLYVLNGCKKIHKLAIRGSPFGDSALLMDVGKYETMQFLWMTSCNVTVG   517
         AT3G26810/AFB2   EGFGAIVKACKSLRRLSLSGLLTDQVFLYIGMYANQLEMLSIAFAGDTDKGMLYVLNGCKKMKKLEIRDSPFGDTALLADVSKYETMRSLWMSSCEVTLS   516
         AT1G12820/AFB3   EGFGAIVQACKGLRRLSVSGLLTDQVFLYIGMYAEQLEMLSIAFAGDTDKGMLYVLNGCKKMRKLEIRDSPFGNAALLADVGRYETMRSLWMSSCEVTLG   518
       Glyma.07G189800.5   EAFGAVVKTCTKLQRLAVSGYLTDLTFEYIGKYAKNLETLSVAFAGSSDWGMRCVLDGCPKLRKLEVRDCPFGNGALLSGLGKYESMRSLWMSDCNLTMN   528
       Glyma.08G059500.6   EAFGAVVKTCTKLQRLAVSGYLTDLTFEYIGKYAKNLETLSVAFAGSSDWGMRCVLDGCPKLRKLEVRDCPFGNGALLSGLGKYESMRSLWMSDCNLTMN   529
  Lj4g0012889.1/AFB6   EAFGAVVKTCSKLQRLAVSGLLTDLTFEYIGKYAKNLETLSVAFAGSSDWGMECVLRGCPKLRKLEIRDCPFGNAALLSGLERYESMRSLWMSDCKLTMN   536
       Glyma.06G095400.1   EGFGAIVMNCKKLTRLAVSGLLTDRAFSYIGTYGKLIRTLSVAFAGDTDLGLQYVLQGCPNLQKLEIRDSPFGDGALHSGLHHFYNMRFLWMSSCKLTRQ   536
       Glyma.04G093500.1   EGFGAIVMNCKKLTRLAVSGLLTDRAFNYIGTYGKLIRTLSVAFAGDTDLGLQYVLEGCPNLQKLEIRDSPFGDGALRSGLHHFYNMRFLWMSSCKLTRQ   532
           Lj1g0027142.1   EGFGAIVMNCRKLTRLAVSGLLTDRAFEYIGRYGKLVRTLSVAFNGDTDLALKYVLEGCPNMQKLEIRDCPFGDAALRSGLHHYYNMRFLWMSNCKLTRQ   579
       Glyma.14G179500.1   EGFGAIVMNCKKLTRLAMSGLLTDRVFEYIGMYGKLVRTLSVAFAGDTDVGLKYVLEGCPNLQKLEIRDSPFGDGALRSGLHHYYNMRFLWMSSCKLTRQ   580
       Glyma.02G211800.1   EGFGAIVMNCKKLTRLAVSGLLTDRAFEYIGTYGKLVRTLSVAFAGDTDVGLKYVLKGCPNLQKLEIRDSPFGDGALRSGLHHYYNMRFLWMSTCKLTLQ   577
         AT5G49980/AFB5   EGFGAIVKNCKKLTRLAVSGLLTDQAFRYMGEYGKLVRTLSVAFAGDSDMALRHVLEGCPRLQKLEIRDSPFGDVALRSGMHRYYNMRFVWMSACSLSKG   567
         AT4G24390/AFB4   DGFGAIVKNCKKLTRLAVSGLLTDEAFSYIGEYGKLIRTLSVAFAGNSDKALRYVLEGCPKLQKLEIRDSPFGDVGLRSGMHRYSNMRFVWLSSCLISRG   567

              Consensus   XXFGAXVXXCXXLXRLXXSGXLTDXXFXYIGXXXXXXXXLSXAFAGXXDXXXXXVLXGCXXXXKLEXRDXPFGXXALXXXXXXXXXMRXXWMSXCXXXXX   514

                                          620                 640                 660                 680                 700
                           .........|.........|.........|.........|.........|.........|.........|.........|.........|.........|

       Glyma.19G206800.1   ACKLLGQKMPRLNVEVIDERG----PPDSRPDDCPVEKLYIYRTIAGPRLDMPGFVWT-------------------------------------ME---   579
       Glyma.03G209400.1   ACKLLGQKMPRLNVEVIDERG----PPDSRPDDCPVEKLYIYRTVAGPRLDMPGFVWT-------------------------------------ME---   578
           Lj1g0015670.1   ACKLLGLKMPKLNVEVIDERG----PPDSRPDSCPVEKLYIYRTIAGPRMDMPGFVYR-------------------------------------MED--   577
       Glyma.10G021500.2   ACKLLGQKLPRLNVEVIDERG----PPDSRPESSPVEKLYMYRTVSGPRLDMPGYVWR-------------------------------------MQ---   577
       Glyma.02G152800.2   ACKLLGQKMPRLNVEVIDERG----PPDSRPESSPVEKLYIYRTVSGPRLDMPGYVWR-------------------------------------MQ---   577
           Lj5g0004781.1   ACKLLGQKMPRLNVEVIDERG----PPDTRPESSPVEKLYIYRTISGPRVDMPGYVWT-------------------------------------ME---   576
         AT3G62980/TIR1   ACKLLGQKMPKLNVEVIDERG----APDSRPESCPVERVFIYRTVAGPRFDMPGFVWN-------------------------------------MD---   577
         AT4G03190/AFB1   ACKLLSQKMPRLNVEVIDEH-----PPESRPESSPVERIYIYRTVAGPRMDTPEFVWT-------------------------------------IHKNP   575
       Glyma.19G100200.1   ACKLLAKKMPRLNVEIFNENE---QEDCSLEDGQKVEKMYLYRTLAGKRKDAPEYVWT-------------------------------------L*---   573
       Glyma.16G050500.1   ACKLLAKKMPRLNVEIFNENE---QEDCSLEDGQKVEKMYLYRTLAGKRKDAPEYVWT-------------------------------------L*---   573
           Lj1g0006513.1   ACKELAEKMPRLNVEIFNENE---QEECSLEDEQSVEKMYLYRTLAGKRNDAPEYVCT-------------------------------------L*---   573
       Glyma.02G065300.1   ACKALAKKMPGLNVEIFNGNE---KVDRDVDDGQKVEKTYLYRTLVGRRKDAPEHVWT-------------------------------------L*---   574
       Glyma.16G146400.1   ACKALAEKMPRLNVEIFNENK---KVDRDVDDGQKVEKMYLYRTLAGRRKDAPELVWT-------------------------------------L*---   574
         AT3G26810/AFB2   GCKRLAEKAPWLNVEIINENDNNRMEENGHEGRQKVDKLYLYRTVVGTRMDAPPFVWI-------------------------------------L*---   576
         AT1G12820/AFB3   GCKRLAQNSPRLNVEIINENENNGMEQNEEDEREKVDKLYLYRTVVGTRKDAPPYVRI-------------------------------------L*---   578
       Glyma.07G189800.5   GVRLLAQEMPRLNVEVIKEES---------YETHQAKKVYVYRSVAGPRRDAPPFVLT-------------------------------------L*---   579
       Glyma.08G059500.6   GVRLLAKEMPRLNVEVIKEET---------YETHQAKKVYVYRSVAGPRRDAPPFVLT-------------------------------------L*---   580
  Lj4g0012889.1/AFB6   GCRLLAKEMPRLNVEVIKEEG---------CYEGPAQRVYVYRSVAGPRRDAPSFVLAHCGPHNARGSGKGSTTWSIVCTMQPYLVCYMRGYFQNL*---   624
       Glyma.06G095400.1   ACQEVAQTLPHLVLEVINSEE---------DKADGIEILYMYRSLDGPRDDAPKVVTI-------------------------------------LC---   587
```

```
   Glyma.04G093500.1   ACREVARMLPHLVLEVINSEE---------DKADDIEILYMYRSLDRPRDDAPKVVTI-------------------------------------LC---   583
     Lj1g0027142.1     ACQDVARALPHLVVEVINKED--------EAAVDDIELLYMYRSLDGPRDDAPQVVTI-------------------------------------LH---   631
   Glyma.14G179500.1   ACQEVARALPNLVLEVINNNNEENAGDEEENAGDEVETLYMYRSLDGPRDDAPRFVTI-------------------------------------LQ---   640
   Glyma.02G211800.1   ACQEVARVLPNLVFEVINNNSE-------ENAGDEVETLYMYRSLDGPRDDAPRFVTI-------------------------------------LQ---   630
      AT5G49980/AFB5   CCKDIARAMPNLVVEVIGSDD----D---DDNRDYVETLYMYRSLDGPRNDAPKFVTI-------------------------------------L----   619
      AT4G24390/AFB4   GCRGVSHALPNVVVEVFGADGDDDED---TVTGDYVETLYLYRSLDGPRKDAPKFVTI-------------------------------------L----   623

           Consensus   XXXXXXXXXPXLXXEXXXXXX+++++++++XXXXXXXXXYXYRXXXGXRXDXPXXVXX-------------------------------------X++++   564


                                         720
                       '''''''''|''''''''''|
   Glyma.19G206800.1   --DDSSLRLE*---------   588
   Glyma.03G209400.1   --DDSSLRLE*---------   587
     Lj1g0015670.1     --DDSALRLE*---------   586
   Glyma.10G021500.2   --DDSALRIS*---------   586
   Glyma.02G152800.2   --DDSALRIS*---------   586
     Lj5g0004781.1     --DDSAY-LK*---------   584
      AT3G62980/TIR1   --QDSTMRFSRQIITTNGL*   595
      AT4G03190/AFB1   ENGVSHLAIK*---------   586
   Glyma.19G100200.1   --------------------   573
   Glyma.16G050500.1   --------------------   573
     Lj1g0006513.1     --------------------   573
   Glyma.02G065300.1   --------------------   574
   Glyma.16G146400.1   --------------------   574
      AT3G26810/AFB2   --------------------   576
      AT1G12820/AFB3   --------------------   578
   Glyma.07G189800.5   --------------------   579
   Glyma.08G059500.6   --------------------   580
 Lj4g0012889.1/AFB6    --------------------   624
   Glyma.06G095400.1   ----------*---------   588
   Glyma.04G093500.1   ----------*---------   584
     Lj1g0027142.1     ----------*---------   632
   Glyma.14G179500.1   ----------*---------   641
   Glyma.02G211800.1   ----------*---------   631
      AT5G49980/AFB5   ----------*---------   620
      AT4G24390/AFB4   ----------*---------   624

           Consensus   ++++++++++XQIITTNGL*   574
```
