# Supplementary material for: Identification of potential auxin response candidate genes for soybean rapid canopy coverage through comparative evolution and expression analysis
Source: Front Plant Sci. 2024 Oct 3;15:1463438. doi: 10.3389/fpls.2024.1463438 (PMC11484095; doi:10.3389/fpls.2024.1463438)
Supplement: Supplementary file 1 [file DataSheet1.zip › AppendixB_IAA_myXStringSet_full_Alignment.pdf]

Positions 1–100

Sequence	Aligned residues	Count
Glyma.06G067700.1		0
Glyma.04G066300.1		0
AtIAA29		0
Glyma.13G159000.1		0
Glyma.17G112300.1		0
Glyma.10G270500.1		0
Glyma.20G120800.1		0
AtIAA34		0
AtIAA32		0
Glyma.07G015200.1	M WQRSHK	7
Glyma.08G200700.1	M WQRSHK	7
Glyma.13G354100.11	MNERSRD EASPQLLD LISNERE WQMNKRN	29
Glyma.15G020300.1	MDERSRN EASPQLLD LISNERE WQMNKRN	29
AtIAA26	MEGCPRNR EIGPKLLD LIPQGRK WYQEDKN	30
AtIAA18	MEGYSRNG EISPKLLDLMIPQERRN WFHDEKN	32
AtIAA28		0
Glyma.19G168500.1	MWVLPHLLVTTNEAPLL PKKYSLTSQ	26
Glyma.03G167400.9		0
Glyma.13G127000.3	MDEPHKPDQTCGPTLPNTLSF PLSLSDTLSLSHFHQHSHLTVPFIILFFF PQNYSIPFK	59
Glyma.10G040400.3	MKEPHEPDQTCGPTLPNALS PSLSHFHQHSHLTVPLIILFFFFC PQNYSIQFNSL	55
AtIAA12		0
AtIAA13		0
AtIAA11	MEGGSASGSASA LSNDENL	19
AtIAA10	MN GLQEVCSSSGSVMIGLPAEEDENA	26
Glyma.05G229300.1	MSEALED	7
Glyma.01G039300.2		0
Glyma.08G036400.1	MLALPCLLFFLYIIFFSSLKCNWPTLHNRAKRKASFNVVV ILQYHIFSFSFLICVVKQLKVVL MSEALEEV	71
Glyma.09G193000.1	MSVSLEQEG	9
AtIAA27	MSVSVAAEHD	10
Glyma.13G356600.1	MSRALEHD	8
Glyma.15G017500.1	MSRALEHD	8
Glyma.07G018100.1	MTTPLEHD	8
Glyma.08G203100.1	MTTPLEHD	8
Glyma.09G203300.1	MSPPTLVT EEEGR STVASDSSQSLDCFSQNGA GLKERN	38
Glyma.01G019400.2	MMSPPAVVT EEEGRSNVS STVASGSSQSLDRFSQNGA GLKERN	43
Glyma.08G273500.1	MSPPLLVTEEDEGQSNA SMVASASSPSSECFTLNEA RLKERN	42
Glyma.01G098000.3	MSPPLLVT EDEGQCHA SMVASASSPSLNCFSLNEA GFKECN	41
AtIAA9	MSPEEELQSNV SVAS SSPTSNCISRNTLG GLKEHN	35
AtIAA8	MSSGNDKIKQVL HIEDLMSYRLLSVDKDELVT SPCLKERN	40
Glyma.06G091700.3	MSPPVLSIGEEEGKSNV TLLVSSSTTMESVCLKSL EFKERN	41
Glyma.04G089900.1	MSPPVLSMGEEEGKSNV TLLGSSSTAMESVCLKSL EFKERN	41
Glyma.14G185400.1	MSKPLLGIGEEEGQSNV TLLVSSSVIMESVCLNSS KLKERN	41
Glyma.02G218100.3	MSKPLLGIAEEEGQSNV TLLVSSSATMESVCLNSS KLKERN	41
Glyma.10G180000.1		0
Glyma.20G210500.1		0
Glyma.10G000700.2		0
Glyma.02G000500.1		0
Glyma.19G161000.1		0
Glyma.03G158600.1		0
Glyma.10G031800.3		0
Glyma.02G142600.1		0
AtIAA4		0
AtIAA3	MGEKESVPTNVPKKMGHTFASKLLLLLPTSLAFFPLSLIQKTVSSLSTSIYLYSPLTCIKHSASSKPLIFIIS SFSISLL SFKDN	85
AtIAA2	MGGLKLRVPLLSPCLFLAFHASDNTSLSSFNPNFDAPLYISLS PFLQNSS	50
AtIAA1		0
Glyma.17G042800.1	MSPTLMANQNQ RECAECLTWLCFLLITLSLSFFLFCFK VASVVCIYKEPKVAAFLFSLDQFHSLEDMG TLEQIDA	75
Glyma.13G117100.1	MSDLALLLLIILSLSFFLFWYK VASVVYIYKDPKVAVFLFSLDQFHSLEDMG TLEQIDA	59
AtIAA31	M	1
Glyma.19G221900.1		0
Glyma.03G224800.1		0
Glyma.10G138500.1		0
Glyma.02G007300.1		0
AtIAA30		0
AtIAA20		0
Glyma.13G361100.1		0
Glyma.15G012800.1		0
AtIAA19		0
AtIAA6		0
Glyma.07G034200.1		0
Glyma.08G207900.1		0
AtIAA5		0
Glyma.10G162400.2		0
Glyma.20G225000.1		0
Glyma.03G247400.1		0
Glyma.19G245200.1		0
AtIAA16		0
Glyma.10G031900.1		0
Glyma.02G142500.3		0
Glyma.19G161100.1		0
Glyma.03G158700.1		0
Glyma.10G180100.1		0
Glyma.20G210400.1	MQPQAQVPQSYII EGYHTHSLSLVIYIAKQTKQHYNRKRTQSEREGSYSHKEKNF	55
AtIAA17		0
AtIAA14		0
AtIAA7		0
Glyma.13G361200.5		0
Glyma.15G012700.1		0
AtIAA15	MSPEEY	6
Glyma.20G083900.1	MDFRHL	6
AtIAA33	M	1
Consensus	MGEKXXXPXXXXXXXXXXXXXXXX++++++++++++++++++++++++++++++++++++++XXXXXXXX+XXXXXX+++++++++---+++++++++++	38

Positions 101–200

Sequence	Aligned residues	Count
Glyma.06G067700.1	MELESGL ALPTHNSVEEFKLN K	22
Glyma.04G066300.1	MEVDLVL ALPTQNSVEEFKLN K	22
AtIAA29	MELDLGL SLSPHKSSKLGFNFDL	23
Glyma.13G159000.1	MELQLGL ALSTHSRAYDQPFSHK	23
Glyma.17G112300.1	MELQLAL ALPI NPTYHK	17

```
  Glyma.10G270500.1  --------------------------------------------------------------------------------M-ESNTSSFLLN--------   11
  Glyma.20G120800.1  --------------------------------------------------------------------------------M-ESNTSRFLLN--------   11
            AtIAA34  -------------------------------MYCSDPPHPLH--------LVASDKQQ-------KDHKLLLSW--KKPTM-DSDPLGVFPN--------   43
            AtIAA32  --------------------------------------------------------------------------------MDPNTPADFFKG--------   12
  Glyma.07G015200.1  -----------------------------------------------------SSEE--------RKLELRLGP----PGEE-SLNESIRKCNRERRRNE   41
  Glyma.08G200700.1  -----------------------------------------------------SSEE--------RKLELRLGP----PGEE-SLNESIRKSNRE--RNE   39
 Glyma.13G354100.11  --------------------------------------------------EGKSSEE--------RKLELRLGP----PGEDWSLGGKMKNNNTE-REDQ   66
  Glyma.15G020300.1  --------------------------------------------------EGKSSEE--------RKLELRLGP----PGEDWSLGGKMK--NTE-RE-E   63
            AtIAA26  --------------------------------------------------NT--DQE--------KKLELRLGP----PGGDEEDHSAIKKKNTE-----   61
            AtIAA18  --------------------------------------------------SVFKTEE--------KKLELKLGP----PGEEDDDESMI-----------   59
            AtIAA28  ------------------------------------------------------MEEE-------KRLELRLAP----PCHQFTSNNNINGS--------   27
  Glyma.19G168500.1  --------------------------------------------------LETRAEK----PLQCLKMTTLLLRILHLTLN-------------------   53
  Glyma.03G167400.9  ---------------------------------------------------MSKDDN----SSSLDSSSSDLQLSLSLTL--------------------   25
  Glyma.13G127000.3  -FFHHPFSLFFLNSPKNHIFTVLIGFSACGSMST----------VSKDDNLVLSSED----SSCPEESELELGLGLSLSSGPSSKS--------------  130
  Glyma.10G040400.3  LILPLPLFVILLQFSQKSYFYTLSGFSACGSMST----------VSKDDNLALSSED----SSCPEESELELGLGLSLSSGSSKSH--------------  128
            AtIAA12  -------------------------------MRG----------VS-----ELEVGK----SNLPAESELELGLGLSLGG--------------------   30
            AtIAA13  -------------------------------------------MIT-----ELEMGK--------GESELELGLGLSLGGGTAAKI--------------   30
            AtIAA11  --------------------------------------------------VVSCED-----SSSPIGNELELGL--------------------------   38
            AtIAA10  --------------------------------------------------AHSSEDSS-CPDESVSETELDLAL--------------------------   49
  Glyma.05G229300.1  ----------------------------VPSMKGG-----------------C-EEEE--EGLNLKATELRLGL----PGCESPERE-------------   42
  Glyma.01G039300.2  ----------------------------------------------------------------------------------------------------    0
  Glyma.08G036400.1  ----------------------------VPSMKG--------------------GCEE--EGLNLKATELRLGL----PGCESPERE-------------  104
  Glyma.09G193000.1  ----------------------YVGLSEVPAMEG------------------CSERTG--GGLNLKATELRLGL----PGSESPEREEG----VEDKNV-   58
            AtIAA27  ----------------------YIGLSEFPTMEATTMS-----------DKTKTRDNN--NGLNFKATELRLGL----PGSESPER--------VDSRF-   62
  Glyma.13G356600.1  ----------------------YIGLAENPSMDG---------------------KNS--SSLNLKETELRLGL----PGCESPERKSGSALCLFGKELQ   59
  Glyma.15G017500.1  ----------------------YIGLAENPSMDGSSDK------------LSSEDGKT--SSLNLKETELRLGI----PGCESPERKSGSALCLFGKELQ   68
  Glyma.07G018100.1  ----------------------YIGLSEAPSMEKSCDKISSSV----SSNLSSEDENT--SSLNFKETELRLGL----PGSESPE-NNKLGISLFGKDLQ   75
  Glyma.08G203100.1  ----------------------YIGLSEAPSMEKSCDKISSSV----SSNLSSEDENTT-SSLNFKETELRLGL----PGCDSPENNNKSGVSLFGKDLQ   77
  Glyma.09G203300.1  ----------------------YLGLSDCSSVDSCA-----------STVPSLCDEKK--ENMNLKATELRLGL----PGFQSPEREPDLFS-LSSPKLD   98
  Glyma.01G019400.2  ----------------------YLGLSDCSSVDSSA-----------STVPSLCDEKK--ENMNLKATELRLGL----PGSQSPEREPDLFS-LSPAKLD  103
  Glyma.08G273500.1  ----------------------YLGLSDCSSVDS-------------SIVPSLSDEKK--ENLNLKATELRLGL----PGSQSPERDPDLFS-LSSTKLD  100
  Glyma.01G098000.3  ----------------------YLGLSDCSSVDS-------------STVPNLSDEKK--ENLNLKATELRLGL----PGSQSPERETELFS-LSSTKLD   99
             AtIAA9  ----------------------YLGLSDCSSVGS-------------STLSPLAEDDK--ATISLKATELTLGL----PGSQSPARDTELNL-LSPAKLD   93
             AtIAA8  ----------------------YLGLSDCSSVDS-------------STIPNVVGK----SNLNFKATELRLGL----PESQSPERETDFGL-LSPRTPD   96
  Glyma.06G091700.3  ----------------------YMGLSDCSSVDS--------------SVPSFSEETK--SNLNLKATELRLGL----PGSQSPDRDSDLCL-RSSTQFD   98
  Glyma.04G089900.1  ----------------------YMGSSDCSSVDS--------------SVPSFSEECK--SNLNLKATELRLGL----PGSQSPERDSDLCL-RSSTQLD   98
  Glyma.14G185400.1  ----------------------YMGLSDCSSVDS--------------SAPSFSDETK--SNLNLKATELRLGL----PGLQSPERDSDLCL-RSSIQFD   98
  Glyma.02G218100.3  ----------------------YMGLSDCSSVDS--------------SAPSFSDETK--SNLNLKATELRLGL----PGSQSPERDSDLCL-RSSIQFD   98
  Glyma.10G180000.1  ----------------------------------------------------------------------------------------------------    0
  Glyma.20G210500.1  ----------------------------------------------MENTTVTYQTD-----LNLKATELRLGL----PGTEESEE--------------   31
  Glyma.10G000700.2  ----------------------------------------------------------------------------------------------------    0
  Glyma.02G000500.1  -----------------------------------------------MESRVVFEND-----LNLKATELRLGL----PGTED-----------------   27
  Glyma.19G161000.1  --------------------------------------------------MGSFETE-----LNLKATELRLGL----PGCDETHDKS------------   29
  Glyma.03G158600.1  --------------------------------------------------MGSFETE----LMNLKATELRLGL----PGCDETNEKS------------   30
  Glyma.10G031800.3  --------------------------------------------------MGKYEKE-----LNLEATELRLGL----PGSDE-----------------   24
  Glyma.02G142600.1  ----------------------------------------------MENSLGKYGKE-----LNLEATELRLGL----PGSDE-----------------   28
             AtIAA4  -----------------------------------------------MEKVDVYDEL-----VNLKATELRLGL----PGTEE-----------------   27
             AtIAA3  ---------------------------------------NQK--LFFLSSPAILEEMD--EFVNLKETELRLGL----PGTDNVCEAK------------  126
             AtIAA2  ------------TSSPRTRTRRRRTRQEEQSTQENTQEGNTK--IDIDPKAMAYEKVN---ELNLKDTELCLGL----PGRTEKIKEE------------  117
             AtIAA1  --------------------------------------------------MEVTNG------LNLKDTELRLGL----PGAQEE----------------   24
  Glyma.17G042800.1  -------------------PQTQSSSSSSSSIDSNNNPSRTRTPPSSSPSSSLCRNRT---D---LSTDLRLGL--------------------------  124
  Glyma.13G117100.1  -------------------PQTRSSSSSSSSIDSNNHPSRTRTPPPSSPSSSACRNRT---D---LSTDLRLGL--------------------------  108
            AtIAA31  -------------------EVSNSCSSFSSSSVDSTKPSPSE-----------------------SSVNLSLSL--------------------------   33
  Glyma.19G221900.1  --------------------MGKPASSSSSSISSTTNRHLLL-----STASSLTQE---------LPTDLRLGL--------------------------   40
  Glyma.03G224800.1  --------------------MGKPASSSSSSISSTTNRRLLI-----STASSLTQQ---------LPTDLRLGL--------------------------   40
  Glyma.10G138500.1  --------------------MGKASSSSSSSISSCRNPSN-------STASSLTHQHS----QDHLRTDLRLGL--------------------------   43
  Glyma.02G007300.1  --------------------MGKASSSSSSSISSCRNPSNY------STASSLTHQHS---DQDHLRTDLRLGL--------------------------   45
            AtIAA30  --------------------MGRGRSSSSSSIESSCKSNPF------GVSSSNTRN---------LSTDLRLGL--------------------------   39
            AtIAA20  --------------------MGRGRSSSSSSIESSSKSNPF------GASSS-TRN---------LSTDLRLGL--------------------------   38
  Glyma.13G361100.1  -----------------------------------------------------MEKEA--VGLEIT-TELRLGL----PGGE------------------   22
  Glyma.15G012800.1  -----------------------------------------------------MEKEG--VGLEIT-TELRLGL----PGGE------------------   22
            AtIAA19  -----------------------------------------------------MEKE----GLGLEITELRLGL----PGRDVA----------------   23
             AtIAA6  -----------------------------------------------------MAKE----GLALEITELRLGL----PGDNYS----------------   23
  Glyma.07G034200.1  -----------------------------------------------------MAKE----GLGLEITELRLGL----PDAEH-----------------   22
  Glyma.08G207900.1  -----------------------------------------------------MAKE----GLGLEITELRLGL----PDAEHQ----------------   23
             AtIAA5  ---------------------------------------------------MANESN----NLGLEITELRLGL----PGDIVV----------------   25
  Glyma.10G162400.2  -------------------------------------------------------------MINFEETELRLGL----PGGSASDH--------------   21
  Glyma.20G225000.1  -------------------------------------------------------------MINFEETELRLGL----PGN-------------------   16
  Glyma.03G247400.1  --------------------------------------------------MEAERDKY--KMINFEETELRLGL----PLSGN-----------------   27
  Glyma.19G245200.1  --------------------------------------------------MEAEPDKY--KMINFEETELRLGL----PLSGN---E-------------   28
            AtIAA16  -------------------------------------------------------------MINFEATELRLGL----PGG----NH-------------   18
  Glyma.10G031900.1  --------------------------------------------------------------MVFEETELRLGLRLGLPGNGAAPT--------------   24
  Glyma.02G142500.3  --------------------------------------------------------------MVFEETELRLGLGLCLPGNGTTAT--------------   24
  Glyma.19G161100.1  --------------------------------------------------MEVGL-KK--ENMGFEETELRLGL----PGNGG---TE------------   28
  Glyma.03G158700.1  --------------------------------------------------MEVGLNKK--ENMGFEETELRLGL----PGNVGGTGTE------------   32
  Glyma.10G180100.1  ------------------------------------------------MTTMLTNEHG--LSLNLKETELCLGL----PGGGSE----------------   30
  Glyma.20G210400.1  QRGESKRVLLFI-------FYIYLGAKLCEFIDK--------------PEMATMLTKE--HGLNLKETELCLGL----PGGGGGGGGGGGGG--------  120
            AtIAA17  ------------------------------------------------------MMGS--VELNLRETELCLGL----PGGDT-----------------   23
            AtIAA14  --------------------------------------------------------------MNLKETELCLGL----PGGTET----------------   18
             AtIAA7  -------------------------------------------------------MIG--QLMNLKATELCLGL----PGGAEA----------------   23
  Glyma.13G361200.5  ---------------------------------------------------MSSQSDA--VGCNLKETELTLGL----PGTKT-----------------   26
  Glyma.15G012700.1  ---------------------------------------------------MSSESHA--VDCNLKETELTLGL----PGTKT-----------------   26
            AtIAA15  --------------------------------------------------VRVWPDSG-----DLGGTELTLAL----PGTPTNASEGPKKF--------   39
  Glyma.20G083900.1  --------------------------------------------------SNLSPHLW-------QDHRRQITS---APSTSFS----------------   30
            AtIAA33  --------------------------------------------------NSFEPQSQ-------DSLQRRFHQ---DNSTTQQPRDT------------   29

          Consensus  ++++++++++++++++++++++++++++++++++++++++++++++++++++++XX++--+++++XXXXXXLXX++++++++++++++++++++++++++   49


                                     220                 240                 260                 280                 300
  Glyma.06G067700.1  SKQIVSLELWR-----------------------------------------PSCGSDSEKRVKHNKR-----SFEESFG-------QFLK-----PFPL   64
  Glyma.04G066300.1  SKQIVSLQLWR-----------------------------------------ASCGSESEKRVKHNKR-----SFEESFG-------HFLK-----PLPL   64
            AtIAA29  ---------------------------------------------------NKHCAIEGAASCLGTEK----LRFEATFGLGNVEENCYMP------KQR   62
  Glyma.13G159000.1  ------------------------------------------------------------------KR-----TFSQLMMMEH-APF---------PHHV   42
  Glyma.17G112300.1  ------------------------------------------------------------------KR-----TFSQLLMMDHAAPPESTL-----TDHV   41
  Glyma.10G270500.1  -------------------------------------------------------SSTLHSVFYQDKQ---DDGIIDLGLSLG-------------TVQ-   39
  Glyma.20G120800.1  -------------------------------------------------------SSTLHSVFYQEKQ---DDGIIDLGLSLG-------------TVR-   39
            AtIAA34  -------------------------------------------------------SPKYHPYYSQTTE---FGGVIDLGLSLR-------------TIQ-   71
            AtIAA32  -------------------------------------------------------SSKFHTYYSQTKK---GGGVIDLGLSLR-------------TIQ-   40
  Glyma.07G015200.1  SPLTLGCFSTQ---KIFTSDTPGPGGAMLPSAWPSTSYHHQ-DQAKAKASSFLQLQSSPQNMILMGKD------VSQFS-----------------CVE-  113
  Glyma.08G200700.1  SQFTLGCFSTQ---NFFTSDKQGPGGTTLPSAWPSTSYHHQ-HQAKAKASSFLQLQSSPQNMIVMGKD------VSQFS-----------------CVE-  111
 Glyma.13G354100.11  SLLSLGCFSPNINSNAFQSKASH--------PWPNYHHHGQGNNKKASSSSFLQFPSSTQ-PVMMGKDASCPKVVVELQQ-----------N---GGD-  141
  Glyma.15G020300.1  SLLSLGCFSPN---NGFQSKAS---------PWPNYHHHHQGNNNKASSSSFLQ----------------CPKVVVELQ------------N---GGD-  118
```

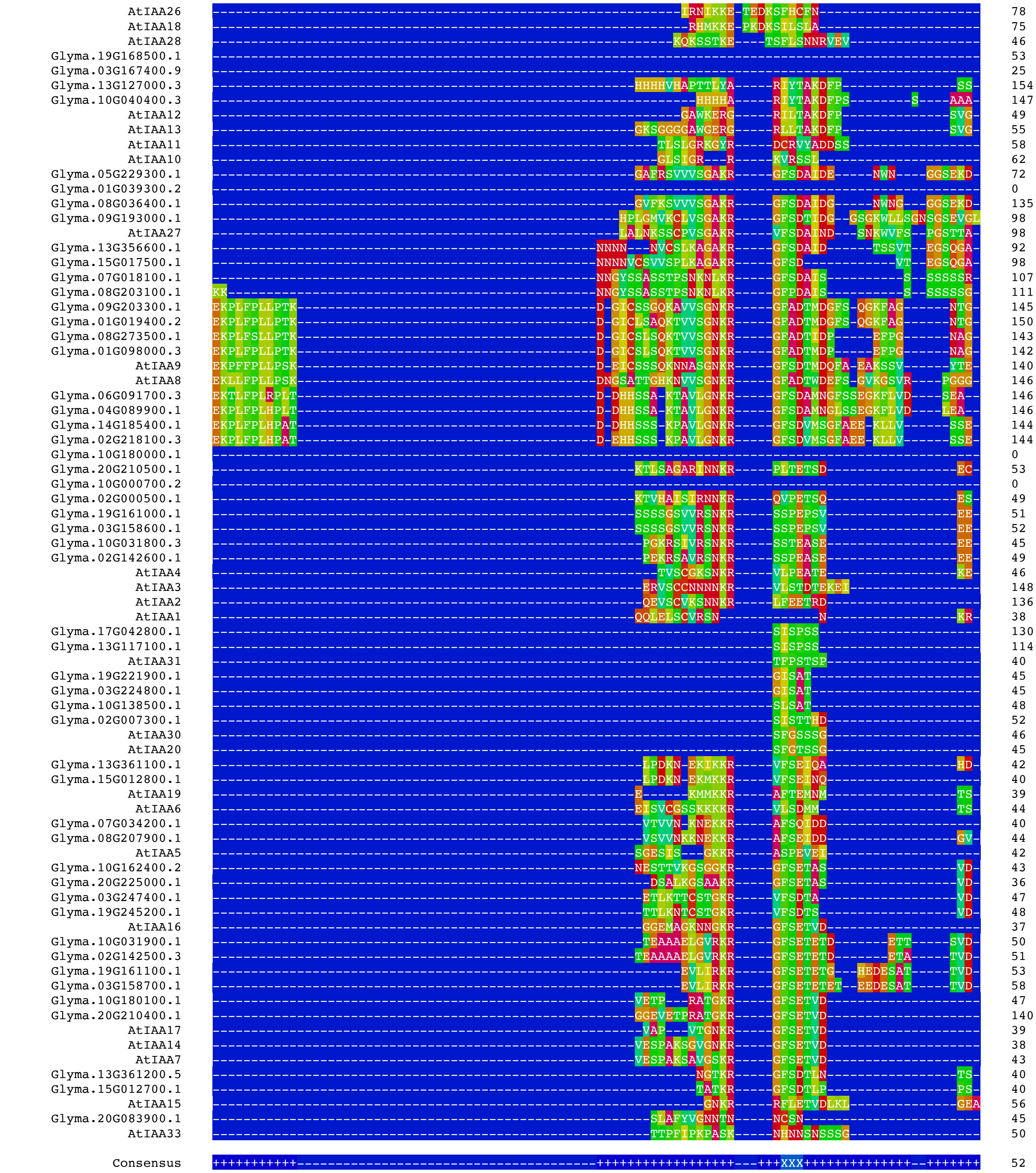
AtIAA26 78
AtIAA18 75
AtIAA28 46
Glyma.19G168500.1 53
Glyma.03G167400.9 25
Glyma.13G127000.3 154
Glyma.10G040400.3 147
AtIAA12 49
AtIAA13 55
AtIAA11 58
AtIAA10 62
Glyma.05G229300.1 72
Glyma.01G039300.2 0
Glyma.08G036400.1 135
Glyma.09G193000.1 98
AtIAA27 98
Glyma.13G356600.1 92
Glyma.15G017500.1 98
Glyma.07G018100.1 107
Glyma.08G203100.1 111
Glyma.09G203300.1 145
Glyma.01G019400.2 150
Glyma.08G273500.1 143
Glyma.01G098000.3 142
AtIAA9 140
AtIAA8 146
Glyma.06G091700.3 146
Glyma.04G089900.1 146
Glyma.14G185400.1 144
Glyma.02G218100.3 144
Glyma.10G180000.1 0
Glyma.20G210500.1 53
Glyma.10G000700.2 0
Glyma.02G000500.1 49
Glyma.19G161000.1 51
Glyma.03G158600.1 52
Glyma.10G031800.3 45
Glyma.02G142600.1 49
AtIAA4 46
AtIAA3 148
AtIAA2 136
AtIAA1 38
Glyma.17G042800.1 130
Glyma.13G117100.1 114
AtIAA31 40
Glyma.19G221900.1 45
Glyma.03G224800.1 45
Glyma.10G138500.1 48
Glyma.02G007300.1 52
AtIAA30 46
AtIAA20 45
Glyma.13G361100.1 42
Glyma.15G012800.1 40
AtIAA19 39
AtIAA6 44
Glyma.07G034200.1 40
Glyma.08G207900.1 44
AtIAA5 42
Glyma.10G162400.2 43
Glyma.20G225000.1 36
Glyma.03G247400.1 47
Glyma.19G245200.1 48
AtIAA16 37
Glyma.10G031900.1 50
Glyma.02G142500.3 51
Glyma.19G161100.1 53
Glyma.03G158700.1 58
Glyma.10G180100.1 47
Glyma.20G210400.1 140
AtIAA17 39
AtIAA14 38
AtIAA7 43
Glyma.13G361200.5 40
Glyma.15G012700.1 40
AtIAA15 56
Glyma.20G083900.1 45
AtIAA33 50
Consensus 52

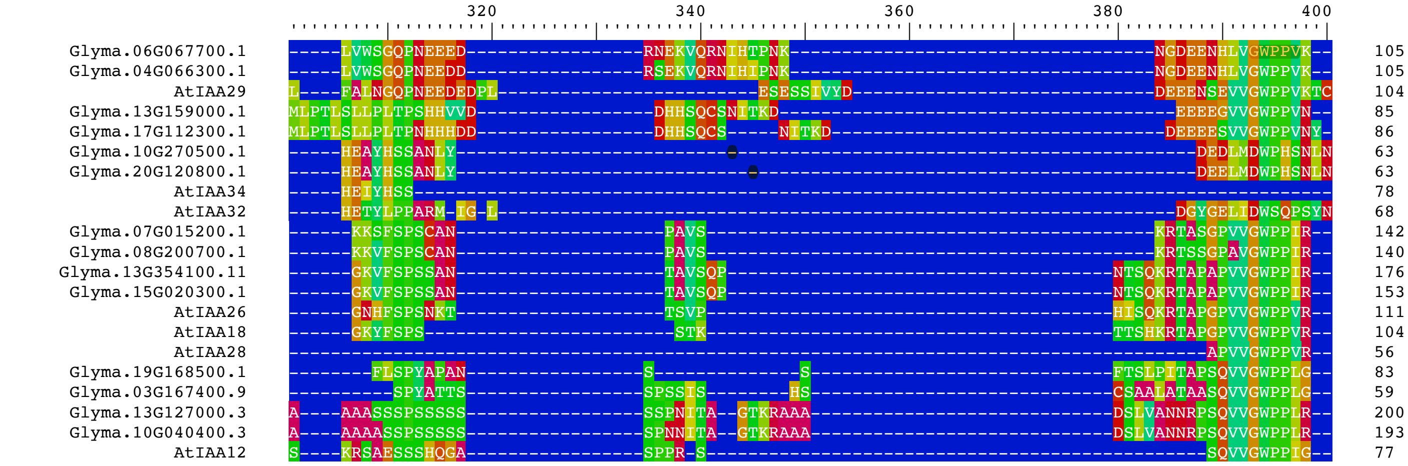
320
340
360
380
400
Glyma.06G067700.1 105
Glyma.04G066300.1 105
AtIAA29 104
Glyma.13G159000.1 85
Glyma.17G112300.1 86
Glyma.10G270500.1 63
Glyma.20G120800.1 63
AtIAA34 78
AtIAA32 68
Glyma.07G015200.1 142
Glyma.08G200700.1 140
Glyma.13G354100.11 176
Glyma.15G020300.1 153
AtIAA26 111
AtIAA18 104
AtIAA28 56
Glyma.19G168500.1 83
Glyma.03G167400.9 59
Glyma.13G127000.3 200
Glyma.10G040400.3 193
AtIAA12 77

```
            AtIAA13  S----KRAADSASHAGS-----------------SPPRSS------------------------------------------------SQVVGWPPIG--   84
            AtIAA11  ----------SSSSSSSL------------------SRASVIAGIKR--------------------------------TADSMAATSGQVVGWPPIR--   96
            AtIAA10  -----------SSSSSSL------------------TRES---GTKRSADSS---------------------------PAAASNATRQVAVGWPPLR--  101
  Glyma.05G229300.1  ------AALFSPRGAVS-----------VS--AAKSLTLTATDCTNQPTALGASVLKETVPRSPKPLHEK--------KPQISAPAAKAQVVGWPPIR--  143
  Glyma.01G039300.2  ------------------------------------------------------------------------------------------------MAS-    3
  Glyma.08G036400.1  -----AAALFSPTS-RG--------AVSVSVSAAKSLTLTATDCTNQPTALGASVLKETVPHSPKPLHEN--------KPQISAPAAKAQVVGWPPIR--  211
  Glyma.09G193000.1  GK---DGGFFSPRGVG------------VSVSAAK------AECTNQQTC----VVKDKVPQSPKPLNEK--------KPQISAPAAKEQVVGWPPIR--  163
            AtIAA27  -----TGDVGSGSGPRT-----------SVVKDGKSTTFTKPAV---------------------PVKEK--------KSSATAPASKAQVVGWPPIR--  151
  Glyma.13G356600.1  ------SALFSPRGGNV--------GKPLIGLDTQTNT------TIKEVGAV--------PQSAKPVQENNDQFAAT-NAHAIAPAAKAQVVGWPPIR--  161
  Glyma.15G017500.1  -------ALFSPRGANV--------GKPIIGLDTQTNTQQQANTTIKEVGAVL-------PQSTKPVQEKNDQVAAT-NGHASAPAAKAQVVGWPPIR--  173
  Glyma.07G018100.1  ------KWIFSQSDAA------------ATEADLENGSNNTSARCNREVDMV-------------PHYEKPAQVAAT-NDHATVPAPKAQVVGWPPIR--  173
  Glyma.08G203100.1  ------KWIFSASDAA-------------TEADLESGS-NISGGCNKEVGMV-------------PHYEKPAQVAAT-NEHAPAPAPKAQVVGWPPIR--  175
  Glyma.09G203300.1  -----MNAVLSPRPSG--------------AQPSAMKETP-SKLSERPCSTN---------------NGTGHNHT-GASISGSAPASKAQVVGWPPIR--  207
  Glyma.01G019400.2  -----MNAMLSPRPSG--------------AQPSAMKEIP-SKLQERPCSTK---------------NGTGHNHT-GASISGSAPASKAQVVGWPPIR--  212
  Glyma.08G273500.1  -----INMMLSPKPSG--------------VKPTTVKEIP-SKVLQEHPSAA---------------NGTGHNHT-GASISSSAPAAKAQVVGWPPIR--  205
  Glyma.01G098000.3  -----INMMLSPKPSG--------------VQPTTVKEIP-SKVLQELPSAA---------------NGTGHNHTSGASISGSAPAAKAQVVGWPPIR--  205
             AtIAA9  -----KNWMF---PEA------------AATQSVTKKDVPQNIPKGQSSTT---------------------------NNSSSPPAAKAQIVGWPPVR--  191
             AtIAA8  -----INMMLSPKVKD--------------VSKSIQEERSHAKG-----------------------------------GLNNAPAAKAQVVGWPPIR--  190
  Glyma.06G091700.3  -----ANPILSPRPASNLGLKPGSTLEKVGVQQTKMKEVATTKA-----------------------NEARPTIDGSANNNNSAPATKAQVVGWPPIR--  216
  Glyma.04G089900.1  -----ANPILSPRPACNLGLKPGSTLDKVGAQQTKMKEVATTKG-----------------------NETRPSIDGSANNN-SAPATKAQVVGWPPIR--  215
  Glyma.14G185400.1  -----VNTILSPRPSSNVALKPSSMLENVGAQQSKAKELATAKVGLERSHVF---------------NDSRTNLNDSANNNSSAPATKAQVVGWPPIR--  222
  Glyma.02G218100.3  -----VNTILPPRPSSNVGLKPSSMLENVGAQQ-QAKELATVKVGHERSHAV---------------NESRPNLNDSTNNNSSAPATKAQVVGWPPIR--  221
  Glyma.10G180000.1  ------------------------------------------------------------------------------------MYYRAKIVGWPPIR--   14
  Glyma.20G210500.1  ------ASNGTSSAP--------------------------------------------------------------HEKTETAPPAKTKIVGWPPIR--   83
  Glyma.10G000700.2  ----------------------------------------------------------------------------------------------------    0
  Glyma.02G000500.1  ------VSISKASPDQ-------------------------------------------------------------HVESDPAPPAKAKIVGWPPIR--   80
  Glyma.19G161000.1  -------SRCNSNGSSD-------------------------------------------------------STTTSDHDEDSVQPAKVQVVGWPPIR--   87
  Glyma.03G158600.1  -------SRCNSNGSSD-------------------------------------------------------STTTSDHDQDSAQPEKVQVVGWPPIR--   88
  Glyma.10G031800.3  -----CISKGNMN-SNG-------------------------------------------------------SDITSDDQDNLVPPAKAQVVGWPPVR--   82
  Glyma.02G142600.1  -----CISKGNMNSSDG-------------------------------------------------------SDITSDDQDNVVPPAKAQVVGWPPVR--   87
             AtIAA4  ------IESTGK-------------------------------------------------------------------TETASPP-KAQIVGWPPVR--   70
             AtIAA3  -------------ESSSR-------------------------------------------------------------KTETSPPRKAQIVGWPPVR--  172
             AtIAA2  -------------------------------------------------------------------------------EEESTPPTKTQIVGWPPVR--  155
             AtIAA1  ------KNNDST-------------------------------------------------------------------EESAPPPAKTQIVGWPPVR--   63
  Glyma.17G042800.1  ------QSESPFNSTRRE-----------------------------------------------------------------------ESFDWPPIK--  151
  Glyma.13G117100.1  ------QSELPFNSTPRE-----------------------------------------------------------------------ESFDWPPIK--  135
            AtIAA31  ------QREA--------------------------------------------------------------------------------RQDWPPIK--   52
  Glyma.19G221900.1  ------QHFASSISRGQW-----------------------------------------------------------------------QQSHHPFVN--   66
  Glyma.03G224800.1  ------QHVASSISRGQW-----------------------------------------------------------------------QQPHHPFVN--   66
  Glyma.10G138500.1  ------QHVGSSSSGGHW-----------------------------------------------------------------------QPMQPHLIS--   69
  Glyma.02G007300.1  ------QHVGSSSSGGHW-----------------------------------------------------------------------QPMQPHL----   71
            AtIAA30  ------QYY----NGGDN-----------------------------------------------------------------------HEYD--GVGA-   62
            AtIAA20  ------TQYF---NGGYG-----------------------------------------------------------------------YSVAAPAVED-   64
  Glyma.13G361100.1  -----DDENSSSEQ---------------------------------------------------------------DRKIQT----KNQVVGWPPVC--   68
  Glyma.15G012800.1  -----GDENSSSEE---------------------------------------------------------------DRKIQT----KNQVVGWPPVC--   66
            AtIAA19  -----SGSNSDQCESGVV------------------------------------------------------SSGGDAEKVNDSPAAKSQVVGWPPVC--   78
             AtIAA6  -----SALDTEN-ENSVV----------------------------------------------------------SSVEDESLPVVKSQAVGWPPVC--   78
  Glyma.07G034200.1  -------ENSSSGG---------------------------------------------------------------DRKIKTN---KSQVVGWPPVC--   65
  Glyma.08G207900.1  -----GDENSSSG--------------------------------------------------------------GGDRKMETN---KSQVVGWPPVC--   72
             AtIAA5  -------------------------------------------------------------------------------DLKCEPAKKSQVVGWPPVC--   61
  Glyma.10G162400.2  -----LKLNLSSS-DDS-----------ASDSPSSASTEK----TTTAAPPPPS-----------------------RANDPAKPPAKAQVVGWPPVR--   97
  Glyma.20G225000.1  -----LKLNLSSCINDS-----------ASDSPSSVSTEKPKENKTTTAEPPP-------------------------ANDPAKPPAKAQVVGWPPVR--   93
  Glyma.03G247400.1  -----LKLNLSSTSNS----------------ASSDLTKE----KNITAAAPP-------------------------ANDPAKPPAKAQVVGWPPVR--   95
  Glyma.19G245200.1  -----LKLNLSSTSNN--------------------------------------------------------------APPPAKPPAKAQVVGWPPVR--   79
            AtIAA16  -----LKLNLSSTAMDS-----------VSKVDLENMK-----------------------------------------EKVVKPPAKAQVVGWPPVR--   78
  Glyma.10G031900.1  -----LMLNLSPKEASA-----------AATTDGADPRENPKTSPKEKNLP---------------------------LLDPAKPPAKAQVVGWPPVR--  105
  Glyma.02G142500.3  -----LMLNLSPKE--------------AAAADGADPREKPKTSPKEKTLL---------------------------LPDPAKPPAKAQVVGWPPVR--  103
  Glyma.19G161100.1  -----LMLNLSSKEAA------------TTAAAAADPTDKHKTLPKEKTLL---------------------------PADPAKPPAKTQVVGWPPVR--  107
  Glyma.03G158700.1  -----LMLNLSSKEAA----------------AAADPTDKHKTLPKEKTLL---------------------------PADPAKPPAKAQVVGWPPVR--  108
  Glyma.10G180100.1  -----LKLNLQTKED------------------LNENLKNVS---KEKTLL----------------------------KDPAKPPAKAQVVGWPPVR--   91
  Glyma.20G210400.1  -----LKLNLHSKED---------------------LNENLKNVSKEKTLL----------------------------KDPAKPPAKAQVVGWPPVR--  184
            AtIAA17  -----LKLNLNNEPA---------------NKEGSTTHDVVTFDSKEKSAC---------------------------PKDPAKPPAKAQVVGWPPVR--   90
            AtIAA14  -----LKLNLQSNKQ------------------GHVDLNTNGA-PKEKTFL----------------------------KDPSKPPAKAQVVGWPPVR--   84
             AtIAA7  -----LMLNLQSNKE------------------GSVDLKNVSAVPKEKTTL----------------------------KDPSKPPAKAQVVGWPPVR--   90
  Glyma.13G361200.5  -----HNKMLR-------------------------------------------------------------------------PTSKEQVVGWPPVR--   60
  Glyma.15G012700.1  -----QNKILRPTS-------------------------------------------------------------------KFPTPNREQLVGWPPVR--   66
            AtIAA15  H----ENNYISSMVTND------------------------------------------------------------------------QLVGWPPVA--   78
  Glyma.20G083900.1  -----------------------------------------------------------------------------------------KFKGLLGLK--   54
            AtIAA33  -------------------------------------------------------------------------------------AAGRSFQGFGLNV--   63

          Consensus  +----+++++++++++++++-----++++++++++++++++++++++++++++-------+++++++++++++++++++++++++++++XXXXXXXXX+-   61


                                       420                 440                 460                 480                 500
                     ''''''''''|'''''''''|'''''''''|'''''''''|'''''''''|'''''''''|'''''''''|'''''''''|'''''''''|'''''''''|

  Glyma.06G067700.1  -----SWRRKELHRQQYP-----------ARGQIRNDRIQANE----NQSRRP-----------------NSLYVKVNMEGVAIGRKINLRLFNSYQTLT  168
  Glyma.04G066300.1  -----SWRRKELH-QQHP-----------ARGRIRNDRIQANE----NQSRGP-----------------NSLYVKVNMEGVAIGRKINLRLFNSYQTLT  167
            AtIAA29  MIKYGSYHHRHIRNHHHCP------------YHHRGRRITAMN----NNISNPTTATVGSSSS-SSISSRSSMYVKVKMDGVAIARKVDIKLFNSYESLT  187
  Glyma.13G159000.1  -----HWRKKLHVEEVVG---------------NNNNIDHMVWV--------DHRQTHSLQGY-----SSNTLYVKVKMEGVGIARKVDLSMHQSFHTLK  152
  Glyma.17G112300.1  -----HWRKKLRVDEVVG---------------NNNNNNHMVSV--------ADHRHHS-------------VYVKVKMEGVGIARKVDLSMHQSFHTLK  145
  Glyma.10G270500.1  LKNSSTMHSRSAHHQNFD---------------------EEIE---------------GVQSN----ER--WAYVKVNMDGVTIGRKICVLDHGGYSSLA  121
  Glyma.20G120800.1  LKNSRTMHSRSV-HQNFD---------------------EEIE---------------GVQSN----ER--WAYVKVNMDGVTIGRKICVLDHGGYSSLA  120
            AtIAA34  -----------------G---------------------QRYC---------------SNEGY----RR-KWGYVKVTMDGLVVGRKVCVLDHGSYSTLA  120
            AtIAA32  --SITQLKSEDTGHQRLA---------------------QGYY---------------NNEGE----SRGKYAYVKVNLDGLVVGRKVCLVDQGAYATLA  126
  Glyma.07G015200.1  -----SFRKNIASGSTSN-----------SKLPSGSQQQHQNVV--PGKVASQKPTD--NSG--------KGLFVKINMDGVAIGRKVDINAYDSYEKLS  214
  Glyma.08G200700.1  -----SFRKNIASGSTS-------------KLPSGSHQQHQNVV--PYKVASQKPTD--KSG--------KGLFVKINMDGVPIGRKVDINAYDSYEKLS  210
 Glyma.13G354100.11  -----SFRKNLSSSSSA------------SKPPPPPESQAEQ---QHNKVAGKKPVDNYANN--------KGLFVKINMDGVPIGRKVDLNAYDSYENLS  248
  Glyma.15G020300.1  -----SFRKNLASSSSA------------SK--PPPESQAE----QHNKVAGKKPVDNYANN--------KGLFVKINMDGVPIGRKVDLNAYDSYENLS  222
            AtIAA26  -----SFRKNLASTSSS-------------KLGNESSHGGQIN----KSDDGEKQVETKKE----------GMFVKINMDGVPIGRKVDLNAYNSYEQLS  179
            AtIAA18  -----SFRKNLASGSSS-------------KLGNDSTTSNGVTLKNQKCDAAAKTTEPKRQG---------GMFVKINMYGVPIGRKVDLSAHNSYEQLS  177
            AtIAA28  -----SSRRNLTAQLKEE--------------MKKKESDEEKE-----------------------------LYVKINMEGVPIGRKVNLSAYNNYQQLS  108
  Glyma.19G168500.1  -----AYRMNSYNSHAKSPATEVFNSTLD-KRASNSAGVRKSA----DGGSDSSNIISKEKGN-----LRTSLFVKVKMDGIPIGRKVDLGAHDSYETLA  168
  Glyma.03G167400.9  -----AYRMNIYNSQAKSPATEVFNSTVDNKRASNSTGVRKTA----DGGSDSSNIIFKEKGN-----LRSSLFVKVKMDGIPIGRKVDLGAHGSYETLA  145
  Glyma.13G127000.3  -----TYRVNSFNSHAKS--TEVFNSVAE-KSKINNTVVRKTN----DNDND-NNINAKEKRH-----LRSSLFVKVNMDGIPIGRKVDLSAHSSYETLA  282
  Glyma.10G040400.3  -----TYRVNSFNSHAKS--TEVFNSVAE-KSKTDNTVARKTN----DNGND-NNINAKEKRH-----LRSSLFVKVNMDGIPIGRKVDLSAHSSYETLA  275
            AtIAA12  -----LHRMNSLVNNQ----------AMKAARAEEGDGEKKVV----KNDEL-KDVSMKVNPK-----VQGLGFVKVNMDGVGIGRKVDMRAHSSYENLA  152
            AtIAA13  -----SHRMNSLVNNQ----------ATKSAREEEEAGKKK-V----KDDEP-KDVTKKVNGK-----VQ-VGFIKVNMDGVAIGRKVDLNAHSSYENLA  157
            AtIAA11  -----TYRMNSMVNQAKASATE----------DPNLEISQAVN----------KNRSDSTKM-------RNSMFVKVTMDGIPIGRKIDLNAHKCYESLS  164
            AtIAA10  -----TYRINSLVNQAKSLATEGGLSSGIQKETTKSVVVAAKN----------DDACFIKSS-------RTSMLVKVTMDGVIIGRKVDLNALDSYAALE  179
  Glyma.05G229300.1  -----SFRKNSMASQP-----------------QK--NDTDAE---------------AKSG---------CLYVKVSMEGAPYLRKVDLNSFTTYKDLS  195
  Glyma.01G039300.2  -----QPQKNDVAANAEAKS--------------------------------------------------GCLYVKVNMEGSPYLRKVDLNSFTTYKDLS   48
  Glyma.08G036400.1  -----SFRKNSMASQP-----------------QKNDAAADAE---------------AKSG---------CLYVKVSMEGAPYLRKVDLNSFTTYKDLS  265
  Glyma.09G193000.1  -----SFRKNSMATQP-----------------QK--NDDNAE---------------AKSV---------CLYVKVSMDGAPYLRKVDLKNFGTYMELS  215
            AtIAA27  -----SFRKNSMASSQ-------------SQKPGNNSETEEAE---------------AKSGP----EQPCLYVKVSMEGAPYLRKIDLKTYKSYLELS  213
```

```
Glyma.13G356600.1   -----SFRKNTMASNL-----------------TK--NNDEAE---------------GKSG-------FGCLYVKVSMDGAPYLRKVDLKTYNNYMELS   215
Glyma.15G017500.1   -----SFRKNTMASNL-----------------TK--NNDDDE---------------GKSG-------FGCLYVKVSMDGAPYLRKVDLKTYNNYMELS   227
Glyma.07G018100.1   -----SFRKNTMAYNL-----------------AK--CNNETE---------------EKPG-------VACLYVKVSMDGAPYLRKVDLKTYSNYIELS   227
Glyma.08G203100.1   -----SFRKNTM-MAY---------------NLAK--CDNEAE---------------EKSG-------VGCLYVKVSMDGAPYLRKVDLKTYSNYIELS   230
Glyma.09G203300.1   -----SFRKNSMATTT-----------------NK--NNDEVD---------------GKPG-------VGALFVKVSMDGAPYLRKVDLRSYTTYQELS   261
Glyma.01G019400.2   -----SFRKNSMATTT-----------------NK--NNDEVD---------------GKPG-------VGALFVKVSMDGAPYLRKVDLRSYTTYQELS   266
Glyma.08G273500.1   -----SFRKNSLATTS------------------K--NNDEVD---------------GKPG-------AAAIFVKVSMDGAPYLRKVDLTNYTTYRELS   258
Glyma.01G098000.3   -----SFRKNSLATTS------------------K--NNDEVD---------------GKPG-------AAALFVKVSMDGAPYLRKVDLRNYTMYQELS   258
           AtIAA9   -----SYRKNTLATTC------------------K--NSDEVD---------------GRPG-------SGALFVKVSMDGAPYLRKVDLRSYTNYGELS   244
           AtIAA8   -----SYRKNTMASST-----------------SK--NTDEVD---------------GKPG-------LGVLFVKVSMDGAPYLRKVDLRTYTSYQQLS   244
Glyma.06G091700.3   -----SFRKNSLATTS------------------K--NNEEVD---------------GKVG-------VGALFVKVSMDGAPYLRKVDLKNYSTYPELS   269
Glyma.04G089900.1   -----SFRKNSLATTS------------------K--NNEVVD---------------GKKG-------VGALFVKVSMDGAPYLRKVDLKNYSTYPELS   268
Glyma.14G185400.1   -----SFRKNSLATTT------------------K--NVEEVD---------------GKAG-------SGALFVKVSMDGAPYLRKVDLKNYSAYAELS   275
Glyma.02G218100.3   -----SFRKNSLVTTS------------------K--NVEEVD---------------GKVG-------PGALFVKVSMDGAPYLRKVDLKNYNAYADLS   274
Glyma.10G180000.1   -----SYRKNSLQEND---------------------------------------------G--------AGIYVKVSMDGAPYLRKIDLKVYGGYTQLL   56
Glyma.20G210500.1   -----SYRKNSLQESE---------------------------------------------G--------AGIYVKVSMDGAPYLRKIDLKVYGGYTQLL   125
Glyma.10G000700.2   ------------------------------------------------------------------------------MDGAPYLRKIDLRVY------V   16
Glyma.02G000500.1   -----SYRKQSLQEGD-----------------------Q---------------------G--------DGIYVKVIMDGAPYLRKIDLKVYRGYPELL   123
Glyma.19G161000.1   -----SFRKNSLQQQK-----------------------KVEQ---------------QGDG--------SGTYLKVSMAGAPYLRKIDLKVYNSYPELL   136
Glyma.03G158600.1   -----SFRKNSLQQQK-----------------------KVEQL--------------QGDG--------GGMYVKVSMAGAPYLRKIDLKVYNSYPELL   138
Glyma.10G031800.3   -----SYRKNTLQQK------------------------KEE----------------QGEG--------SGMYVKVSMAGAPYLRKIDLNVYKSYPELL   129
Glyma.02G142600.1   -----SYRKNSLQQK------------------------KEE----------------QAEG--------AGMYVKVSMEGAPYLRKIDLKVYKSYPELL   134
           AtIAA4   -----SYRKNNVQTKK-----------------------SE------------------SEG--------QGNYVKVSMDGAPYLRKIDLTMYKQYPELM   116
           AtIAA3   -----SYRKNNIQSKK---------------------NESEHE------------------GQ--------GIYVKVSMDGAPYLRKIDLSCYKGYSELL   220
           AtIAA2   -----SSRKNNNS----------------------------------------------------------VSYVKVSMDGAPYLRKIDLKTYKNYPELL   192
           AtIAA1   -----SNRKNNNNKN--------------------------------------------------------VSYVKVSMDGAPYLRKIDLKMYKNYPELL   102
Glyma.17G042800.1   ----SILRSTLVGKQSYLSQ-------------------------------------------------RPSLFVKVYMEGIPIGRKLNLMAHYGYDGLV   198
Glyma.13G117100.1   ----SILRSTLVGKQSHLSQ-------------------------------------------------RPSLFVKVYMEGIPIGRKLNLMAHYSYDGLV   182
          AtIAA31   ----SRLRDTLKGRRLL-----------------------------------------RRGD-------DTSLFVKVYMEGVPIGRKLDLCVFSGYESLL   100
Glyma.19G221900.1   -----IYSQ--VPAEVNDCSN----------------------------------------D-------HSSFFVKVYMEGIPIGRKLNILAHGGYYELV   112
Glyma.03G224800.1   ----NNYSQAAASAEVNDCSN----------------------------------------D-------HSSFFVKVYMEGIPIGRKLNILAHGGYYELV   115
Glyma.10G138500.1   -----SFSQ---ATEVN-----------------------------------------DCSD-------HTSFFVKVYMEGIPIGRKLNLLAHDGYHELV   113
Glyma.02G007300.1   ----SSFSQ---ATEVN-----------------------------------------HCSD-------HTSFFVKVYMEGIPIGRKLNLLAHDGYHELV   116
          AtIAA30   ----AEEMMIMEEEEQN-----------------------------------------ECNS-------VGSFYVKVNMEGVPIGRKIDLLSLNGYHDLI   110
          AtIAA20   ----AEYVAAVEEEEEN-----------------------------------------ECNS-------VGSFYVKVNMEGVPIGRKIDLMSLNGYRDLI   112
Glyma.13G361100.1   -----SYRKKNTVNET-------------------------------------------------------KMYVKVSMDGAPFLRKIDLAMHKGYSELV   108
Glyma.15G012800.1   -----SYRKKNTINET-------------------------------------------------------KMYVKVSMDGAPFLRKIDLAMHKGYSELA   106
          AtIAA19   -----SYRKKNSCKEA-----------------------ST------------------TKV--------GLGYVKVSMDGVPYLRKMDLGSSQGYDDLA   124
           AtIAA6   -----SYRRKKNNEEA-----------------------S---------------------K--------AIGYVKVSMDGVPYMRKIDLGSSNSYINLV   121
Glyma.07G034200.1   -----SYRKKNSMNEG------------------------------------------------------SKMYVKVSMDGAPFLRKIDLGLHKGYSDLA   106
Glyma.08G207900.1   -----SYRKKNSMNEG-----------------------A------------------------------SKMYVKVSMDGAPFLRKIDLGLHKGYSDLA   114
           AtIAA5   -----SYRRKNSLERT-----------------------K-------------------------------SSYVKVSVDGAAFLRKIDLEMYKCYQDLA   102
Glyma.10G162400.2   -----SFRKNIVQR---------------------NKNEEEA------------------------------AFVKVSMDGAPYLRKVDIKLYKSYQELS   141
Glyma.20G225000.1   -----SFRKNIVQR---------------------NSNEEEAE---------------KSTKN---------AFVKVSMDGAPYLRKVDIKLYKSYQELS   143
Glyma.03G247400.1   -----SFRKNIVQR------------------SNNNEGEKAAT---------------SSSNN----VNTGAAFVKVSMDGAPYLRKVDLKLYKSYQELL   153
Glyma.19G245200.1   -----SFRKNIVNNVQ---------------RSNNNDGEKAAT---------------SSSNN----VNMGAAFVKVSMDGAPYLRKVDLKMYKSHQELL   140
          AtIAA16   -----SFRKNVMSGQKPT--------TGDATEGNDKTSGSSGA---------------TSSAS----ACATVAYVKVSMDGAPYLRKIDLKLYKTYQDLS   146
Glyma.10G031900.1   -----SFRKNMFAAQK-------------------SSGGEESE---------------KNSP--------NASFVKVSMDGAPYLRKVDLKMYKSYPELS   158
Glyma.02G142500.3   -----SFRKNMFAAQK-------------------SSGGEESE---------------KSSP--------NASFVKVSMDGAPYLRKVDLKMYKSYPELS   156
Glyma.19G161100.1   -----SFRKNMLAVQK-------------------SV-GEESE---------------KN-SS------PNASFVKVSMDGAPYLRKVDLKMYKSYRELS   160
Glyma.03G158700.1   -----SFRKNMLAVQK-------------------SV-GEENE---------------KNSSS------PNASFVKVSMDGAPYLRKVDLKMYKSYRELS   162
Glyma.10G180100.1   -----SYRKNMMAVQK-----------------VSN---EEVA---------------EKTTS--STIANSGAFVKVSMDGAPYLRKVDLTMYKSYKDLS   149
Glyma.20G210400.1   -----SYRKNMMAVQK----------------VSTEDVAEKTT---------------SSTA-------NPGAFVKVSMDGAPYLRKVDLTMYKSYKELS   241
          AtIAA17   -----SYRKNVMVSCQ-----------------KSS------------------------------GGPEAAAFVKVSMDGAPYLRKIDLRMYKSYDELS   138
          AtIAA14   -----NYRKNVMANQK-----------------SGE-AEEAMS---------------SGGG--------TVAFVKVSMDGAPYLRKVDLKMYTSYKDLS   138
           AtIAA7   -----NYRKNMMTQQK-----------------TSSGAEEASS---------------EKAGNF-GGGAAGAGLVKVSMDGAPYLRKVDLKMYKSYQDLS   152
Glyma.13G361200.5   -----ASRKNAMKMS--------------------------------------------------------CKLVKVAVDGAPYLRKVDLEMYETYEHLM   99
Glyma.15G012700.1   -----ASRKNAMKSC--------------------------------------------------------CKLVKVAVDGAPYLRKVDLDMYDSYEHLM   105
          AtIAA15   -------TARKTVRRK---------------------------------------------------------YVKVALDGAAYLRKVDLGMYDCYGQLF   114
Glyma.20G083900.1   --------DDDLGSTV-------------------------------------------------------VLVVTIMLEGCSIYQRISLHNHDS-----   86
          AtIAA33   --------EDDLVSSV-------------------------------------------------------VPPVTVVLEGRSICQRISLDKHGSYQSLA   100

        Consensus   ----+XXXXXXXXX++++++++++++++++++++++++++++++--+++++++++++++++++----+++++XXVKXXXXGXXXXRKXXXXXXXXXXXLX   98
```

```
                              520                 540                 560                 580                 600
                    .........|.........|.........|.........|.........|.........|.........|.........|.........|.........|

Glyma.06G067700.1   SSLISMFA------------------------------------KYQKFEEVGESYTLNFQNEQGDWLQVGHVPWQSFIGTVRRLVILRNGSE--T----   226
Glyma.04G066300.1   SSLISMFA------------------------------------KYQKFEEVGESYTLTFQNEQGEWLQVGHVPWQSFIGTVRRLVILRNGSE--T----   225
          AtIAA29   NSLITMFTE------------------------------------YEDCDREDTNYTFTFQGKEGDWLLRGDVTWKIFAESVHRISIIRDRPC-------   244
Glyma.13G159000.1   ETLMDMFGK--------------------------------------CHHQQSNNYELAYLDKEGDWLLAQDVPWRSFVGCARRLKLVKTS---------   205
Glyma.17G112300.1   QTLMDMFGK--------------------------------------CNIQQSNNYELAYLDKEGDWLLAQDLPWRSFVGCARRLKLVKSS---------   198
Glyma.10G270500.1   LQLEDMFGS--------------------------------HSVSGLRLFQSGSEYSLFYKDRQDNWRPVGDVPWKEFIECVKRLRIARKNSG--IVSCS   187
Glyma.20G120800.1   LQLEDMFGS--------------------------------QSVSGLRLFQSGSEYSLFYKDRQDNWRPVGDVPWKEFIECVKRLRIARKNSG--IVSYS   186
          AtIAA34   HQLEDMFGM--------------------------------QSVSGLRLFQMESEFCLVYRDEEGLWRNAGDVPWNEFIESVERLRITRRNDA--VLPF-   185
          AtIAA32   LQLNDMFGM--------------------------------QTVSGLRLFQTESEFSLVYRDREGIWRNVGDVPWKEFVESVDRMRIARRNDA--LLPF-   191
Glyma.07G015200.1   SAVDELFRGLLAEMKLSHIASSQCCSGQRDSCAGGIQNKEQEEKSNTGLLVGSGEYTLVYEDNEGDRMLVGDVPWHMFVSTVKRLRVLKSSDL-PAFTLG   313
Glyma.08G200700.1   SAVDELFRGLLAEMKLSHIGSSQCCSGQRDSCAGGIQNKEQEEKSNKGLLVGSGEYTLVYEDNEGDRMLVGDVPWHMFVSTVKRLRVLKSSDL-PAFTLG   309
Glyma.13G354100.11  SAVDELFRGLLAAQRDS--------------SAGGVHNKQEEEKAITGLLDGSGEFTLVYEDNEGDRMLVGDVPWHMFVSTVKRLRVLKSSEL-SAFTLG   333
Glyma.15G020300.1   SAVDELFRGLLAAQRDS--------------SAGGVHNKQEEEKAITGLLDGSGEYTLVYEDNEGDRMLVGDVPWHMFVSTVKRLRVLKSSEL-SAFTLG   307
          AtIAA26   FVVDKLFRGLLAAQRD-------------------ISDGQGEEKPIIGLLDGKGEFTLTYEDNEGDKMLVGDVPWQMFVSSVKRLRVIKSSEISSALTFG   260
          AtIAA18   FTVDKLFRGLLAAQRD-------------------FPSSIEDEKPITGLLDGNGEYTLTYEDNEGDKMLVGDVPWQMFVSSVKRLRVIKTSEISSALTYG   258
          AtIAA28   HAVDQLFSK-------------------------------------KDSWDLNRQYTLVYEDTEGDKVLVGDVPWEMFVSTVKRLHVLKTSHA---FSLS   168
Glyma.19G168500.1   QTLEDMFDE-----------STTVLTHKV-GSNGEDHGTEVGTDGHSKLLDGSSDFVLTYEDKEGDWVLVGDVPWWMFLNSVRRLRIMRTPED---NGLA   253
Glyma.03G167400.9   QTLEDMFDE-----------SATVLTHK--GSNGEDHGTEVGADGHSKLLHGSSDLVLTYEDKEGDWMLVGDVPWWMFLNSVRRLRIMRTPEA---NGLA   229
Glyma.13G127000.3   QTLEDMFNE-----------STTVTTCK--GSNGEDYGIIIGGERHSKLLDGSSKFVLTYEDKEGDWMLVGDVPWGMFLSSVRRLRIMRTSEA---NGLA   366
Glyma.10G040400.3   QTLEDMFNE-----------STTVTTCK--GSNGEDYGFIIGGERHSKLLDGSSKFVLTYEDKEGDWMLVGDVPWGMFFSSVRRLRIMRTSEA---NGLA   359
          AtIAA12   QTLEEMFF------------GMTGTTCR-------------EKVKPLRLLDGSSDFVLTYEDKEGDWMLVGDVPWRMFINSVKRLRIMGTSEA---SGLA   224
          AtIAA13   QTLEDMFFR--------TNPGTVGLT-S-------------QFTKPLRLLDGSSEFVLTYEDKEGDWMLVGDVPWRMFINSVKRLRVMKTSEA---NGLA   232
          AtIAA11   NTLEEMFLK----------PKLGSRTLE---------TDGHMETPVKILPDGSSGLVLTYEDKEGDWMLVGDVPWGMFIGSVRRLRIMKTSEA---TGKD   242
          AtIAA10   KTLDLMFFQI-------PSPVTRSNTQG---------YKTIKETCTSKLLDGSSEYIITYQDKDGDWMLVGDVPWQMFLGSVTRLRIMKTSIG---AGVG   260
Glyma.05G229300.1   LALEKMFSC---------------FTLSQCGSYGVSSRENLSESRLMDLLHG-SEYVLTYEDKDGDWMLVGDVPWEMFTESCKRLRIMKSFEA---IGLA   276
Glyma.01G039300.2   LALEKMFSC---------------FTLSQCGSYGVSSRENLSESRLMDLLHG-SKYVLIYEDKDGDWMLVGDVLWEGKVH*-------------------   113
Glyma.08G036400.1   LALEKMFSC---------------FTLSQCGSYGVSSRENLSESRLMDLLHG-SEYVLTYEDKDGDWMLVGDVPWEMFTESCKRLRIMKSSEA---IGLA   346
Glyma.09G193000.1   SALEKMFSC---------------FTISQCGSHGVCGRDKLTENRLMDLLHG-SEYVLTYEDKDGDWMLVGDVPWEMFTDSCKRLRIMKSSEA---IGLA   296
          AtIAA27   SALEKMFSC---------------FTIGQFGSHGGCGRDGLNESRLTDLLRG-SEYVVTYEDKDSDWMLVGDVPWEMFICSCKKLRIMKSSEA---IGLA   294
Glyma.13G356600.1   SALEKMFSC---------------FTIGQCNSPGLPGKDGLSESSLRDLLHG-SEYVLTYEDKDGDWMLVGDVPWEMFTDSCRRLRIMKGSEA---IGLA   296
Glyma.15G017500.1   SALEKMFSC---------------FTIGQCNSPGLPGKDGLSESSLRDLLHG-SEYVLTYEDKDGDWMLVGDVPWEMFTDSCRRLRIMKGSEA---IGLA   308
Glyma.07G018100.1   SGLEKMFSC---------------FTIGQCNSRALPGKDGLSESAFRDIVDG-SEYVLTYVDKEGDWMLVGDVPWEMFTESCNKLRIMKGSEA---IGLA   308
Glyma.08G203100.1   SALEKMFSC---------------FTIGQCNSRALPGKDGLSESAFRDLVDG-SEYVLTYEDKEGDWMLVGDVPWKMFTESCKKLRIMKGSEA---IGLA   311
Glyma.09G203300.1   SALEKMFLS--------------CFTLGQCGSHGAPGREMLSESKLRDLLHG-SEYVLTYEDKDGDWMLVGDVPWEMFIETCKRLKIMKGSDA---IGLA   343
Glyma.01G019400.2   SALEKMFLS--------------CFTLGQCGSHGAPGREMLSESKLRDLLHG-SEYVLTYEDKDGDWMLVGDVPWEMFIDTCKRLKIMKGSDA---IGLA   348
Glyma.08G273500.1   SALEKMFSC---------------FTLGQCGSHGAPGREMLSESKLKDLLHG-SEYVLTYEDKDGDWMLVGDVPWDMFIDTCKRLKIMKGSDA---IGLA   339
Glyma.01G098000.3   SALEKMFSC---------------FTLGQCGSHGAPGREMLSESKLKDLLHG-SEYVLTYEDKDGDWMLVGDVPWDMFIDTCKRLKIMKGSDA---IGLA   339
```

```
        AtIAA9  SALEKMFTT---------------FTLGQCGSNGAAGKDMLSETKLKDLLNG-KDYVLTYEDKDGDWMLVGDVPWEMFIDVCKKLKIMKGCDA---IGLA  325
        AtIAA8  SALEKMFSC---------------FTLGQCGLHGAQGRERMSEIKLKDLLHG-SEFVLTYEDKDGDWMLVGDVPWEIFTETCQKLKIMKGSDS---IGLA  325
Glyma.06G091700.3  SALEKMFSC---------------FTMSKCGSHGILGREMLNETKLKDLLHG-SEYVLTYEDREGDWMLVGDVPWEMFIETCKRLRIMKSSDA---IGLA  350
Glyma.04G089900.1  SALEKMFSC---------------FTISKCGSHGILGREMLNETKLKDLLHG-SEYVLTYEDKDGDWMLVGDVPWEMFIETCKRLRIMKSSDA---IGLA  349
Glyma.14G185400.1  SALENMFSC---------------FTIGSCGSHGNLGGEVLNETKLKDLLHG-SEYVLTYKDKDGDWMLVGDVPWEMFIETCKRLRIMKSSEA---IGLA  356
Glyma.02G218100.3  SALENMFSC---------------FTIGSCGSHGNLGGEVLNETKLKDLLHG-SEYVLTYEDKDGDWMLVGDVPWEMFTETCKRLRIMKSSEA---IGLA  355
Glyma.10G180000.1  KALENMFKL----------------TIG--------------EYSEKEGYKG-SDYAPTYEDKDGDWMLVGDVPWDMFVTSCKRLRIMKGSEA---RGLG  122
Glyma.20G210500.1  KSLENMFKL----------------TIG--------------EHSEKEGYKG-SDYAPTYEDKDGDWMLVGDVPWDMFVTSCRRLRIMKGSEA---RGLG  191
Glyma.10G000700.2  QALETMFKL--------------TIVVSS------------GEYSKREGYKG-SEYAPTYEDKDGDWMLVGDVPLDMFMTSCKRLRVM------------  77
Glyma.02G000500.1  KALETMFKL----------------TIG--------------EYSEREGYKG-SEYAPTYEDKDGDWMLVGDVPWDMFMTSCKRLRVMKGSEA---RGLG  189
Glyma.19G161000.1  MALQNLFKC----------------TFG--------------EYSEREGYNG-SEYAPTYEDKDGDWMLVGDVPWNMFVSSCKRLKIIKGSEA---KGLG  202
Glyma.03G158600.1  AALQSLFTC----------------TFG--------------EYSEREGYNG-SEYAPTYEDKDGDWMLVGDVPWNMFVSSCKRLKIIKGSEA---KGLG  204
Glyma.10G031800.3  KALGNMFKC----------------TFG--------------EYSEREGYNG-SEYAPTYEDKDGDWMLVGDVPWNMFVSSCKRLRIMKASEA---KGLG  195
Glyma.02G142600.1  KALENMFKC----------------TFG--------------QYSEREGYNG-SEYAPTYEDKDGDWMLVGDVPWNMFVSSCKRLRIMKGSEA---KGLG  200
        AtIAA4  KSLENMFKF----------------SVG--------------EYFEREGYKG-SDFVPTYEDKDGDWMLVGDVPWEMFVSSCKRLRIMKGSEV---KGLG  182
        AtIAA3  KALEVMFKF------------------------------SVGEYFERDGYKG-SDFVPTYEDKDGDWMLIGDVPWEMFICTCKRLRIMKGSEA---KGLG  286
        AtIAA2  KALENMFKV------------------------------MIGEYCEREGYKG-SGFVPTYEDKDGDWMLVGDVPWDMFSSSCKRLRIMKGSDA---PALD  258
        AtIAA1  KALENMFKF----------------TVG--------------EYSEREGYKG-SGFVPTYEDKDGDWMLVGDVPWDMFSSSCQKLRIMKGSEA---PT--  166
Glyma.17G042800.1  KTLGHMFRT--------------NILC-----------------PNSQPLNSGNFHVLTYEDQEGDWMMVGDVPWEMFLNSVKRLKITRADRC-------  260
Glyma.13G117100.1  KTLGHMFRT--------------NILC-----------------PNSQPLNSRNFHVLTYEDQEGDWMMVGDVPWEMFLNSVKRLKITRADRC-------  244
       AtIAA31  ENLSHMFDT--------------SIICG---------------------NRDRKHHVLTYEDKDGDWMMVGDIPWDMFLETVRRLKITRPERY-------  158
Glyma.19G221900.1  RTLEHMFDT--------------TILWG----------------TEMNGVQPERCHVLTYEDEEGDLVMVGDVPWEMFLSTVKRLKITRVDTF-------  175
Glyma.03G224800.1  RTLEHMFDT--------------TILWG----------------TEMNGVQPERCHVLTYEDEEGDLVMVGDVPWEMFLSTVKRLKITRVDTF-------  178
Glyma.10G138500.1  KTLEQMFDT--------------TILWG----------------TEMDGVQPERCHVLTYEDGEGDLIMVGDVPWEMFLSAVKRLKITRVEAF-------  176
Glyma.02G007300.1  KTLEQMFDT--------------TILWG----------------TEMDGVQPDRCHVLTYEDGEGDLIMVGDVPWEMFLSAVKRLKITRVETF-------  179
       AtIAA30  TTLDYMFNA--------------SILW-----------------AEEEDMCSEKSHVLTYADKEGDWMMVGDVPWEMFLSSVRRLKISRAYHY-------  172
       AtIAA20  RTLDFMFNA--------------SILW-----------------AEEEDMCNEKSHVLTYADKEGDWMMVGDVPWEMFLSTVRRLKISRAN-Y-------  173
Glyma.13G361100.1  LALEKFFGC----------------Y---------------GIREALKDAEN-AEHVPIYEDKDGDWMLVGDVPWEMFIESCKRLRIMKRSDA---KGFD  173
Glyma.15G012800.1  LALEKFFGC----------------Y---------------GIGSALKDEEN-VEQVPIYEDKDGDWMLVGDVPWEMFIESCKRLRIMKRSDA---KGFD  171
       AtIAA19  FALDKLFGF----------------R---------------GIGVALKDGDN-CEYVTIYEDKDGDWMLAGDVPWGMFLESCKRLRIMKRSDA---TGFG  189
        AtIAA6  TVLENLFGC----------------L---------------GIGVA-KEGKK-CEYIIIYEDKDRDWMLVGDVPWQMFKESCKRLRIVKRSDA---TGFG  185
Glyma.07G034200.1  LALDKLFGS----------------Y---------------GMVEALKNADN-SEHVPIYEDKDGDWMLVGDVPWEMFMESCKRLRIMKRSDA---KGFG  171
Glyma.08G207900.1  LALDKLFGC----------------Y---------------GMVEALKNADN-SEHVPIYEDKDGDWMLVGDVPWEMFMESCKRLRIMKKSDA---KGFG  179
        AtIAA5  SALQILFGC----------------Y----------------INFD-DTLKE-SECVPIYEDKDGDWMLAGDVPWEMFLGSCKRLRIMKRSCN---RG--  163
Glyma.10G162400.2  DALAKMFSS---------------FTIEKCGSQGM--KDFMNETKLIDLLNG-SDYVPTYQDKDGDWMLVGDVPWEMFVESCQRLRIMKGSEA---IGLA  220
Glyma.20G225000.1  DALAKMFSS---------------FTIEKCGSQGM--KDFMNET------NG-SDYVPTYEDKDGDWMLVGDVPWEMFVESCKRLRIMKGSEA---IGLA  216
Glyma.03G247400.1  DALAKMFSS---------------FTIDKCGSQGM--KDFMNESKLIDLLNG-SDYVPTYEDKDADWMLVGDVPWEMFVESCKRLRIMKGSEA---IGLA  232
Glyma.19G245200.1  DALAKMFSS---------------FTIDKCSSQGM--KDFMNEGKLIDLLNG-SDYVPTCEDKDGDWMLVGDVPWEILVESCKRLRIMKGSAA---IGLA  219
       AtIAA16  NALSKMFSS---------------FTIGNYGPQGM--KDFMNESKLIDLLNG-SDYVPTYEDKDGDWMLVGDVPWEMFVDSCKRIRIMKGSEA---IGLA  225
Glyma.10G031900.1  DALGKMFSS---------------FTIGNCESQGF--KDFMNESKLMDLLNS-SDYVPTYEDRDGDWMLVGDVPWEMFVESCKRLRIMKGKEA---IGLA  237
Glyma.02G142500.3  DALGKMFSS---------------FTIGNCESQGF--KDFMNESKLMDLLNS-SDYVPTYEDRDGDWMLVGDVPWEMFVESCKRLRIMKGKEA---IGLA  235
Glyma.19G161100.1  DSLGKMFSS---------------FTFGNCESQGM--KDFMNESKLNDLLNS-SDYVPTYEDKDGDWMLVGDVPWEMFVESCKRLRIMKGKEA---IGLG  239
Glyma.03G158700.1  DSLGKMFSS---------------FTIGNCESQGM--KDFMNESKLNDLLNS-SDYVPTYEDKDGDWMLVGDVPWEMFVESCKRLRIMKGKEA---IGLG  241
Glyma.10G180100.1  DALAKMFSS---------------FTMGNYGAQGM--IDFMNESKLMDLLNS-SEYVPTYEDKDGDWMLVGDVPWEMFVGSCKRLRIMKGSEA---IGLA  228
Glyma.20G210400.1  DALAKMFSS---------------FTMGNYGAQGM--IDFMNESKLMDLLNS-SEYVPSYEDKDGDWMLVGDVPWEMFVESCKRLRIMKGSEA---IGLA  320
       AtIAA17  NALSNMFSS---------------FTMGKHGG-EEGMIDFMNERKLMDLVNS-WDYVPSYEDKDGDWMLVGDVPWPMFVDTCKRLRLMKGSDA---IGLA  218
       AtIAA14  DALAKMFSS---------------FTMGSYGAQGM--IDFMNESKVMDLLNS-SEYVPSYEDKDGDWMLVGDVPWPMFVESCKRLRIMKGSEA---IGLG  217
        AtIAA7  DALAKMFSS---------------FTMGNYGAQGM--IDFMNESKLMNLLNS-SEYVPSYEDKDGDWMLVGDVPWEMFVESCKRLRIMKGSEA---VGLA  231
Glyma.13G361200.5  RELETMFCG---------------LAIRN---------HLMNERKLMESGNG-IEYMPTYEDKDGDWMLVGDVPWKMFVESCKRIRLMISSEA---VGLG  171
Glyma.15G012700.1  RELETMFCG---------------LAIRN---------HLMNERKLMDPGNG-IEYMPTYEDKDGDWMLVGDVPWKMFVESCKRIRLMISSEA---VGLG  177
       AtIAA15  TALENMFQG--------------IITICK*----------------------------------------------------------------------  130
Glyma.20G083900.1  -----------------------------------------TSDDDLDHSNAILGHLIAYEDMENNLLLAGD--------LIKRIDILPIRGN-------  130
       AtIAA33  SALRQMFVD------------------------------GADSTDDLDLSNAIPGHLIAYEDMENDLLLAGDLTWKDFVRVAKRIRILPVKGN-------  163

     Consensus  XXXXXXFXX+++++++---++++++++++++++++++++++++++XXXXX+XXXXXXYXDXXXXXXXXGDVPWXXFXXXXXXXXXXXXXXX-++++++  152

                                620                 640                 660                 680                 700

Glyma.06G067700.1  ----------------------------------------------------------------------------I*----------------------  228
Glyma.04G066300.1  ----------------------------------------------------------------------------I*----------------------  227
       AtIAA29  ----------A------------YTR---------------CLF---------------------------------*----------------------  252
Glyma.13G159000.1  -R---------------------------------------------------------------------------*----------------------  207
Glyma.17G112300.1  -R---------------------------------------------------------------------------*----------------------  200
Glyma.10G270500.1  SR---------------------------------------Y----------------------------------T*----------------------  192
Glyma.20G120800.1  SR---------------------------------------C----------------------------------T*----------------------  191
       AtIAA34  -----------------------------------------------------------------------------*----------------------  186
       AtIAA32  -----------------------------------------------------------------------------*----------------------  192
Glyma.07G015200.1  SK-------QD------------------------------------------------------------------*----------------------  318
Glyma.08G200700.1  SK-------QD------------------------------------------------------------------*----------------------  314
Glyma.13G354100.11  SK-------QDK---------IPLDS------------------------------------------------AMK*----------------------  347
Glyma.15G020300.1  SK-------QDK---------IPLDS------------------------------------------------AMK*----------------------  321
       AtIAA26  CS-------KQ-------------EK------------------------------------------------MMH*----------------------  270
       AtIAA18  NG-------KQ-------------EK------------------------------------------------MRR*----------------------  268
       AtIAA28  PR-----------------------K-----------------------------------------------HGKE*----------------------  176
Glyma.19G168500.1  PR--------L------------EEKNRRSNTSSYR*---------------------------------------------------------------  270
Glyma.03G167400.9  LIQLLICSPKI------------EGKEQKIEMQAHIDNVIVCT----------FTCARIHK---TYELKCTTRIEGI*----------------------  282
Glyma.13G127000.3  PR--------L------------EEN-------------------------------------------IKQRCKPI*----------------------  381
Glyma.10G040400.3  PR--------L------------EEN-------------------------------------------IKKRCKPI*----------------------  374
       AtIAA12  PR--------R------------QEQ------------------------------------------KDRQRNNPV*----------------------  240
       AtIAA13  AR--------N------------QEP------------------------------------------NERQRKQPV*----------------------  248
       AtIAA11  DI---------------------MKQ----------------------------------------------------IIIYEEPFMFEAVIRQITDQRE  269
       AtIAA10  K*--------------------------------------------------------------------------------------------------  262
Glyma.05G229300.1  PR--------A------------MEK---------------C-------------------------------KSRN*----------------------  288
Glyma.01G039300.2  ----------------------------------------------------------------------------------------------------  113
Glyma.08G036400.1  PR--------A------------MEK---------------C-------------------------------KSRY*----------------------  358
Glyma.09G193000.1  PR--------A------------MEK---------------C-------------------------------KSRN*----------------------  308
       AtIAA27  PR--------V------------MEK---------------C-------------------------------RSRN*----------------------  306
Glyma.13G356600.1  PR--------A------------MEK---------------S-------------------------------RSQN*----------------------  308
Glyma.15G017500.1  PR--------A------------MEK---------------S-------------------------------RSQN*----------------------  320
Glyma.07G018100.1  PR--------G------------MEK---------------C-------------------------------RSQY*----------------------  320
Glyma.08G203100.1  PR--------G------------MEK---------------F-------------------------------RSQY*----------------------  323
Glyma.09G203300.1  PR--------A------------MEK---------------S-------------------------------KSRI*----------------------  355
Glyma.01G019400.2  PR--------A------------MEK---------------S-------------------------------KSRS*----------------------  360
Glyma.08G273500.1  PR--------A------------MEK---------------S-------------------------------RSRC*----------------------  351
Glyma.01G098000.3  PR--------A------------MEK---------------S-------------------------------RSRC*----------------------  351
        AtIAA9  AAP------RA------------MEK---------------S-------------------------------KMRA*----------------------  339
        AtIAA8  PG--------A------------VEK---------------SK-----------------------------NKERV*----------------------  339
Glyma.06G091700.3  PR--------A------------VEK---------------C-------------------------------KSRT*----------------------  362
Glyma.04G089900.1  PR--------A------------VEK---------------S-------------------------------KSRT*----------------------  361
Glyma.14G185400.1  PR--------A------------VEK---------------S-------------------------------KRRN*----------------------  368
Glyma.02G218100.3  PR--------A------------VEK---------------S-------------------------------KSRN*----------------------  367
Glyma.10G180000.1  C--------------------------------------------------------------------------AV*----------------------  126
Glyma.20G210500.1  C--------------------------------------------------------------------------AV*----------------------  195
```

```
Glyma.10G000700.2  -----------------------------------------------------------------------------*----------------------  78
Glyma.02G000500.1  C--------------------------------------------------------------------------GV*----------------------  193
Glyma.19G161000.1  C---------------------------------------------------------------------------L*----------------------  205
Glyma.03G158600.1  C---------------------------------------------------------------------------L*----------------------  207
Glyma.10G031800.3  C---------------------------------------------------------------------------F*----------------------  198
Glyma.02G142600.1  C---------------------------------------------------------------------------F*----------------------  203
           AtIAA4  C-------------------------------------------------------------------------GGL*----------------------  187
           AtIAA3  CG--------------------------------------------------------------------------V*----------------------  290
           AtIAA2  SS--------------------------------------------------------------------------L*----------------------  262
           AtIAA1  ---------------------------------------------------------------------------AL*----------------------  169
Glyma.17G042800.1  -----------------------------------------------------------------------------*----------------------  261
Glyma.13G117100.1  -----------------------------------------------------------------------------*----------------------  245
          AtIAA31  -----------------------------------------------------------------------------*----------------------  159
Glyma.19G221900.1  ----------GC-----------------------------------------------------------------*----------------------  178
Glyma.03G224800.1  ----------GC-----------------------------------------------------------------*----------------------  181
Glyma.10G138500.1  ----------G------------------------------------------------------------------*----------------------  178
Glyma.02G007300.1  ----------G------------------------------------------------------------------*----------------------  181
          AtIAA30  -----------------------------------------------------------------------------*----------------------  173
          AtIAA20  ----------HY-----------------------------------------------------------------*----------------------  176
Glyma.13G361100.1  LQ------PKGS-------LKGFIEG------------------------------------------------VTK*----------------------  190
Glyma.15G012800.1  LQ------PKGS-------LKGFIEG------------------------------------------------VTK*----------------------  188
          AtIAA19  LQ------PRG---------------------------------------------------------------VDE*----------------------  198
           AtIAA6  LQ-------------------------------------------------------------------------QD*----------------------  190
Glyma.07G034200.1  LQ------PKGS-------LKGFIES------------------------------------------------AAK*----------------------  188
Glyma.08G207900.1  LQ------PKGS-------LKGFIES------------------------------------------------AAK*----------------------  196
           AtIAA5  -----------------------------------------------------------------------------*----------------------  164
Glyma.10G162400.2  PR--------A------------VEK---------------C-------------------------------KNRS*----------------------  232
Glyma.20G225000.1  PR--------A------------VEK---------------C-------------------------------KNRS*----------------------  228
Glyma.03G247400.1  PR--------A------------VEK---------------C-------------------------------KNRS*----------------------  244
Glyma.19G245200.1  PR--------A------------VQK---------------C-------------------------------KNRS*----------------------  231
          AtIAA16  PR--------A------------LEK---------------C-------------------------------KNRS*----------------------  237
Glyma.10G031900.1  PR--------A------------VEK---------------C-------------------------------KNRS*----------------------  249
Glyma.02G142500.3  PR--------A------------VEK---------------C-------------------------------KNRS*----------------------  247
Glyma.19G161100.1  LA--------PR----------AMAK---------------C-------------------------------KNRS*----------------------  253
Glyma.03G158700.1  LA--------PR----------AMAK---------------S-------------------------------KNRS*----------------------  255
Glyma.10G180100.1  PR--------A------------MEK---------------C-------------------------------KSRS*----------------------  240
Glyma.20G210400.1  PR--------A------------MEK---------------C-------------------------------KSRS*----------------------  332
          AtIAA17  PR--------A------------MEK---------------C-------------------------------KSRA*----------------------  230
          AtIAA14  KF--------S-------------------------------------------------------------------FLFV------------------  224
           AtIAA7  PR--------A------------MEKY--------------C-------------------------------KNRS*----------------------  244
Glyma.13G361200.5  S-----------------FISFFIFSL--------------CICDKIIRYDFDFDSSDLLILQVRGLLLQNVRVPPS*----------------------  218
Glyma.15G012700.1  PR------STSSKCTGST*---------------------------------------------------------------------------------  190
          AtIAA15  ----------------------------------------------------------------------------------------------------  130
Glyma.20G083900.1  SK-------KNS-----------QEY-------------------------------------------------EP*----------------------  141
          AtIAA33  TR-------Q-------------VKR-------------------------------------------------NE*----------------------  172

        Consensus  ++------++++-------+++++++---------------+---------------------------++++++++*XJXX++++++++++++++++++  157
```

```
                                  720
Glyma.06G067700.1  ----------------------------------  228
Glyma.04G066300.1  ----------------------------------  227
          AtIAA29  ----------------------------------  252
Glyma.13G159000.1  ----------------------------------  207
Glyma.17G112300.1  ----------------------------------  200
Glyma.10G270500.1  ----------------------------------  192
Glyma.20G120800.1  ----------------------------------  191
          AtIAA34  ----------------------------------  186
          AtIAA32  ----------------------------------  192
Glyma.07G015200.1  ----------------------------------  318
Glyma.08G200700.1  ----------------------------------  314
Glyma.13G354100.11 ----------------------------------  347
Glyma.15G020300.1  ----------------------------------  321
          AtIAA26  ----------------------------------  270
          AtIAA18  ----------------------------------  268
          AtIAA28  ----------------------------------  176
Glyma.19G168500.1  ----------------------------------  270
Glyma.03G167400.9  ----------------------------------  282
Glyma.13G127000.3  ----------------------------------  381
Glyma.10G040400.3  ----------------------------------  374
          AtIAA12  ----------------------------------  240
          AtIAA13  ----------------------------------  248
          AtIAA11  DKNIVRSFFFFSPLYSFFFGSAIFLLVSYMFSL*  303
          AtIAA10  ----------------------------------  262
Glyma.05G229300.1  ----------------------------------  288
Glyma.01G039300.2  ----------------------------------  113
Glyma.08G036400.1  ----------------------------------  358
Glyma.09G193000.1  ----------------------------------  308
          AtIAA27  ----------------------------------  306
Glyma.13G356600.1  ----------------------------------  308
Glyma.15G017500.1  ----------------------------------  320
Glyma.07G018100.1  ----------------------------------  320
Glyma.08G203100.1  ----------------------------------  323
Glyma.09G203300.1  ----------------------------------  355
Glyma.01G019400.2  ----------------------------------  360
Glyma.08G273500.1  ----------------------------------  351
Glyma.01G098000.3  ----------------------------------  351
           AtIAA9  ----------------------------------  339
           AtIAA8  ----------------------------------  339
Glyma.06G091700.3  ----------------------------------  362
Glyma.04G089900.1  ----------------------------------  361
Glyma.14G185400.1  ----------------------------------  368
Glyma.02G218100.3  ----------------------------------  367
Glyma.10G180000.1  ----------------------------------  126
Glyma.20G210500.1  ----------------------------------  195
Glyma.10G000700.2  ----------------------------------  78
Glyma.02G000500.1  ----------------------------------  193
Glyma.19G161000.1  ----------------------------------  205
Glyma.03G158600.1  ----------------------------------  207
Glyma.10G031800.3  ----------------------------------  198
Glyma.02G142600.1  ----------------------------------  203
           AtIAA4  ----------------------------------  187
           AtIAA3  ----------------------------------  290
```

```
          AtIAA2    ----------------------------------    262
          AtIAA1    ----------------------------------    169
Glyma.17G042800.1   ----------------------------------    261
Glyma.13G117100.1   ----------------------------------    245
         AtIAA31    ----------------------------------    159
Glyma.19G221900.1   ----------------------------------    178
Glyma.03G224800.1   ----------------------------------    181
Glyma.10G138500.1   ----------------------------------    178
Glyma.02G007300.1   ----------------------------------    181
         AtIAA30    ----------------------------------    173
         AtIAA20    ----------------------------------    176
Glyma.13G361100.1   ----------------------------------    190
Glyma.15G012800.1   ----------------------------------    188
         AtIAA19    ----------------------------------    198
          AtIAA6    ----------------------------------    190
Glyma.07G034200.1   ----------------------------------    188
Glyma.08G207900.1   ----------------------------------    196
          AtIAA5    ----------------------------------    164
Glyma.10G162400.2   ----------------------------------    232
Glyma.20G225000.1   ----------------------------------    228
Glyma.03G247400.1   ----------------------------------    244
Glyma.19G245200.1   ----------------------------------    231
         AtIAA16    ----------------------------------    237
Glyma.10G031900.1   ----------------------------------    249
Glyma.02G142500.3   ----------------------------------    247
Glyma.19G161100.1   ----------------------------------    253
Glyma.03G158700.1   ----------------------------------    255
Glyma.10G180100.1   ----------------------------------    240
Glyma.20G210400.1   ----------------------------------    332
         AtIAA17    ----------------------------------    230
         AtIAA14    --SISGSFLFFQ*---------------------    235
          AtIAA7    ----------------------------------    244
Glyma.13G361200.5   ----------------------------------    218
Glyma.15G012700.1   ----------------------------------    190
         AtIAA15    ----------------------------------    130
Glyma.20G083900.1   ----------------------------------    141
         AtIAA33    ----------------------------------    172

       Consensus    ++XIXXSFXFFXXLYSFFFGSAIFLLVSYMFSL*    189
```
