# Supplementary material for: Identification of potential auxin response candidate genes for soybean rapid canopy coverage through comparative evolution and expression analysis
Source: Front Plant Sci. 2024 Oct 3;15:1463438. doi: 10.3389/fpls.2024.1463438 (PMC11484095; doi:10.3389/fpls.2024.1463438)
Supplement: Supplementary file 1 [file DataSheet1.zip › AppendixC_ARF_myXStringSet_full_Alignment.pdf]

```
                                   20        40        60        80       100
Glyma.01G103500.1  ------------------------------MLSRGA--------------HGEVVGSGES------------------------------GEDELYEQQW  26
Glyma.03G070500.1  ------------------------------MLSRAA--------------NGEVAGSGYS------------------------------GEDEMYEPLW  26
Glyma.07G134800.1  ---------------------------------------------------MSLNCGGC-----------------------------GGEEDELYEQLW  20
Glyma.18G184500.1  ---------------------------------------------------MSLNRGG------------------------------GGEEDELYEQLW  19
           AtARF21  ------------------------------MESGNI-----------------VNAQPKLSG------------------------IIDGSKSYMYEQLW  29
           AtARF20  ------------------------------METGNV-----------------VNAQPELSG------------------------IIDGSKSYMYEQLW  29
           AtARF15  ------------------------------METGNV-----------------VNAQPELSG------------------------IIDRSKSYMYEQLW  29
           AtARF23  ------------------------------MESGNV-----------------VNVQSELSG------------------------IIDGSKSYMYEQLW  29
           AtARF12  ------------------------------MESGNV-----------------VNAQPELSG------------------------IIDGSKSYVYEQLW  29
           AtARF22  ------------------------------MESGNI-----------------VNAQPELSG------------------------IIDGSKSYMYEQLW  29
           AtARF14  ------------------------------MESGNV-----------------VNTQPELSG------------------------IIDGSKSYMYEQLW  29
           AtARF13  ------------------------------MENNGE-----------------MNAQPELS--------------------------VDITKTYMYEKLW  27
            AtARF9  -------------------------------------------------------MANR-------------------------------GGEYLYDELW  14
Glyma.16G023600.1  ------------------------------MAHLGC---------------CNLGGPGSSG----TSL-----------------PEKGLKDDDLYRELW  34
Glyma.07G054800.1  ------------------------------MAHLEC----------------NLGGPGSSG----TSQ-----------------PEKGLKDDDLYRELW  33
Glyma.03G258300.1  ------------------------------MAGVG--------------------------------------------------------DGDLYTQLW  14
           AtARF18  ------------------------------MASVEG--------------DDDFGSSSS-----------------------------RSYQDQLYTELW  27
           AtARF11  ------------------------------MANVEA----------------DFRTSGS-------------------------------NDDELYTELW  23
Glyma.12G164100.1  ------------------------------MASA--------------------APGA--------------------------------TNDALYKELW  18
Glyma.16G000300.1  ------------------------------MASA--------------------APGA--------------------------------TNDALYKELW  18
Glyma.07G272800.1  MLGLQELVPIQAANILELQLIPH---SPLSMASTLK--------------SGGIHTGA--------------------------------INDDLYKELW  51
            AtARF1  ------------------------------MAASNH--------------SSGKPGGV--------------------------------LSDALCRELW  24
Glyma.06G164900.3  --------------------MAS---SEVTMKGNC------------------LNHNDGGATEPHSP------------------STAKDAEAALFRELW  41
Glyma.04G200600.1  --------------------MTS---LEVTMKGNC------------------LNHNDGGATEPHSP------------------STAKDAEAALFRELW  41
Glyma.05G200800.4  --------------------MAT---SEVSIKGNS------------------VNGKGDNSSGGYTNDVRNGSGGGEARNSSSSSSARDAEAALYRELW  59
Glyma.08G008100.3  -------------------MLET---VEARLR-------------------MLLHHHHPREVTWFPLRVPCG----VFGVTITSFFLVSDAEAALYRELW  55
            AtARF2  --------------------MAS---SEVSMKGNRG--------------GDNFSSSGFSDPKETRNVSVAGEGQKSNSTRSAAAERALDPEAALYRELW  63
Glyma.03G208800.1  ----------------------------------------------------------------------------------------------------  0
Glyma.07G202200.1  -----------MAGLIDLNNATE----DDEMPSSG-------------------------------------------------------SSSTVCLELW  30
Glyma.13G174000.1  -----------MAGLIDLNNATE----DDETPSSGS--------------------------------------------------SSSSSSSTVCLELW  35
Glyma.13G234200.1  ------------MGLIDLNTTED----DEAAPLSASSPASY----------SSSSSHSGISTSAST-------------------LVVPPTPPSVCLELW  55
Glyma.15G078800.1  ------------MGLIDLNTTED----DEAAPLSA------------------SSSHSGISTSAST-------------------LVVS-PPPSVCLELW  46
            AtARF3  -----------MGGLIDLNVMET----EEDETQTQTPSSAS----------GSVSPTSSSSASVSV-------------------VSSNSAGGGVCLELW  56
Glyma.12G071000.1  -------------MEIDLNHEVT----EAEKNAFCDRECEKGAG-----AGAGITCWSSSTCSSS----------------------SAACVSSSYLELW  56
Glyma.11G154632.1  -------------MEIDLNHEVT----EVEKNAFCDRECEK---------GVGVTCWSSSTCSSST-------------------SSSSALVSSSYLELW  55
Glyma.12G171000.1  -------------MEIDLNDAVTS---EAEKSASCNGECEKG------------AALSSPTCSSS--------------------GSSSTRVSSSYIELW  52
            AtARF4  -------------MEFDLNTEIA----EVEEEENDDVGVGVGGGTRIDKGRLGISPSSSSSCSSGSSSS----------------SSSTGSASSIYSELW  67
Glyma.08G100100.3  ------------------------------MKLSSP----------------GFSPPPQ-----------------------------EGEKRVLDSELW  25
Glyma.05G143800.2  ------------------------------MKLSSS----------------GFSSPPQ-----------------------------EGEKRVLDSELW  25
Glyma.13G221400.1  ------------------------------MRLSSA----------------GFSPPPQ-----------------------------EGENRVLDSELW  25
Glyma.15G091000.1  ------------------------------MRLSSA----------------DFSPPPQ-----------------------------EGEKRVLDSELW  25
            AtARF6  ------------------------------MRLSSA----------------GFNPQPH-----------------------------EGEKRVLNSELW  25
Glyma.02G281700.1  ------------------------------MKLSSS----------------GFNPPAEE----------------------------EGEKKCLNSELW  26
Glyma.14G032700.1  ------------------------------MKLSSS----------------GFNPPAEE----------------------------EGEKKCLNSELW  26
Glyma.11G204200.1  ------------------------------MKLSTS----------------GLGQQGHE----------------------------GGEKKCLNSELW  26
Glyma.18G046800.1  ------------------------------MKLSTS----------------GLGQQGHE----------------------------GGEKKCLNSELW  26
Glyma.02G239600.1  ------------------------------MKLSTS----------------GLGQQGHE----------------------------GGEKKCLNSELW  26
Glyma.14G208500.1  ------------------------------MKLSTS----------------GLGQQGHE----------------------------GGEKKCLNSELW  26
            AtARF8  ------------------------------MKLSTS----------------GLGQQGHE------------------------------GEKCLNSELW  24
Glyma.09G072200.1  ------------------------------MKAPPN----------------GYLPNSG-----------------------------EGERKTINSELW  25
Glyma.15G181000.1  ------------------------------MKAPPS----------------GYLPNSG-----------------------------EGERKTINSELW  25
Glyma.17G047100.1  ------------------------------MKAPSN----------------GYLPNSG-----------------------------EGERKTMNSELW  25
Glyma.13G112600.1  ------------------------------MKAPSN----------------GYLPNSG-----------------------------EGERKTINSELW  25
            AtARF7  ------------------------------MKAPS---------------SNGVSPNPV-----------------------------EGERRNINSELW  26
           AtARF19  ------------------------------MKAPSN----------------GFLPSSN-----------------------------EGEKKPINSQLW  25
Glyma.07G130400.1  -----------------------------------M--------------KTTQQPEAPQG---------------D--------PCEE-KKKSINPELW  27
Glyma.01G002100.1  ---------------------------------------------------------------------------------------MK-KKSSIKAELW  12
Glyma.05G221300.1  ----------------------------MKAQPPDG--------------AAAVAPNAGE----------------------------GGEKKTINAELW  30
Glyma.08G027800.1  ----------------------------MITQPPDA--------------APAVAPNSGE----------------------------GGEKKSINAELW  30
Glyma.14G217700.1  -------------------MMAS---VEEKIKTGG-----------------GMIVGGQTLAAEMKLLKEMQE--------------HSGVRKTLNSELW  47
Glyma.17G256500.1  -------------------MMAS---VEEKIKTGGV--------------GGGMVVGGQTLVAEMKLLKEMQE--------------HSGVRKTLNSELW  50
            AtARF5  -------------------MMASLSCVEDKMKTSCLVN------------GGGTITTTTSQSTLLEEMKLLKDQSG-------------TRKPVINSELW  56
Glyma.12G174100.1  ---------------------------------------------------------------------------------------MKETDKSLDPQLW  13
Glyma.13G325200.1  ---------------------------------------------------------------------------------------MKETDKSLDPQLW  13
Glyma.12G076200.1  ---------------------------------------------------------------------------------------MKEGEKVLDPQLW  13
Glyma.11G145500.1  ---------------------------------------------------------------------------------------MKEGEKVLDPQLW  13
           AtARF16  --------------------MINV------MN-----------------------------------------------------PMKGGTEKGLDPQLW  21
           AtARF10  ----------------------------------------------------------------------------------------MEQEKSLDPQLW  12
Glyma.10G210600.1  --------------------MFM---------------------------------------------------------------VMDSAERCLDSQLW  17
Glyma.20G180000.1  --------------------MFM---------------------------------------------------------------VMDSAERCLDSQLW  17
Glyma.10G053500.1  --------------------MITF------MDTKEK---------------------------------------------------SKEVESCLDPQLW  23
Glyma.13G140600.1  --------------------MITF------MDTKEK---------------------------------------------------LKEVERCLDPQLW  23
Glyma.19G181900.2  ----------MLFQEFAVRTGYCM------MESKEK---------------------------------------------------LKEVEKCLDSRLW  33
Glyma.13G084700.1  --------------------------------------------------------------------------------------MSPPQPSRVDPKIW  14
Glyma.14G166500.1  --------------------------------------------------------------------------------------MSPPQPRRVDPKIW  14
Glyma.04G254200.1  ------------------------------MPRQPP-------------------------------------------------PSPPSQPGVLDPALW  21
           AtARF17  ------------------------------MSPPSA-------------------------------------------------TAGDINHREVDPTIW  21

         Consensus  MLGLQELVPIXXXXXXXXXXXXX+--++++XX+++++++++---------+++++++++++++++++++++++---+--------+++++XXXXXXXXXW  35

                                  120       140       160       180       200
Glyma.01G103500.1  KACAGPLVDVPRVGQRVFYFPQGHMEQLEASTNQEL-NQRIPLL-KLP---TKILCRVVNVHLLAEQETDEVYAQITLVPESSQ---------DEPTNAD  112
Glyma.03G070500.1  KGCAGPLVDVPRVGQRVFYFPQGHMEQLEASTNQEL-NQRIPLL-KLP---TKILCRVVNVHLLAEQETDEVYAQITLVPESNQ---------DEPMNPD  112
Glyma.07G134800.1  KACAGPHVEVPRTGQRVFYFPQGHMEQLEVSTNQEL-NQRIPLF-KLS---SKILCRVVNVHLLAEQETDEVYAQITLVPESNQ---------TEPTSPD  106
Glyma.18G184500.1  KACAGPHVEVPRTGQRVFYFPQGHMEQLEVSTNQEL-NQRIPLF-KLP---SKILCRVVNVHLLAEQETDEVYAQITLVPESNQ---------AEPMSPD  105
           AtARF21  KLCAGPLCDIPKLGENVYYFPQGNIELVQASTREEL-NELQPIC-DLP---SKLQCRVIAIHLKVENNSDEIYAEITLMPDTTQ------------VVIP  112
           AtARF20  KLCAGPLCDIPKLGENVYYFPQGNIELVDASTREEL-NELQPIC-DLP---SKLQCRVIAIHLKVENNSDETYAEITLMPDTTQ------------VVIP  112
           AtARF15  KLCAGPLCDIPKLGEKVYYFPQGNIELVEASTREEL-NELQPIC-DLP---SKLQCRVIAIHLKVENNSDETYAKITLMPDTTQ------------VVIP  112
           AtARF23  KLCAGPLCDIPKLGEKVYYFPQGHIELVEASTREEL-NELQPNC-DLP---SKLQCRVIAIHLKVENNSDETYVEITLMPDTTQ------------VVIP  112
           AtARF12  KLCAGPLCDIPKLGEKVYYFPQGHIELVETSTREEL-NELQPIC-DLP---SKLQCRVIAIHLKVENNSDETYAEITLMPDTTQ------------VVIP  112
           AtARF22  KLCAGPLCDIPKLGEKIYYFPQGNIELVEASTREEL-NELKPIC-DLP---SKLQCRVIAIQLKVENNSDETYAEITLMPDTTQ------------VVIP  112
           AtARF14  KLCAGPLCDIPKLGEKVYYFPQGHIELVEASTREEL-NELQPIC-DFP---SKLQCRVIAIQLKVENNSDETYAEITLMPDTTQ------------VVIP  112
           AtARF13  NICAGPLCVLPKPGEKVYYFPQGHIELIENSTRDEL-DHIRPIF-DLP---SKLRCRVVAIDRKVDKNTDEVYAQISLMPDTTE------------VMTH  110
            AtARF9  KLCAGPLVDVPQAQERVYYFPQGHMEQLEASTQQVDLNTMKPLF-VLP---PKILCNVMNVSLQAEKDTDEVYAQITLIPVGTE--------VDEPMSPD  102
Glyma.16G023600.1  KLCAGPLVDVPRTGDRVFYFPQGHMEQLQASTDQEL-NQEIPHF-NLP---AKIFCRVVNIQLLAEQDTDEVYACIALLPESDQ---------TEPTNPD  120
Glyma.07G054800.1  KLCAGPLVDVPRNGDRVFYFPQGHMEQLQASTDQEL-NQEIPHF-NLP---AKIFCRVVNIQLLAEQDTDEVYACIALLPESDQ---------TEPTNPD  119
Glyma.03G258300.1  KLCAGPLVDVPRQGERVFYFPQGHMEQLQASTNQGL-NQEIPHF-NLP---PKILCRVVHIQLLAEQETDEVYARITLLPESNQ---------EEPTSPD  100
           AtARF18  KVCAGPLVEVPRAQERVFYFPQGHMEQLVASTNQGINSEEIPVF-DLP---PKILCRVLDVTLKAEHETDEVYAQITLQPEEDQ---------SEPTSLD  114
           AtARF11  KACAGPLVEVPRYGERVFYFPQGHMEQLVASTNQGVVDQEIPVF-NLP---PKILCRVLSVTLKAEHETDEVYAQITLQPEEDQ---------SEPTSLD  110
```

```
Glyma.12G164100.1  HACAGPLVTLPREGERVYYFPQGHMEQLEASMNQGL-EQQMPSF-NLP---SKILCKVVNVHLRAEPETDEVYAQITLLPEADQ---------SEVTSPD  104
Glyma.16G000300.1  HACAGPLVTLPREGERVYYFPQGHMEQLEASMNQGL-EQQMPSF-NLP---SKILCKVVNVHLRAEPETDEVYAQITLLPEADQ---------SEVTSPD  104
Glyma.07G272800.1  HACAGPLVTLPREGERVYYFPQGHMEQLEASMYEGL-EQQMPSF-NLP---SKILCKVVNVHLRAEPETDEVYAQITLLPEADQ---------SEVTSPD  137
           AtARF1  HACAGPLVTLPREGERVYYFPEGHMEQLEASMHQGL-EQQMPSF-NLP---SKILCKVINIQRRAEPETDEVYAQITLLPELDQ---------SEPTSPD  110
Glyma.06G164900.3  HACAGPLVTVPREKERVFYFPQGHIEQVEASTNQVA-DQHMPVY-DLP---PKILCRVINVQLKAEPDTDEVFAQVTLLPEPNQ--------DENAVEKE  128
Glyma.04G200600.1  HACAGPLVTVPRERERVFYFPQGHIEQVEASTNQVA-DQHMPVY-DLP---PKILCRVINVQLKAEPDTDEVFAQVTLLPEPNQ--------DENAVEKE  128
Glyma.05G200800.4  HACAGPLVTVPREGERVFYFPQGHIEQVEASTNQVA-EQHMPVY-DLP---PKILCRVINVMLKAEPDTDEVFAQVTLLPEPNQ--------DENAVEKE  146
Glyma.08G008100.3  HACAGPLVTVPRERERVFYFPQGHIEQVEASTNQVA-EQHMPVY-DLP---PKILCRVINVMLKAEPDTDEVFAQVTLLPEPNQ--------DENAVEKE  142
           AtARF2  HACAGPLVTVPRQDDRVFYFPQGHIEQVEASTNQAA-EQQMPLY-DLP---SKLLCRVINVDLKAEADTDEVYAQITLLPEANQ--------DENAIEKE  150
Glyma.03G208800.1  ----------------------------------------------------------MNVELKAEAYSDEVYAQVTLVPEVQKDNLCF----EEEVNID  38
Glyma.07G202200.1  HACAGPLISLPKKGSVVVYFPQGHLEQ-----HLH--DFPLPASANIP---SHVFCRVLDVKLHAEEGSDEVHCQVVLVPETEQVH--QK-LREGEFDAD  117
Glyma.13G174000.1  HACAGPMISLPKKGSVVVYFPQGHLEQ-----HLH--DFPLPASANIP---SHVFCRVLDVKLHAEEGSDEVYCQVVLVPESEQVQ--QK-LREGEFDAD  122
Glyma.13G234200.1  HACAGPLISLPKKGSVVVYLPQGHFE------HVQ--DFPVTAY-DIP---PHVFCRVLDVKLHAEEGSDEVYCQVLLVPESEQVE--QS-LREGEIVAD  140
Glyma.15G078800.1  HACAGPLISLPKRGSVVVYLPQGHFE------HVQ--DFPVNAF-DIP---PHVFCRVLDVKLHAEEGSDEVYCQVLLVPESEQVE--HS-LREGEIVAD  131
           AtARF3  HACAGPLISLPKRGSLVLYFPQGHLE------QAP--DFSAAIY-GLP---PHVFCRILDVKLHAETTTDEVYAQVSLLPESEDIE--RK-VREGIIDVD  141
Glyma.12G071000.1  HACAGPLTSLLKKGNVVVYFPQGHLEQVASFSPFT--PLEIPTY-DLQ---PQIFCRVVNVQLLANKENDEVYTQVTLLPQPELEG--MY-SEGKELEEL  147
Glyma.11G154632.1  HACAGPLTSLPKKGNVVVYFPQGHLEQVASFSPFT--PLEIPTY-DLQ---PQIFCRVVNVQLLANKENDEVYTQVTLLPQAELEG--MY-LEGKELEEL  146
Glyma.12G171000.1  HACAGPLTSLPKKGNVVVYFPQGHLEQAASFSPFS--PMEMPTY-DLQ---PQIFCRVVNIQLLANKENDEVYTQVTLLPQAELAG--MY-MEGKELEKL  143
           AtARF4  HACAGPLTCLPKKGNVVVYFPQGHLEQDAMVSYSS--PLEIPKF-DLN---PQIVCRVVNVQLLANKDTDEVYTQVTLLPLQEFSM--LN-GEGKEVKEL  158
Glyma.08G100100.3  HACAGPLVSLPAVGSRVVYFPQGHSEQVAVSTNREV-DGHIPNYPSLP---PQLICQLHNLTMHADTETDEVYAQMTLQPLNPCFQ----QEQKGAY-LP  116
Glyma.05G143800.2  HACAGPLVSLPAVGSRVVYFPQGHSEQVAVSTNREV-DGHIPNYPSLP---PQLICQLHNVTMHADTETDEVYAQMTLQPLNPQE-------QKEAY-LP  113
Glyma.13G221400.1  HACAGPLVSLPAVGSRVVYFPQGHSEQVAVSTNKEV-DAHIPNYPSLP---PQLICQLHNMTMHADAETDEVYAQMTLQPLNPQE-------QKEAY-LP  113
Glyma.15G091000.1  HACAGPLVSLPAVGSRVVYFPQGHSEQVAVSTNKEV-DAHIPNYPSLP---PQLICQLHNMTMHADVETDEVYAQMTLQPLNPQE-------QNEAY-LP  113
           AtARF6  HACAGPLVSLPPVGSRVVYFPQGHSEQVAASTNKEV-DAHIPNYPSLH---PQLICQLHNVTMHADVETDEVYAQMTLQPLNAQE-------QKDPY-LP  113
Glyma.02G281700.1  HACAGPLVSLPPVGSRVVYFPQGHSEQVAASTNREV-DAHIPNYPNLP---PQLICQLHNVTMHADAETDEVYAQMTLQPLSPQE-------QKEVYLLP  115
Glyma.14G032700.1  HACAGPLVSLPLVGSRVVYFPQGHSEQVAASTNREV-DAHIPNYPNLP---PQLICQLHNVTMHADAETDEVYAQMTLQPLSPQE-------QKEVYLLP  115
Glyma.11G204200.1  HACAGPLVSLPTAGTRVVYFPQGHSEQVAATTNREI-DGHIPNYPSLP---PQLICQLHNITMHADVETDEVYAQMTLQPLTPQE-------QKDTF-LP  114
Glyma.18G046800.1  HACAGPLVSLPTAGTRVVYFPQGHSEQVAATTNREI-DGHIPNYPSLP---PQLICQLHNVTMHADVETDEVYAQMTLQPLTPQE-------QKDTF-LS  114
Glyma.02G239600.1  HACAGPLVSLPTAGTRVAYFPQGHSEQVAATTNREV-DGHIPNYPSLP---PQLICQLHNVTMHADVETDEVYAQMTLQPLTPQE-------QKDTF-LP  114
Glyma.14G208500.1  HACAGPLVSLPTAGTRVVYFPQGHSEQVAATTNREV-DGHIPNYPSLP---PQLVCQLHNVTMHADVETDEVYAQMTLQPLTPQE-------QKDTF-LP  114
           AtARF8  HACAGPLVSLPSSGSRVVYFPQGHSEQVAATTNKEV-DGHIPNYPSLP---PQLICQLHNVTMHADVETDEVYAQMTLQPLTPEE-------QKETF-VP  112
Glyma.09G072200.1  HACAGPLVSLPPVGSVVVYFPQGHSEQVAASMQKEA-DF-IPSYPNLP---SKLICMLHNVALHADPETDEVYAQMTLQPVNKY--------DKEAL-LA  111
Glyma.15G181000.1  HACAGPLVSLPPVGSLVVYFPQGHSEQVAASMQKEA-DF-IPSYPNLP---SKLICMLHNVALHADPETDEVYAQMTLQPVNKY--------DKEAI-LA  111
Glyma.17G047100.1  HACAGPLVSLPPVGSLVVYFPQGHSEQVAASMQKEA-DF-IPSYPNLP---SKLICMLHNVALHADPETDEVYAQMTLQPVNKY--------EKEAI-LA  111
Glyma.13G112600.1  HACAGPLVSLPPVGSLVVYFPQGHSEQVAASMQKEA-DF-IPSYPNLP---SKLICMLHNVALHADPETDEVYAQMTLQPVNKY--------EKEAI-LA  111
           AtARF7  HACAGPLISLPPAGSLVVYFPQGHSEQVAASMQKQT-DF-IPSYPNLP---SKLICMLHNVTLNADPETDEVYAQMTLQPVNKY--------DRDAL-LA  112
          AtARF19  HACAGPLVSLPPVGSLVVYFPQGHSEQVAASMQKQT-DF-IPNYPNLP---SKLICLLHSVTLHADTETDEVYAQMTLQPVNKY--------DREAL-LA  111
Glyma.07G130400.1  QACAGPLVNLPPSGTHVIYFPQGHSEQVAASLNKDP-HSQIPNYPNLP---SKLLCLLHNLTLLADPETDEVYAQITLQPVPSF--------DKDAL-LR  114
Glyma.01G002100.1  HACAGPLVKLPPSGTHVIYFPQGHSEQVSASLNRDV-HSQIPNYPNLP---SKLLCLLHTLTLHADPQTDQVYAQITLQPLPSF--------DKDAL-LR  99
Glyma.05G221300.1  QACAGPLLNLPSPGTHVVYFPQGHSEQVAASLKKDV-DAQVPNYTNLP---SKIPCLLHNVTLHADPDTDEVYAQMTLQPVPSF--------DTDAL-LR  117
Glyma.08G027800.1  QACAGPLLNLPSPGTHVVYFPQGHSEQVAASLKKDV-DAQVPNYTNLP---SKIPCLLHNVTLHADPDTDEVYAQMALRPVPSF--------DTDAL-LR  117
Glyma.14G217700.1  HACAGPLVSLPQVGSLVFYFPQGHSEQVAASTRRTA-TSQIPNYPNLP---YQLLCQVQNVTLHADKETDEIYAQMTLQPLNSE---------REVF-PI  133
Glyma.17G256500.1  HACAGPLVSLPQVGSLVFYFPQGHSEQVAASTRRTA-TSQIPNYPNLP---SQLLCQVQNATLHADKETDEIYAQMTLQPLNSE---------REVF-PI  136
           AtARF5  HACAGPLVCLPQVGSLVYYFSQGHSEQVAVSTRRSA-TTQVPNYPNLP---SQLMCQVHNVTLHADKDSDEIYAQMSLQPVHSE---------RDVF-PV  142
Glyma.12G174100.1  HACAGGMVQMPPVNSKVFYFPQGHAEHAQSNVDFGA----ARI--PIP---PLILCCVAAVKFLADPETDEVFARLRMVPLRNSE---LD-YEDSDGNG-  99
Glyma.13G325200.1  HACAGGMVQMPPVNSKVFYFPQGHAEHAQSNVDFGA----ARI--PIP---PLILCRVAAVKFLADPETDEVFARLRLVPLRNSE---LD-YEDSDANGE  100
Glyma.12G076200.1  HACAGGMVQMPQMNSKVFYFPQGHAEHAHTNIHL-----------RLP---PFILCNVEAVKFMANPETDEVFAKLSLLPLRNSE---LG-ADSDGAGGD  95
Glyma.11G145500.1  HACAGGMVQMPQVHSKVFYFPQGHAEHAHTTIDL-----------RVP---PFILCNVEAVKFMADPETDQVFAKLSLVPLRNSE---LG-PDSDSAAGD  95
          AtARF16  HACAGGMVRMPPMNSKVFYFPQGHAENAYDCVDFG------NL--PIP---PMVLCRVLAIKYMADAESDEVFAKLRLIPLKDDE---Y--VDHEYGDGE  105
          AtARF10  HACAGSMVQIPSLNSTVFYFAQGHTEHAHAPPDF--------HAPRVP---PLILCRVVSVKFLADAETDEVFAKITLLPLPGND---LD-LENDAVLGL  97
Glyma.10G210600.1  HACAGAMVQMPPLNTKVFYFPQGHAEHAHGKVEYFG----KNHQTRVP---PLIPCRLSAMKYMADPDTDEVYVKMRLTPLREHEL--LD-SQDDCFLGN  107
Glyma.20G180000.1  HACAGAMVQMPPLNTKVFYFPQGHAEHAHGKRVDFP-----KNQTRVP---PLIPCRLSAMKYMADPDTDEVYVKMRLTPLREHEL--LD-SQDDCFLGN  106
Glyma.10G053500.1  HACAGGIVQMPAVNSKVYYFPQGHAEHACGPVNFR-------TCPKVP---PFVPCRVTAVKYRADPETDEVYAKLKLIPLNAND---VD-YDRDVVGGA  109
Glyma.13G140600.1  HACAGGMVQMPTVNTKVYYFPQGHAEHACGPVNFK-------TCPKVP---PFVPCRVVAVKYMADPETDEVYAKLKLVPLNAND---VD-YDHDVIGAE  109
Glyma.19G181900.2  HACAGGMVQMPVVNAKVFYFPQGHAEHACGPVDFR-------VYPKIP---PFIQCKVGAIKYMADPETDEVYVKLRLVPLTRNE---GD-FEDDAVGGI  119
Glyma.13G084700.1  RACAGAAVQIPKLHSRVYYFPQGHMEHA-SPSHYLS-----PLIRSLP----FVPCHVSSLDFLADPFSDEVFAKFLLTPLSQSQQQPFQ-NDTKEARND  103
Glyma.14G166500.1  RACAGAAVQIPKLHSRVYYFPQGHLEHA-SPSHYLN-----PLLRSLP----FVPCHVSSLDFLADPFSDEVFAKFLLTPLSQQ---PFP-NDTTEARNE  100
Glyma.04G254200.1  LVCAGTTVEIPTLHSRVYYFPQGHFDQASSAPRNLS-----PLLLSKP----AVLCRVESVQFLADPLTDEVFAKLILHPVADC----FA-SGPSAVAPA  107
          AtARF17  RACAGASVQIPVLHSRVYYFPQGHVEHCC------------PLLSTLPSSTSPVPCIITSIQLLADPVTDEVFAHLILQPMTQQ-----Q-FTPTNYSRF  103

        Consensus  XXCAGXXXXXPXXXXXVXYFPQGXXEX+++++XX++-+++++X++XXX---XXXXCXXXXXXXXXXXXXDEXXXXXXLXPXXXX++--++-+++++X+XX  104

                                      220                 240                 260                 280                 300
                   ''''''''''|'''''''''|'''''''''|'''''''''|'''''''''|'''''''''|'''''''''|'''''''''|'''''''''|'''''''''|

Glyma.01G103500.1  PC-----TAEPPRAPVHSFSKVLTASDTSTHGGFSVLRKHATECLPVLDMSQPTPTQELVAKDLHGYEWRFKHIFRGQPRRHLLTT--GWSTFVTSKRLV  205
Glyma.03G070500.1  PC-----TAEPPRAPVHSFSKVLTASDTSTHGGFSVLRKHAMECLPALDMSQPTPTQELVAKDLHGYEWRFKHIFRGQPRRHLLTT--GWSTFVTSKRLV  205
Glyma.07G134800.1  PC-----PAELPRPRVHSFCKVLTASDTSTHGGFSVLRKHATECLPALDMSKSTPTQELVAKDLQGFEWRFKHIFRGQPRRHLLTT--GWSTFVTSKRLV  199
Glyma.18G184500.1  PC-----PAELPSPRVHSFCKVLTASDTSTHGGFSVLRKHATECLPALDMSKSTPTQELVAKDLQGYEWRFKHIFRGQPRRHLLTT--GWSNFVTSKRLV  198
          AtARF21  TQ-----SENRFRPLVNSFTKVLTASDTSAYGGFSVPKKHAIECLPPLDMSQPLPAQEILAIDLHDNQWRFRHNYRGTPQRHSLTT--GWNEFITSKKLV  205
          AtARF20  TQ-----SENQFRPLVNSFTKVLTASDTSAYGGFFVPKKHAIECLPPLDMSQPLPAQELLAKDLHGNQWRFRHSYRGTPQRHSLTT--GWNEFTTSKKLV  205
          AtARF15  TQ-----NENQFRPLVNSFTKVLTASDISANGVFSVPKKHAIECLPPLDMSQPLPAQELLAIDLHGNQWSFRHSYRGTPQRHLLTT--GWNEFTTSKKLV  205
          AtARF23  TE-----NENQFRPIVNSFTKVLTASDTSAQGEFSVPCKHAIECLPPLDMSQPIPAQELIAIDLHGNQWRFKHSYR-VPRGD--TT--GWNAFTTSKKLV  202
          AtARF12  TQ-----NENQFRPLVNSFTKVLTASDTSAHGGFFVPKKHAIECLPSLDMSQPLPAQELLAIDLHGNQWRFNHNYRGTPQRHLLTT--GWNAFTTSKKLV  205
          AtARF22  TQ-----NENQFRPLVNSFTKVLTASDTS--GGFFVPKKHAIECLPPLDMSQPLPTQELLATDLHGNQWRFNHNYRGTPQRHLLTT--GWNAFTTSKKLV  203
          AtARF14  TQ-----NQNQFRPLVNSFTKVLTASDTSVHGGFSVPKKHAIECLPPLDMSQPLPTQEILAIDLHGNQWRFRHIYRGTAQRHLLTI--GWNAFTTSKKLV  205
          AtARF13  NT-----TMDTRRPIVYFFSKILTASDVSLSGGLIIPKQYAIECFPPLDMSQPISTQNLVAKDLYGQEWSFKHVFRGTPQRHMFTSGGGWSVFATTKRLI  205
           AtARF9  PS-----PPELQRPKVHSFSKVLTASDTSTHGGFSVLRKHATECLPPLDMTQQTPTQELVAEDVHGYQWKFKHIFRGQPRRHLLTT--GWSTFVTSKRLV  195
Glyma.16G023600.1  PN-----ISEPPKQKFHSFCKILTASDTSTHGGFSVLRKHATECLPALDMTQATPTQELAAKDLHGFEWKFKHIYRGQPRRHLLTT--GWSTFVASKRLV  213
Glyma.07G054800.1  PN-----VSEAPKQKFHSFCKILTASDTSTHGGFSVLRKHATECLPELDMTQSTPTQELAAKDLHGFEWKFKHIYRGQPRRHLLTT--GWSTFVASKRLV  212
Glyma.03G258300.1  PS-----PPETQKQVFHTFSKILTASDTSTHGGFSVLRRHATECLPQLDMTQTTPSQELVAEDLHGFEWKFKHIFRGQPRRHLLTT--GWSTFVTSKKLV  193
          AtARF18  PP-----IVGPTKQEFHSFVKILTASDTSTHGGFSVLRKHATECLPSLDMTQATPTQELVTRDLHGFEWRFKHIFRGQPRRHLLTT--GWSTFVSSKRLV  207
          AtARF11  PP-----LVEPAKPTVDSFVKILTASDTSTHGGFSVLRKHATECLPSLDMTQPTPTQELVARDLHGYEWRFKHIFRGQPRRHLLTT--GWSTFVTSKRLV  203
Glyma.12G164100.1  DP-----LPESPRCTVHSFCKTLTASDTSTHGGFSVLRRHADDCLPPLDMTQQPPWQELVATDLHGNEWHFRHIFRGQPRRHLLTT--GWSVFVSSKKLV  197
Glyma.16G000300.1  DP-----LPESPRCTVHSFCKTLTASDTSTHGGFSVLRRHADDCLPPLDMTQQPPWQELVATDLHGNEWHFRHIFRGQPRRHLLTT--GWSVFVSSKKLV  197
Glyma.07G272800.1  DP-----LPESPRVKIHSFCKTLTASDTSTHGGFSVLRRHADDCLPPLDMSQQPPWQELVATDLHGNEWHFRHIFRGQPKRHLLTT--GWSVFVSSKKLA  230
           AtARF1  AP-----VQEPEKCTVHSFCKTLTASDTSTHGGFSVLRRHADDCLPPLDMSQQPPWQELVATDLHNSEWHFRHIFRGQPRRHLLTT--GWSVFVSSKKLV  203
Glyma.06G164900.3  PP-----PPPPPRFHVHSFCKTLTASDTSTHGGFSVLRRHADECLPPLDMSKQPPTQELVAKDLHANEWRFKHIFRGQPRRHLLQS--GWSVFVSSKRLV  221
Glyma.04G200600.1  PP-----PPPPPRFHVHSFCKTLTASDTSTHGGFSVLRRHADECLPPLDMSKQPPTQELVAKDLHANEWRFKHIFRGQPRRHLLQS--GWSVFVSSKRLV  221
Glyma.05G200800.4  GP-----PAAPPRFHVHSFCKTLTASDTSTHGGFSVLRRHADECLPPLDMTKQPPTQELVAKDLHGNEWRFRHIFRGQPRRHLLQS--GWSVFVSSKRLV  239
Glyma.08G008100.3  GP-----PAPPPRFHVHSFCKTLTASDTSTHGGFSVLRRHADECLPPLDMSKQPPTQELVAKDLHANEWRFRHIFRGQPRRHLLQS--GWSVFVSSKRLV  235
           AtARF2  AP-----LPPPPRFQVHSFCKTLTASDTSTHGGFSVLRRHADECLPPLDMSRQPPTQELVAKDLHANEWRFRHIFRGQPRRHLLQS--GWSVFVSSKRLV  243
Glyma.03G208800.1  QI--------PSRNAAYSFSKILTPSDTSTHGGFSVPKKYADECFPPLDMTLQTPAQEIVAKDLNGFEWRFRHIYRGQPKRHLLTS--GWSLFVNAKKLV  128
Glyma.07G202200.1  GEEED-AEAVMKSTTPHMFCKTLTASDTSTHGGFSVPRRAAEDCFPPLDYSQQRPSQELVAKDLHGQEWRFRHIYRGQPRRHLLTT--GWSAFVNKKKLV  214
Glyma.13G174000.1  GEEED-AEAVMKSTTPHMFCKTLTASDTSTHGGFSVPRRAAEDCFPPLDYSQQRPSQELVAKDLHGQEWRFRHIYRGQPRRHLLTT--GWSAFVNKKKLV  219
Glyma.13G234200.1  GEEED-TEAIVKSTTPHMFCKTLTASDTSTHGGFSVPRRAAEDCFPPLDYSQQRPSQELVAKDLHGLEWRFRHIYRGQPRRHLLTT--GWSAFVNKKKLV  237
Glyma.15G078800.1  GEEED-TGATVKSTTPHMFCKTLTASDTSTHGGFSVPRRAAEDCFPPLDYSQQRPSQELVAKDLHGLEWRFRHIYRGQPRRHLLTT--GWSAFVNKKKLV  228
           AtARF3  GGEED-YEVLKRSNTPHMFCKTLTASDTSTHGGFSVPRRAAEDCFPPLDYSQPRPSQELLARDLHGLEWRFRHIYRGQPRRHLLTT--GWSAFVNKKKLV  238
Glyma.12G071000.1  GAEEDGDERSPTKSTPHMFCKTLTASDTSTHGGFSVPRRAAEDCFPPLDYKQQRPSQELVAKDLHGVEWKFRHIYRGQPRRHLLTT--GWSIFVSQKNLV  245
Glyma.11G154632.1  GAEEEGDDRSPTKSTPHMFCKTLTASDTSTHGGFSVPRRAAEDCFPPLDYKQQRPSQELVAKDLHDVEWKFRHIYRGQPRRHLLTT--GWSIFVSQKNLV  244
Glyma.12G171000.1  GADEEGNETTPTKSTPHMFCKTLTASDTSTHGGFSVPRRAAEDCFPPLDYKKQRPSQELVAKDLHGVEWKFRHIYRGQPRRHLLTT--GWSIFVSQKNLV  241
           AtARF4  GGEEERNGSSSVKRTPHMFCKTLTASDTSTHGGFSVPRRAAEDCFAPLDYKQQRPSQELIAKDLHGVEWKFRHIYRGQPRRHLLTT--GWSIFVSQKNLV  256
Glyma.08G100100.3  AE----LGT-PSKQPTNYFCKILTASDTSTHGGFSVPRRAAEKVFPPLDFSQQPPCQELIARDLHGNEWKFRHIFRGQPKRHLLTT--GWSVFVSAKRLV  209
Glyma.05G143800.2  AE----LGT-PSKQPTNYFCKILTASDTSTHGGFSVPRRAAEKVFPPLDFSQQPPCQELIARDLHGNEWKFRHIFRGQPKRHLLTT--GWSVFVSAKRLV  206
```

```
Glyma.13G221400.1  AE----LGT-PSKQPTNYFCKTLTASDTSTHGGFSVPRRAAEKVFPPLDFSQQPPAQELIARDLHGNEWKFRHIFRGQPKRHLLTT--GWSVFVSAKRLV  206
Glyma.15G091000.1  AE----LGT-ASKQPTNYFCKTLTASDTSTHGGFSVPRRAAEKVFPPLDFSQQPPAQELIARDLHGNEWKFRHIFRGQPKRHLLTT--GWSVFVSAKRLV  206
           AtARF6  AE----LGV-PSRQPTNYFCKTLTASDTSTHGGFSVPRRAAEKVFPPLDYSQQPPAQELMARDLHDNEWKFRHIFRGQPKRHLLTT--GWSVFVSAKRLV  206
Glyma.02G281700.1  AE----LGT-PSKQPTNYFCKTLTASDTSTHGGFSVPRRAAEKVFPPLDYSQQPPAQELIARDLHDNEWKFRHIFRGQPKRHLLTT--GWSVFVSAKRLV  208
Glyma.14G032700.1  AE----LGT-PGKQPTNYFCKTLTASDTSTHGGFSVPRRAAEKVFPPLDYSQQPPAQELIARDLHDNEWKFRHIFRGQPKRHLLTT--GWSVFVSAKRLV  208
Glyma.11G204200.1  ME----LGI-PSKQPSNYFCKTLTASDTSTHGGFSVPRRAAEKVFPPLDFSQQPPAQELIARDLHDVEWKFRHIFRGQPKRHLLTT--GWSIFVSAKRLV  207
Glyma.18G046800.1  ME----LGI-PSKQPSNYFCKTLTASDTSTHGGFSVPRRAAEKVFPPLDFSLQPPAQELIARDLHDAEWKFRHIFRGQPKRHLLTT--GWSIFVSAKRLV  207
Glyma.02G239600.1  ME----LGV-PSKQPSNYFCKTLTASDTSTHGGFSVPRRAAEKVFPPLDFSQQPPAQELIARDLHDVEWKFRHIFRGQPKRHLLTT--GWSVFVSAKRLV  207
Glyma.14G208500.1  ME----LGV-PSKQPSNYFCKTLTASDTSTHGGFSVPRRAAEKVFPPLDFSQQPPAQELIARDLHDVEWKFRHIFRGQPKRHLLTT--GWSVFVSAKRLV  207
           AtARF8  IE----LGI-PSKQPSNYFCKTLTASDTSTHGGFSVPRRAAEKVFPPLDYTLQPPAQELIARDLHDVEWKFRHIFRGQPKRHLLTT--GWSVFVSAKRLV  205
Glyma.09G072200.1  SD----MGLKQNQQPTEFFCKTLTASDTSTHGGFSVPRRAAEKIFPPLDFSMQPPAQEIVAKDLHDNTWTFRHIYRGQPKRHLLTT--GWSVFVSTKRLF  205
Glyma.15G181000.1  SD----MGLKQNQQPTEFFCKTLTASDTSTHGGFSVPRRAAEKIFPPLDFSMQPPAQEIVAKDLHDNTWTFRHIYRGQPKRHLLTT--GWSVFVSTKRLF  205
Glyma.17G047100.1  SD----IGLKQNRQPTEFFCKTLTASDTSTHGGFSVPRRAAEKILPPLDYSMQPPAQELVAKDLHDNTWAFRHIYRGQPKRHLLTT--GWSVFVSTKRLF  205
Glyma.13G112600.1  SD----MGLKQNRQPTEFFCKTLTASDTSTHGGFSVPRRAAEKIFPPLDYSMQPPAQELVAKDLHDNTWAFRHIYRGQPKRHLLTT--GWSVFVSTKRLF  205
           AtARF7  SD----MGLKLNRQPNEFFCKTLTASDTSTHGGFSVPRRAAEKIFPALDFSMQPPCQELVAKDIHDNTWTFRHIYRGQPKRHLLTT--GWSVFVSTKRLF  206
          AtARF19  SD----MGLKLNRQPTEFFCKTLTASDTSTHGGFSVPRRAAEKIFPPLDFSMQPPAQEIVAKDLHDTTWTFRHIYRGQPKRHLLTT--GWSVFVSTKRLF  205
Glyma.07G130400.1  SD----LALKSSKPQPDFFCKQLTASDTSTHGGFSVPRRAADKIFPPLDYSMQPPAQELVARDLHDTVWTFRHIYRGQPKRHLLTT--GWSLFVSGKRLL  208
Glyma.01G002100.1  SD----LALESTKPPPDFFCKQLTASDTSTHGGFSVPRRAAEKIFPPLDYSMQPPAQELVARDLHDTVWKFRHIYRGQPKRHLLTT--GWSLFVSGKRLF  193
Glyma.05G221300.1  SD----IFLRSSKPQPEFFCKQLTASDTSTHGGFSVPRRAAEKIFPPLDYSVQPPAQELVARDLHDNVWRFRHIYRGQPKRHLLTT--GWSLFIGGKRLL  211
Glyma.08G027800.1  SD----ISLKLSKPQPEFFCKQLTASDTSTHGGFSVPRRAAEKIFPPLDYSLQSPVQELVARDLHDNVWRFRHIYRGKPKRHLLTT--GWSLFISGKRLL  211
Glyma.14G217700.1  SD----FGHKHSKHPSEFFCKTLTASDTSTHGGFSVPRRAAEKLFPPLDYTIQPPTQELVVRDLHDNTWTFRHIYRGQPKRHLLTT--GWSLFVGSKRLR  227
Glyma.17G256500.1  SD----FGLKHSKHPSEFFCKTLTASDTSTHGGFSVPRRAAEKLFPPLDYTIQPPTQELVVRDLHDNTWTFRHIYRGQPKRHLLTT--GWSLFVGSKRLR  230
           AtARF5  PD---FGMLRGSKHPTEFFCKTLTASDTSTHGGFSVPRRAAEKLFPPLDYSAQPPTQELVVRDLHENTWTFRHIYRGQPKRHLLTT--GWSLFVGSKRLR  237
Glyma.12G174100.1  --------AEGSEKPA-SFAKTLTQSDANNGGGFSVPRYCAETIFPRLDYSAEPPVQTVIAKDVHGEVWKFRHIYRGTPRRHLLTT--GWSSFVNQKKLV  188
Glyma.13G325200.1  --------AEGSEKPA-SFAKTLTQSDANNGGGFSVPRYCAETIFPRLDYSAEPPVQTVIARDVHGEVWKFRHIYRGTPRRHLLTT--GWSSFVNQKKLV  189
Glyma.12G076200.1  DV----AEPSCCEKPA-SFAKTLTQSDANNGGGFSVPRYCAETIFPRLDYTAEPPVQTVVAKDVHGETWRFRHIYRGTPRRHLLTT--GWSSFVNQKKLV  188
Glyma.11G145500.1  DA----AEPSSCEKPA-SFAKTLTQSDANNGGGFSVPRYCAETIFPRLDCTAEPPVQTVVAKDVHGETWRFRHIYRGTPRRHLLTT--GWSSFVNQKKLV  188
          AtARF16  DS---NGFESNSEKTP-SFAKTLTQSDANNGGGFSVPRYCAETIFPRLDYNAEPPVQTILAKDVHGDVWKFRHIYRGTPRRHLLTT--GWSNFVNQKKLV  199
          AtARF10  TPPSSDGNGNGKEKPA-SFAKTLTQSDANNGGGFSVPRYCAETIFPRLDYSAEPPVQTVIAKDIHGETWKFRHIYRGTPRRHLLTT--GWSTFVNQKKLI  194
Glyma.10G210600.1  TN--S-GGVENQEKPPTSFAKTLTQSDANNGGGFSVPRYCAETIFPRLDYSAEPPVQTIIAKDMHGQCWKFRHIYRGTPRRHLLTT--GWSNFVNQKRLV  202
Glyma.20G180000.1  SA-GGGGVDQGQEKPPTSFAKTLTQSDANNGGGFSVPRYCAETIFPRLDYSAEPPVQTIIAKDMLGQCWKFRHIYRGTPRRHLLTT--GWSNFVNQKRLV  203
Glyma.10G053500.1  ---------ETQDKPA-SFAKTLTQSDANNGGGFSVPRYCAETIFPRLDYSVDPPVQNILAKDVHGETWKFRHIYRGTPRRHLLTT--GWSTFVNHKKLV  197
Glyma.13G140600.1  ----------TRDKPA-SFAKTLTQSDANNGGGFSVPRYCAETIFPRLDYSADPPVQNILAKDVHGETWKFRHIYRGTPRRHLLTT--GWSTFVNHKKLV  196
Glyma.19G181900.2  N------GSENKDKSP-SFAKTLTQSDANNGGGFSVPRYCAETIFPRLDYSADPPVQNILAKDVHGETWKFRHIYRGTPRRHLLTT--GWSSFVNHKKLV  210
Glyma.13G084700.1  DD---DEDREN-NGVV-SFAKILTPSDANNGGGFSVPRFCADSCFPPLDFRADPPVQLLSVADIHGVEWRFRHIYRGTPRRHLFTT--GWSKFVNHKKLV  196
Glyma.14G166500.1  EE----KDRE--NGVV-SFSKILTPSDANNGGGFSVPRYCADSCFPPLDFRADPPVQLLSVADVHGVEWRFRHIYRGTPRRHLFTT--GWSKFVNHKKLV  191
Glyma.04G254200.1  AA--SASAQTGENNVV-SFSKVLTASDANNGGGFSVPRFCADSIFPPLNFQADPPVQNLLVTDVHGFVWEFRHIYRGTPRRHLLTT--GWSTFVNNKKLV  202
          AtARF17  GR--FDGDVDDNNKVT-TFAKILTPSDANNGGGFSVPRFCADSVFPLLNFQIDPPVQKLYVTDIHGAVWDFRHIYRGTPRRHLLTT--GWSKFVNSKKLI  198

        Consensus  ++++++++X+XXXXXX+XFXKXLTXSDXXXXGGFXVXXXXAXXXXPXLDXXXXXPXQXXXXXDXXXXXWXFXHXXRGXPXRHXXXX--GWXXFXXXKXLX  192

                                       320                 340                 360                 380                 400
                   ''''''''''|''''''''''|''''''''''|''''''''''|''''''''''|''''''''''|''''''''''|''''''''''|''''''''''|''''''''''|
Glyma.01G103500.1  AGDTFVFLRGDNGELRVGVRRLARQASSMP------------------------------------SSVISSQS--------------------------  243
Glyma.03G070500.1  AGDTFVFLRGDNGELRVGVRRLARQASSMP------------------------------------SSVISSQS--------------------------  243
Glyma.07G134800.1  AGDTFVFLRGNNGELRVGVRRIAPLQSSMP------------------------------------SSVISSQS--------------------------  237
Glyma.18G184500.1  AGDTFVFLRGNNGELRVGVRRIAPVQSSMP------------------------------------SSVISSQS--------------------------  236
          AtARF21  KGDVIVFVRGETGELRVGIRRARHQQGNIP------------------------------------SSIVSIDC--------------------------  243
          AtARF20  KGDVIVFVRGETGELRVGIRRARHQQGNIP------------------------------------SSIVSIDC--------------------------  243
          AtARF15  KGDVIVFVRGETGELRVGIRRARHQQGNIP------------------------------------SSIVSIDC--------------------------  243
          AtARF23  VGDVIVFARGETGELRVGIR*-------------------------------------------------------------------------------  223
          AtARF12  AGDVIVFVRGETGELRVGIRRARHQQGNIP------------------------------------SSIVSIDC--------------------------  243
          AtARF22  AGDVIVFVRGETGELRVGIRRAGHQQGNIP------------------------------------SSIISIES--------------------------  241
          AtARF14  EGDVIVFVRGETGELRVGIRRAGHQQGNIP------------------------------------SSIVSIES--------------------------  243
          AtARF13  VGDIFVLLRGENGELRFGIRRAKHQQGHIP------------------------------------SSVISANC--------------------------  243
           AtARF9  AGDTFVFLRGENGELRVGVRRANLQQSSMP------------------------------------SSVISSHS--------------------------  233
Glyma.16G023600.1  AGDAFVFLRGEHGQLRVGVRRLARQQSPMP------------------------------------SSVISSQS--------------------------  251
Glyma.07G054800.1  AGDAFVFLRGEHGQLRVGVRRLARQQSPMP------------------------------------SSVISSQS--------------------------  250
Glyma.03G258300.1  AGDAFVFLRGENGELRVGVRRVARQQSPMP------------------------------------SSVISSQS--------------------------  231
          AtARF18  AGDAFVFLRGENGDLRVGVRRLARHQSTMP------------------------------------TSVISSQS--------------------------  245
          AtARF11  AGDAFVFLRGETGDLRVGVRRLAKQQSTMP------------------------------------ASVISSQS--------------------------  241
Glyma.12G164100.1  AGDAFIFLRGENGELRVGVRRLMRQQSNMP------------------------------------SSVISSHS--------------------------  235
Glyma.16G000300.1  AGDAFIFLRGENGELRVGVRRLMRQHSNMP------------------------------------SSVISSHS--------------------------  235
Glyma.07G272800.1  AGDAFIFLRGENGELRVGVRRVMRQQSNVP------------------------------------SSVISSHS--------------------------  268
           AtARF1  AGDAFIFLRGENEELRVGVRRHMRQQTNIP------------------------------------SSVISSHS--------------------------  241
Glyma.06G164900.3  AGDAFIFLRGENGELRVGVRRAMRQQGNVP------------------------------------SSVISSHS--------------------------  259
Glyma.04G200600.1  AGDAFIFLRGENGELRVGVRRAMRQQGNVP------------------------------------SSVISSHS--------------------------  259
Glyma.05G200800.4  AGDAFIFLRGENGELRVGVRRAMRQQGNVP------------------------------------SSVISSHS--------------------------  277
Glyma.08G008100.3  AGDAFIFLRGENGELRVGVRRAMRQQGNVP------------------------------------SSVISSHS--------------------------  273
           AtARF2  AGDAFIFLRGENGELRVGVRRAMRQQGNVP------------------------------------SSVISSHS--------------------------  281
Glyma.03G208800.1  AGDSCIFVRGESGELRVGIRRAAENLSNISQ-----------------S-----------------SSLISGHS--------------------------  168
Glyma.07G202200.1  SGDAVLFLRGEDGELRLGIRRAAQLKSGST------------------------------------FSALSGQQ--------------------------  252
Glyma.13G174000.1  SGDAVLFLRGEDGELRLGIRRAAQLKSGST------------------------------------FSALSGQQ--------------------------  257
Glyma.13G234200.1  SGDAVLFLRGDDGELRLGIRRAAQLKSAGS------------------------------------FAVPSGQQ--------------------------  275
Glyma.15G078800.1  SGDAVLFLRGNDGELRLGIRRAAQLKWAGS------------------------------------FAVPSGQQ--------------------------  266
           AtARF3  SGDAVLFLRGDDGKLRLGVRRASQIEGTAA------------------------------------LSAQYNQN--------------------------  276
Glyma.12G071000.1  SGDAVLFLRGENGELRLGIRRAVRPRNDLP------------------------------------ESVIGSQN--------------------------  283
Glyma.11G154632.1  SGDAVLFLRGENGELRLGIRRAVRPRNDLP------------------------------------ESVIGSQN--------------------------  282
Glyma.12G171000.1  SGDAVLFLRGENGELRLGIRRAARPRNGLP------------------------------------ESIVGSQS--------------------------  279
           AtARF4  SGDAVLFLRDEGGELRLGIRRAARPRNGLP------------------------------------DSIIEKNS--------------------------  294
Glyma.08G100100.3  AGDSVLFIWNEKNQLLLGIRRANRPQPVMP------------------------------------SSVLSSDS--------------------------  247
Glyma.05G143800.2  AGDSVLFIWNEKNQLLLGIRRANRPQPVMP------------------------------------SSVLSSDS--------------------------  244
Glyma.13G221400.1  AGDSVLFIWNEKNQLLLGIRRANRPQTVMP------------------------------------SSVLSSDS--------------------------  244
Glyma.15G091000.1  AGDSVLFIWNEKNQLLLGIRRANRPQTVMP------------------------------------SSVLSSDS--------------------------  244
           AtARF6  AGDSVLFIWNDKNQLLLGIRRANRPQTVMP------------------------------------SSVLSSDS--------------------------  244
Glyma.02G281700.1  AGDSVLFIWNEKNQLLLGIRRANRPQTIMP------------------------------------SSVLSSDS--------------------------  246
Glyma.14G032700.1  AGDSVLFIWNEKNQLLLGIRRANRPQTIMP------------------------------------SSVLSSDS--------------------------  246
Glyma.11G204200.1  AGDSVLFIWNEKNQLLLGIRRANRPQTVMP------------------------------------SSVLSSDS--------------------------  245
Glyma.18G046800.1  AGDSVLFIWNEKNQLLLGIRRANRPQTVMP------------------------------------SSVLSSDS--------------------------  245
Glyma.02G239600.1  AGDSVLFIWNEKNQLLLGIRRANRPQTVMP------------------------------------SSVLSSDS--------------------------  245
Glyma.14G208500.1  AGDSVLFIWNEKNQLLLGIRRANRPQTVMP------------------------------------SSVLSSDS--------------------------  245
           AtARF8  AGDSVIFIRNEKNQLFLGIRHATRPQTIVP------------------------------------SSVLSSDS--------------------------  243
Glyma.09G072200.1  AGDSVLFIRDEKQQLLLGIKRANRQQPALS------------------------------------SSVISSDS--------------------------  243
Glyma.15G181000.1  AGDSVLFIRDEKQQLLLGIKRANRQQPALS------------------------------------SSVISSDS--------------------------  243
Glyma.17G047100.1  AGDSVLFIRDEKQHLLLGIRRANRQQPALS------------------------------------SSVISSDS--------------------------  243
Glyma.13G112600.1  AGDSVLFIRDEKQHLLLGIRRANRQQPALS------------------------------------SSVISSDS--------------------------  243
           AtARF7  AGDSVLFIRDGKAQLLLGIRRANRQQPALS------------------------------------SSVISSDS--------------------------  244
          AtARF19  AGDSVLFVRDEKSQLMLGIRRANRQTPTLS------------------------------------SSVISSDS--------------------------  243
Glyma.07G130400.1  AGDSVLFIRDEKQHLLLGIRRANRQPTNIS------------------------------------SSVLSSDS--------------------------  246
Glyma.01G002100.1  AGDSVLFIRDEKQQLLLGIRRANRQPTNIS------------------------------------SSVLSSDS--------------------------  231
Glyma.05G221300.1  AGDSVLFVRDEKQQLLLGIRRANRQPSNLS------------------------------------SSVLSSDS--------------------------  249
Glyma.08G027800.1  AGDSVLFVRDEKQQLLLGIRRANRQPSNLS------------------------------------SSVLSSDS--------------------------  249
Glyma.14G217700.1  AGDSVLFIRDERSQLRVGVRRVNRQQTTLP------------------------------------SSVLSADS--------------------------  265
```

```
Glyma.17G256500.1  AGDSVLFIRDEKSQLRVGVRRVNRQQTTLP------------------------------------SSVLSADS--------------------------  268
           AtARF5  AGDSVLFIRDEKSQLMVGVRRANRQQTALP------------------------------------SSVLSADS--------------------------  275
Glyma.12G174100.1  AGDSIVFLRAENGDLCVGIRRAKRGGVGGA----EGPCG-WSSSGGGLGPGPGLGLGPGPGPYGAFSGFLREES--------KVVR-------------S  262
Glyma.13G325200.1  AGDSIVFLRAENGDLCVGIRRAKRGGVGGP----EGPCG-WSSYGSG-----GLGLG----PYGAFSGFMREE--------------------------S  249
Glyma.12G076200.1  AGDSVVFLRAENGDLCVGIRRAKKGISEGSG-SGSSSVW-SSASGSGNG---NCGIG----PYGPFSFFLKEEN--------KMLRNGCG------GNLS  265
Glyma.11G145500.1  AGDSVVFLRAENGDLCVGIRRAKKGIDEGSG-LASSSVW-SSASGSGI---------------GPFSFFLKEEN--------KMLRNGCGVG----GNLS  259
          AtARF16  AGDSIVFMRAENGDLCVGIRRAKRGGIG---NGPEYSAG-WNPIGGSCG----------------YSSLLREDE-------SNSLRRS--NC----SLAD  266
          AtARF10  AGDSIVFLRSESGDLCVGIRRAKRGGLGSNAGSDNPYPG--------------------------FSGFLRDDESTTTTSKLMMMKRNGNND----GNAA  264
Glyma.10G210600.1  AGDSIVFLRAENGDLCVGIRRAKKGIGGGT----EFSSG-WNNPLFGGG-----------------GGFLCGSE-------SSFVSGAKSGG----DHE-  268
Glyma.20G180000.1  AGDSIVFLRAENGDLCVGIRRAKKGIGGGT----EFSSGGWNNPLF--G-----------------GGFLCGSE-------SNLMSGG--------DHEM  265
Glyma.10G053500.1  AGDSIVFLRAENGDLCVGIRRAKKGICGGL----ETSSG-WNPAGGNCH-----------IPYGGFSPFFREDD-------NRISRNGNSNGLNPSVSMM  274
Glyma.13G140600.1  AGDSIVFLRAENGDLCVGIRRAKKGIGGGL----ETSSG-WNPAGGNFP-----------MPYSGFSPFLREDD-------NRILRNGNSNGLNPSVSMM  273
Glyma.19G181900.2  AGDSIVFLRAEKDDLRVGIRRAKRGI--GIGGGPEAPAG-WNSGGGIRP-----------MPYGGFSAFLREED-------SQLLRNG--------LSPN  281
Glyma.13G084700.1  AGDTVVFVKDSDGIVSVGIRRAARFAAA---IETPPPAE------------------------------------------REGFSR------------S  239
Glyma.14G166500.1  AGDTVVFVKDSDGRVSVGIRRAARFAAA---IETPQPP----------------------------------------PAEREGFSR------------S  236
Glyma.04G254200.1  AGDVVVFMKNSGGGLFVGIRRATRFSMG---KGGDRGGM-RIRVDEEEE-----------------EEEEEEEE----EEVREVFSR------------D  265
          AtARF17  AGDSVVFMRKSADEMFIGVRRTPISSSD---GGSSYYGG-DEYNGYYSQ-----------------SSVAKEDD----GSPKKTFRR------------S  261

        Consensus  XGDXXXFXXXXXXXLXXGXRRXXXXXXX+++++++++++-+++++++++-----------++++++XXXXXXXX----++++++++++++++----++++  228

                                       420                 440                 460                 480                 500
                   .........|.........|.........|.........|.........|.........|.........|.........|.........|.........|

Glyma.01G103500.1  ----MHLGVLATASHAVATQTLFVVYYKPR-T--SQFIIGVNKYLEAM-DKKFSVGMRFKMRFEGDDSAETDKRFSGTIVGVEDI--SPHWVNSKWRSLK  333
Glyma.03G070500.1  ----MHLGVLATASHAVATQTLFVVYYKPR-T--SQFIISVNKYLEAM--NRFSVGMRLKMRFEGDDSAETDKRFSGTIVGVEDI--SPHWVNSKWRSLK  332
Glyma.07G134800.1  ----MHLGVLATASHAVATQTLFVVYYKPR-T--SQFIVSVNKYLEAI-NQKCNVGMRFKMRFEGDESPENDKRFSGTILGVEDI--SPHWVNSNWRSLK  327
Glyma.18G184500.1  ----MHLGVLATASHAVATQTLFVVYYKPR-A--SQFIVSVNKYLEAI-NQKCNVGMRFKTRFEGDESPENYKRFSGTIVGVEDI--SPHWVNSNWRSLK  326
          AtARF21  ----MRHGVIASAKHAFDNQCIFIVVYKPR---SSQFIVSYDKFLDAV-NNKFNVGSRFTMRFEGDDFSE--RRYFGTIIGVSDF--SPHWKCSEWRSLE  331
          AtARF20  ----MRHGVIASAKHALDNQCIFIVVYKPR---SSQFIVSYDKFLDAM-NNKFIVGSRFTMRFEGDDFSE--RRYFGTIIGVNDF--SPHWKCSEWRSLE  331
          AtARF15  ----MRHGVIASAKHAFDNQCMFIVVYKPR---SSQFIVSYDKFLDAV-NNKFNVGSRFTMRFEGDDLSE--RRYFGTIIGVSNF--SPHWKCSDWRSLE  331
          AtARF23  ----------------------------------------------------------------------------------------------------  223
          AtARF12  ----MRHGVVASAKHAFDNQCMFTVVYKPR---SSKFIVSYDKFLDAV-NNKFNVGSRFTMRLEGDDFSE--RRCFGTIIGVSDF--SPHWKCSEWRSLE  331
          AtARF22  ----MRHGVIASAKHAFDNQCMFIVVYKPR---SSQFIVSYDKFLDAV-NNKFNVGSRFTMRFEGDDFSE--RRYFGTIIGVSDF--SPHWKCSEWRNLE  329
          AtARF14  ----MRHGIIASAKHAFDNQCMFIVVYKPR---SSQFIVSYDKFLDVV-NNKFNVGSRFTMRFEGDDFSE--RRSFGTIIGVSDF--SPHWKCSEWRSLE  331
          AtARF13  ----MQHGVIASVVNAFKTKCMFNVVYKPS---SSQFVISYDKFVDAM-NNNYIVGSRFRMQFEGKDFSE--KRYDGTIIGVNDM--SPHWKDSEWRSLK  331
           AtARF9  ----MHLGVLATARHATQTKTMFIVYYKPR-T--SQFIISLNKYLEAM-SNKFSVGMRFKMRFEGEDSPE--RRYSGTVIGVKDC--SPHWKDSKWRCLE  321
Glyma.16G023600.1  ----MHLGVLATASHAVMTRTMFLVYYKPR-T--SQFIVGLNKYLEAV-NNKFSLGMRFKMRFEGDDSPE--RRFSGTIVGVGDV--SAGWSNSQWRSLK  339
Glyma.07G054800.1  ----MHLGVLATASHAVMTRTMFLVYYKPR-T--SQFIVGLNKYLEAV-NNKFSLSMRFKMRFEGDDSPE--RRFSGTIVGVGDV--SAGWSNSQWRSLK  338
Glyma.03G258300.1  ----MHLGVLATASHAFLTSTMFVVYYKPR-T--SQFIIGVNKYLEAE-NNKFSVGMRFKMRFEVEDSPE--RRFSGTIVGVGDV--SPGWWNSQWRSLK  319
          AtARF18  ----MHLGVLATASHAVRTTTIFVVFYKPR-I--SQFIVGVNKYMEAI-KHGFSLGTRFRMRFEGEESPE--RIFTGTIVGSGDL--SSQWPASKWRSLQ  333
          AtARF11  ----MRLGVLATASHAVTTTTIFVVFYKPR-I--SQFIISVNKYMMAM-KNGFSLGMRYRMRFEGEESPE--RIFTGTIIGSGDL--SSQWPASKWRSLQ  329
Glyma.12G164100.1  ----MHLGVLATASHAIATGTLFSVFYKPR-TSRSEFIVSVNKYLEAR-SHKLSVGMRFKMRFEGDEVPE--RRFSGTIVGVGDNK-SSVWADSEWRSLK  326
Glyma.16G000300.1  ----MHLGVLATASHAIATGTLFSVFYKPR-TSRSEFIVSVNKYLEAQ-SHKLSVGMRFKMRFEGDEVPE--RRFSGTIVGVEDNK-SLVWADSEWRSLK  326
Glyma.07G272800.1  ----MHLGVLATASHAIATGTLFSVFYKPR-TSRSEFIVSVNKYLEVQ-SHKLSVGMRFKMRFEGDEIPE--RRFSGTIVGVGDNKSSSVWPDSEWRSLK  360
           AtARF1  ----MHIGVLATAAHAITTGTIFSVFYKPR-TSRSEFIVSVNRYLEAK-TQKLSVGMRFKMRFEGEEAPE--KRFSGTIVGVQENK-SSVWHDSEWRSLK  332
Glyma.06G164900.3  ----MHLGVLATAWHAILTGTIFTVYYKPR-TSPAEFIVPYDQYMESL-KNSYSIGMRFKMRFEGEEAPE--QRFTGTVVGIEDSD-PKRWRDSKWRCLK  350
Glyma.04G200600.1  ----MHLGVLATAWHAISTGTIFTVYYKPR-TSPAEFIVPYDQYMESL-KNNYSIGMRFKMRFEGEEAPE--QRFTGTIVGIEDSD-PKRWRDSKWRCLK  350
Glyma.05G200800.4  ----MHLGVLATAWHAILTGTMFTVYYKPR-TSPAEFIVPYDQYMESL-KNNYTIGMRFKMRFEGEEAPE--QRFTGTIVGIEDAD-TKRWPKSKWRSLK  368
Glyma.08G008100.3  ----MHLGVLATAWHAILTGTMFTVYYKPR-TSPAEFIVPYDQYMESL-KNNYTIGMRFKMRFEGEEAPE--QRFTGTIVGIEDAD-TKRWPKSKWRSLK  364
           AtARF2  ----MHLGVLATAWHAISTGTMFTVYYKPR-TSPSEFIVPFDQYMESV-KNNYSIGMRFKMRFEGEEAPE--QRFTGTIVGIEESD-PTRWPKSKWRSLK  372
Glyma.03G208800.1  ----MQLGILTNASNAVGNRTMFLVYYRPW-TNPFEFIVHLQTYLKST-LQDYPIGTRVQMQHEVEESLR---RLAGTIIGNEDID-SIRWPGSAWRRLK  258
Glyma.07G202200.1  ----LSPTSLMDVVNALSARCAFSIHYNPR-VSTSEFIIPIHRFLKSL-DYSYSAGMRFRMRFETEDAAER-R-FTGLIVGIADVD-PVRWPGSKWRCLM  343
Glyma.13G174000.1  ----GSPTSLMDVVNALSARCAFSIHYNPR-VSSSEFIIPIHRFVKSL-DYSYSAGMRFRMRFETEDAAER-R-FTGLIVGIADVD-PVRWPGSRWRCLM  348
Glyma.13G234200.1  ----LNPATLKGVVNALSTRCAFSVCYNPR-FSSSEFIIPVHKFLKSL-DCSYSVGMRFRMRFETEDAAER-R-CTGLIAGISDVD-PVRWLGSKWRCLL  366
Glyma.15G078800.1  ----LNPATLMDVVNALSTRCAFSVCYNPR-FSSSEFIIPVHKFLESL-DCSYSVGMRFRMRFETEDAADR-R-FTGLIAGISDVD-PVRWPGSKWRCLL  357
           AtARF3  ----MNHNNFSEVAHAISTHSVFSISYNPK-ASWSNFIIPAPKFLKVV-DYPFCIGMRFKARVESEDASER-R-SPGIISGISDLD-PIRWPGSKWRCLL  367
Glyma.12G071000.1  ----CYSNVLSSVANAISTKSKFHVFYSPR-ASHADFVVPYQKYVKSI-KNPVSIGTRFKMRFEMDESQER-RCSSGTLIATSDLD-PYRWAKSKWRCLM  375
Glyma.11G154632.1  ----CYPNVLSSVANAISTKSKFHVFYSPR-ASQADFVVPYQKYVKSI-KNPVSIGTRFKMRFEMDESQER-RCCSGMLIGTSDLD-PYRWPKSKWRCLM  374
Glyma.12G171000.1  ----YYPNFLSSVANAISAKSMFHVFYSPR-ASHADFAVPYQKYIKSI-KNPVTIGTRFKMKFEMDESPER-RCTSGIVTGMSDLD-PYKWPKSKWRCLM  371
           AtARF4  ----CS-NILSLVANAVSTKSMFHVFYSPR-ATHAEFVIPYEKYITSI-RSPVCIGTRFRMRFEMDDSPER-R-CAGVVTGVCDLD-PYRWPNSKWRCLL  384
Glyma.08G100100.3  ----MHLGLLAAAAHAAATNSRFTIFYNPR-ASPSEFVIPLAKYVKAVYHTRVSVGMRFRMLFETEESSV--RRYMGTITGISDLD-SIRWPNSHWRSVK  339
Glyma.05G143800.2  ----MHLGLLAAAAHAAATNSRFTIFYNPR-ASPSEFVIPFAKYVKAVYHTRVSVGMRFRMLFETEESSV--RRYMGTITGISDLD-SVRWPNSHWRSVK  336
Glyma.13G221400.1  ----MHLGLLAAAAHAAATNSRFTIFYNPR-ASPSEFVIPLAKYVKAVYHTRVSVGMRFRMLFETEESSV--RRYMGTITGISDLD-PVRWQNSHWRSVK  336
Glyma.15G091000.1  ----MHLGLLAAAAHAAATNSRFTIFYNPR-ASPSEFVIPLAKYVKAVYHTRVSVGMRFRMLFETEESSV--RRYMGTITGIGDLD-PVRWPNSHWRSVK  336
           AtARF6  ----MHLGLLAAAAHAAATNSRFTIFYNPR-ASPSEFVIPLAKYVKAVYHTRVSVGMRFRMLFETEESSV--RRYMGTITGICDLD-PTRWANSHWRSVK  336
Glyma.02G281700.1  ----MHIGLLAAAAHAAATNSRFTIFYNPR-ASPSEFAIPLAKYVKAVYHTRVSVGMRFRMLFETEESSV--RRYMGTITGISDLD-PVRWPNSHWRSVK  338
Glyma.14G032700.1  ----MHIGLLAAAAHAAATNSRFTIFYNPR-ASPSEFVIPLAKYVKAVYHTRVSVGMRFRMLFETEESSV--RRYMGTITGISDLD-PVRWPNSHWRSVK  338
Glyma.11G204200.1  ----MHIGLLAAAAHAAATNSCFTVFYNPR-ASPSEFVIPLSKYIKAVYHTRISVGMRFRMLFETEESSV--RRYMGTITGISDLD-SVRWPNSHWRSVK  337
Glyma.18G046800.1  ----MHIGLLAAAAHAAATNSCFTVFYNPR-ASPSEFVIPLSKYIKAVYHTRISVGMRFRMLFETEESSV--RRYMGTITGISDLD-SVRWPNSHWRSVK  337
Glyma.02G239600.1  ----MHIGLLAAAAHAAATNSCFTVFYNPR-ASPSEFVIPLSKYIKAVYHTRVSVGMRFRMLFETEESSV--RRYMGTITGISDLD-PVRWPNSHWRSVK  337
Glyma.14G208500.1  ----MHIGLLAAAAHAAATNSCFTVFYNPR-ASPSEFVIPLSKYIKAVYHTRVSVGMRFRMLFETEESSV--RRYMGTITGISDLD-PVRWPNSHWRSVK  337
           AtARF8  ----MHIGLLAAAAHASATNSCFTVFFHPR-ASQSEFVIQLSKYIKAVFHTRISVGMRFRMLFETEESSV--RRYMGTITGISDLD-SVRWPNSHWRSVK  335
Glyma.09G072200.1  ----MHIGILAAAAHAASNNSPFTIFYNPR-ASPSEFVIPLAKYNKAL-FNQVSLGMRFRMMFETEESGV--RRYMGTITGITDLD-PVRWKNSQWRNLQ  334
Glyma.15G181000.1  ----MHIGILAAAAHAASNNSPFTIFYNPR-ASPSEFVIPSAKYNKAL-YNHASLGMRFRMMFETEESGV--RRYMGTITGITDVD-PVRWKNSQWRNLQ  334
Glyma.17G047100.1  ----MHIGILAAAAHAAANNSPFTIFYNPR-ASPSEFVVPLAKYNKAM-YTQVSLGMRFRMMFETEESGV--RGYMGTITGISDLD-PVRWKSSQWRNIQ  334
Glyma.13G112600.1  ----MHIGILAAAAHAAANNSPFTIFYNPR-ASPSEFVVPLAKYNKVT-YTQVSLGMRFRMMFETEESGV--RRYMGTITGINDLD-PVRWKSSQWRNIQ  334
           AtARF7  ----MHIGVLAAAAHANANNSPFTIFYNPRWAAPAEFVVPLAKYTKAM-YAQVSLGMRFRMIFETEECGV--RRYMGTVTGISDLD-PVRWKNSQWRNLQ  336
          AtARF19  ----MHIGILAAAAHANANSSPFTIFFNPR-ASPSEFVVPLAKYNKAL-YAQVSLGMRFRMMFETEDCGV--RRYMGTVTGISDLD-PVRWKGSQWRNLQ  334
Glyma.07G130400.1  ----MHIGILAAAAHAAANNSPFTVFYNPR-TSPSEFVIPLAKYYKSVYSHQPSLGMRFRMMFETEDSGT--RRYMGTITGISDLD-PVRWKNSQWRNLQ  338
Glyma.01G002100.1  ----MHIGILAAAAHAAANNSPFTVFYNPR-ASPSEFVIPLAKYYKSVYSHQPSLGMRFRMMFETEDSGT--RRHMGTVTGISDLD-PVQWKNSQWRNLQ  323
Glyma.05G221300.1  ----MHIGVLAAAAQAVANNSPFTVFYNPR-ASPSEFVIPLAKYSKAVYSHHISPGMRFRMMFETEDSGT--RRYMGTIIGVSDLD-SVRWKNSLWRNLQ  341
Glyma.08G027800.1  ----MHIGVLAAAAQAVANNSPFTVFYNPR-ASPSEFVIPLAKYYKAVYSHHISPGMHFRMTFETEDSGT--RRYMGTIIGVSDLD-SVRWKNSLWRNLQ  341
Glyma.14G217700.1  ----MHIGVLAAAAHAAANRSPFTIFYNPR-ACPSEFVIPLAKYRKSVFGTQVSVGMRFGMMFETEESGK--RRYMGTIVGISDVD-PLRWPGSKWRNIQ  357
Glyma.17G256500.1  ----MHIGVLAAAAHAAANRSPFTIFYNPR-ACPSEFVIPLAKYRKSVFGTQVSVGMRFGMMFETEESGK--RRYMGTIVGISDVD-PLRWPGSKWRNIQ  360
           AtARF5  ----MHIGVLAAAAHATANRTPFLIFYNPR-ACPAEFVIPLAKYRKAICGSQLSVGMRFGMMFETEDSGK--RRYMGTIVGISDLD-PLRWPGSKWRNLQ  367
Glyma.12G174100.1  GRPKVSGESVREAVTLAASNQPFEVVYYPR-ANTPEFCIRTSAVRGAM-RIQWSSGMRFKMPFETEDSSRI-SWFMGTIASVQLLD-PIRWPNSPWRLLQ  358
Glyma.13G325200.1  GRAKVSGESVREAVTLAASNQAFEVVYYPR-ANTPEFCIRTSAVRGAM-RIQWCSGMRFKMPFETEDSSRI-SWFMGTIASVQVLD-PIRWPNSPWRLLQ  345
Glyma.12G076200.1  GRVKVRAEDVVEAVTLAASNKPFEVVYYPR-ASTPEFCVKASAVRAAM-RIQWCSGMRFKMAFETEDASRI-SWFMGTIASVQVVD-PIRWPNSPWRLLQ  361
Glyma.11G145500.1  GRVKVRAEDVVEAVTLAASNKAFEVVYYPR-ASTPEFCVKASSVGAAM-RIQWCSGMRFKMAFETEDATRI-SWFMGTIASVQVVD-PICWPNSPWRLLQ  355
          AtARF16  RKGKVTAESVIEAATLAISGRPFEVVYYPR-ASTSEFCVKALDARAAM-RIPWCSGMRFKMAFETEDSSRI-SWFMGTVSAVNVSD-PIRWPNSPWRLLQ  362
          AtARF10  ATGRVRVEAVAEAVARAACGQAFEVVYYPR-ASTPEFCVKAADVRSAM-RIRWCSGMRFKMAFETEDSSRI-SWFMGTVSAVQVAD-PIRWPNSPWRLLQ  360
Glyma.10G210600.1  IVGRVAPESVVEAVTCAVNGRPFEVVYYPR-ASSPEFCVKASVVKAAM-QIQWCSGMRFKMPFETEDSSRI-SWFMGTISSVQVAD-PIRWPDSPWRLLQ  364
Glyma.20G180000.1  LVGRVAAESVVEAVTCAVNGRPFEVVYYPR-ASSPEFCVKASVVKAAM-QIQWCSGMRFKMPFETEDSSRI-SWFMGTISSVQVAD-PILWPDSPWRLLQ  361
Glyma.10G053500.1  GKGKVRPEAVSEASNLAANKKPFEVVYYPR-ASTPEFCVKASLVEAAL-QIRWCSGIRFKMAFETEDSSRI-SWFMGTISSAQVAD-PLNWPNSPWRLLQ  370
Glyma.13G140600.1  GKGKVRPEAIIEAANLAANKKPFEVVYYPR-ASTPEFCVKASLVEAAM-QTRWYSGIRFKMAFETEDSSRI-SWFMGTISSVQVAD-PLNWPNSPWRLLQ  369
Glyma.19G181900.2  AKGKVRPEAVIEAATLAANMQPFEVVYYPR-ASAPEFCVKANLVRAAL-QVRWCPGMRFKMPFETEDSSRI-SWFMGTISSVNFAD-P-RWPNSPWRLLQ  376
Glyma.13G084700.1  TTGRVTAEAVAAAAESAARNAPFEVVYYPR-TGFADFVVSAEVVEESM-KCAWVGGMRVKISMETEDSSRM-TWYQGTVSSACASE------NGPWRMLQ  330
Glyma.14G166500.1  ATGRVTAEAVAAAAESAARNAPFEVVYYPR-TGFADFVVSAEVVEESM-KCAWVGGMRVKIAMETEDSSRM-TWFQGTVSSACASE------NGPWRMLQ  327
Glyma.04G254200.1  GRGKLSAKVVAEAAELAARNMPFEVVYYPK-ERWSEFVVKTEAVNEAM-KVAWSPGIRVKIAAETDDSSRV-SWCQGTVSSVALHG-NGQWRGSLWRMLQ  361
          AtARF17  GNGKLTAEAVTDAINRASQGLPFEVVFYPA-AGWSEFVVRAEDVESSM-SMYWTPGTRVKMAMETEDSSRI-TWFQGIVSSTY-QE-TGPWRGSPWKQLQ  356

        Consensus  ++++XXXXXXXXXXXXXXXXXXFXXXYXPX-+++XXFXXXXXXXXXXX+XXXXXXGXRXXXXXEXXXXXX++X+XXGXXXXXXXX+-XXXWXXSXWRXXX  314
```

```
                                         520                 540                 560                 580                 600
Glyma.01G103500.1   VQWDE-PAAVPRPDRVSPWEIEPFVAS-ASTPSVQPTMVKTKRPRPPSE---TPDVDT---------------------TSVASVFWDAGLQ--------   399
Glyma.03G070500.1   VQWDE-PAAVPRPDRVSPWEIEPFVAS-ASTPSVQPTMVKTKRPRPPSE---TPDVDT---------------------TSAASVFWDAGLQ--------   398
Glyma.07G134800.1   VQWDE-PASFPRPDRVSSWEIEHILAC-VPTTSSQPAVIKNKRPRQASE---VPDLGD--------------------TPLAAPTFWDAGLT--------   394
Glyma.18G184500.1   VQWDE-PASFPRPDRVLPWEIEPILAS-VPTTSSQTAAIKNKRPRQASE---LADLGD--------------------TPLAFPTFWDAGLT--------   393
         AtARF21   VQWDE-FASFSRPNKVSPWEIEHLVP---ALNVPRSSLLKNKRLREVN---------E------------------------FGSSSSHLLPPI------   388
         AtARF20   VQWDE-FASFSRPNKVSPWEIEHLMS---ALNVPRSSLLKNKRLREVN---------E----------------FG------------------------   378
         AtARF15   VQWDE-FASFLRPNKVSPWEIEHLMP---ALNVPRSSFLKNKRLREVN---------E------------------------FGSSSSHLLPPI------   388
         AtARF23   ----------------------------------------------------------------------------------------------------   223
         AtARF12   VQWDE-FTSFPGPKKVSPWDIEHLMP---AINVPRSFLLKNKRLREVN---------E------------------------IGSSSSHLLPPI------   388
         AtARF22   VQWDE-FASFSRPNKVSPWEIEHLMP---ALNVPRPSLLKNKRLREVN---------E------------------------IGSSSSHLLPPI------   386
         AtARF14   VQWDE-FASFPRPNQVSPWDIEHLTP---WSNVSRSSFLKNKRSREVN---------E------------------------IGSSSSHLLPPT------   388
         AtARF13   VQWDE-LSPFLRPNQVSPWDIEHLIP---SSDISQSSLKKKKHWLQLN---------E------------------------IGATLSNLW---------   385
          AtARF9   VHWDE-PASISRPNKVSPWEIEPFVN---SENVPKSVMLKNKRPRQVSE---VSALDV---------------------GITASNLWSSVLT--------   385
Glyma.16G023600.1   VQWDE-PATIPRPDRVSCWEIEPFVAS--TALNVTQPAVKGKRSRPADV---SSSA--------------------------ASGFWYHGSS--------   399
Glyma.07G054800.1   VQWDE-PATIPRPDRVSCWEIEPFVAS--TALNVTQPAVKGKRSRPADV---LSSA--------------------------ASGFWYHGSS--------   398
Glyma.03G258300.1   VQWDE-PAIIPRPERVSSWEIEPFAAS--TALNVTQQLVKSKRSRTEVS---SSEIAP---------------------NSPALAFWYRG-P--------   383
         AtARF18   VQWDE-PTTVQRPDKVSPWEIEPFLATSPISTPAQQPQSKCKRSRPIEP---SVKTP-------------------------APPSFLYSLP--------   396
         AtARF11   IQWDE-PSSIQRPNKVSPWEIEPFSPS-ALTPTPTQQQSKSKRSRPISE---ITGSP-------------------------VASSFLSSFS--------   391
Glyma.12G164100.1   VQWDE-PSSILRPDRVSPWELEPLVS---TPPTNPQPSQRNKRSRPPIL---PSTMPD----------------------SSLQGVWKSPVESAPFS---   394
Glyma.16G000300.1   VQWDE-PSSILRPDRVSPWELEPLVS---NPPTNSQPSQRNKRSRPPIL---PSTMLD----------------------SSLQGVWKSPVESAPFS---   394
Glyma.07G272800.1   VQWDE-PSSILRPDRVSSWELEPLVS---TTLANSQPTQRNKARPLIL---PSTMPD----------------------SSLQGIWKSSVESTSFS---   428
          AtARF1   VQWDE-PSSVFRPERVSPWELEPLVANS-TPSSQPQPPQRNKRPRPPGL---PSPATG-----------------P-SGPVTPDGVWKSPAD--------   401
Glyma.06G164900.3   VRWDE-TSNTPRPERVSPWKIEPALA---PPALNPLSMPRPKRPRSNAV---PSSPDS--------------------SVLTREASSKVSIDPSPAN-GF   422
Glyma.04G200600.1   VRWDE-TSNTPRPERVSPWKIEPALA---PPALNPLSMPRPKRPRSNAV---PSSPDS--------------------SVLTREASSKVSIDPSPAN-GF   422
Glyma.05G200800.4   VRWDE-TSNIPRPERVSQWKIEPALA---PPALNPLPMPRPKRPRSNVV---PSSPDS--------------------SVLTREASSKVSVDPLPTS-GF   440
Glyma.08G008100.3   VRWDE-TSNIPRPERVSQWKIEPALA---PLALNPLPMPRPKRPRSNVV---PSSPDS--------------------SVLTREA-SKVSVDPLPTS-GF   435
          AtARF2   VRWDE-TSSIPRPDRVSPWKVEPALA---PPALSPVPMPRPKRPRSNIA---PSSPDS--------------------SMLTREGTTKANMDPLPAS-GL   444
Glyma.03G208800.1   VQWDAIVEDKMHPERVCPWWIEPLESA-------------KEKKQVPAL---PTKKKG-----HALLNQRSLPGIS---GFGKNDVHQNSAGPSSQTRRA   334
Glyma.07G202200.1   VRWDD-LEVTRH-NRVSPWEIEPSGS---ASTANNLMSAGLKRTKIGLP--SAKLEFP-----------------------VSNAIGTSDFGES---LRF   410
Glyma.13G174000.1   VRWDD-LEATRH-NRVSPWEIEPSGS---ASTANNLMSAGLKRTKIGLP--SAKLDFP-----------------------VSNAIGTSDFGES---LRF   415
Glyma.13G234200.1   VRWDD-IEAARR-NRVSPWEIEPSGS---ASNSSNLMSAGLKRTRIGMT--SVKLEFP-----------------------TPDGIGASDFGES---LRF   433
Glyma.15G078800.1   VRWDD-IEAARH-NRVSPWEIEPSGS---ASNSSNLMAAGLKRNRIEMT--SAKLEFP-----------------------NPNGIQTSDFGES---LRF   424
          AtARF3   VRWDD-IVANGHQQRVSPWEIEPSGS---ISNSGSFVTTGPKRSRIGFS--SGKPDIP-----------------------VSEGIRATDFE--------   430
Glyma.12G071000.1   VRWDE-DIETNHQDRVSPWEIDPSAP---LPPLSIQSSPRLKKLRTGLQVASPSHLIT-----------------------AARGSGLVGFEES---VRS   445
Glyma.11G154632.1   VRWDE-DIETNHKDRVSPWEIDPSAP---LPPLSIQSSPRLKKLRTGLQVASPSHLIT-----------------------AARGRGLIDFEES---VRS   444
Glyma.12G171000.1   VRWDE-DIEINHQDRVSPWEVDPSAS---LPPLSIQSSRRLKKLRPGLLAAAPNHLTT-----------------------VG-SSGFMDSEES---VRS   440
          AtARF4   VRWDE-SFVSDHQERVSPWEIDPSVS---LPHLSIQSSPRPKRPWAGLLDTTPPGNPI-----------------------TKRG-GFLDFEES---VRP   453
Glyma.08G100100.3   VGWDE-STAGERQPRVSLWEIEPLTT---FPMYPSPFPLRLKRPWPPGL---PLFHGL-KDDDFG--INSSLMWL----RDTDRGLPSLNFQGI----GV   421
Glyma.05G143800.2   VGWDE-STAGERQPRVSLWEIEPLTT---FPMYPSPFPLRLKRPWPPGL---PSFHGL-KDDDFG--TNSSLMWL----RDTDRGLPSLNFQGI----GV   418
Glyma.13G221400.1   VGWDE-STAGDRQPRVSLWEIEPLTT---FPMYPSPFPLRLKRPWPPGL---PSFHAGMKDDDFG--PNSPLLWL----RDPDRGLPSLNFQGI----GI   419
Glyma.15G091000.1   VGWDE-STAGERQPRVSLWEIEPLTT---FPMYPSSFPLRLKRPWPPGL---PSFHAGMKDDDFG--LNSPLLWL----RDTDRGLQSLNFQGI----GV   419
          AtARF6   VGWDE-STAGERQPRVSLWEIEPLTT---FPMYPSPFPLRLKRPWPPGL---PSFHGL-KEDDMGMSMSSPLMW--------DRGLQSLNFQGM----GV   416
Glyma.02G281700.1   VGWDE-STAGERQPRVSLWEIEPLTT---FPMYPSPFPLRLKRPWPSGL---PSLYGL-KDGDMG--IGSPFMWLQ--GGLGDQGMQSLNFQGL----GV   422
Glyma.14G032700.1   VGWDE-STAGERQPRVSLWEIEPLTT---FPMYPSPFPLRLRRPWPSGL---PSLYGL-KDGDMG--IGSPFMWLQ--GGLGDQGMQSLNFQGL----GV   422
Glyma.11G204200.1   VGWDE-STAGERQPRVSLWEIEPLTT---FPMYPSLFPLRLKRPWHPGT---SSLHDG----RDE--ATNGLMWLR--GGPVDQGLNSLNFQGAG---GM   419
Glyma.18G046800.1   VGWDE-STAGERQPRVSLWEIEPLTT---FPMYPSLFPLRLKRPWHPGT---SSLHDG----RDE--ATNGLMWMR--GGPVDQGLNSLNFQGA----GM   418
Glyma.02G239600.1   VGWDE-STAGERQPRVSLWEIEPLTT---FPMYPSLFPLRLKRPWHPGT---SSFHDG----RDE--ATNGLMWLR--GGPGDQALNSLNFQGS----GL   418
Glyma.14G208500.1   VGWDE-STAGERQPRVSLWEIEPLTT---FPMYPSLFPLRLKRPWHPGT---SSFHDG----RDE--ATNGLMWLR--GGPGDQALNSLNFQGS----GL   418
          AtARF8   VGWDE-STAGERQPRVSLWEIEPLTT---FPMYPSLFPLRLKRPWHAGT---SSLPDG----RGD--LGSGLTWLRG-GGGEQQGLLPLNYPSV----GL   417
Glyma.09G072200.1   VGWDE-STAGERPSRVSIWDIEPVVTP--FYICPPPF-FRPKFPKQPGM---PDDESD-----IENAFKRAMPWLG--DDLGMKDASSSVFPGF----SL   416
Glyma.15G181000.1   VGWDE-STAGERPSRVSIWDIEPVVTP--FYICPPPF-FRPKFPKEPGM---PDDESD-----IENAFKRAMPWLG--DDLGMKDASSSVFPGF----SL   416
Glyma.17G047100.1   VGWDE-STAGERPRRVSIWEIEPVVTP--FYICPPPF-FRPKFPRQPGM---PDDESD-----MENAFKRAVPWLG--DDFGMKDASSSIFPGF----SL   416
Glyma.13G112600.1   VGWDE-STAGERPSRVSIWEIEPVVTP--FYICPPPF-FRPKFPRQPGM---PDDESD-----MENAFKRAVPWLG--DDFGMKDASSSIFPGF----SL   416
          AtARF7   IGWDE-SAAGDRPSRVSVWDIEPVLTP--FYICPPPF-FRPRFSGQPGM---PDDETD-----MESALKRAMPWLD--NSLEMKDPSSTIFPGL----SL   418
         AtARF19   VGWDE-STAGDRPSRVSIWEIEPVITP--FYICPPPF-FRPKYPRQPGM---PDDELD-----MENAFKRAMPWMG--EDFGMKDAQSSMFPGL----SL   416
Glyma.07G130400.1   VGWDE-STAGEKRSRVSLWEIEPVTAP--FFICPPPF-FRSKRPRQPGM---PDDELS----DFDNIFKRTMPWLG--DDMCMKDP--QGLPGL----SL   419
Glyma.01G002100.1   VGWDE-STAGEKRSRVSIWEIEPVTAP--FFICPPPF-FRSKRPRQPGM---PDDELS----DFDNIFKQTMPWPG--DDMCVKDP--QGLPGL----NL   404
Glyma.05G221300.1   VGWDE-STAEERQSRVSVWEIEPVTTP--YFICPPPF-FRSKIPRLLGM---PDDEPD-----FNNLFKSTVPWLG--DDMCVKGP--QALPGL----SL   421
Glyma.08G027800.1   VGWDE-STAEDRRSRVSVWEIEPVTTP--YFICPPPF-FRSKRPRLLGM---PDDEPD-----FNNLFKSTVPWLG--DDMCIKDP--QALPGL----SL   421
Glyma.14G217700.1   VEWDE-PGCGDKQNRVSVWEIETPES---LFIFPSLT-SGLKRPL-PSG---LL-ENE-----WGTLLRRPFIRV----PENGTMELSNSIPNL----YS   434
Glyma.17G256500.1   VEWDE-PGCGDKQNRVSVWEIETPES---LFIFPSLT-SGLKRPL-PSG---LL-ENE-----WGTLLTRPFIRV----PENGTMELSNSIPNL----YS   437
          AtARF5   VEWDE-PGCNDKPTRVSPWDIETPES---LFIFPSLT-SGLKRQLHPSY---FAGETE-----WGSLIKRPLIRVP--DSANGIMPYA-SFPSM----AS   447
Glyma.12G174100.1   VTWDE-PDLLHNVKRVSPWLVELVSNV--PIIHLAPFSPPRKKLRFPQH---PEFPLD---------FQ-----------FPIPSFSGNPFGSS-TSSPL   431
Glyma.13G325200.1   VSWDE-PDLLHNVKRVSPWLVELVSNV--PIIHLAAFSPPRKKLRFP------------------LDVQ-----------FPIPSFSGNPFGSSSSSSPF   413
Glyma.12G076200.1   VTWDE-PDLLQNVKRVSPWLVELVSNI--PLINFTPFSPPRKKLR-PQH---PDFPLD---------VQ-----------FPIPMFSGNQLGPN---SPL   431
Glyma.11G145500.1   VTWDE-PDLLQNVKRVSPWLVELVSNI--PLINFTPFSPPRKKLR-PQH---PDFPLD---------VQ-----------FPIPMLSGNQHGPN---SPL   425
         AtARF16   VAWDE-PDLLQNVKRVNPWLVELVSNV--HPIPLTSFSPPRKKMRLPQH---PDYNNL--------INS-----------IPVPSFPSNPLIRS---SPL   434
         AtARF10   VAWDE-PDLLQNVKRVSPWLVELVSNM--PTIHLSPF-SPRKKIRIPQP---FEFPFH--------GTKFPIF---------SPGFANNGGG-----ESM   431
Glyma.10G210600.1   VVWDE-PDLLQNVKCVNPWLVELVSNM--PTFNLSAYSPPRKKQRFLQD---PYFQVI---------NQ-----------LPMPSFSSNLLNYT---NSL   435
Glyma.20G180000.1   VVWDE-PDLLQNVKCVNPWLVELVSNM--PTFNLSAYSPPRKKQRFLQD---PYFQVI---------NQ-----------LPMPSFSSNLLNYT---NSI   432
Glyma.10G053500.1   VTWDE-PDLLQNVRRVSPWLVELVSNM--PAIHFSPFSPPRKKLRLPQQ---PDFPLD---------GQ-----------IPLSTFPSNLLGPS-NTNQF   443
Glyma.13G140600.1   VTWDE-PDLLQNVRRVSPWLVELVSNM--PAIHFSPFSPPRKKLRLPQH---PDFPLD---------GQ-----------IPLPTLPNNLLGPN-NTNQF   442
Glyma.19G181900.2   VTWDE-PELLQNVKRVSPWLVEIVSNM--PTIHLSHYSTQQKKPRFPQH---PDFSFD---------GQ-----------ISLPAFPSNFLGPS---NPF   447
Glyma.13G084700.1   VNWDE-PEVLQNAKQVSPWQVELVSPP--FALH-TVF-SPNKRLRADQG---SGLLSN-----REQDPFFPMPGFSNSAMGHMTGFPNSTVGQM--DKPL   415
Glyma.14G166500.1   VNWDE-PEVLQNAKRVSPWQVELVSLP--FALH-TVY-SPNKRLRSDQG---SGLLSN-----REGDPFFPMTGFPNSTMEHMTGFPNSTVGHM--DKSL   412
Glyma.04G254200.1   VTWDE-PEGLQIAKWVSPWQVELVSTT--PALH-SAF-PPIKRIKAAHD---SGVFTN-----GERDP------------FPMTGFTNSTMGQL--NQAL   434
         AtARF17   ITWDE-PEILQNVKRVNPWQVEIAAHA--TQLH-TPF-PPAKRLKYPQP--GGGFLSG-----DDGEIL-----------YPQSGLSSAAAPDP--SPSM   431

       Consensus   VXWDX-XXXXXX+XXVXXWXXXXXXX+-+XXXX+XXX+XXXKXXX+XX++++++++++-+++++++++++++++++--++++XXXX++XXXX++++++++   362

                                         620                 640                 660                 680                 700
Glyma.01G103500.1   ----------------------------------------------------------------------------------------------------   399
Glyma.03G070500.1   ----------------------------------------------------------------------------------------------------   398
Glyma.07G134800.1   ----------------------------------------------------------------------------------------------------   394
Glyma.18G184500.1   ----------------------------------------------------------------------------------------------------   393
         AtARF21   --LTQGQEIGQ-------------LSVAS-----------------------------------------------------PMN---------------   405
         AtARF20   ------QEIGQ-------------LSVAS-----------------------------------------------------PMN---------------   391
         AtARF15   --LTQGQEIGQ-------------LSVAS-----------------------------------------------------PMN---------------   405
         AtARF23   ----------------------------------------------------------------------------------------------------   223
         AtARF12   --LTQGQENEQ-------------LSVAS-----------------------------------------------------PMN---------------   405
         AtARF22   --LTQGQEIGQ-------------LSVAS-----------------------------------------------------PMN---------------   403
         AtARF14   --LTQGQEIGQ-------------QSMAT-----------------------------------------------------PMN---------------   405
         AtARF13   ----TCQEIGQ-------------RSMNS-----------------------------------------------------PIS---------------   400
          AtARF9   ----------------------------------------------------------------------------------------------------   385
Glyma.16G023600.1   ----------------------------------------------------------------------------------------------------   399
Glyma.07G054800.1   ----------------------------------------------------------------------------------------------------   398
Glyma.03G258300.1   ----------------------------------------------------------------------------------------------------   383
         AtARF18   ----------------------------------------------------------------------------------------------------   396
         AtARF11   ----------------------------------------------------------------------------------------------------   391
Glyma.12G164100.1   ----------------------------------------------------------------------------------------------------   394
```

```
Glyma.16G000300.1  ----------------------------------------------------------------------------------------------------  394
Glyma.07G272800.1  ----------------------------------------------------------------------------------------------------  428
           AtARF1  ----------------------------------------------------------------------------------------------------  401
Glyma.06G164900.3  PRVLQGQE--------------------------------------------------------------------------------------------  430
Glyma.04G200600.1  PRVLQGQE--------------------------------------------------------------------------------------------  430
Glyma.05G200800.4  QRVLQGQE--------------------------------------------------------------------------------------------  448
Glyma.08G008100.3  QRVLQGQE--------------------------------------------------------------------------------------------  443
           AtARF2  SRVLQGQE--------------------------------------------------------------------------------------------  452
Glyma.03G208800.1  DGDLQGQDYSG----------------LS-----------------------------------------------------PPQ---------------  350
Glyma.07G202200.1  QKVLQGQEMLG---------------VNP-----------------------------------------------------T-----------------  425
Glyma.13G174000.1  QKVLQGQEMLG---------------VNT-----------------------------------------------------T-----------------  430
Glyma.13G234200.1  RKVLQGQEILG---------------VNT-----------------------------------------------------P-----------------  448
Glyma.15G078800.1  RKVLQGQEILG---------------VNT-----------------------------------------------------P-----------------  439
           AtARF3  ----------------------------------------------------------------------------------------------------  430
Glyma.12G071000.1  PKVLQGQENAG---------------FVS-----------------------------------------------------FYY---------------  462
Glyma.11G154632.1  PKVLQGQENAG---------------FGS-----------------------------------------------------LYY---------------  461
Glyma.12G171000.1  SKVLQGQENTG---------------FMS-----------------------------------------------------LYY---------------  457
           AtARF4  SKVLQGQENIG---------------SAS-----------------------------------------------------PSQ---------------  470
Glyma.08G100100.3  SPWMQPRLDPS---------------MVN-----------------------------------------------------------------------  435
Glyma.05G143800.2  SPWLQPRLDPS---------------MVN-----------------------------------------------------------------------  432
Glyma.13G221400.1  NPWMQPRFDPT---------------MLN-----------------------------------------------------------------------  433
Glyma.15G091000.1  NPWMQPRFDPT---------------VLN-----------------------------------------------------------------------  433
           AtARF6  NPWMQPRLDTS--------------GLLG-----------------------------------------------------------------------  431
Glyma.02G281700.1  TPWMQPRLDPS---------------IPG-----------------------------------------------------------------------  436
Glyma.14G032700.1  TPWMQPRLDAS---------------IPG-----------------------------------------------------------------------  436
Glyma.11G204200.1  LPWMQQRLDPT--------------LLGN-----------------------------------------------------------------------  434
Glyma.18G046800.1  LPWMQQRLDPT--------------LLGN-----------------------------------------------------------------------  433
Glyma.02G239600.1  LPWMQQRMDPT--------------LLAN-----------------------------------------------------------------------  433
Glyma.14G208500.1  LPWMQQRMDPT--------------LLGN-----------------------------------------------------------------------  433
           AtARF8  FPWMQQRLDLS--------------QMGT-----------------------------------------------------------------------  432
Glyma.09G072200.1  MQWMSMQQNNQ---FSAAQSGFIPPSMLS-SNALHGNL-TTDDPSKLLSFQAPV--LSSPNLQFNKPNLANQVN---QLQQSPTSWS-------------  493
Glyma.15G181000.1  MQWMSMQQNNQ---FSAAQSGFIPPSMLS-SNALHGNL-TTDDPSKLLSFQAPV--LSSPNLQFNKPNLANQVN---QLQQSPTSW--------------  492
Glyma.17G047100.1  MQWMSMQQNNQ---LSAAQSGCF-PSMLS-SNTLQGNL-STDDPSKLLSFQAPV--LSTPSLQLNKPNLPNQIN---QLQQSPVSWP-------------  492
Glyma.13G112600.1  VQWMSMQQNNQ---LTAAQSGCF-PSMLP-FNTLQGNL-STDDPSKLLSFQAPV--LSTPSLQLNKPNLPNQIN---QLQQSPVSWP-------------  492
           AtARF7  VQWMNMQQQNGQLPSAAAQPGFF-PSMLSPTAALHNNLGGTDDPSKLLSFQTPHGGISSSNLQFNKQNQQAPMS---QLPQPPTTLS--QQQQLQQLLHS  512
          AtARF19  VQWMSMQQNNP---LSGSATPQL-PSALS-SFNLPNNF-ASNDPSKLLNFQSPN--LSSANSQFNKPNTVNHISQ--QMQAQPAMVKSQQQQQQQQQQHQ  506
Glyma.07G130400.1  AQWMNMQQNPA---LANSLQPNYAPSLSG---SILQNI-PGADISRQLGFSAPQ-ISQSDNVALNTQRLLQTAQQLDHLQKLPSTSS-------------  498
Glyma.01G002100.1  AQWMNMQQNPA---LASSLQPNYAPSLSG---SILQNI-PGPDISHQLGFSAPQ-ISQSNNVALNTQRLLQTAPQLDHLQKLPSTSS-------------  483
Glyma.05G221300.1  VQWMNIQQNPA---LASSLQPNCGPSMSG---LVLQNL-PGADIANPLGFSTSQ-ISQSNNVSVDAQNILQTSQQLDHIQKPPCPSS-------------  500
Glyma.08G027800.1  VQWMNMQQNPA---LASSLQPNCVPSMSG---LVLQNL-PGADIANQLGFSTSQ-TSQSNNVSVNAQNILQTSQQLDHIQKLPCPSS-------------  500
Glyma.14G217700.1  EHMMRMLLKPQ--------LINNNGAFLS-----------------------------------------------------------------------  455
Glyma.17G256500.1  EHMMKMLFKPQ--------LINNNGAFLS-----------------------------------------------------------------------  458
           AtARF5  EQLMKMMMRPH--------NNQNVPSFMS-----------------------------------------------------------------------  468
Glyma.12G174100.1  CCLS---DNAP-----------------------------------------------------------------------------------------  439
Glyma.13G325200.1  CCLS---DNAP-----------------------------------------------------------------------------------------  421
Glyma.12G076200.1  CGFS---DNAP-----------------------------------------------------------------------------------------  439
Glyma.11G145500.1  CGFS---DNAP-----------------------------------------------------------------------------------------  433
          AtARF16  SSVI---DNVP-----------------------------------------------------------------------------------------  442
          AtARF10  CYLSNDNNNAP-----------------------------------------------------------------------------------------  442
Glyma.10G210600.1  CTIQ--DSNSS-----------------------------------------------------------------------------------------  444
Glyma.20G180000.1  CTIE--DNNSS-----------------------------------------------------------------------------------------  441
Glyma.10G053500.1  GCLI---ESTP-----------------------------------------------------------------------------------------  451
Glyma.13G140600.1  GCLI---ESTP-----------------------------------------------------------------------------------------  450
Glyma.19G181900.2  GCLA---ESTP-----------------------------------------------------------------------------------------  455
Glyma.13G084700.1  LSY----ESFP-----------------------------------------------------------------------------------------  422
Glyma.14G166500.1  LSY----DTFP-----------------------------------------------------------------------------------------  419
Glyma.04G254200.1  LSY----GTFP-----------------------------------------------------------------------------------------  441
          AtARF17  FSY----STFP-----------------------------------------------------------------------------------------  438

        Consensus  +++++++++++---+++++++++++++++-++++++++-+++++++++++++++-++++++++++++++++++++++++++++++++-------------  362
```

```
                                       720                 740                 760                 780                 800

Glyma.01G103500.1  ---------------------------------------------------------------QADMAQKNVLAESKW----------------------  414
Glyma.03G070500.1  ---------------------------------------------------------------QADMAQKNVLAESKR----------------------  413
Glyma.07G134800.1  ---------------------------------------------------------------QCDMTQLRVMTESKR----------------------  409
Glyma.18G184500.1  ---------------------------------------------------------------QSDMAKLSVMAEIKR----------------------  408
          AtARF21  --------------------------------------------------------------I-SLRYRDTTEAAMNPS---------------------  421
          AtARF20  --------------------------------------------------------------T-SLRYRDTTEDAMNPS---------------------  407
          AtARF15  --------------------------------------------------------------I-SLLYRETTEDAMNPS---------------------  421
          AtARF23  ----------------------------------------------------------------------------------------------------  223
          AtARF12  --------------------------------------------------------------I-SLRYRDATEDAMNPS---------------------  421
          AtARF22  --------------------------------------------------------------I-SLTYRDTTEDVMNPS---------------------  419
          AtARF14  --------------------------------------------------------------I-SLRYRDITEDAMTPS---------------------  421
          AtARF13  --------------------------------------------------------------VPEFSYPNAIEDSKFLS---------------------  417
           AtARF9  ---------------------------------------------------------------QPHEFAQSCIT-SQW----------------------  399
Glyma.16G023600.1  ---------------------------------------------------------------NELSQLGAATAEVQS----------------------  414
Glyma.07G054800.1  ---------------------------------------------------------------NELSQLGAA-AEVQS----------------------  412
Glyma.03G258300.1  ---------------------------------------------------------------QDPTALGSS-TEVQS----------------------  397
          AtARF18  ---------------------------------------------------------------QSQDSINASLK--------------------------  407
          AtARF11  ---------------------------------------------------------------QSHESNPSVKL--------------------------  402
Glyma.12G164100.1  --------------------------------------------------------------YCDHQHGRDVYPSTNF----------------------  410
Glyma.16G000300.1  --------------------------------------------------------------YRDHQHGRDVYPSTNF----------------------  410
Glyma.07G272800.1  --------------------------------------------------------------YCDPQQGRGLYPSPKF----------------------  444
           AtARF1  --------------------------------------------------------------------TPSSVPLFSP----------------------  411
Glyma.06G164900.3  --------------------------------------------------------------FSTLRGNFTESNEFDT----------------------  446
Glyma.04G200600.1  --------------------------------------------------------------FSTLRGNFAESNESDT----------------------  446
Glyma.05G200800.4  --------------------------------------------------------------LSTLRGNFAESNESDT----------------------  464
Glyma.08G008100.3  --------------------------------------------------------------LSTLRGNFAESNESDT----------------------  459
           AtARF2  --------------------------------------------------------------YSTLRTKHTESVECDA----------------------  468
Glyma.03G208800.1  -------------------------------------------------------------PLQRAPSTDIIRPSKVP----------------------  367
Glyma.07G202200.1  --------------------------------------------------------------YDSINAQSHQVSDLRR----------------------  441
Glyma.13G174000.1  --------------------------------------------------------------YDSFNAQSHQLSDLRR----------------------  446
Glyma.13G234200.1  --------------------------------------------------------------FDGINAQSPRLYELGR----------------------  464
Glyma.15G078800.1  -------------------------------------------------------------PFDGINAQSPWLYELGR----------------------  456
           AtARF3  ----------------------------------------------------------------------ESLRFQRV----------------------  438
Glyma.12G071000.1  -------------------------------------------------------------GCDTVTKP-PGFEMSSP----------------------  478
Glyma.11G154632.1  -------------------------------------------------------------GCDTVTKP-PGFEMSSQ----------------------  477
Glyma.12G171000.1  -------------------------------------------------------------GCDTVTKQ-PEFEIRSP----------------------  473
           AtARF4  -------------------------------------------------------------GFDVMNRRILDFAMQSH----------------------  487
Glyma.08G100100.3  -------------------------------------------------YQSDMYQAMAAAALQDMWTSNPSKQHPT-----------------------  463
Glyma.05G143800.2  -------------------------------------------------YQSDMYQAMAAAALQDMWTSDPSKQHPT-----------------------  460
Glyma.13G221400.1  -------------------------------------------------MQTDMYQ---AAAVQDMRSLDPSKQHSA-----------------------  458
```

```
Glyma.15G091000.1 -------------------------------------------------MQTDMYQAAAAAAVQDMRSLDPSKQLSA----------------------- 461
           AtARF6 -------------------------------------------------MQNDVYQAMAAAALQDMRGIDPAKA-AA----------------------- 458
Glyma.02G281700.1 -------------------------------------------------LQPELYQAITSSAFQEMRTMDLSKS-SQ----------------------- 463
Glyma.14G032700.1 -------------------------------------------------LQPELYQAMASSAFQEIRTMDPSKS-SQ----------------------- 463
Glyma.11G204200.1 -------------------------------------------------DQNQQYQAMLAAGLQNLGSGYLMKQ-------------------------- 459
Glyma.18G046800.1 -------------------------------------------------DQNQQYQAMLAAGLQNLGSGYLMKQ-------------------------- 458
Glyma.02G239600.1 -------------------------------------------------DHNQHYQAMFASGLQNLGSGDLMRQ-------------------------- 458
Glyma.14G208500.1 -------------------------------------------------DHNQQYQAMFASGLQNLGSGDLMRQ-------------------------- 458
           AtARF8 -------------------------------------------------DNNQQYQAMLAAGLQNIGGGDPLRQ-------------------------- 457
Glyma.09G072200.1 ----------------------PQQQQQ---------------------QQQQKLQSMLQTPLNPL---QQQRQQQL-----PVPQNLPQPQQQQPQMP- 541
Glyma.15G181000.1 -----------------------PPQQQ---------------------QQQQKLQSMLQTPLNPLQQQQQQRQQQL-----PVPQNLSQPQQQQPQMP- 542
Glyma.17G047100.1 -QQQQLQQQQQQQQQQQQQQQQQQQQQ---------------------QQQQQLQSLLQIPMNQL---QQQRQQQL-----PEPQNLP----LLQQLP- 557
Glyma.13G112600.1 -QQQQLQQQQQLQQQQQQQQQQQQQLQQ---------------------QQQQQLQSLLQVPMNHL---QQQREQQL-----PEPQNLP----LWQQLP- 557
           AtARF7 SLNHQQQQSQSQQQQQQQQLLQQQQQLQSQQHSNNNQSQSQQQQQLLQQQQQQQLQQQHQQPLQQQTQQQQLRTQPLQSHSHPQPQQLQQHKLQQLQVPQ 612
          AtARF19 HQQQQLQQQQQL---------------Q---------------------MSQQQVQ-------------------------------------------- 526
Glyma.07G130400.1 -------------------------------------------TLGTVLLPQQQLGDITQQPRQNL------ANQTI----------------------- 526
Glyma.01G002100.1 -------------------------------------------TLGTVLPPQQQLGDITQQSRQNL------ANQTI----------------------- 511
Glyma.05G221300.1 -------------------------------------------ALGAVTQPLQQLGDITQQP-RNL------TNQTL----------------------- 527
Glyma.08G027800.1 -------------------------------------------ALGAVTQLPQQLADITQQP-RNL------TNQTL----------------------- 527
Glyma.14G217700.1 -------------------------------------------------AMQQESAATR-GPLQEMKTTLAAENQMP----------------------- 482
Glyma.17G256500.1 -------------------------------------------------AMQQESAATR-GPLQEMKTTLAAENQML----------------------- 485
           AtARF5 -------------------------------------------------EMQQNIVMGNGGLLGDMKMQQPLMMNQK----------------------- 496
Glyma.12G174100.1 ------------------------------------------------------------AGIQGARH--AQIG-------------------------- 451
Glyma.13G325200.1 ------------------------------------------------------------AGIQGARH--SQIG-------------------------- 433
Glyma.12G076200.1 ------------------------------------------------------------AGIQGARH--AQFG-------------------------- 451
Glyma.11G145500.1 ------------------------------------------------------------AGIQGARH--AQFG-------------------------- 445
          AtARF16 ------------------------------------------------------------VGLQGARHNAHQYYG------------------------- 457
          AtARF10 ------------------------------------------------------------AGIQGARQAQQLFGSPSP---------------------- 460
Glyma.10G210600.1 ------------------------------------------------------------GGIQGARH--AQFG-------------------------- 456
Glyma.20G180000.1 ------------------------------------------------------------GGIQGARH--PQFG-------------------------- 453
Glyma.10G053500.1 ------------------------------------------------------------AGMQGARH--AHYG-------------------------- 463
Glyma.13G140600.1 ------------------------------------------------------------AGMQGARH--AHYG-------------------------- 462
Glyma.19G181900.2 ------------------------------------------------------------AGIQGARH--ANYG-------------------------- 467
Glyma.13G084700.1 ------------------------------------------------------------AGMQGARH--DLYSP------------------------- 435
Glyma.14G166500.1 ------------------------------------------------------------AGMQGARH--DLFSP------------------------- 432
Glyma.04G254200.1 ------------------------------------------------------------AGMQGARH--DAFSA------------------------- 454
          AtARF17 ------------------------------------------------------------AGMQGARQ--YDFGS------------------------- 451

        Consensus -+++++++++++----------++++++---------------+++++++++++++++++++++XX++++++XX+++++---+++++++----++++++- 366

                                     820                 840                 860                 880                 900
Glyma.01G103500.1 ----------------NDNTGTWHHMQT-DMNSKSNSGNT---------MLRNQTEGSWLS---------------------------------SPH-SS 454
Glyma.03G070500.1 ----------------NDNTGTWHHMQT-DMNSKSNSGNA---------MLRNQTEGSWLS---------------------------------SPH-SS 453
Glyma.07G134800.1 ----------------SDSSSHMRHHNS-KSNNNGI--------------SMNQTEASWLS---------------------------------SP---- 441
Glyma.18G184500.1 ----------------SDSSSHMWHHNS-KSSNNGI--------------SMNQTEASWLS---------------------------------SP---- 440
          AtARF21 --------------------------------------------------------RLLMSY----------------------------PVQPMPKLNY 437
          AtARF20 --------------------------------------------------------RLLMSY----------------------------PVQPMPKLNY 423
          AtARF15 --------------------------------------------------------RLLMSY----------------------------PVQPMPKRNY 437
          AtARF23 ---------------------------------------------------------------------------------------------------- 223
          AtARF12 --------------------------------------------------------KLLMSY----------------------------PVQPMPKLNY 437
          AtARF22 --------------------------------------------------------RLLMSY----------------------------PVQPMPKLNY 435
          AtARF14 --------------------------------------------------------RLLMSY----------------------------PVQPMAKLNY 437
          AtARF13 --------------------------------------------------------GLLLNH----------------------------SLLAIPNENY 433
           AtARF9 ----------------SSPQQCHRDANE-DAKKSD-----------------------WLN---------------------------------NS---- 422
Glyma.16G023600.1 ----------------KENQVVPCSLRQ-KDIINSNPIDAN----NSSISSRVRMEGVWPS---------------------------------SPH-LN 459
Glyma.07G054800.1 ----------------KENQVVPCSLRQ-KDIINSNPINAN----NSSISTRVRMEGVWPS---------------------------------SPH-LN 457
Glyma.03G258300.1 ----------------NENPVVW-STRQ-KE-INGNPMNS---------SSKVRVEGMRPS---------------------------------SPH--- 433
          AtARF18 ---------------------------------------------------------------------------------------------------- 407
          AtARF11 ---------------------------------------------------------------------------------------------------- 402
Glyma.12G164100.1 ----------------NSTATGFLGFGG-NCYA------------------------------------------------------------------- 426
Glyma.16G000300.1 ----------------NSTATGFLGFGG-NCSA------------------------------------------------------------------- 426
Glyma.07G272800.1 ----------------NSSATNFIGFSG-NSSVGS---------------------------------------------------------------P 463
           AtARF1 ----------------PAKAATF-GHGG-NKSFGV----------------------------------------------------------------- 428
Glyma.06G164900.3 ----------------AEKSVVWPPTAVDDEKMDVST-------------SRKYGSESWMS---------------------------------MGRNEP 484
Glyma.04G200600.1 ----------------AEKSVVWPPAAVDDEKMDVST-------------SRRYGSESWMS---------------------------------MGRNEP 484
Glyma.05G200800.4 ----------------VEKSAVWPPVAD-DEKIDVST-------------SRRYGSDSWMS---------------------------------MGRHEL 501
Glyma.08G008100.3 ----------------AEKSGVWPPATD-DEKIDVST-------------SRRYGSDSWMS---------------------------------MGRHEP 496
           AtARF2 ----------------PENSVVWQSSAD-DDKVDVVS------------GSRRYGSENWMS---------------------------------SARHEP 506
Glyma.03G208800.1 -----------------------------------------------------------IRG------------------------------SRFGKENR 378
Glyma.07G202200.1 ----------------CYPVSNYPRIAATGNSIGIPQV------------------SSNVSS-------------------------------NGIGFSE 476
Glyma.13G174000.1 ----------------CYPGSNYPRIAATGNSIGISQV------------------SSNVSN-------------------------------NGIGFSE 481
Glyma.13G234200.1 ----------------CYPGSNCSGIPPTGNNIRMPHP------------------ASDFSC-------------------------------NGIGFSE 499
Glyma.15G078800.1 ----------------CYPGSNCSGIPPTGNNIRVPHP------------------ASDFSC-------------------------------NGIGFSE 491
           AtARF3 ----------------LQGQEIFPGFINTCSDGGAGAR------------------RGRFKG---------------------------------TEFGD 471
Glyma.12G071000.1 ----------------SHPNLGSAEV----RKVSSSEL------------------NSVHPF-------------------------------SYAGFVE 509
Glyma.11G154632.1 ----------------SHPNLGSAEV----RKITSSEL------------------SSVHPF-------------------------------SYAGFVE 508
Glyma.12G171000.1 ----------------SHPNLASTGV----RKIAAAEF------------------MRVHPS-------------------------------SYAGFTE 504
           AtARF4 ----------------ANPVLVSSRVKD--RFGEFVDA------------------TGVNP--------------------------------ACSGVMD 519
Glyma.08G100100.3 ---------------------------------------------------------SSIQF-------------QQP--------------QNFPNQTS 479
Glyma.05G143800.2 ---------------------------------------------------------SAIQF-------------QQQ--------------QNFPNRTS 476
Glyma.13G221400.1 ---------------------------------------------------------SLLPF-------------QQP--------------QNFPNRTA 474
Glyma.15G091000.1 ---------------------------------------------------------SLLQF-------------QQP--------------QNFPNRTA 477
           AtARF6 ---------------------------------------------------------SLLQF-------------QNS--------------PGFSMQSP 474
Glyma.02G281700.1 ---------------------------------------------------------SLLQF-------------QQT--------------SNVPSAHA 479
Glyma.14G032700.1 ---------------------------------------------------------SLLQF-------------QQT--------------SNVPSAHA 479
Glyma.11G204200.1 ---------------------------------------------------------QLMNF-------------QQP--------------YHYLQQSG 475
Glyma.18G046800.1 ---------------------------------------------------------QMMNF-------------QQP--------------YHYLQQSG 474
Glyma.02G239600.1 ---------------------------------------------------------QIMNF-------------QQP--------------FNYLQQSG 474
Glyma.14G208500.1 ---------------------------------------------------------QMMNF-------------QQP--------------FNYLQQSG 474
           AtARF8 ---------------------------------------------------------QFVQL-------------QEPHH-------------QYLQQSA 474
Glyma.09G072200.1 ------QQRAQQPQEQQQQSCQ-QTIMNNGAVA-SNQIPNQCVQQP--VSYSQLQQQQLIS-----GSIPPQQCFPSP-NKNTLLMTSLPQDSQFQQQID 625
Glyma.15G181000.1 ------QQRAQQPQQQQQQSCQ-QTIMSNGTVA-SNQIPNQCVQQP--VTYSQLQQQQLLS-----GSIPPQQSFQSP-NKNALLMTSLPQDSQFQQQID 626
Glyma.17G047100.1 ------QHLGQQPQKQQQSSQH-ATIMNNGVVA-SNQITNQFAQQP--MAYGQLQQQQLLS-----GGIQPQQGIQSA-TKNTFPLTSLPQDSQFQQQID 641
Glyma.13G112600.1 ------QQLGQQPQK-QQSSQH-ATIMNNGVVA-SNQITNQFSQQP--MAYGQLQQQQLLS-----GG------IQSA-TKNTLPLTSLPQDSQFQQQID 634
           AtARF7 NQLYNGQQAAQQHQSQQASTHHLQPQLVSGSMA-SSVITPPSSSLNQSFQQQQQQSKQLQQAHHHLGASTSQSSVIETSKSSSNLMSAPPQETQFSRQVE 711
          AtARF19 ---------------------Q-QGIYNNGTIAVANQVSCQSPNQPTGFSQSQLQQQSMLP----TGAKMTHQNINSMGNKGLSQMTSFAQEMQFQQQLE 600
Glyma.07G130400.1 ----------------PQGQVQ-SQLLHPQNMVQTNNILQQ--------QQPSIQNHQLHR--------------------------------SLSQNPS 569
Glyma.01G002100.1 ----------------PQGQVQ-AQLVHPQNIVQTNNILQQ--------QQPSSQNHQLHR--------------------------------SLSQNPS 554
Glyma.05G221300.1 ----------------PHDQAH-TQLLNPQRVVQTNNIHQQ--------QQSSIQKQQLLR--------------------------------SLSQNRA 570
Glyma.08G027800.1 ----------------PQNEAH-TQLLNSQRVVQTNNILQQ--------QQSSIQNHQLLR--------------------------------SLSQNPP 570
Glyma.14G217700.1 --------------------------------------------------------LKNLH----------------P--------------HSIPDQPN 496
Glyma.17G256500.1 --------------------------------------------------------LKNLH----------------P--------------QSIPDQPN 499
```

```
AtARF5              ---SEMVQ---PQN---KLTVNPS   511
Glyma.12G174100.1   ---ISL---SDLHLN-           460
Glyma.13G325200.1   ---ISL---SDLHLN-           442
Glyma.12G076200.1   ---KSL---SDLHLN-           460
Glyma.11G145500.1   ---KSL---SDLHLNN-          455
AtARF16             ---LSS---SDLHH---          465
AtARF10             ---SLL---SDLNLSSY          471
Glyma.10G210600.1   ---LSP---SDFPF---          464
Glyma.20G180000.1   ---LSP---SDFPF---          461
Glyma.10G053500.1   ---LSL---SDLHL---          471
Glyma.13G140600.1   ---LSL---SDLHL---          470
Glyma.19G181900.2   ---ISL---SNLHF---          475
Glyma.13G084700.1   ---LSF---SNFLNDN-          445
Glyma.14G166500.1   ---SSF---SNFLNDK-          442
Glyma.04G254200.1   ---SRF---CNYPTDM-          464
AtARF17             ---FNP---TGFIGGN-          461

Consensus           -----++++++++++++++++----+++++++++++++++++   366
```

```
                    920   940   960   980   1000

Glyma.01G103500.1   CP---SHLFQD---            462
Glyma.03G070500.1   CP---SHLFQD---            461
Glyma.07G134800.1   ---QLYQD---               446
Glyma.18G184500.1   ---SQLYHD---              446
AtARF21             N---NQMVTQIE---           446
AtARF20             N---NQMVTQIE---           432
AtARF15             N---NQMVTQIE---           446
AtARF23             ---                       223
AtARF12             N---NQMVTEME---           446
AtARF22             N---NQMVTQIE---           444
AtARF14             N---N-VVTPIE---           445
AtARF13             NS---DQMIQPRK---          443
AtARF9              ---                       422
Glyma.16G023600.1   VT---PNLFSD---            467
Glyma.07G054800.1   AT---PNLFSDP---           466
Glyma.03G258300.1   SK---PNLFMD---            441
AtARF18             ---LFQD---                411
AtARF11             ---LFQD---                406
Glyma.12G164100.1   SN---KSIYWS---            434
Glyma.16G000300.1   SN---KSIYWS---            434
Glyma.07G272800.1   SN---KSIYWS---            471
AtARF1              SI---GSAFWP---            436
Glyma.06G164900.3   TY---SDLLSGFG---          494
Glyma.04G200600.1   TY---SDLLSGFG---          494
Glyma.05G200800.4   TY---PDLLSGFG---          511
Glyma.08G008100.3   TY---PDLLSGFG---          506
AtARF2              TY---TDLLSGFG---          516
Glyma.03G208800.1   NQ---HPFLKQDPL---         389
Glyma.07G202200.1   SFRF---QKVLQGQE---        488
Glyma.13G174000.1   SFRF---QKVLQGQE---        493
Glyma.13G234200.1   SFRF---QKVLQGQE---        511
Glyma.15G078800.1   SFRF---QKVLQGQE---        503
AtARF3              SYGF---HKVLQGQE---        483
Glyma.12G071000.1   TNRF---PRVLQGQE---        521
Glyma.11G154632.1   TNRF---PRVLQGQE---        520
Glyma.12G171000.1   TNMF---PRVLQGQE---        516
AtARF4              LDRF---PRVLQGQE---        531
Glyma.08G100100.3   PLM---QTQLLQQSP---SQQAFPNSQENPHP---SPSQSQAQTQTHFQQHLQHQHSF---NNQN   532
Glyma.05G143800.2   ALM---QTQMLQQSQ---PQQAFPNSQENSHPSP---SPSQSQAQTQTHFQQHLQHQHSF---NTQN   531
Glyma.13G221400.1   ALM---QAQMLQQSQ---PQQIFGNTQENQHS---PQSQAHLQQHLQHQHSF---NSQH   521
Glyma.15G091000.1   ALM---QAQMLQKSQ---PQQIFGNNQENQHSP---QSQPQTQAHLQQHLQHQHSF---NSQ-   527
AtARF6              SLV---QPQMLQQQL---SQQQQQLSQQQQQQ---QLSQQQQQQLSQQQQQQLSQ---QQQQ   525
Glyma.02G281700.1   SEV---QRQLLPQSQ---LQNTLLHNFQENQVP---AQSQLLQQQLHRYHPY---SDQQ   526
Glyma.14G032700.1   SEV---QRQVLPQSQ---PQNTLLHNYQENQVP---AQSQLLQQQLHRYHPY---SDPR   526
Glyma.11G204200.1   NSN---SPLQLQQQQ---PIQQSVSS-NMLQPQ---THVLTENLSQHLLQKPHNN---QEVQ   524
Glyma.18G046800.1   NSN---SPLQLQQQQ---PIQQSVSS-NMLQPQ---AHVLTENLSQHLLQKPHNN---QEVQ   523
Glyma.02G239600.1   NPN---PPLQLQQPQ---AIQQSVSSNNILQPQ---AQVMAENLSQHLLQKSHNN---REDQ   524
Glyma.14G208500.1   NPN---LPLQLQQPQ---AVQQSVSSNNILQPQ---AQVLAENLSQH-LQKSHNN---REDQ   523
AtARF8              SHN---SDLMLQQQQ---QQQASR-HLMHAQ---TQIMSENLPQQNMRQEVSN---QPAG   521
Glyma.09G072200.1   QQ---ASLLQRQQLQQQQQTQLQSSPLQLLQQSLSQRAPQQPQMTQMSQQNSSDQQPQLQMLQKLQQQQQQQQQQQQQKPLSTSSPLLQSQL   715
Glyma.15G181000.1   QQ---ASLLQRQQ---QQQTQLQSSPLQLLQQSLSQRASQQPQMTQMCQQNSSEQQPQLQLLQKLQQQQQQQQQQQQQQLLSTSSPLLQSQL   712
Glyma.17G047100.1   QQ---ASLLQRQQ---QQQQTQLQQSPLQLLQQNLPQRALQQPPATQMLQQNPSEQQLHLQLLQKLQQQQQ---QQLLSTSTPLLQSQL   721
Glyma.13G112600.1   QQ---ASLLQRQQ---QQQQTQLQQSPLQLLQQNLPQRAPQQPPATQMLQQNPSEQQLHLQLLQKLQQQQQHQ---QRLLSTSTPLLLSQL   716
AtARF7              QQQPPGLNGQNQQTLLQQKA---HQAQAQQIFQQSLLEQ---PHIQFQLLQRLQQQQQQFLS---PQSQL   773
AtARF19             MHN---SSQLLRNQQ---EQSSLHSLQQNLSQN-PQQLQMQQQSSKPSPSQQLQLQLLQKLQQQQQQ---SIPPVSSSLQPQLSA   676
Glyma.07G130400.1   QQ---QTTIGQNQ---PQNLIQSPMPDHV-QQLQMS---DNQIQLQLLQKLQQQKQTLLAQ--QTALQQPTQLTQIQD   635
Glyma.01G002100.1   QQQ---QQTIIGQNQ---HQNLIQSPMPDHV-QQLQMS---DDQIQLQLLQKLQQQKQTLLAQ--QTALQHSTQLTQIQD   622
Glyma.05G221300.1   QKH---QQTTLGLNE---RQIVFQSPMPDHFNQQLQMS---DNQVRFQLLQKLQQQQQTLLAQ--QSALQQP-ALIQIQD   638
Glyma.08G027800.1   QKD---QQTTFGQNE---RQNVFQSPMPDHFNQQLQMS---DNQVRFQLLQKLQQQQQTLLAQ--QSALQQP-ALIQVQD   638
Glyma.14G217700.1   ALN---MQSLLKNDQ---PEKLHPLGKIDNHLSSGIVI---DKPKSESEVLPDHVIDYPSMEGCNIEKVAANP-VNQQG   565
Glyma.17G256500.1   ALN---MQSLLKNDQ---PEKFHPLAKIDNHLPSGIVI---DKPKLECEVLPDRVIDYPSMEGCNNEKVAVNP-VNQQG   568
AtARF5              ASN---TSGQEQN-L---SQSMSAPAKPENSTLSGCSS---GRVQHGLEQSMEQASQVTTSTVCNEEKV---   570
Glyma.12G174100.1   ---NKLQLG---              466
Glyma.13G325200.1   ---NKLQLG---              448
Glyma.12G076200.1   ---NKLQLG---              466
Glyma.11G145500.1   ---NKLQLG---              461
AtARF16             ---                       465
AtARF10             TGN---NKLHSP---           480
Glyma.10G210600.1   ---NKLPAD---              470
Glyma.20G180000.1   ---NKLPAD---              467
Glyma.10G053500.1   ---SKLQSG---              477
Glyma.13G140600.1   ---SKLQSG---              476
Glyma.19G181900.2   ---NKLQSG---              481
Glyma.13G084700.1   ---SYLYMGSG---            453
Glyma.14G166500.1   ---SYLYMGSG---            450
Glyma.04G254200.1   ---SRLCMGN---             471
AtARF17             ---PPQLFTN---             468

Consensus           ++++-------+++XXXX++----++++++++++++++++++++   370

                    1020   1040   1060   1080   1100
```

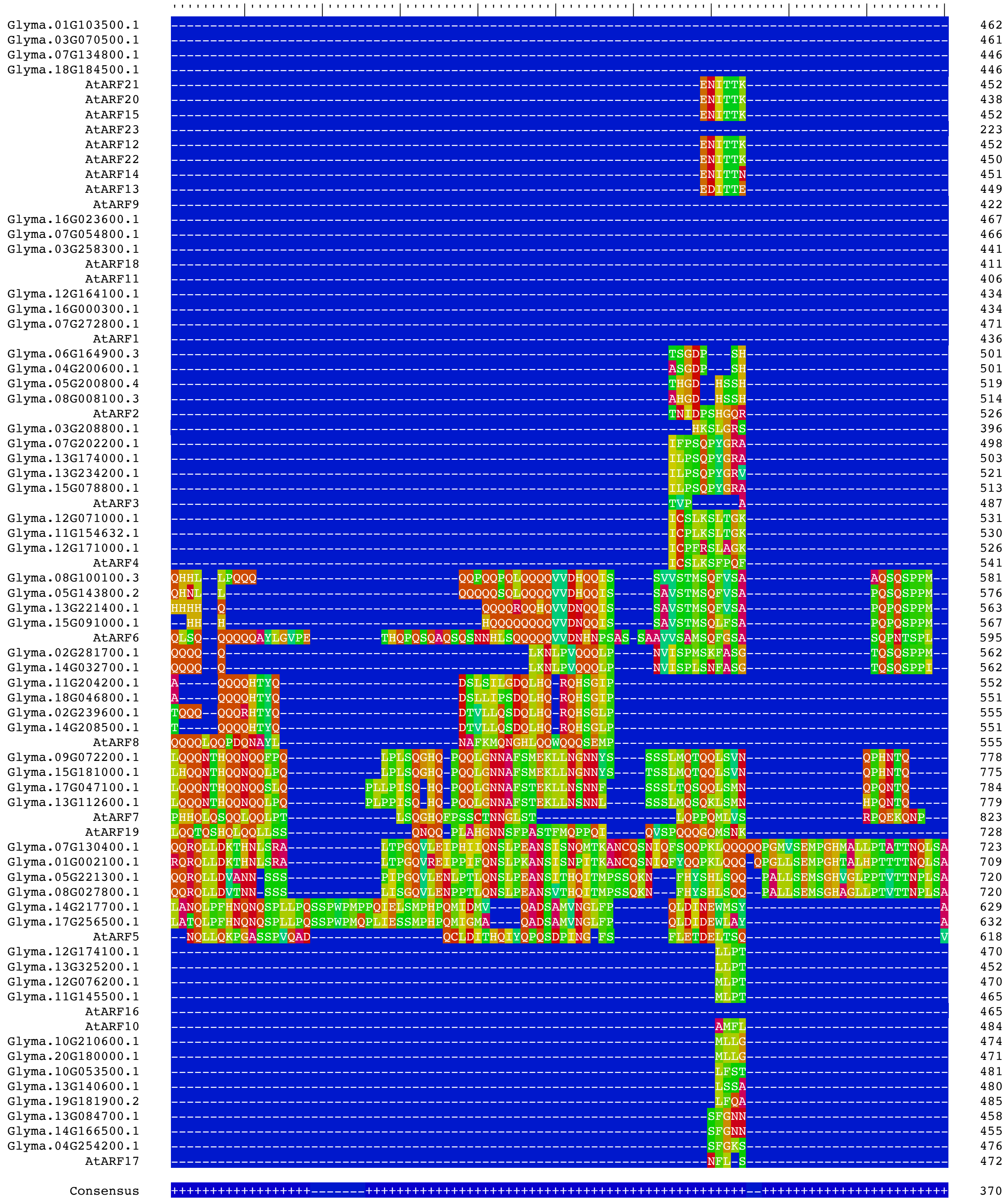

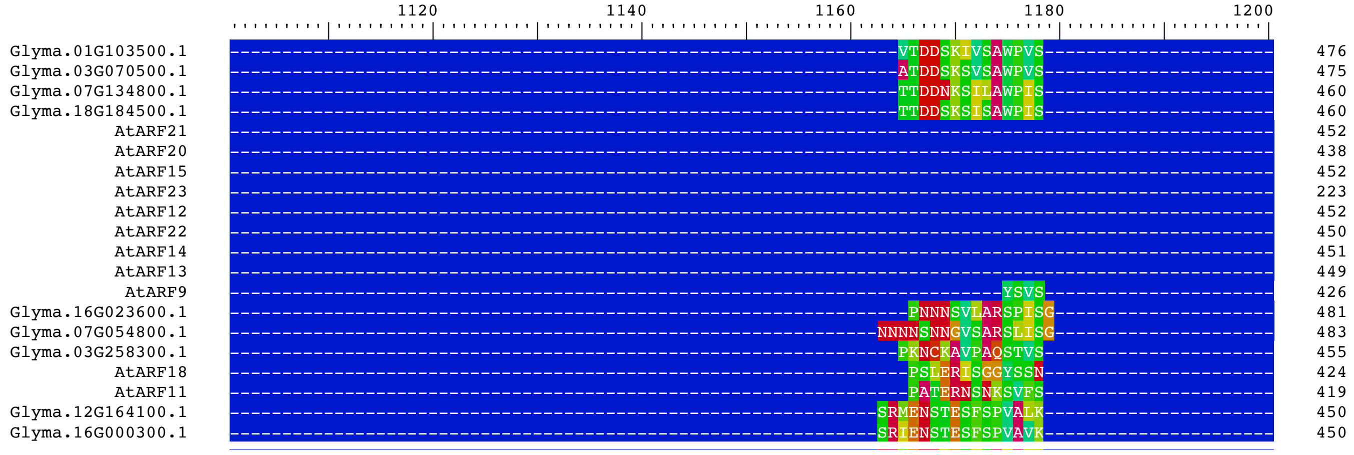

```
Glyma.07G272800.1  --------------------------------------------------------------NRMENNLESISAIALK----------------------  487
           AtARF1  --------------------------------------------------------------TNADSAAESFASAFNN----------------------  452
Glyma.06G164900.3  ---SSLKDQMSP-AYSARKQSLDHEGKLH--MPHPWPVMPSSLSLNILDSN------AKGPTHGGDTSFQARGNLRFSA---------------------  568
Glyma.04G200600.1  ---LSLKDQMSP-AYSARKQSLDHEGKLH--MPHPWPVMPSSLSLSILDSN------TKGPAHGGDTTYKARGNLRYSA---------------------  568
Glyma.05G200800.4  ---PSFVDQNGPVANVGRKHLLDCEGKHN--VLSPWSGVPSSLSLNLLDSN------TKGSAQGGDTTYQVRGNLRYSSA--------------------  588
Glyma.08G008100.3  ---PSFVDQNGPVANLSRKHLLDREGKHN--VLSPWPGVPSSLSLNLLDSN------LKGSAQGGDTAYQVRGNLRYSSA--------------------  583
           AtARF2  ---IPFYDHSSSPSMPAKRILSDSEGKFDY-LANQWQMIHSGLSLKLHES-------PKVPAA-TDASLQGRCNVKYS----------------------  592
Glyma.03G208800.1  ---MSLTHEDLS-----------------------------------------------------ITSSNLTSIGSESLGMPSTESR-------------  427
Glyma.07G202200.1  ---LSVDEACG----NSRCGLFD--GYQVMRSRNGWCSDMSNNSSNLHPPV---------PSGQVSSPSSVLMFQQAVNLVSNSDYN-------------  567
Glyma.13G174000.1  ---LSVDEACG----NGRFGLFD--GFQAMRSRNGWSSHVSNNSSHLHPPV---------PSGQVSSPSSVFMFQQAVNPVLNSDYN-------------  572
Glyma.13G234200.1  ---LSIEEARA----NGCYGLYD--GCQLLNTRNGWSAQMHDNAPYLHASV---------TPAQVSSPSSVLMFQQAVNPVSNCDYNK------------  591
Glyma.15G078800.1  ---LSIEEARA----NGRYGFYD--GCQLLNSRNGWSAQMHDNASHLHASV---------TPAQVSSPSSVLMFQQAVNPVSNCDYNI------------  583
           AtARF3  ---YSITDHRQ----Q-----------HGLSQRNIWCGPFQNFSTRI---L---------PPSVSSSPSSVLLTNSN-----------------------  534
Glyma.12G071000.1  ---VDLNLGAWG---MPNLSCTTFN-LHQATKPNF--------QPSLFPYG---------DIHQAGQ-ASLFCSKSTTFQRENV----------------  590
Glyma.11G154632.1  ---VDMNLGAWG---MPNLSCTTFN-LHQATKPSF--------QLSLFPYG---------DIHQASQ-ASLFCSKSTTFQRENV----------------  589
Glyma.12G171000.1  ---VDLNFGSWG---KPNVSYTNYN-LHQATKPNFHSFGPEVVQTAYFPYG---------DIHKAGQGSSMLCSNPTNFQREDI----------------  594
           AtARF4  ---AGFSPAAAP---NPF--------AYQANKSSYYPLALHGIRSTHVPYQ---------NPYNAGNQSSGPPSRAINFGEETRKF--------------  604
Glyma.08G100100.3  ---QAISSLGH------QQSFSDSN-GNP--TTTAIVSPLHSILDSF---------------PQ-DDTSHLLSLPRSTSWVPVQHSTAWPP--SKRVVVD  651
Glyma.05G143800.2  ---QAISSLGH------QQSFSDSN-GNP--VTTAVVSPLHSILGSF---------------PQ-DDTSHLLNLPRSTSWVPVQHSTAWPS--SKRVAVD  646
Glyma.13G221400.1  ---QVISSMCQ------QQNFSDSN-GN---TVTTIVSPLHSILGSF---------------PQ-DETSHLLNLPRTSSWIPVQNSSGWP---SKRVAVD  631
Glyma.15G091000.1  ---QAISSLC--------QNFSNSN-GN---SVTTIVSPLHSILGSF---------------PQ-DETSHLLNLPRTSSWIPIQNSSGWP---SKRVAVD  633
           AtARF6  ---QSMTSLCH------QQSFSDTN-GG-----NNPISPLHTLLSNF---------------SQ-DESSQLLHLTRTNSAM---TSSGWP---SKRPAVD  658
Glyma.02G281700.1  ---QALASHCQ------QQSFPEPM-RNH--ISGSDVSPIQSLLGSF---------------SQ-DGTSQLLNLSGSNSVMS--SAAILP----KQITAE  628
Glyma.14G032700.1  ---QALASHCQ------QQSFPELM-RNH--ISGSDVSSIHSLLGSF---------------SQ-DGTSQLLNLSGSNSVMS--SAAMLP----KQITTE  628
Glyma.11G204200.1  ------SSSYS------KPDFLDSS-MK----FPASVSPGQNMLSSL---------------CP-EGSGSLLNLSRSGQSLL------------------  601
Glyma.18G046800.1  ------SPSYS------KPDFLDSS-MK----FPASVSPGQNMLSSL---------------CP-EGSGSLLNLSRSSLSLL------------------  600
Glyma.02G239600.1  ------SPSYS------KPDFLDSS-MK----FPASVSPGQNILGSL---------------CP-EGSGNLLNLSRSGQSML------------------  604
Glyma.14G208500.1  ------SPSYS------KPDFLDSS-MK----FPASVSPGQNMLGSL---------------CP-EGSGNLLNLSRSSQSML------------------  600
           AtARF8  ------SPSFM------KSDFTDSS-NK----FATTASPA-------------------------SGDGNLLNFSITGQSVL------------------  595
Glyma.09G072200.1  ---KSLTNTRA------PSTLTD---GD---APSCSTSPSTNNCQIS--------------PNLMKRNQQVSATLGGPSVVEPTNH--LMQELHSKSEMQ  847
Glyma.15G181000.1  ---KSLTNTRA------PSTLTD---GD---APSCSTSPSTNNCQIS--------------PNLMKRNQHVPATLGGPSVLEPTNH--LMQGLHSKSEMQ  844
Glyma.17G047100.1  ---KSHTITRA------PSTLTD---GD---APSCSTSPSTNNCQVSPP-------------NLLKRNQQIPATLGGGLIVEPTSN--LIQELHSKPDTQ  854
Glyma.13G112600.1  ---KSLTITRA------PSTLTE---GD---APSCSTSPSTNNCQVTPP-------------NLLKRNQQLPATLRGSLIVEPTSN--LIQELHSKPDTQ  849
           AtARF7  ---PVGGGVKA------YSGITD--GGD---APSSSTSPSTNNCQISSS-------------GFLNRSQSGPAILIPDAAIDMSGN--LVQDLYSKSDMR  894
          AtARF19  ---NLVAAGRS------HSGHTD---GE---APSCSTSPSANNTGH-------DNVSPTNFLSRNQQQGQAASVSASDSVFERASN--PVQELYTKTESR  804
Glyma.07G130400.1  GGSSIVTGAGG----AGQSVITD----D---VPSRSTSPSTNNCTNALPQLINSRFPRSTMVGD-DMAHSAATILSSSALETSSSNANMLKDLQPKFEVK  811
Glyma.01G002100.1  AGSSILTGAGGAG-GAGQSVITD----E---VLSCSTSPSANNCTNALPQLINSRFQRSTLVGD-DMAQSAATILSSSALETTSSNANMLKDLQPKSEVK  800
Glyma.05G221300.1  SGGSILTGVG-------QSVITD----D---VPSCSTSPSTNNRASVLPPVVSSQIHRSTTIGD-DMAQSAVTISGASTLETMSSNANIVQ---PKYEVK  802
Glyma.08G027800.1  SGGSILTGAG-------QSVITD----D---VPSCSTSPSTNNRASALPPVVSSQIHRSTTIGD-DMAQSAVTISGPSTLETLSSNANMVKDVRPKYEVK  805
Glyma.14G217700.1  SS-QPFAGQNR-----PTGPLSDLQ-EHTSLQPQVVNPPLPSMNNEVWDHY----VKN---LKFLSQADQLTSICQPGLYGL------------------  697
Glyma.17G256500.1  SS-QPFAGQNR-----PTGPFSDLQ-EHNSLQPQVVNPPLPSMNNEVWDHY----VKN---FKFLSQADQLTSICQPGMYGL------------------  700
           AtARF5  SSFQSLAGSYK-----QPFILSSQD-SSAVVLPDSTNSPLF---HDVWDT-----------QLNGLKFDQFSPLMQQDLYA-------------------  679
Glyma.12G174100.1  -NVHQLN-------------------------LHTGICNGN-ITNHG------------------KSKESLSCLLTMGNSNKSLEKSDHV----------  515
Glyma.13G325200.1  -KVHQLN-------------------------LHAGISNAK-------------------------SKESLSSLLSMGNSNMTLEKSDHV----------  491
Glyma.12G076200.1  -NIHQLG-------------------------VYNEISNGNMMTNHD------------------KSKESLSCFLTMGKSTKSLEKSDDV----------  516
Glyma.11G145500.1  -NIHQLGG------------------------VYTGISSGNMMTKHD------------------KSKESLSCFLTMGKSSKSLEKSDDV----------  512
          AtARF16  ---YYLNRPPPPP-----------------PPSSLQLSPSLGLRNID------------------TKNEKGFCFLTMGTTPCNDTKSKKS----------  517
          AtARF10  -SSFNPRH------------------------HHYQARDSE-------------------------NSNNISCSLTMGNPAMVQDKKKSVG---------  525
Glyma.10G210600.1  -GFSRLDHAAAQP-----------------IRPPCGTYKNNTTTK---------------------ANVGISCLLTVGNPGQNFKESNET----------  525
Glyma.20G180000.1  -GFSRLDHAAAQP-----------------IMPHCGTFKNNTTTK---------------------ANVDISCLLTVGNPGQNFKESNET----------  522
Glyma.10G053500.1  -GFPSLDHAAT--------------------PMRV--SNSITLQKPN-------------------LSENVSCLLTMANSTQSSKKLDVG----------  529
Glyma.13G140600.1  -GFPPLDHAAT--------------------PMKV-----------------------------------------------------------------  494
Glyma.19G181900.2  -GFPPLDHTASP-------------------VLRVSSNNAATMQKVG-------------------TGDNVSCLLSMSTATQPSKKVDDV----------  536
Glyma.13G084700.1  -PVQSLGTVTT--------------------ELNMSSSQSDDL-----------------------SPHSQSSFHSFGTEFTGTRNCDTK----------  504
Glyma.14G166500.1  -PVQSLGTVTT--------------------ELNMSSSQSDDL-----------------------SPHSQSSFHSFAREFAGTRNCDTK----------  501
Glyma.04G254200.1  -TFPWLKTLET--------------------EVNVGSSQSDEL-----------------------SPDSRGSLQSCGTDLVG---NSRK----------  519
          AtARF17  -PLPDLGKVSTE-------------------MMNFGSPPSDNL-----------------------SPNSNTTNLSSGNDLVGNR-GPLS----------  518

        Consensus  ++++++++++++++++++++++++++++++++++++++++++++++++++++++++++++++++++++++++++++++++++++++++++++++++++++  370
```

```
                                 1220                1240                1260                1280                1300
Glyma.01G103500.1  ---------------------------------------------------------------------KPHSSKLNNDHVLDQVDKE-SKVETATSYRL  506
Glyma.03G070500.1  ---------------------------------------------------------------------KPHSSRLNNDHVLDQVDKE-SKVETATSYRL  505
Glyma.07G134800.1  ---------------------------------------------------------------------KPHSERLNNDHFLDQVDKNINKVEAATSYRL  491
Glyma.18G184500.1  ---------------------------------------------------------------------KPHSERLNNDHFLDQVDKEINKVEAATSYRL  491
          AtARF21  ---------------------------------------------------------------------------------------------AGTNFRL  459
          AtARF20  ---------------------------------------------------------------------------------------------AVTNFRL  445
          AtARF15  ---------------------------------------------------------------------------------------------AGTNFRL  459
          AtARF23  ----------------------------------------------------------------------------------------------------  223
          AtARF12  ---------------------------------------------------------------------------------------------TGTNFRL  459
          AtARF22  ---------------------------------------------------------------------------------------------TGTNFRL  457
          AtARF14  ---------------------------------------------------------------------------------------------AVASFRL  458
          AtARF13  ---------------------------------------------------------------------------------------------ATTSCLL  456
           AtARF9  ---------------------------------------------------------------------NVAKDSTLNDQMVSPVEQK--KPETTANYRL  455
Glyma.16G023600.1  --------------------------------------------------------------------YLNVPSRSSDGPTCEHVEDG-KKIENSLDCWL  512
Glyma.07G054800.1  --------------------------------------------------------------------YPNVPSRSSDGPTCDGVEDG-KKTENSLDCWL  514
Glyma.03G258300.1  -------------------------------------------------------------------------SRPKDDLAHDPMECAKRSSQNPMNCWI  482
          AtARF18  ----------------------------------------------------------------------------------NSFKPETPPPPTNCSYRL  442
          AtARF11  ----------------------------------------------------------------------------------SGLQCKITEAPVTSSCRL  437
Glyma.12G164100.1  -------------------------------------------------------------------------------------EFGEKRQGTANGCRL  465
Glyma.16G000300.1  -------------------------------------------------------------------------------------EFGEKRQGTANGCRL  465
Glyma.07G272800.1  -------------------------------------------------------------------------------------EAGEKRQGTGNGCRL  502
           AtARF1  -------------------------------------------------------------------------------------ESTEKKQTNGNVCRL  467
Glyma.06G164900.3  -------------------FGEYPALHG--HKVEDSHGNLMPPPPA------------------PQTQYQSPCSRELMSKHVSAKTCEAVKPKDG-DCKL  628
Glyma.04G200600.1  -------------------FGEYPALHG--HKVEHSHGNLMPPPPA------------------LLTQYQSPCSRELMSKQVSAKTCEAVKPKDG-DCKL  628
Glyma.05G200800.4  -------------------FGEYPMLHG--HKVEHSHGNFLMPPPPSTP-------------------YESPRSRELLPKPISGKPCEVSKPKDS-DCKL  647
Glyma.08G008100.3  -------------------FGEYPVLHG--HKVEHSHRSFLMPPPPSTQ-------------------YESPRSRELLSKPISGKPCEVSKLKDS-DCKL  642
           AtARF2  ---------------------EYPVLNG--LSTENAGGNWPIRPRALNYYE---------E---VVNAQAQAQAREQVTKQPFTIQEETAKSREG-NCRL  656
Glyma.03G208800.1  ----------------------------------------------------------------------------------DENDAPFGQPGSSSTFKL  445
Glyma.07G202200.1  -------------NKISQVMGGKFHQRV--SYASDVKGGKFVSTPYEPLL--CGLAKEGTNSFGLSNFHNQLDSSRSHDSISVLRANQELVPSCKSSCRV  650
Glyma.13G174000.1  -------------NQIGQVMGDKVHQRV--SYASEVKGGKFVSTPYEPLL--RGLSQEGTNSFGLSNFHNQLDSSRSHDSVSVLRASQELVPSCKSRCRV  655
Glyma.13G234200.1  -------------NKRDKEMEGRVHYQG--LYTSEVKGRTFASSPFD-DPILSTLAPEGASSLGMFDVHNQLGSSRPHESVSALRSSQELVSSCKSSCRL  675
Glyma.15G078800.1  -------------NKCNKEMEGRIHYQG--LYTSEMKGRTLASSPSD-DLIVSRRAPEGASSIGMFDVHNQLGSSQLHESVSALRSSQKLVSSCKSSCRL  667
           AtARF3  ----------------------------------------------------------------------------------SPNGRLEDHHGGSGRCRL  552
Glyma.12G071000.1  ------------------PFNKPSTQAG--IIVNEVG---RSDLPNEHKL--Q---------------------DNISSAANMGVSNDNNVQGKVNACKL  644
Glyma.11G154632.1  ------------------PFNKPSTQAG--IIVNEVG---RSDLPNDHKL--QG--------------------NNISAAGNMGVSIDNNVQGKVNACKL  644
Glyma.12G171000.1  ------------------PFNTPSIQSG--ITI-----------PNEQKL--Q-DNISGAASLGAN----------------MRIPNDDNFKGKVNACKL  644
           AtARF4  ----------------------DAQNEG--GLPNNV----TADLP-------------------------------FKIDMMGKQKGSELNMNASSGCKL  645
Glyma.08G100100.3  PLLS-SGA-SQCVLPQVEQLG---------QPHSTMAQNGIALPAF---------------------------------PGRECTIE--GS-NDPQNHLL  704
Glyma.05G143800.2  PLFS-SGA-SQCVLPQVEQLG---------QPQSTMAQNGIALPPF---------------------------------PGRECTIE--GS-NDPQNHLL  699
Glyma.13G221400.1  PLLS-SGA-SQCVLPQVEQLG---------QPQSTMSQNAISLPPF---------------------------------PGRECSIDQEGS-NDPQNHLL  686
Glyma.15G091000.1  PLLS-SGA-SHCVLPQVDQLG---------QPHSTMSLNAITLPPF---------------------------------PGRESSIDQEGS-NDPQNHLL  688
```

```
           AtARF6  SSFQHSGAGNNNTQSVLEQLGQSHT--------SNVPPNAVSLPPFPG--------------------------------GRECSIEQEGSASDPHSHLL  718
Glyma.02G281700.1  PPQLPSAA-SQCILPQVENLG---------TSQSNVSE-LAALPPF---------------------------------PGREHSAYHGAA--DPQSNLL  682
Glyma.14G032700.1  PPQLPSAA-PQCVLPQVENLG---------TSQSNVSE-LAALPPF---------------------------------AGREHSAYHAAA--DPQSNLL  682
Glyma.11G204200.1  ----------------TEQLPQQQWTQKYAPVQVNAYGSTVSHPQY---------------------------------SGKDSVMVLPHCNSDAQNSTL  652
Glyma.18G046800.1  ----------------TEQLPQQQWTQKYAPVQVNTYGGTVSHAQY---------------------------------SGKDSAMVLPHCNSDAQNSTL  651
Glyma.02G239600.1  ----------------TEQLPQQSWAPKFTPLQVNAFGNSMQHVQY---------------------------------SGKDTAMVPPHCNSDTQNPIL  655
Glyma.14G208500.1  ----------------TEQLPQQSWAPKFTPLQINAFGNSMQHVQY---------------------------------SGKDTAMVPPHCNPDSQNPIL  651
           AtARF8  ----------------PEQLTTEGWSPK----ASNTFSEPLSLPQA---------------------------------YPGKSLALEP---GNPQNPSL  639
Glyma.09G072200.1  IKHELPSVR------GTDQLKFKGTVAD--QMEA-SSGTSYCIDPNNIH-----------------------Q-----NFPLPNFCMDGDVQSHPRNNLP  910
Glyma.15G181000.1  IKHELPSVR------GTDQLKFKGTVAD--QMEA-SSGTSYCIDPNNIH-----------------------Q-----NFPLPNFCMDGDVQSNPRNNLP  907
Glyma.17G047100.1  IKQELLNVK------GPDQLKYKGTITD--PLEASSSGTSYCLDPGNVQ-----------------------Q-----NLPLSNFCMERDVQSHPRNSLP  918
Glyma.13G112600.1  IKQEFLNVK------GPDQLKYKGTITD--QLEA-SSGTSYCLDPPGNV----------------------QQ-----NLPLSNFCMEGDVQSHPRNSLP  913
           AtARF7  LKQELVGQQ-----------KSKASLTDH-QLEASASGTSYGLDGGENN---------------------RQQ---------NFLAPTFGLDGDSRNSLL  952
          AtARF19  ISQGMMNMKS-----AGEHFRFKSAVTD--QIDVSTAGTTYCPDVVGPV--------------------QQQQTFPLPSFGFDGDCQSH----HPRNNLA  873
Glyma.07G130400.1  PSLNISKIQNQGHFAPHTYLNGNAAHTD--CLDTSSSTTSVCLSQSDAHM-----------NQNSNPLSYNRQ-----SMLFRDNNQDGEVQADARSNIP  893
Glyma.01G002100.1  PSLNISKIQNQGHFAPQTYLNGNAAHTD--CLDTSSSTTSVCLSQSDAHM-----------HQNNNPLSYNPQ-----SLLFRDNNQDGEVQADARSNIP  882
Glyma.05G221300.1  ASLNISKNQNQGNVAPQTYLNG-VVQTD--YLDSSSSTTSLYHFRSDTHM-----------HQNTNPFSYNPQ-----LVYCRDNSQNVEVQADARNNVL  883
Glyma.08G027800.1  PSSNISKNQNHGNVARQMYLNG-VVQTD--YLDSSSSTTSLYHFQSDTHM-----------HQNNNPFSYNPQ-----LIYCRDNSQNVEVQADARSNVP  886
Glyma.14G217700.1  -----------------NGIPSSNNLRD--LSAESNNQSEICVNVDASN-------------------------------SVGTTVVDPSTSSTILDEFC  747
Glyma.17G256500.1  -----------------NGVPSSNNLRD--LSAESNNQSEICVNVDASN-------------------------------SVGTTMVDPSTSSTILDEFC  750
           AtARF5  -------------------------------------SQNICMSNS-----------------------------------TTSNILDPPLSNTVLDDFC  707
Glyma.12G174100.1  ---------------------------------------------------------------------------------------------KRHQFLL  522
Glyma.13G325200.1  ---------------------------------------------------------------------------------------------KRHHFLL  498
Glyma.12G076200.1  ---------------------------------------------------------------------------------------------KKHQFLL  523
Glyma.11G145500.1  ---------------------------------------------------------------------------------------------KKHQFLL  519
          AtARF16  ------------------------------------------------------------------------------------------------HIVL  521
          AtARF10  -------------------------------------------------------------------------------------------SVKTHQFVL  534
Glyma.10G210600.1  ---------------------------------------------------------------------------------------------KAPHILL  532
Glyma.20G180000.1  ---------------------------------------------------------------------------------------------KAPHILL  529
Glyma.10G053500.1  ---------------------------------------------------------------------------------------------KTPSLVL  536
Glyma.13G140600.1  ----------------------------------------------------------------------------------------------------  494
Glyma.19G181900.2  ---------------------------------------------------------------------------------------------KAPQLVL  543
Glyma.13G084700.1  --------------------------------------------------------------------------------------------VGPGSILL  512
Glyma.14G166500.1  --------------------------------------------------------------------------------------------VASGSILL  509
Glyma.04G254200.1  --------------------------------------------------------------------------------------------LGSVSFQL  527
          AtARF17  --------------------------------------------------------------------------------------------KKVNSIQL  526

        Consensus  ++++++++++++++++++++++++++++++++++++++++++++++++++--+++++++++++++++++++++++++++++++++++++++++XX+XXXX  376
```

```
                                      1320                1340                1360                1380                1400

Glyma.01G103500.1  FGIDLIDPSRNSPSVEKASAQA-VNVPK-------VTTEG-------------CTSTL-SRTDA--------------GHKSDVSMASS--MERKQEQ--  566
Glyma.03G070500.1  FGIDLIDHSRNSPSVEKASAQA-GNAPK-------VTTEG-------------CTSTL-TRTDA--------------GHLSDVPMASS--KERKQEQ--  565
Glyma.07G134800.1  FGIDLIDHARN------NSLSV-ENASG-------VASE--------------------CKTDV--------------NHESDLS-KAS--KEWNQEQ--  538
Glyma.18G184500.1  FGIDLIDHARN------NSLSA-ENASG-------ITSE--------------------CKIDV--------------NHVSDIS-KAS--KEWNQEQ--  538
          AtARF21  FGVTLDTPPMIK-----DPIKQ-------------IG--------------------------------------------SDISKLTER-KKFGQSQ--  494
          AtARF20  FGVSLAIPLVIK-----DPIEE-------------IG--------------------------------------------SDISKLTEG-KKFGQSQ--  480
          AtARF15  FGVSLATPPVIK-----DPIEQ-------------IG--------------------------------------------SDISKLTEG-KKFGQSQ--  494
          AtARF23  ----------------------------------------------------------------------------------------------------  223
          AtARF12  FGVTLDTPPVIK-----DPIEE-------------IG--------------------------------------------SEISKLTEG-KKFGLSQ--  494
          AtARF22  FGVSLVTPSVIK-----DPIEE-------------IG--------------------------------------------SEISKLTEG-KKFGQSQ--  492
          AtARF14  FGVSLATPSVIK-----DPVEQ-------------IG--------------------------------------------LEISRLTQE-KKFGQSQ--  493
          AtARF13  FGVDLTKVSKSK-----DSICP-------------IESCKK----------------------------------------SEISKLKN-----------  487
           AtARF9  FGIDLMSSSLAVPEEKTAPMRP-INISK-------PTMD----------------------SHS--------------DPKSEIS-KVS--EEKKQEP--  506
Glyma.16G023600.1  FGVNLTNNCSNV-IITTPSERE-LRGPI----SSSVAPSGPK---------ESIPAAA-CETER--------------VQTPNYS-LSN--KGQKQII--  577
Glyma.07G054800.1  FGVNLTNNCSNV--ITTPSERE-QRGPSS-----SVVLSGPK---------ESIPAAA-CETER--------------VQTPNYS-LSN--KGQKQII--  577
Glyma.03G258300.1  FGVNLTNNITKN--VTLPDKEQ-LGCPA-------IIPSGPK---------DSIPVAA-CETEA--------------GQNPYYS-LSN--KEHKQNI--  543
          AtARF18  FGFDLTSNSPA-------------PIPQD-----KQPMD-----------------------------------------------TCG--AAKCQEP--  473
          AtARF11  FGFDLTSKP-----------AS-ATIPHD-----KQLIS------------------------------------------VDSNISDS--TTKCQDP--  474
Glyma.12G164100.1  FRIQLHDNSNSN----EESLPM-VTLSG------RMGDDGPL---------PSLDAESDQHSEP--------------SNVNRSDIPSV--SCDAEKS--  527
Glyma.16G000300.1  FGIQLHDNSNSN----EESLPM-VSLSG------RVGDDGLL---------PSLDAESDQHSEP--------------SNVNRSDFPSV--SCDAEKS--  527
Glyma.07G272800.1  FGIQLLENSNA-----EGNLQT-VTLSG------RVGDDRSV---------PSLDAESDQHSEP--------------SNANRSDIPSV--SCDAEKS--  563
           AtARF1  FGFELVENVNV-----DECFSA-ASVSG------AVAVDQPV---------PSNEFDSGQQSEP--------------LNINQSDIPSG--SGDPEKS--  528
Glyma.06G164900.3  FGFSLISGPIVP----EPSLSQ-RNVSE-PAGQMHLTAHQQR---------TSENDEKSDHSKG--------------SRPVDDLVVDD--HDRPLQT--  695
Glyma.04G200600.1  FGFSLISGPTLP----EPSLSQ-RNVSE-AADQMHLTAHQQR---------TSENDEKLDHSKG--------------SRPVDDIVVDD--QDRPLRT--  695
Glyma.05G200800.4  FGISLLSSPIAP----EPSVSQ-RNVPSEPVGHMHTTSHQQR---------AFDNDQKSEHSRGG-------------SKPADGLLIDD--HEKVLQT--  716
Glyma.08G008100.3  FGISLLSSRPI---ASEPSLSQ-RNVTSESVGHMHTASHHQR---------AIENDQKSEHSRG--------------SKPADGLLIDD--HEKVLQT--  711
           AtARF2  FGIPLTNNMNGT----DSTMSQ-RNNLNDAAGLTQIASPKVQ---------DLS-----DQSKG--------------SKSTND--HRE--QGRPFQT--  717
Glyma.03G208800.1  FGVNLIDSSPEIPS---------------------------------------------------------------------VNFVDLNKTSSLPSSP-  475
Glyma.07G202200.1  FGFSLTEVAPIA----------------------------------------------------------------------------------------  662
Glyma.13G174000.1  FGFSLTEGAPVA----------------------------------------------------------------------------------------  667
Glyma.13G234200.1  FGFSLTEDTHLA----------------------------------------------------------------------------------------  687
Glyma.15G078800.1  FGFSLTEDTHVA----------------------------------------------------------------------------------------  679
           AtARF3  FGFPLTD---------------------------------------------------------------------------------------------  559
Glyma.12G071000.1  FGFSLSGETTAQ----------------------------------------------------------------------------------------  656
Glyma.11G154632.1  FGFSLSGETTTQ----------------------------------------------------------------------------------------  656
Glyma.12G171000.1  FGFPLSRETTAQ----------------------------------------------------------------------------------------  656
           AtARF4  FGFSLPVETPAS----------------------------------------------------------------------------------------  657
Glyma.08G100100.3  FGVNIEPSSLL---------MH-NGMSSLKGVSSNSDS-----------------PTIPFQSSNYLN-TTGPDSSLNPGM---THNIGE--TGFLQ----  767
Glyma.05G143800.2  FGVNIEPSSLL---------MH-NGMSSLKGVSSNSDS-----------------PTIPFQSSNYLN-TTVPDSSLNPGM---THNIGE--SGFLQ----  762
Glyma.13G221400.1  FGVNIEPSSLL---------MP-NGMSSLKGVCGNNGS-----------------STLPYQSSNYLNTTTRTDSSLNHGM---TPNIGD--SGFLQ----  750
Glyma.15G091000.1  FGVNIDPSSLL---------MP-NGMSSLKGVSGNNNS-----------------STLPYQSSNYLNTTTGTDSSLNHGM---TPNIGD--SGFLH----  752
           AtARF6  FGVNIDSSSLL---------MP-NGMSNLRSIGIEG----------------GDSTTLPFTSSNFNN-----DFSGNLAMTTPSSCIDE--SGFLQ----  781
Glyma.02G281700.1  FGINIDPSSLM---------LQ-SGMSNLRNIGKVNDS-----------------LSLPFSTSNCGG-ATGTDFPLSSNMTT-SSCVDE--SGFLQ----  747
Glyma.14G032700.1  FGINIDPSSLM---------LQ-NGMSNLRNIGNVNNS-----------------LSLPFSASNCGG-ASGTDFPLSSNMTT-SSCVDE--SGFLQ----  747
Glyma.11G204200.1  FGVNIDSSGLL---------LP-TTVPGYTTSSADTNS-----------------STMPLAESGF--------------QGSLYGCMQD-SSELLQ----  706
Glyma.18G046800.1  FGVNIDSSGLL----------P-ITVPGYTTSSADTNS-----------------STMPLADSGF--------------QGSLYGCM-D-SSELLQ----  703
Glyma.02G239600.1  FGVNIDSSGLL---------LP-TTVPRYTTASADSDA-----------------SAMPLGESGF--------------QSPLYPCGQD-SSELVQ----  709
Glyma.14G208500.1  FGVNIDSSGLL---------LP-TTVPRYTTASAEIDA-----------------SAMPIGESGF--------------QSPLYPCVQD-SSELVQ----  705
           AtARF8  FGVDPDS----------GLFLP-STVPRFASSSGDAEA-----------------SPMSLTDSGF--------------QNSLYSCMQDTTHELLH----  693
Glyma.09G072200.1  FASNLDGLTPD------TLLSR--GYDSQKDFQNLLSNYGGA-----PRDIETELSTAALSPQPFG----VPDMPFKPGCSSDIA-IND--PGVL-----  985
Glyma.15G181000.1  FASNLDGLTPD------TFLSR--GYDSQKDLQNLLSNYGGA-----PRDIETELSTAALSPQPFG----VPGIPFKPGCSSDIA-IND--PGVL-----  982
Glyma.17G047100.1  FDSNLDGLTPD------TMLLR--GYDSQKDLQNLLSNYASA-----PRDIETELSTADISSQSFG----VPNMPFKPGCSSDVG-IND--TGVLNN---  995
Glyma.13G112600.1  FDSNLDGLTPD------TMLLT--GYDSQKDLQNLLSNYGGA-----PREIETELSTADISSQSFG----VPNMPFKPGCSSDVG-IND--TGVLNN---  990
           AtARF7  GGANVDNGFVP-----DTLLSR--GYDSQKDLQNMLSNYGGV-----TNDIGTEMSTSAVRTQSFG----VPNVP---AISNDLA-VND--AGVL----- 1025
          AtARF19  FPGNLEAVTSD------PLYS-------QKDFQNLVPNYGNT-----PRDIETELSSAAISSQSFG----IPSIPFKPGCSNEVGGIND--SGIMN----  945
Glyma.07G130400.1  YANNIDSQIGMPLN-PDSLLTK-GTLRLGKYLSNNFSSEGMLGNYENNRDAQQELSSSMV-SQTFG----VPDMAF---NSIDST-IDD--SNFL-----  975
Glyma.01G002100.1  YANNIDSQMGMPLN-PDSLSTK-GTLRLGKDLSNNFSSEGMLGNYEINRDAQQEPSSSMV-SQTFG----VPDMAF---NSIDST-IDD--SNFL-----  964
Glyma.05G221300.1  IGNNVNGQMGMPSN-LDSLLTK-GTVGLGKELSNKFASGGLLRDLENNKGVPPEISSSMV-SQTFE----VPDMSF---NSIDST-IDG--SSFL-----  965
Glyma.08G027800.1  FVNDINGQMGMPSN-LDSLLTN-GTVGLGKDLSNNFSSGGLLGDLENNKGVQPELSSSMV-SQTFE----VPDMSF---NSIDST-IDG--SSFL-----  968
Glyma.14G217700.1  TMKDREFQNPQ------DCMVG--NLSSSQDVQSQIT------------------SASLTESHAFPL-RDIPDNS--GGTSSSHVDFDE--SSFLQN---  813
Glyma.17G256500.1  TMKDGEFQNPQ------DCMVG--NLSSSQDVQSQIT------------------SASLAESHAYPL-RDIPDNS--GGTSSSHVDFDE--SSFLQN---  816
           AtARF5  AIKDTDFQNHP-----SGCLVGNNNTSFAQDVQSQIT------------------SASFADSQAFSR-QDFPDNSGGTGTSSSNVDFDD--CSLRQNS--  779
```

```
Glyma.12G174100.1 FGQPILTEQQISRS-SSDVLSQ---------NFTVTDDENK--------------------------------------EKKEKGFLSDSQSSVSP---- 570
Glyma.13G325200.1 FGQPILTEQQISRS-SSDV---------------ATDDENK--------------------------------------EKKKKGFLSDSQSSVSP---- 540
Glyma.12G076200.1 FGQPILTEQQISSC-SGDVLSH---------RKRSVSDD----------------------------------------KDKAKCLMDDSQSTLSQQ--- 570
Glyma.11G145500.1 FGQPILTEQQISSC-SRDVLSR---------GKRSLGDE----------------------------------------KDKAKCVLDDSQSTLSQQ--- 566
          AtARF16 FGKLILPEEQLSEKGSTDTANI---------EKTQISSGG----------------------------------------SNQNGVAGREFSSSDE---- 568
          AtARF10 FGQPILTEQQVMNR------------------KRFLEEEAE--------------------------------------AEEEKGLVARGLTW------- 571
Glyma.10G210600.1 FGKLIQTEQKSSN----------------------------------------------------------------------TSSANTNGNSVSEG--- 559
Glyma.20G180000.1 FGKLIHTEQKSSNT---------------------------------------------------------------------TSSASTNGNSVSEG--- 557
Glyma.10G053500.1 FGQKILTEQQISPSSSGDTLSP--------VLTRNCSSDGN--------------------------------------VDKVTNFSDGSGSALHQEGLR 590
Glyma.13G140600.1 ---------------------------------------------------------------------------------------------------- 494
Glyma.19G181900.2 FGQTILTEQQISLNTSAKT-----------DPTRNNSFDGN--------------------------------------ADKMCKFSDGFGYALHPQG-- 592
Glyma.13G084700.1 FGKII---QPAESD------------------------------------------------------------------LHDADCMERDGSR------- 536
Glyma.14G166500.1 FGKII---QPAESD------------------------------------------------------------------LHDGDCMERDGSR------- 533
Glyma.04G254200.1 FGAVIQTEQPVENG------------------------------------------------------------------LQGTGCT------------- 548
          AtARF17 FGKIITVEEHSESG------------------------------------------------------------------PAESGLCEEDGS-------- 552

        Consensus XXXXXXX+X+++++++++++++-++++++++++++++++++++++++++++++++++++++++++++-++++++++++++++++++++++++++++++-- 384
```

```
                                       1420                1440                1460                1480                1500

Glyma.01G103500.1 --LQVSPKDTQ-----------------SKQIC----RSRTKVQM-------QGVAVGRAVDLTMLDGYGQLINELEDMFNIKGQLQHRN---K-WEIVF 632
Glyma.03G070500.1 --QQVSPKETQ-----------------SKQIC----RSRTKVQM-------QGVAVGRAVDLTMLDGYDQLINELEEMFDIKGQLQHRN---K-WEIVF 631
Glyma.07G134800.1 --LLVSPKETQ-----------------SKQVCS---RSCTKVQM-------QGVAVGRAVDLTTLDGYDQLVDELEKMFDIKGQLQHRN---K-WETVF 605
Glyma.18G184500.1 --LQLSPKETQ-----------------SKQVCS---RSCTKVQM-------QGVAVGRAVDLTTLDGYDQLVDELEKMFDIKGQLQLRN---K-WEIVF 605
          AtARF21 --TLRSPIEIQ-------------------SKQFSSSRTCTKVQM-------QGVTIGRAVDLSVLNGYDQLILELEKLFDIKGQLQTRN----QWKIAF 562
          AtARF20 --TLRSPIEIQ-------------------SKQFGSTRTCTKVQM-------QGVTIGRAVDLSVLNGYDQLILELEKLFDLKGQLQTRN----QWKIAF 548
          AtARF15 --TLRSPTKIQ-------------------SKQFSSTRTCTKVQM-------QGVTIGRAVDLSVLNGYDQLILELEKLFDLKGQLQTRN----QWKIIF 562
          AtARF23 ---------------------------------------------------------------------------------------------------- 223
          AtARF12 --TLRSPTEIQ-------------------NKQFSSSRTCTKVQM-------QGVTIGRAVDLSVLNGYDQLILELEKLFDIKGQLQTRN----QWEIAF 562
          AtARF22 --TLRSPTEIQ-------------------SKQFSSTRTCTKVQM-------QGVTIERAVDLSVLNGYDQLILELEELFDLKGQLQTRN----QWEIAF 560
          AtARF14 --ILRSPTEIQ-------------------SKQFSSTRTCTKVQM-------QGVTIGRAVDLSVLNGYDQLILELEKLFDLKGQLQARN----QWEIAF 561
          AtARF13 ---------------------------------QKATTSCLKIKSLTKPNL*------------------------------------------------ 506
           AtARF9 --AEGSPKEVQ-----------------SKQSSS--TRSRTKVQM-------QGVPVGRAVDLNALKGYNELIDDIEKLFDIKGELRSRN---Q-WEIVF 574
Glyma.16G023600.1 --SEASPNEWQ-----------------NKQATVPSMRTRTKVQM-------QGVAVGRAFDLTTLSGYDDLIEELEKLFEIRGELHSQD---K-WAVTF 647
Glyma.07G054800.1 --SEASPNQWQ-----------------NKQATVLSMRTRTKVQM-------QGVAVGRAFDLTTLSGYDDLIDELEKLFEIRGELRSQD---K-WAVTF 647
Glyma.03G258300.1 --SDGSPSASQ-----------------RHT------RTRTKVQM-------QGIAVGRAVDLTVLKDYDDLIDELEKMFDIKGELQMQT---K-WAITF 607
          AtARF18 --ITPTSMSEQ-----------------KKQQTS---RSRTKVQM-------QGIAVGRAVDLTLLKSYDELIDELEEMFEIQGQLLARD---K-WIVVF 540
          AtARF11 --NSSNSPKEQ-----------------KQQTST---RSRIKVQM-------QGTAVGRAVDLTLLRSYDELIKELEKMFEIEGELSPKD---K-WAIVF 541
Glyma.12G164100.1 --CLRSPQESQ-----------------SRQI-----RSCTKVHM-------QGMAVGRAVDLTRFDGYEDLLRKLEEMFDITGELCGST---KKWQVVY 593
Glyma.16G000300.1 --CLRSPQESQ-----------------SRQI-----RSCTKVHM-------QGMAVGRAVDLTRFDGYEDLLRKLEEMFDINGELCGST---KEWQVVY 593
Glyma.07G272800.1 --CLQSPQESQ-----------------SKQI-----RSCTKVHM-------QGMAVGRAVDLTRFDGYEDLLRKLEDMFNIKTELCGSL---KKWQVVY 629
           AtARF1 --SLRSPQESQ-----------------SRQI-----RSCTKVHM-------QGSAVGRAIDLTRSECYEDLFKKLEEMFDIKGELLEST---KKWQVVY 594
Glyma.06G164900.3 --SQSHTKDVQ-----------------AKPLSG-SARSCTKVHK-------KGIALGRSVDLTKYSGYDELVAELDQLFEFGGELLSTK---KDWLIVY 765
Glyma.04G200600.1 --SQLHTKDVQ-----------------AKPLSG-SARSCTKVHK-------KGIALGRSVDLTKYSGYDELVAELDQLFEFGGELLSTK---KDWLIVF 765
Glyma.05G200800.4 --SQTHLKDIQ-----------------AKSHSG-SARSCTKVHK-------KGIALGRSVDLTKFSDYGELIAELDQLFEFGGLLTSPQ---KDWLIVY 786
Glyma.08G008100.3 --SQPHLKDVQ-----------------AKSHSG-SARSCTKVHK-------KGIALGRSVDLTKFSDYGELITELDQLFEFGGELTSPQ---KDWLIVY 781
           AtARF2 --NNPHPKDAQ-----------------TK--TN-SSRSCTKVHK-------QGIALGRSVDLSKFQNYEELVAELDRLFEFNGELMAPK---KDWLIVY 785
Glyma.03G208800.1 -PMGVAPGKTC------------------KKCRCVNNRSCTKVLKI-------GNALGRAVDLARFNGYTELIAELDSMFDFQGTLISGG---SGWHVTC 546
Glyma.07G202200.1 ------------------------------NKEAADS-SSVAC---------SGPSFTRHVD-DDFHPGHSLRSKAVASYCTKGVLQY*----------- 710
Glyma.13G174000.1 ------------------------------SKEATDS-SAVAC---------AGPSFTRHVE-DDFHPGHSLRSKAVASYCTKGVLQY*----------- 715
Glyma.13G234200.1 ------------------------------KKEASASTITLPLS--------SGPSFTRLVE-DEFHPGHSLPSKGVGSNCTKGVLQY*----------- 737
Glyma.15G078800.1 ------------------------------NEEVSASTITLPLS--------SGPSFTRLVE-DEFHPGHSHPSKAVGSNCTKSVLQY*----------- 729
           AtARF3 ------------------------------ETTAVASATAVPCV--------EGNSMKGASA-VQSNHHHSQGRDIYAMRDMLLDIAL*----------- 609
Glyma.12G071000.1 ------------------------------NLQNSAKRSCTKVHK-------QGSLVGRAIDLSRLSGYNDLLSELERLFSMEGLLKDPD---KGWRILY 716
Glyma.11G154632.1 ------------------------------NLQNSAKRSCTKVHK-------QGSLVGRAIDLSRLSGYNDLLSELERLFSMEGLLKDPD---KGWKILY 716
Glyma.12G171000.1 ------------------------------NLQNSSKRSCTKVHK-------QGSLVGRAIDLSRLSSYNDLLIELERLFSMEGLLIDPN---KGWRILY 716
           AtARF4 ------------------------------KPQSSSKRICTKVHK-------QGSQVGRAIDLSRLNGYDDLLMELERLFNMEGLLRDPE---KGWRILY 717
Glyma.08G100100.3 -----TPENG--------------------GQGNPSNKTFVKVY--------KSGSFGRSLDITKFTSYPELRSELARMFGLEGELEDPV--RSGWQLVF 832
Glyma.05G143800.2 -----TPENG--------------------GQGNPTNKTFVKVY--------KSGSFGRSLDITKFTSYPELRSELARMFGLEGELEDPV--RSGWQLVF 827
Glyma.13G221400.1 -----CLEEA--------------------GQGNPLNKTFVKVY--------KSGSFGRSLDITKFSSYHELRGELARMFGLEGELEDPV--RSGWQLVF 815
Glyma.15G091000.1 -----CPEDA--------------------GQGNPLNKTFVKVY--------KSGSFGRSLDITKFSSYHELRGELARMFGLEGELEDPV--RSGWQLVF 817
           AtARF6 -----SSENL--------------------GSENPQSNTFVKVY--------KSGSFGRSLDISKFSSYHELRSELARMFGLEGQLEDPV--RSGWQLVF 846
Glyma.02G281700.1 -----CSENV--------------------DQANIPTGTFVKVH--------KSGSFGRSLDISKFSSYDELISELARMFGLEGQLEDPKTQRSGWQLVF 814
Glyma.14G032700.1 -----SSENV--------------------DQANTPTGTFVKVH--------KSGSFGRSLDISKFSSYDELISELARMFGLEGQLEDPKTQRSGWQLVF 814
Glyma.11G204200.1 -----SAGHT--------------------DPEN-QTQTFVKVY--------KSGSVGRSLDISRFSSYHELREELAQMFGIEGKLEDPL--RSGWQLVF 770
Glyma.18G046800.1 -----SAGHV--------------------DPEN-QSQTFVKVY--------KSGSVGRSLDISRFSSYHELREELAQMFGIEGKLEDPL--RSGWQLVF 767
Glyma.02G239600.1 -----SAGQV--------------------DPQN-QTRTFVKVY--------KSGSVGRSLDISRFSSYHELREELAQMFGIEGKLEDPL--RSGWQLVF 773
Glyma.14G208500.1 -----SAGQV--------------------DPQN-QTRTFVKVY--------KSGSVGRSLDISRFSSYHELREELAQMFGIEGKLEDPL--RSGWQLVF 769
           AtARF8 -----GAGQI--------------------NSSN-QTKNFVKVY--------KSGSVGRSLDISRFSSYHELREELGKMFAIEGLLEDPL--RSGWQLVF 757
Glyma.09G072200.1 -----NNGLW--------------------ANQTQRMRTYTKVQ--------KCGSVGRCIDVTRYKGYDELRHDLARMFGIEGQLEDPQ--RTEWKLVY 1050
Glyma.15G181000.1 -----NNGLW--------------------ANQTQRMRTYTKVQ--------KCGSVGRCIDVTRYKGYDELRHDLARMFGIEGQLEDPQ--RTEWKLVY 1047
Glyma.17G047100.1 -----NNGLR--------------------TNQTPRMRTYTKVQ--------KRGSVGRCIDVTRYKGYDELRHDLARMFGIEGQLEDPL--RTDWKLVY 1060
Glyma.13G112600.1 -----NNGLR--------------------ANQTPRMRTYTKVQ--------KRGSVGRCIDVTRYKGYDELRHDLARMFGIEGQLEDPL--RTDWKLVY 1055
           AtARF7 -----GGGLW--------------------PAQTQRMRTYTKVQ--------KRGSVGRSIDVNRYRGYDELRHDLARMFGIEGQLEDPQ--TSDWKLVY 1090
          AtARF19 -----GGGLW--------------------PNQTQRMRTYTKVQ--------KRGSVGRSIDVTRYSGYDELRHDLARMFGIEGQLEDPL--TSDWKLVY 1010
Glyma.07G130400.1 -----NSGPWA-PPPAPP---------PLPPAQFQRMRTYTKVY--------KRGAVGRSIDITRYSGYEELKKDLARRFGIEGQLEDRQ--RIGWKLVY 1050
Glyma.01G002100.1 -----NSGPWA-PPPAPPLPPLP----PLPPAQFQRMRTYTKVY--------KRGAVGRSIDITRYSGYEELKQDLARRFGIEGQLEDRQ--RIGWKLVY 1044
Glyma.05G221300.1 -----NRGPWDLPPPPPP-----------QQQQVQRIRTYTKVY--------KRGAVGRSIDITRYSGYEDLKQDLALRFGIEGQLEDLQ--RIGWKLVY 1039
Glyma.08G027800.1 -----NRGPWDLPPPPPPPPPPPPPPPPQQQQQIQRIRTYTKVY--------KRGAVGRSIDITRYSGYEELKQDLALKFGIEGQLEDRE--RIGWKLVY 1053
Glyma.14G217700.1 -------NSWQ-------------------QVPAP-IRTYTKVQ--------KAGSVGRSIDVTTFKNYEELIRAIECMFGLDGLLNDTK--CSGWKLVY 876
Glyma.17G256500.1 -------NSWQ-------------------QVPAP-IRTYTKVQ--------KAGSVGRSIDVTTFKNYEELIRAIECMFGLDGLLNDTK--GSGWKLVY 879
           AtARF5 -----KGSSWQ-------------------KIATPRVRTYTKVQ--------KTGSVGRSIDVTSFKDYEELKSAIECMFGLEGLLTHPQ--SSGWKLVY 845
Glyma.12G174100.1 -------GKSS--------------STTEFSWQVGSDTSHCKVFI-------ESEDVGRTLDLSCLGSYEELYMRLANMFGI----ERSE---MLSHVLY 635
Glyma.13G325200.1 -------GNLS--------------STAEFSWQLGSDTSHCKVFM-------ESEDVGRTLDLSCLSSYQELYMRLANMFGI----ERSD---MLSHVLY 605
Glyma.12G076200.1 ----FSPGK-A--------------SSAEFSWQLGLDTGHCKVFL-------ESEDVGRTLDLSLFGSYEDLYRRLAIMFGI----ERSE---ILNHVLY 637
Glyma.11G145500.1 ----FSPGK-A--------------SSAEFFWQLGLDTGHCKVFL-------ESEDVGRTLDLSQFGSYEELYRRLGNMFGI----ERSE---ILNHVLY 633
          AtARF16 -------GS-P--------------CSKKVHDASGLETGHCKVFM-------ESDDVGRTLDLSVLGSYEELSRKLSDMFGI----KKSE---MLSSVLY 632
          AtARF10 ----------------------------NYSLQ-GLETGHCKVFM-------ESEDVGRTLDLSVIGSYQELYRKLAEMFHIE---ERSD---LLTHVVY 629
Glyma.10G210600.1 --------------------------------------NSHKTSN-------ASDGVGSGLH-------------------------------------- 576
Glyma.20G180000.1 --------------------------------------NSLKTSN-------ASD--------------------------------------------- 567
Glyma.10G053500.1 EHSSCERFQWC--------------KDNHQETEAGLEIGHCKVFM-------ESEDVGRTMDLSLLRSYDELHRKLADMFGI----EKSE---MLSHVLY 662
Glyma.13G140600.1 ----------S--------------NNRHCKSLASVRTSLAYYL*------------------------------------------------------- 515
Glyma.19G181900.2 --SSLERLQWY--------------KDQQKETMASLETGHCKVFM-------ESEDIGRTMDLTMLGSYDELYRKLADMFGI----EKSV---VLSHMLY 662
Glyma.13G084700.1 ---------------------------------------------------------------------------------------------------- 536
Glyma.14G166500.1 ---------------------------------------------------------------------------------------------------- 533
Glyma.04G254200.1 ---------------------------------------------------------------------------------------------------- 548
          AtARF17 ---------------------------------------------------------------------------------------------------- 552

        Consensus --+++++++++-++++++-------++++++++++++++++++++-------++++++++++++++++++++++++++++++++++++++--++++++++ 384

                                       1520                1540                1560                1580                1600
```

```
Glyma.01G103500.1  TDDEGDMMLVGDDPWP------EFCNMVRRIFICSSQDVKKMSCGS---KLPIS------------SVEDG------TVISSD-------------TTET  692
Glyma.03G070500.1  TDDEGDMMLVGDDPWP------EFCNMVRRIFICSSQDVKKMSCGS---KLPIS------------SVEDG------TVISSD-------------TTET  691
Glyma.07G134800.1  TDDEGDMMLVGDDPWP------EFCNMVKRIFICSSQDVHKLSSGS---KLPIS------------SMGE-------IVISLN-------------TTET  664
Glyma.18G184500.1  TDDEGDMMLVGDDPWL------EFCKMVRRIFIYSSQDVHKLSSGS---KLPIS------------SMEE-------IVTSLD-------------TTET  664
         AtARF21  TDSDGYEMLVGDDPWP------EFCKMVKKILIYSKEEVKNLK--------SSK------------------------------------------SLSS  606
         AtARF20  TDSDGYEMLVGDDPWP------EFCKMVKKILIYSKEEVKNLK--------SSK------------------------------------------SLSS  592
         AtARF15  TGSDEDEMLVGDDPWP------EFCNMVKRIYIQKR---------------------------------------------------------------R  593
         AtARF23  ----------------------------------------------------------------------------------------------------  223
         AtARF12  TDSDEDKMLVGDDPWP------EFCNMVKKIFIQKR---------------------------------------------------------------R  593
         AtARF22  TDSDDDKMLVGDDPWP------EFCNMVKKILIFKRG--------------GQK------------------------------------------LEVQ  598
         AtARF14  TNNEEDKMLVGEDPWP------EFCNMVKKIFIYSKEEVKNLK--------SRK------------------------------------------SLSS  605
         AtARF13  ----------------------------------------------------------------------------------------------------  506
          AtARF9  TDDEGDMMLVGDDPWP------EFCNMVKRIFIWSKEEVKKMTPGN-QLRMLLR------------EVETTL----TTTSKTD-------------NHSN  638
Glyma.16G023600.1  TDDENDMMLVGDDPWP------EFCNMVKRIFICSREDLKKMKC----CKLPAS------------SSEVE-----EVLLSPDSQNRD----ETQQSHMP  716
Glyma.07G054800.1  TDDENDMMLAGDDPWP------EFCNMVKRIFICSREDLKKMKC----CKLPAS------------SSEVE-----EILLSPDSQNRD----ETQQSHMP  716
Glyma.03G258300.1  TDDGNDMMLVGDDPWP------EFCTVVKRIFICSREDVKKMKS-----KHSSS------------SSVGE-----ETLLSQD-----------------  662
         AtARF18  TDDEGDMMLAGDDPWN------EFCKMAKKIFIYSSDEVKKMTT-----KLKIS------------SSLEN------EEYGNESFE---------NRSRG  602
         AtARF11  TDDEGDRMLVGDDPWN------EFCKMAKKLFIYPSDEVKKMRS-----KSLLG------------D--KG------TIVNLESDQ---------RTVHV  601
Glyma.12G164100.1  TDNEDDMMMVGDDPWL------EFCSIVRKIFIYTAEEVRKLSPKI---GLPIS------------EEVKPSKMDSEAVANPEDQSSI-------VGPGC  665
Glyma.16G000300.1  TDNEDDMMMVGDDPWL------EFCSIVRKIFIYTAEEVKKLSPKI---GLPIS------------EEVKPCKMDSEAVVNPGDQSSI-------LGPGC  665
Glyma.07G272800.1  TDNEDDMMMVGDDPWD------EFCSVVRKIFIYTAEEVKKLSPKI---RLPMN------------EKVNPSKQDSEAIVNPEDQSSN-------MGPSC  701
          AtARF1  TDDEDDMMMVGDDPWN------EFCGMVRKIFIYTPEEVKKLSPKN---KLAVN------------ARMQ-LKADAEENGNTEGRSSS-------MAGSR  665
Glyma.06G164900.3  TDNEGDMMLVGDDPWQ------EFCAMVCKIYIYPKEEIQKMSPGTLSSKNEEN------------QSVMASDGADAKVVKCQPHQKF----NSENGLDA  843
Glyma.04G200600.1  TDNEGDMMLVGDDPWQ------EFCAMVRKIYIYPKEEIQKMSPGTLSSKNEEN------------HSVTASEGADTKDVKCQPHQKF----NSENGLDA  843
Glyma.05G200800.4  TDNEGDMMLVGDDPWQ------EFVAMVRKIYIYPKEEIQKMSPGTLSSKNEEN------------QSASEG-ATDTQEIKCQLNN---------SASDT  858
Glyma.08G008100.3  TDNEGDMMLVGDDPWQ------EFVAMVRKIYIYPKEEIQKMSPGTLSSKNEEN------------QSASEG-AADAQEIECQLNH---------SSSDT  853
          AtARF2  TDEENDMMLVGDDPWQ------EFCCMVRKIFIYTKEEVRKMNPGTLSCRSEEE------------AVVGEG--SDAKDAKSASNPSL----SS--AGNS  859
Glyma.03G208800.1  LDDEGDMMQLGDYPWQ------DFLGVVQKMIICPKEGTDNLKPGS--------------------------------------------------SANP  590
Glyma.07G202200.1  ----------------------------------------------------------------------------------------------------  710
Glyma.13G174000.1  ----------------------------------------------------------------------------------------------------  715
Glyma.13G234200.1  ----------------------------------------------------------------------------------------------------  737
Glyma.15G078800.1  ----------------------------------------------------------------------------------------------------  729
          AtARF3  ----------------------------------------------------------------------------------------------------  609
Glyma.12G071000.1  TDSENDIMVVGDDPWH------EFCDVVSKIHIYTQEEVEKMTIG-MISDDTHS-----------------CLEEAPVIMEASKSSSVGQPDYSPTAVRV  792
Glyma.11G154632.1  TDSENDIMVVGDDPWH------EFCDVVSKIHIYTQEEVEKMTIE-MISDDTHS-----------------CLEEAPVIMEASKSSSVGQPDYSPTAVRV  792
Glyma.12G171000.1  TDSENDIMVVGDDPWHLPFVCSEFCDVVSKIHIHTQEEVEKMTIGMMINDDSQS-----------------CLEQAPVMVEASKSSSVGQPDSSPTVVRM  799
          AtARF4  TDSENDMMVVGDDPWH------DFCNVVWKIHLYTKEEVENA------NDDNKS-----------------CLEQAALMMEASKSSSVSQPDSSPTITRV  788
Glyma.08G100100.3  VDQENDVLLLGDGPWP------EFVNSVGYIKILSPQEVQQMGNNELELLNSVP----IQRL--SNGVC------EDPRNLSTGITTV-------GSLNY  907
Glyma.05G143800.2  VDQENDVLLLGDGPWP------EFVNSVGCIKILSPQEVQQMGNNGLELLNSVP----IQRL--SNGVCDDYAGREDPRNLSTGITIV-------GSLNY  908
Glyma.13G221400.1  VDRENDVLLLGDGPWP------EFVNSVWCIKILSPQEVQQMGNNGLELLNSFP----IQRL--SNGICDDYVSRQDPRNLGTGITTV-------GSLDY  896
Glyma.15G091000.1  VDRENDVLLLGDGPWP------EFVNSVWCIKILSPQEVQQMGNNGLELLNSVP----NQRL--SNGICDDYVSRQDPRNLSTGITTV-------GSLDY  898
          AtARF6  VDRENDVLLLGDDPWP------EFVSSVWCIKILSPQEVQQMGKRGLELLNSAPSSNNVDKLP-SNGNCDDFGNRSDPRNLGNGIASVG------GSFNY  933
Glyma.02G281700.1  VDRENDVLLLGDDPWQ------EFVNNVWYIKILSPLEVQQMG-KVLSPSTSAP----GDKLSTPVNSCDNYVSQQELRSSRNGMASM-------GSFHY  896
Glyma.14G032700.1  VDRENDVLLLGDDPWQ------EFVNNVWYIKILSPLEVQQMG-KGLSPSTSAP----GNKLSTPANSCDNYVSQQELRSSRNGMASM-------GSFHY  896
Glyma.11G204200.1  VDRENDVLLLGDDPWE------SFVNNVWYIKILSPEDIQKMGDQAVESLALGS-------------GQRLNGTGAESQDIVSGPPSI-------GSLEY  844
Glyma.18G046800.1  VDRENDVLLLGDDPWE------SFVNNVWYIKILSPEDIQKMGEQAVESLALGS-------------GQRLNGTGAESQDIVSGPPSI-------GSLEY  841
Glyma.02G239600.1  VDRENDVLLLGDDPWE------SFVNNVWYIKILSPEDIHKMGEQALESLGPSP-------------GQRLNSTGADSHEIVSGLPSI-------GSLEY  847
Glyma.14G208500.1  VDRENDVLLLGDDPWE------SFVNNVWYIKILSPEDIHKMGEQAVESLGPSS-------------GHRLNSTGADSHEIVSGLPSI-------GSLEY  843
          AtARF8  VDKENDILLLGDDPWE------SFVNNVWYIKILSPEDVHQMGD-----------------------------HGEGSGGLFPQNP-----------THL  811
Glyma.09G072200.1  VDHENDILLVGDDPWE------EFVSCVQSIKILSSSEVQQMSLDGDLGHVPVP-----------NQACSGTDNGNAWRGQYDDNS-A-------ASFNR  1125
Glyma.15G181000.1  VDHENDILLVGDDPWE------EFVSCVQSIKILSSAEVQKMSLDGDLGHVPVP-----------NQACSGTDNGNAWRGQYEDNS-A-------ASFNR  1122
Glyma.17G047100.1  VDHENDILLVGDDPWD------EFVSCVQSIKILSSAEVQQMSLDGDLGGNVPI----------PNQACSGTDSGNAWRGQYEDNS-A-------ASFNR  1136
Glyma.13G112600.1  VDHENDILLVGDDPWD------EFVSCVQSIKILSSAEVQQMSLDGDLGGNVPI----------PNQAYSGTDSGNAWRGQYEDNS-A-------ASFNR  1131
          AtARF7  VDHENDILLVGDDPWE------EFVNCVQSIKILSSAEVQQMSLDGNFAGVPVT-----------NQACSGGDSGNAWRGHYDDNS-A-------TSFNR  1165
         AtARF19  TDHENDILLVGDDPWE------EFVNCVQNIKILSSVEVQQMSLDGDLAAIPTT-----------NQACSETDSGNAWKVHYEDTSAA-------ASFNR  1086
Glyma.07G130400.1  VDHESDVLLVGDDPWE------EFVNCVRCIKILSPQEVQQMSLDGDFGNGGLQ-----------NQACSSSD-----------------------GGNT  1110
Glyma.01G002100.1  VDHESDVLLLGDDPWE------EFVNCVRCIKILSPQEVQQMSLDGDFGNGGLP-----------NQACSSSD-----------------------GGDT  1104
Glyma.05G221300.1  VDHENDVLLVGDDPWE------EFVNCVRCIKILSPQEVQQMSLDGDFGNGGLP-----------YPAGSSSD-----------------------GGNA  1099
Glyma.08G027800.1  VDHENDVLLVGDDPWE------EFVNCVRCIKILSPQEVQQMSLDGDFGNGGLP-----------YPAGSSSG-----------------------GGNA  1113
Glyma.14G217700.1  VDYESDVLLVGDDPWE------EFVGCVRCIRILSPSEVQQMSEEGMKLLNSGA---------------------------LQGM-------------NV  930
Glyma.17G256500.1  VDYESDVLLVGDDPWE------EFVGCVRCIRILSPSEVQQMSEEGMKLLNSGA---------------------------LQGM-------------NV  933
          AtARF5  VDYESDVLLVGDDPWE------EFVGCVRCIRILSPTEVQQMSEEGMKLLNSAG---------------------------INDLK----------TSVS  902
Glyma.12G174100.1  RDAAGALKQTGEEPFS------EFMKTAKRLTILTDSNNKDSRRVWITGIRNAE---------------------HGLDTASNKTGPL--------GIFA  700
Glyma.13G325200.1  CDSSGALKQIGEEPFS------EFMKTAKRLTILTDSNNKDSRRVWITGTRNAE---------------------HGLDAASNKTGPL--------SIFA  670
Glyma.12G076200.1  HDAAGAAKKTGEEPFS------DFMKTAKRLTILTDSSSKNIKRAFITGTRNGE---------------------HGLD-ASNKTGPL--------SIFA  701
Glyma.11G145500.1  YDAAGAVKQTGEEPFS------DFMKTAKRLTILTDSGSKNIKRAFITGTRNGE---------------------HGLD-PSNKTGPL--------SIFA  697
         AtARF16  RDASGAIKYAGNEPFS------EFLKTARRLTILTEQGSE--------------------------------------------------------SVVV  670
         AtARF10  RDANGVIKRIGDEPFS------DFMKATKRLTIKMDIGGDNVRKTWITGIRTGE---------------------NGID-ASTKTGPL--------SIFA  693
Glyma.10G210600.1  ---QGSPIENNSDGGS------PWYKDQHKSDLGTDNVNT-----------------------------------------------L--------CIAL  612
Glyma.20G180000.1  -------------GGS------PWYKEQHKSDLGNDNVNT-----------------------------------------------L--------CIAL  593
Glyma.10G053500.1  RDSTGAVKRISDESFS------DFTRTAKRLTILMDSGSN--------------------------------------------------------NVGV  700
Glyma.13G140600.1  ----------------------------------------------------------------------------------------------------  515
Glyma.19G181900.2  RDTTGAVKHIGDEAFS------EFTKTARRLTILMDSNSD--------------------------------------------------------GRGI  700
Glyma.13G084700.1  --------------GS------NKLKTVEACYFSK-----------------------------------------------------------------  551
Glyma.14G166500.1  --------------GS------NKLKTVEACYFSK-----------------------------------------------------------------  548
Glyma.04G254200.1  -------------------------CTGDECQCQR-------------------------------------------------------------ALNS  562
         AtARF17  -----------------------KESSDNETQLSLSHAPPSVPK------------------------------------HSNSNAGS--------SSQG  585

       Consensus  ++++++++++++++XX------XXXXXXXXXXJXXX++++++++++++++++++----++++--+++++++++++++++++++++++++++++++++++X  401
```

```
                   '
Glyma.01G103500.1  *    693
Glyma.03G070500.1  *    692
Glyma.07G134800.1  *    665
Glyma.18G184500.1  *    665
         AtARF21  *    607
         AtARF20  *    593
         AtARF15  *    594
         AtARF23  -    223
         AtARF12  *    594
         AtARF22  *    599
         AtARF14  *    606
         AtARF13  -    506
          AtARF9  *    639
Glyma.16G023600.1  *    717
Glyma.07G054800.1  *    717
Glyma.03G258300.1  *    663
         AtARF18  *    603
         AtARF11  *    602
Glyma.12G164100.1  *    666
Glyma.16G000300.1  *    666
Glyma.07G272800.1  *    702
```

AtARF1	*	666
Glyma.06G164900.3	*	844
Glyma.04G200600.1	*	844
Glyma.05G200800.4	*	859
Glyma.08G008100.3	*	854
AtARF2	*	860
Glyma.03G208800.1	*	591
Glyma.07G202200.1	-	710
Glyma.13G174000.1	-	715
Glyma.13G234200.1	-	737
Glyma.15G078800.1	-	729
AtARF3	-	609
Glyma.12G071000.1	*	793
Glyma.11G154632.1	*	793
Glyma.12G171000.1	*	800
AtARF4	*	789
Glyma.08G100100.3	*	908
Glyma.05G143800.2	*	909
Glyma.13G221400.1	*	897
Glyma.15G091000.1	*	899
AtARF6	*	934
Glyma.02G281700.1	*	897
Glyma.14G032700.1	*	897
Glyma.11G204200.1	*	845
Glyma.18G046800.1	*	842
Glyma.02G239600.1	*	848
Glyma.14G208500.1	*	844
AtARF8	*	812
Glyma.09G072200.1	*	1126
Glyma.15G181000.1	*	1123
Glyma.17G047100.1	*	1137
Glyma.13G112600.1	*	1132
AtARF7	*	1166
AtARF19	*	1087
Glyma.07G130400.1	*	1111
Glyma.01G002100.1	*	1105
Glyma.05G221300.1	*	1100
Glyma.08G027800.1	*	1114
Glyma.14G217700.1	*	931
Glyma.17G256500.1	*	934
AtARF5	*	903
Glyma.12G174100.1	*	701
Glyma.13G325200.1	*	671
Glyma.12G076200.1	*	702
Glyma.11G145500.1	*	698
AtARF16	*	671
AtARF10	*	694
Glyma.10G210600.1	*	613
Glyma.20G180000.1	*	594
Glyma.10G053500.1	*	701
Glyma.13G140600.1	-	515
Glyma.19G181900.2	*	701
Glyma.13G084700.1	*	552
Glyma.14G166500.1	*	549
Glyma.04G254200.1	*	563
AtARF17	*	586
Consensus	*	402
