# Supplementary material for: Identification of potential auxin response candidate genes for soybean rapid canopy coverage through comparative evolution and expression analysis
Source: Front Plant Sci. 2024 Oct 3;15:1463438. doi: 10.3389/fpls.2024.1463438 (PMC11484095; doi:10.3389/fpls.2024.1463438)
Supplement: Supplementary file 1 [file DataSheet1.zip › Supplemental information.docx]

**Supplemental Information for:**

**Identification of potential auxin response candidate genes for soybean rapid canopy coverage through comparative evolution and expression analysis**

Deisiany Ferreira Neres^1,2^, Joseph S. Taylor^2,3^, John A. Bryant Jr.^1,2,4^, Bastiaan O. R. Bargmann^2,3^, R. Clay Wright^1,2*^

^1^ Biological Systems Engineering, Virginia Polytechnic Institute and State University, Blacksburg Virginia United States.

^2^ Translational Plant Science Center, Virginia Polytechnic Institute and State University, Blacksburg Virginia United States.

^3^School of Plant and Environmental Sciences, Virginia Polytechnic Institute and State University, Blacksburg Virginia United States.

^4^Current affiliation: Department of Structural Biology, St. Jude Children’s Research Hospital, Memphis, TN, USA.

^*^ Corresponding author: wrightrc@vt.edu


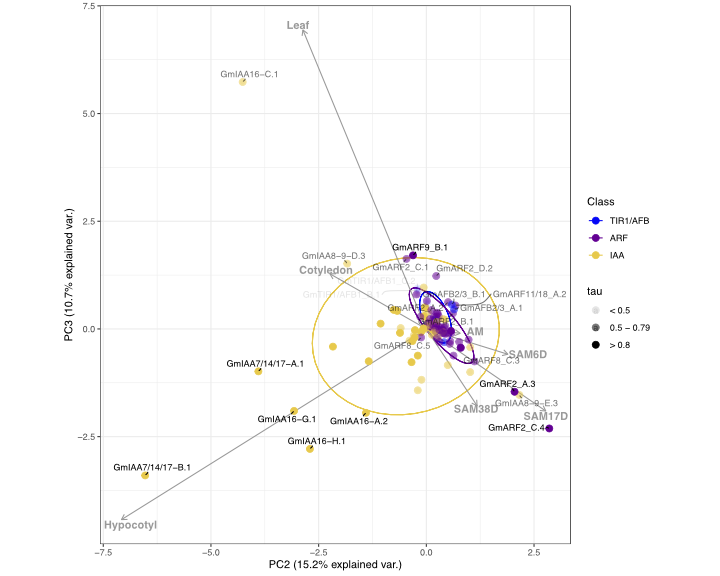


**Figure S1. Correlation-based Principal Component Analysis (PCA): biplot of gene transcript expression and explanatory tissues involved in plant aerial architecture as eigenvectors (grey arrows), n = 7). Principal components 2 and 3 account for 25.9% of the total inertia. Ellipses are used here as a visual representation of dispersion of data points within each group (TIR1/AFB, ARF, and Aux/IAA (IAA)) with a 70% confidence interval. TIR1/AFB genes are colored cyan, ARF genes are colored purple, and Aux/IAA (IAA) genes are colored yellow. Some labels are connected to their respective points with hard lines. Both data points and label are also determined by the gene tissue specificity index (tau), with tau < 0.5 represented in light grey, tau between 0.5 and 0.79 appearing as grey, and those with tau > 0.8 in black. Genes clustering together inside the ellipses, and/or having smaller tau values, are hypothesized to have more pleiotropic effects on plant growth and development., Conversely, genes associated with a specific RCC tissue (genes that fall along an eigenvector, outside of the respectively colored ellipse, and/or having intermediate to high tau) are hypothesized to have narrower effects and be more amenable to engineering RCC traits through gene editing.**

**Figure S2. Correlation-based Principal Component Analysis (PCA): biplot of gene transcript expression and explanatory tissues involved in plant growth as eigenvectors (grey arrows), n = 7). Principal components 1 and 2 account for 69.8% of the total inertia. Both data points and label are also determined by the gene tissue specificity index (tau), with tau < 0.5 represented in light grey, tau between 0.5 and 0.79 appearing as grey, and those with tau > 0.8 in black.**

**Figure S3. Correlation-based Principal Component Analysis (PCA): biplot of gene transcript expression and explanatory tissues involved in plant growth as eigenvectors (grey arrows), n = 14). Principal components 3 and 4 account for 14.1% of the total inertia. Both data points and label are also determined by the gene tissue specificity index (tau), with tau < 0.5 represented in light grey, tau between 0.5 and 0.79 appearing as grey, and those with tau > 0.8 in black.**


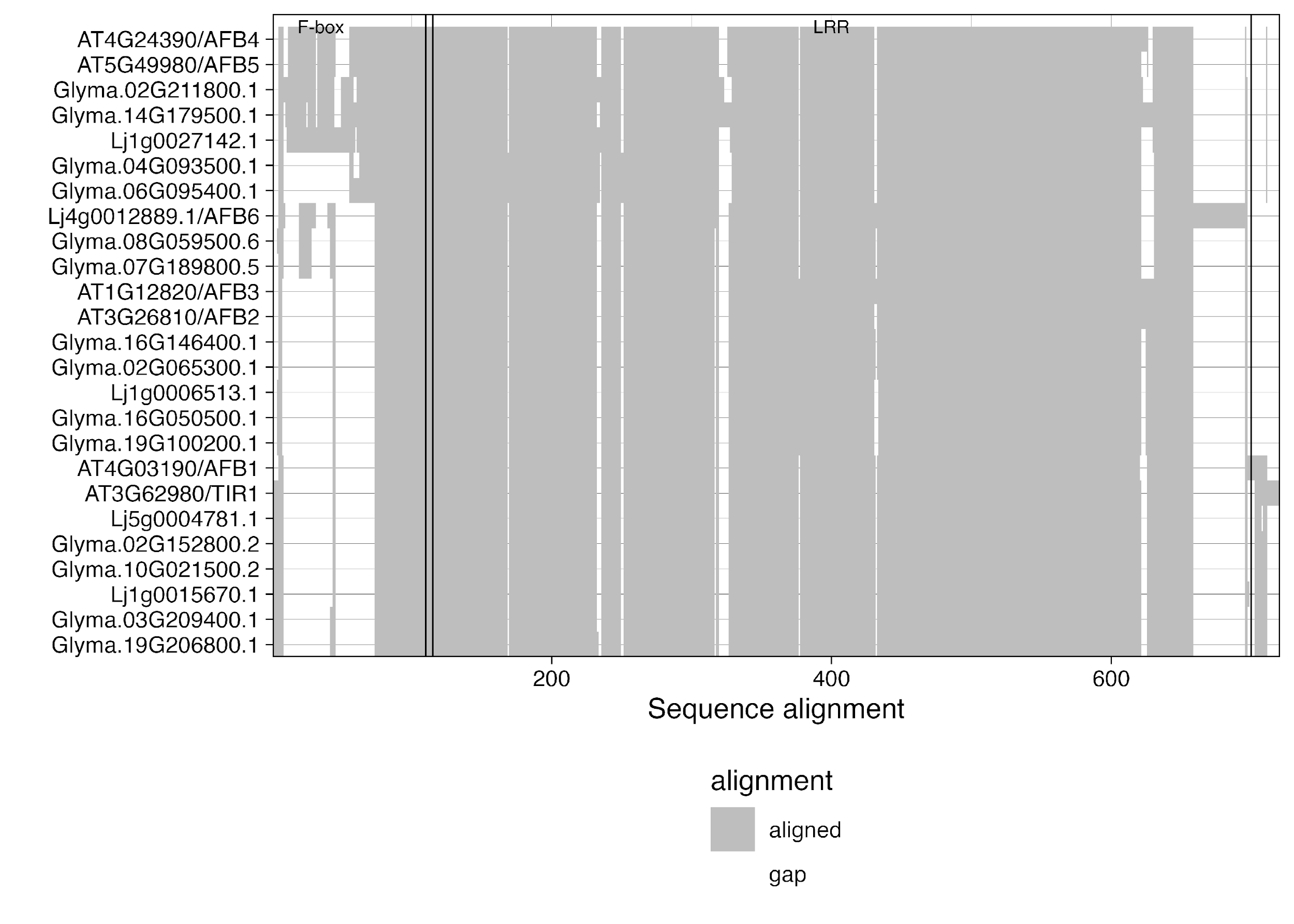


**Figure S4. Critical functional domain similarity shared between *A. thaliana, L. japonicus* and *G. max* TIR1/AFB protein sequences. Conserved critical domains are depicted by the F-box label and its black line marking the end of it, which then follows a second black line demarking the start of the LRR region. Complete alignment showing conserved amino acids can be found in Appendix A. Protein sequences were aligned with DECIPHER, Wright (2015), in both cases.**


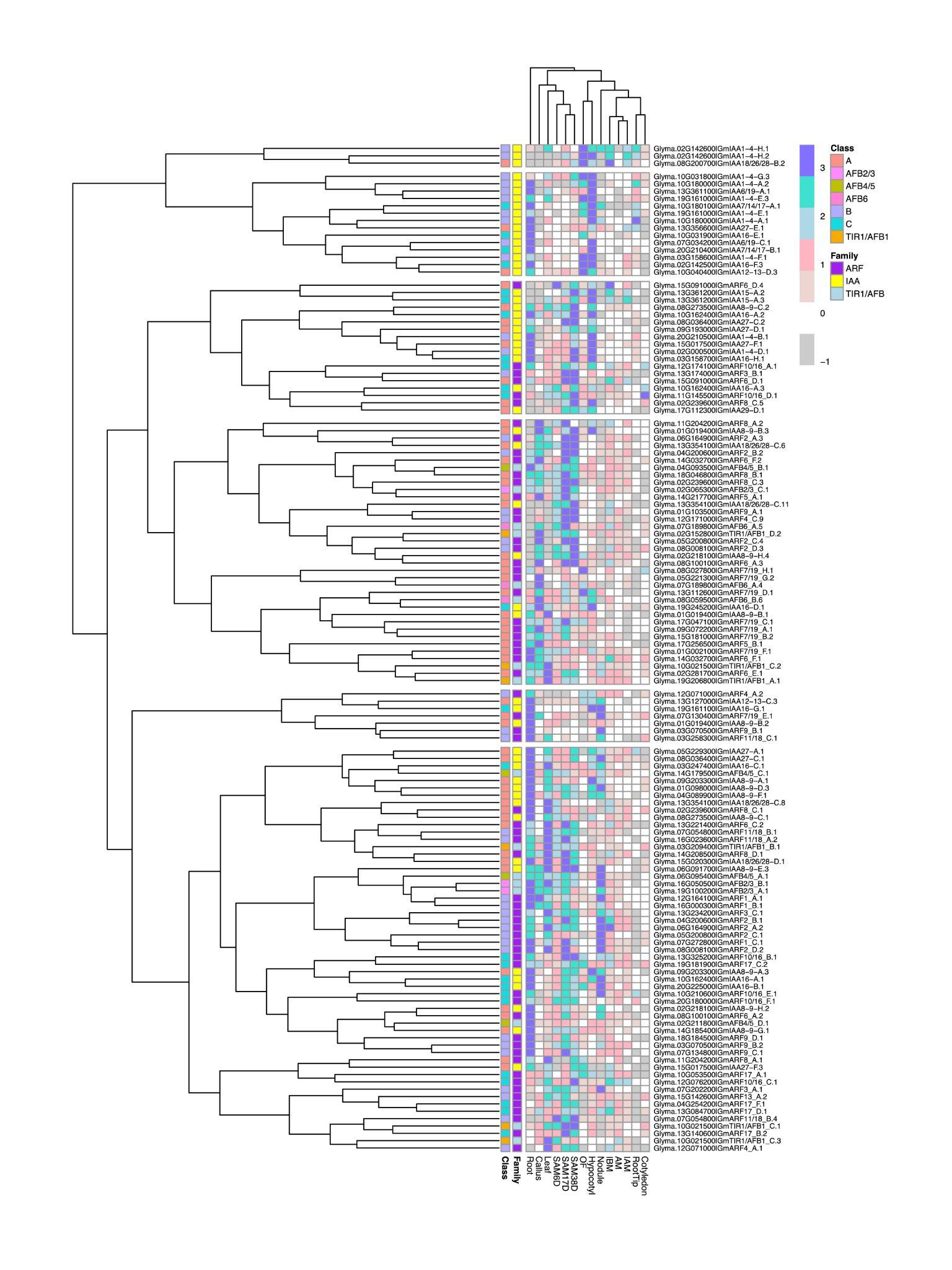


**Figure S5. Heatmap of gene expression of G. max. Colors in the heatmap represent the z-score values of gene expression levels. The scale ranges from -1 in grey, representing gene expression levels below the mean of their respective rows. White color depicts values close to 0 or equal to 0, in which genes have expression levels similar to the mean expression of transcripts. Finally, positive values, ranging from 1 to 3, are depicted here in misty rose to blue and indicate gene expression levels above the mean expression of transcripts.**

**Figure S6. Correlation-based Principal Component Analysis (PCA): biplot of gene transcript expression and explanatory tissues involved in plant aerial architecture as eigenvectors (grey arrows), n = 7). Principal components 3 and 4 account for 14.3% of the total inertia. Both data points and label are also determined by the gene tissue specificity index (tau), with tau < 0.5 represented in light grey, tau between 0.5 and 0.79 appearing as grey, and those with tau > 0.8 in black.**

**Table S1. Full list of *G. max* orthologous genes with Gene Identities, its assigned orthology, transcript identifier, classification , assigned clade and lastly its assigned family.**

| **Gene ID** | **Orthology** | **Transcript ID** | **Class** | **Clade** | **Family** |
| --- | --- | --- | --- | --- | --- |
| Glyma.19G206800 | GmTIR1/AFB1_A.1 | Glyma.19G206800.1 | TIR1/AFB1 | I | TIR1/AFB |
| Glyma.03G209400 | GmTIR1/AFB1_B.1 | Glyma.03G209400.1 | TIR1/AFB1 | I | TIR1/AFB |
| Glyma.10G021500 | GmTIR1/AFB1_C.1 | Glyma.10G021500.1 | TIR1/AFB1 | I | TIR1/AFB |
| Glyma.10G021500 | GmTIR1/AFB1_C.2 | Glyma.10G021500.2 | TIR1/AFB1 | I | TIR1/AFB |
| Glyma.10G021500 | GmTIR1/AFB1_C.3 | Glyma.10G021500.3 | TIR1/AFB1 | I | TIR1/AFB |
| Glyma.02G152800 | GmTIR1/AFB1_D.1 | Glyma.02G152800.1 | TIR1/AFB1 | I | TIR1/AFB |
| Glyma.02G152800 | GmTIR1/AFB1_D.2 | Glyma.02G152800.2 | TIR1/AFB1 | I | TIR1/AFB |
| Glyma.19G100200 | GmAFB2/3_A.1 | Glyma.19G100200.1 | AFB2/3 | II | TIR1/AFB |
| Glyma.16G050500 | GmAFB2/3_B.1 | Glyma.16G050500.1 | AFB2/3 | II | TIR1/AFB |
| Glyma.02G065300 | GmAFB2/3_C.1 | Glyma.02G065300.1 | AFB2/3 | II | TIR1/AFB |
| Glyma.16G146400 | GmAFB2/3_D.1 | Glyma.16G146400.1 | AFB2/3 | II | TIR1/AFB |
| Glyma.07G189800 | GmAFB6_A.3 | Glyma.07G189800.3 | AFB6 | III | TIR1/AFB |
| Glyma.07G189800 | GmAFB6_A.4 | Glyma.07G189800.4 | AFB6 | III | TIR1/AFB |
| Glyma.07G189800 | GmAFB6_A.5 | Glyma.07G189800.5 | AFB6 | III | TIR1/AFB |
| Glyma.08G059500 | GmAFB6_B.6 | Glyma.08G059500.6 | AFB6 | III | TIR1/AFB |
| Glyma.06G095400 | GmAFB4/5_A.1 | Glyma.06G095400.1 | AFB4/5 | IV | TIR1/AFB |
| Glyma.04G093500 | GmAFB4/5_B.1 | Glyma.04G093500.1 | AFB4/5 | IV | TIR1/AFB |
| Glyma.14G179500 | GmAFB4/5_C.1 | Glyma.14G179500.1 | AFB4/5 | IV | TIR1/AFB |
| Glyma.02G211800 | GmAFB4/5_D.1 | Glyma.02G211800.1 | AFB4/5 | IV | TIR1/AFB |
| Glyma.18G030200 | GmCOI1_A.1 | Glyma.18G030200.1 | COI1 | V | TIR1/AFB |
| Glyma.11G227300 | GmCOI1_B.1 | Glyma.11G227300.1 | COI1 | V | TIR1/AFB |
| Glyma.16G023600 | GmARF11/18_A.1 | Glyma.16G023600.1 | B | I | ARF |
| Glyma.16G023600 | GmARF11/18_A.2 | Glyma.16G023600.2 | B | I | ARF |
| Glyma.16G023600 | GmARF11/18_A.3 | Glyma.16G023600.3 | B | I | ARF |
| Glyma.07G054800 | GmARF11/18_B.1 | Glyma.07G054800.1 | B | I | ARF |
| Glyma.07G054800 | GmARF11/18_B.2 | Glyma.07G054800.2 | B | I | ARF |
| Glyma.07G054800 | GmARF11/18_B.3 | Glyma.07G054800.3 | B | I | ARF |
| Glyma.07G054800 | GmARF11/18_B.4 | Glyma.07G054800.4 | B | I | ARF |
| Glyma.03G258300 | GmARF11/18_C.1 | Glyma.03G258300.1 | B | I | ARF |
| Glyma.15G142600 | GmARF13_A.1 | Glyma.15G142600.1 | B | I | ARF |
| Glyma.15G142600 | GmARF13_A.2 | Glyma.15G142600.2 | B | I | ARF |
| Glyma.15G142600 | GmARF13_A.3 | Glyma.15G142600.3 | B | I | ARF |
| Glyma.12G164100 | GmARF1_A.1 | Glyma.12G164100.1 | B | I | ARF |
| Glyma.16G000300 | GmARF1_B.1 | Glyma.16G000300.1 | B | I | ARF |
| Glyma.07G272800 | GmARF1_C.1 | Glyma.07G272800.1 | B | I | ARF |
| Glyma.06G164900 | GmARF2_A.2 | Glyma.06G164900.2 | B | I | ARF |
| Glyma.06G164900 | GmARF2_A.3 | Glyma.06G164900.3 | B | I | ARF |
| Glyma.04G200600 | GmARF2_B.1 | Glyma.04G200600.1 | B | I | ARF |
| Glyma.04G200600 | GmARF2_B.2 | Glyma.04G200600.2 | B | I | ARF |
| Glyma.05G200800 | GmARF2_C.1 | Glyma.05G200800.1 | B | I | ARF |
| Glyma.05G200800 | GmARF2_C.4 | Glyma.05G200800.4 | B | I | ARF |
| Glyma.05G200800 | GmARF2_C.5 | Glyma.05G200800.5 | B | I | ARF |
| Glyma.08G008100 | GmARF2_D.2 | Glyma.08G008100.2 | B | I | ARF |
| Glyma.08G008100 | GmARF2_D.3 | Glyma.08G008100.3 | B | I | ARF |
| Glyma.03G208800 | GmARF2_E.1 | Glyma.03G208800.1 | B | I | ARF |
| Glyma.07G202200 | GmARF3_A.1 | Glyma.07G202200.1 | B | I | ARF |
| Glyma.13G174000 | GmARF3_B.1 | Glyma.13G174000.1 | B | I | ARF |
| Glyma.13G174000 | GmARF3_B.2 | Glyma.13G174000.2 | B | I | ARF |
| Glyma.13G174000 | GmARF3_B.3 | Glyma.13G174000.3 | B | I | ARF |
| Glyma.13G234200 | GmARF3_C.1 | Glyma.13G234200.1 | B | I | ARF |
| Glyma.12G071000 | GmARF4_A.1 | Glyma.12G071000.1 | B | I | ARF |
| Glyma.12G071000 | GmARF4_A.2 | Glyma.12G071000.2 | B | I | ARF |
| Glyma.12G171000 | GmARF4_C.1 | Glyma.12G171000.1 | B | I | ARF |
| Glyma.12G171000 | GmARF4_C.2 | Glyma.12G171000.2 | B | I | ARF |
| Glyma.12G171000 | GmARF4_C.3 | Glyma.12G171000.3 | B | I | ARF |
| Glyma.12G171000 | GmARF4_C.4 | Glyma.12G171000.4 | B | I | ARF |
| Glyma.12G171000 | GmARF4_C.5 | Glyma.12G171000.5 | B | I | ARF |
| Glyma.12G171000 | GmARF4_C.6 | Glyma.12G171000.6 | B | I | ARF |
| Glyma.12G171000 | GmARF4_C.7 | Glyma.12G171000.7 | B | I | ARF |
| Glyma.12G171000 | GmARF4_C.8 | Glyma.12G171000.8 | B | I | ARF |
| Glyma.12G171000 | GmARF4_C.9 | Glyma.12G171000.9 | B | I | ARF |
| Glyma.01G103500 | GmARF9_A.1 | Glyma.01G103500.1 | B | I | ARF |
| Glyma.01G103500 | GmARF9_A.2 | Glyma.01G103500.2 | B | I | ARF |
| Glyma.03G070500 | GmARF9_B.1 | Glyma.03G070500.1 | B | I | ARF |
| Glyma.03G070500 | GmARF9_B.2 | Glyma.03G070500.2 | B | I | ARF |
| Glyma.07G134800 | GmARF9_C.1 | Glyma.07G134800.1 | B | I | ARF |
| Glyma.18G184500 | GmARF9_D.1 | Glyma.18G184500.1 | B | I | ARF |
| Glyma.18G184500 | GmARF9_D.2 | Glyma.18G184500.2 | B | I | ARF |
| Glyma.18G184500 | GmARF9_D.3 | Glyma.18G184500.3 | B | I | ARF |
| Glyma.14G217700 | GmARF5_A.1 | Glyma.14G217700.1 | A | II | ARF |
| Glyma.17G256500 | GmARF5_B.1 | Glyma.17G256500.1 | A | II | ARF |
| Glyma.08G100100 | GmARF6_A.2 | Glyma.08G100100.2 | A | II | ARF |
| Glyma.08G100100 | GmARF6_A.3 | Glyma.08G100100.3 | A | II | ARF |
| Glyma.13G221400 | GmARF6_C.1 | Glyma.13G221400.1 | A | II | ARF |
| Glyma.13G221400 | GmARF6_C.2 | Glyma.13G221400.2 | A | II | ARF |
| Glyma.15G091000 | GmARF6_D.1 | Glyma.15G091000.1 | A | II | ARF |
| Glyma.15G091000 | GmARF6_D.2 | Glyma.15G091000.2 | A | II | ARF |
| Glyma.15G091000 | GmARF6_D.3 | Glyma.15G091000.3 | A | II | ARF |
| Glyma.15G091000 | GmARF6_D.4 | Glyma.15G091000.4 | A | II | ARF |
| Glyma.02G281700 | GmARF6_E.1 | Glyma.02G281700.1 | A | II | ARF |
| Glyma.02G281700 | GmARF6_E.2 | Glyma.02G281700.2 | A | II | ARF |
| Glyma.02G281700 | GmARF6_E.3 | Glyma.02G281700.3 | A | II | ARF |
| Glyma.14G032700 | GmARF6_F.1 | Glyma.14G032700.1 | A | II | ARF |
| Glyma.14G032700 | GmARF6_F.2 | Glyma.14G032700.2 | A | II | ARF |
| Glyma.09G072200 | GmARF7/19_A.1 | Glyma.09G072200.1 | A | II | ARF |
| Glyma.15G181000 | GmARF7/19_B.1 | Glyma.15G181000.1 | A | II | ARF |
| Glyma.15G181000 | GmARF7/19_B.2 | Glyma.15G181000.2 | A | II | ARF |
| Glyma.17G047100 | GmARF7/19_C.1 | Glyma.17G047100.1 | A | II | ARF |
| Glyma.13G112600 | GmARF7/19_D.1 | Glyma.13G112600.1 | A | II | ARF |
| Glyma.07G130400 | GmARF7/19_E.1 | Glyma.07G130400.1 | A | II | ARF |
| Glyma.07G130400 | GmARF7/19_E.2 | Glyma.07G130400.2 | A | II | ARF |
| Glyma.07G130400 | GmARF7/19_E.3 | Glyma.07G130400.3 | A | II | ARF |
| Glyma.01G002100 | GmARF7/19_F.1 | Glyma.01G002100.1 | A | II | ARF |
| Glyma.01G002100 | GmARF7/19_F.2 | Glyma.01G002100.2 | A | II | ARF |
| Glyma.05G221300 | GmARF7/19_G.1 | Glyma.05G221300.1 | A | II | ARF |
| Glyma.05G221300 | GmARF7/19_G.2 | Glyma.05G221300.2 | A | II | ARF |
| Glyma.05G221300 | GmARF7/19_G.3 | Glyma.05G221300.3 | A | II | ARF |
| Glyma.08G027800 | GmARF7/19_H.1 | Glyma.08G027800.1 | A | II | ARF |
| Glyma.08G027800 | GmARF7/19_H.2 | Glyma.08G027800.2 | A | II | ARF |
| Glyma.11G204200 | GmARF8_A.1 | Glyma.11G204200.1 | A | II | ARF |
| Glyma.11G204200 | GmARF8_A.2 | Glyma.11G204200.2 | A | II | ARF |
| Glyma.18G046800 | GmARF8_B.1 | Glyma.18G046800.1 | A | II | ARF |
| Glyma.02G239600 | GmARF8_C.1 | Glyma.02G239600.1 | A | II | ARF |
| Glyma.02G239600 | GmARF8_C.2 | Glyma.02G239600.2 | A | II | ARF |
| Glyma.02G239600 | GmARF8_C.3 | Glyma.02G239600.3 | A | II | ARF |
| Glyma.02G239600 | GmARF8_C.4 | Glyma.02G239600.4 | A | II | ARF |
| Glyma.02G239600 | GmARF8_C.5 | Glyma.02G239600.5 | A | II | ARF |
| Glyma.14G208500 | GmARF8_D.1 | Glyma.14G208500.1 | A | II | ARF |
| Glyma.12G174100 | GmARF10/16_A.1 | Glyma.12G174100.1 | C | III | ARF |
| Glyma.13G325200 | GmARF10/16_B.1 | Glyma.13G325200.1 | C | III | ARF |
| Glyma.12G076200 | GmARF10/16_C.1 | Glyma.12G076200.1 | C | III | ARF |
| Glyma.11G145500 | GmARF10/16_D.1 | Glyma.11G145500.1 | C | III | ARF |
| Glyma.10G210600 | GmARF10/16_E.1 | Glyma.10G210600.1 | C | III | ARF |
| Glyma.20G180000 | GmARF10/16_F.1 | Glyma.20G180000.1 | C | III | ARF |
| Glyma.10G053500 | GmARF17_A.1 | Glyma.10G053500.1 | C | III | ARF |
| Glyma.13G140600 | GmARF17_B.1 | Glyma.13G140600.1 | C | III | ARF |
| Glyma.13G140600 | GmARF17_B.2 | Glyma.13G140600.2 | C | III | ARF |
| Glyma.19G181900 | GmARF17_C.2 | Glyma.19G181900.2 | C | III | ARF |
| Glyma.13G084700 | GmARF17_D.1 | Glyma.13G084700.1 | C | III | ARF |
| Glyma.04G254200 | GmARF17_F.1 | Glyma.04G254200.1 | C | III | ARF |
| Glyma.19G168500 | GmIAA12-13-A.1 | Glyma.19G168500.1 | A | I | IAA |
| Glyma.19G168500 | GmIAA12-13-A.2 | Glyma.19G168500.2 | A | I | IAA |
| Glyma.19G168500 | GmIAA12-13-A.3 | Glyma.19G168500.3 | A | I | IAA |
| Glyma.19G168500 | GmIAA12-13-A.4 | Glyma.19G168500.4 | A | I | IAA |
| Glyma.19G168500 | GmIAA12-13-A.5 | Glyma.19G168500.5 | A | I | IAA |
| Glyma.19G168500 | GmIAA12-13-A.6 | Glyma.19G168500.6 | A | I | IAA |
| Glyma.03G167400 | GmIAA12-13-B.10 | Glyma.03G167400.10 | A | I | IAA |
| Glyma.03G167400 | GmIAA12-13-B.11 | Glyma.03G167400.11 | A | I | IAA |
| Glyma.03G167400 | GmIAA12-13-B.9 | Glyma.03G167400.9 | A | I | IAA |
| Glyma.13G127000 | GmIAA12-13-C.3 | Glyma.13G127000.3 | A | I | IAA |
| Glyma.10G040400 | GmIAA12-13-D.3 | Glyma.10G040400.3 | A | I | IAA |
| Glyma.07G015200 | GmIAA18/26/28-A.1 | Glyma.07G015200.1 | A | I | IAA |
| Glyma.08G200700 | GmIAA18/26/28-B.1 | Glyma.08G200700.1 | A | I | IAA |
| Glyma.08G200700 | GmIAA18/26/28-B.2 | Glyma.08G200700.2 | A | I | IAA |
| Glyma.13G354100 | GmIAA18/26/28-C.11 | Glyma.13G354100.11 | A | I | IAA |
| Glyma.13G354100 | GmIAA18/26/28-C.6 | Glyma.13G354100.6 | A | I | IAA |
| Glyma.13G354100 | GmIAA18/26/28-C.8 | Glyma.13G354100.8 | A | I | IAA |
| Glyma.15G020300 | GmIAA18/26/28-D.1 | Glyma.15G020300.1 | A | I | IAA |
| Glyma.15G020300 | GmIAA18/26/28-D.2 | Glyma.15G020300.2 | A | I | IAA |
| Glyma.05G229300 | GmIAA27-A.1 | Glyma.05G229300.1 | A | I | IAA |
| Glyma.01G039300 | GmIAA27-B.2 | Glyma.01G039300.2 | A | I | IAA |
| Glyma.08G036400 | GmIAA27-C.1 | Glyma.08G036400.1 | A | I | IAA |
| Glyma.08G036400 | GmIAA27-C.2 | Glyma.08G036400.2 | A | I | IAA |
| Glyma.09G193000 | GmIAA27-D.1 | Glyma.09G193000.1 | A | I | IAA |
| Glyma.13G356600 | GmIAA27-E.1 | Glyma.13G356600.1 | A | I | IAA |
| Glyma.15G017500 | GmIAA27-F.1 | Glyma.15G017500.1 | A | I | IAA |
| Glyma.15G017500 | GmIAA27-F.2 | Glyma.15G017500.2 | A | I | IAA |
| Glyma.15G017500 | GmIAA27-F.3 | Glyma.15G017500.3 | A | I | IAA |
| Glyma.07G018100 | GmIAA27-G.1 | Glyma.07G018100.1 | A | I | IAA |
| Glyma.08G203100 | GmIAA27-H.1 | Glyma.08G203100.1 | A | I | IAA |
| Glyma.08G203100 | GmIAA27-H.2 | Glyma.08G203100.2 | A | I | IAA |
| Glyma.06G067700 | GmIAA29-A.1 | Glyma.06G067700.1 | A | I | IAA |
| Glyma.04G066300 | GmIAA29-B.1 | Glyma.04G066300.1 | A | I | IAA |
| Glyma.13G159000 | GmIAA29-C.1 | Glyma.13G159000.1 | A | I | IAA |
| Glyma.17G112300 | GmIAA29-D.1 | Glyma.17G112300.1 | A | I | IAA |
| Glyma.10G270500 | GmIAA32/34-A.1 | Glyma.10G270500.1 | A | I | IAA |
| Glyma.20G120800 | GmIAA32/34-B.1 | Glyma.20G120800.1 | A | I | IAA |
| Glyma.09G203300 | GmIAA8-9-A.1 | Glyma.09G203300.1 | A | I | IAA |
| Glyma.09G203300 | GmIAA8-9-A.2 | Glyma.09G203300.2 | A | I | IAA |
| Glyma.09G203300 | GmIAA8-9-A.3 | Glyma.09G203300.3 | A | I | IAA |
| Glyma.01G019400 | GmIAA8-9-B.1 | Glyma.01G019400.1 | A | I | IAA |
| Glyma.01G019400 | GmIAA8-9-B.2 | Glyma.01G019400.2 | A | I | IAA |
| Glyma.01G019400 | GmIAA8-9-B.3 | Glyma.01G019400.3 | A | I | IAA |
| Glyma.01G019400 | GmIAA8-9-B.4 | Glyma.01G019400.4 | A | I | IAA |
| Glyma.08G273500 | GmIAA8-9-C.1 | Glyma.08G273500.1 | A | I | IAA |
| Glyma.08G273500 | GmIAA8-9-C.2 | Glyma.08G273500.2 | A | I | IAA |
| Glyma.01G098000 | GmIAA8-9-D.3 | Glyma.01G098000.3 | A | I | IAA |
| Glyma.06G091700 | GmIAA8-9-E.3 | Glyma.06G091700.3 | A | I | IAA |
| Glyma.04G089900 | GmIAA8-9-F.1 | Glyma.04G089900.1 | A | I | IAA |
| Glyma.14G185400 | GmIAA8-9-G.1 | Glyma.14G185400.1 | A | I | IAA |
| Glyma.14G185400 | GmIAA8-9-G.2 | Glyma.14G185400.2 | A | I | IAA |
| Glyma.02G218100 | GmIAA8-9-H.2 | Glyma.02G218100.2 | A | I | IAA |
| Glyma.02G218100 | GmIAA8-9-H.4 | Glyma.02G218100.4 | A | I | IAA |
| Glyma.10G180000 | GmIAA1-4-A.1 | Glyma.10G180000.1 | B | II | IAA |
| Glyma.10G180000 | GmIAA1-4-A.2 | Glyma.10G180000.2 | B | II | IAA |
| Glyma.20G210500 | GmIAA1-4-B.1 | Glyma.20G210500.1 | B | II | IAA |
| Glyma.10G000700 | GmIAA1-4-C.2 | Glyma.10G000700.2 | B | II | IAA |
| Glyma.02G000500 | GmIAA1-4-D.1 | Glyma.02G000500.1 | B | II | IAA |
| Glyma.19G161000 | GmIAA1-4-E.1 | Glyma.19G161000.1 | B | II | IAA |
| Glyma.19G161000 | GmIAA1-4-E.2 | Glyma.19G161000.2 | B | II | IAA |
| Glyma.19G161000 | GmIAA1-4-E.3 | Glyma.19G161000.3 | B | II | IAA |
| Glyma.03G158600 | GmIAA1-4-F.1 | Glyma.03G158600.1 | B | II | IAA |
| Glyma.10G031800 | GmIAA1-4-G.3 | Glyma.10G031800.3 | B | II | IAA |
| Glyma.02G142600 | GmIAA1-4-H.1 | Glyma.02G142600.1 | B | II | IAA |
| Glyma.02G142600 | GmIAA1-4-H.2 | Glyma.02G142600.2 | B | II | IAA |
| Glyma.17G042800 | GmIAA31-A.1 | Glyma.17G042800.1 | B | II | IAA |
| Glyma.17G042800 | GmIAA31-A.2 | Glyma.17G042800.2 | B | II | IAA |
| Glyma.13G117100 | GmIAA31-B.1 | Glyma.13G117100.1 | B | II | IAA |
| Glyma.13G117100 | GmIAA31-B.2 | Glyma.13G117100.2 | B | II | IAA |
| Glyma.13G117100 | GmIAA31-B.3 | Glyma.13G117100.3 | B | II | IAA |
| Glyma.13G117100 | GmIAA31-B.4 | Glyma.13G117100.4 | B | II | IAA |
| Glyma.19G221900 | GmIAA31-C.1 | Glyma.19G221900.1 | B | II | IAA |
| Glyma.19G221900 | GmIAA31-C.2 | Glyma.19G221900.2 | B | II | IAA |
| Glyma.03G224800 | GmIAA31-D.1 | Glyma.03G224800.1 | B | II | IAA |
| Glyma.10G138500 | GmIAA31-E.1 | Glyma.10G138500.1 | B | II | IAA |
| Glyma.02G007300 | GmIAA31-F.1 | Glyma.02G007300.1 | B | II | IAA |
| Glyma.13G361100 | GmIAA6/19-A.1 | Glyma.13G361100.1 | B | II | IAA |
| Glyma.15G012800 | GmIAA6/19-B.1 | Glyma.15G012800.1 | B | II | IAA |
| Glyma.07G034200 | GmIAA6/19-C.1 | Glyma.07G034200.1 | B | II | IAA |
| Glyma.13G361200 | GmIAA15-A.2 | Glyma.13G361200.2 | C | III | IAA |
| Glyma.13G361200 | GmIAA15-A.3 | Glyma.13G361200.3 | C | III | IAA |
| Glyma.13G361200 | GmIAA15-A.4 | Glyma.13G361200.4 | C | III | IAA |
| Glyma.13G361200 | GmIAA15-A.5 | Glyma.13G361200.5 | C | III | IAA |
| Glyma.15G012700 | GmIAA15-B.1 | Glyma.15G012700.1 | C | III | IAA |
| Glyma.15G012700 | GmIAA15-B.2 | Glyma.15G012700.2 | C | III | IAA |
| Glyma.15G012700 | GmIAA15-B.3 | Glyma.15G012700.3 | C | III | IAA |
| Glyma.15G012700 | GmIAA15-B.4 | Glyma.15G012700.4 | C | III | IAA |
| Glyma.15G012700 | GmIAA15-B.5 | Glyma.15G012700.5 | C | III | IAA |
| Glyma.10G162400 | GmIAA16-A.1 | Glyma.10G162400.1 | C | III | IAA |
| Glyma.10G162400 | GmIAA16-A.2 | Glyma.10G162400.2 | C | III | IAA |
| Glyma.10G162400 | GmIAA16-A.3 | Glyma.10G162400.3 | C | III | IAA |
| Glyma.20G225000 | GmIAA16-B.1 | Glyma.20G225000.1 | C | III | IAA |
| Glyma.03G247400 | GmIAA16-C.1 | Glyma.03G247400.1 | C | III | IAA |
| Glyma.19G245200 | GmIAA16-D.1 | Glyma.19G245200.1 | C | III | IAA |
| Glyma.10G031900 | GmIAA16-E.1 | Glyma.10G031900.1 | C | III | IAA |
| Glyma.10G031900 | GmIAA16-E.2 | Glyma.10G031900.2 | C | III | IAA |
| Glyma.02G142500 | GmIAA16-F.3 | Glyma.02G142500.3 | C | III | IAA |
| Glyma.19G161100 | GmIAA16-G.1 | Glyma.19G161100.1 | C | III | IAA |
| Glyma.03G158700 | GmIAA16-H.1 | Glyma.03G158700.1 | C | III | IAA |
| Glyma.10G180100 | GmIAA7/14/17-A.1 | Glyma.10G180100.1 | C | III | IAA |
| Glyma.20G210400 | GmIAA7/14/17-B.1 | Glyma.20G210400.1 | C | III | IAA |

Table S2. Tissue specificity index (tau) analysis. The relative expression for each transcript in each tissue are presented as fraction of maximum expression value, $\hat{x}_{i}.$Abbreviations correspond to axillary meristem (AM), open flower (OF), inflorescence before and after meiosis (IBM and IAM, respectively), root tip (RT), cotyledon (Ct), hypocotyl (Hp), shoot apical meristem at 6, 17, and 38 days (SAM6D, SAM17D, and SAM38D, respectively), and nodule (nd). All Orthology names are for Glycine max as proposed previously in Table S1.

| **Orthology** | **AM** | **OF** | **IAM** | **IBM** | **RT** | **Ct** | **Hp** | **SAM6D** | **SAM17D** | **SAM38D** | **Callus** | **Leaf** | **Root** | **Nd** | **tau** |
| --- | --- | --- | --- | --- | --- | --- | --- | --- | --- | --- | --- | --- | --- | --- | --- |
| ARF7/19_F.1 | 0.82 | 0.89 | 0.80 | 0.94 | 0.49 | 0.50 | 1.00 | 0.72 | 0.65 | 0.46 | 0.74 | 0.39 | 0.55 | 0.15 | 0.38 |
| IAA8-9-B.1 | 0.38 | 0.72 | 0.00 | 0.14 | 0.35 | 0.10 | 0.84 | 0.28 | 0.75 | 0.33 | 0.57 | 1.00 | 0.50 | 0.05 | 0.61 |
| IAA8-9-B.2 | 0.17 | 0.00 | 0.27 | 0.34 | 0.10 | 0.09 | 0.50 | 0.42 | 0.00 | 0.11 | 0.04 | 0.52 | 1.00 | 0.08 | 0.80 |
| IAA8-9-B.3 | 0.25 | 0.40 | 0.23 | 0.30 | 0.18 | 0.05 | 1.00 | 0.28 | 0.30 | 0.33 | 0.20 | 0.07 | 0.00 | 0.00 | 0.80 |
| IAA8-9-D.3 | 0.40 | 0.30 | 0.31 | 0.56 | 0.19 | 0.45 | 1.00 | 0.59 | 0.63 | 0.33 | 0.02 | 0.42 | 0.31 | 0.13 | 0.64 |
| ARF9_A.1 | 0.69 | 0.21 | 0.42 | 0.84 | 0.15 | 0.06 | 0.07 | 0.79 | 1.00 | 0.49 | 0.03 | 0.00 | 0.00 | 0.00 | 0.71 |
| IAA1-4-D.1 | 0.25 | 0.32 | 0.14 | 0.11 | 0.27 | 0.06 | 1.00 | 0.22 | 0.21 | 0.41 | 0.01 | 0.07 | 0.11 | 0.01 | 0.83 |
| AFB2/3_C.1 | 0.52 | 0.42 | 0.51 | 0.77 | 0.25 | 0.08 | 0.30 | 0.86 | 1.00 | 0.43 | 0.30 | 0.15 | 0.20 | 0.15 | 0.62 |
| IAA16-F.3 | 0.03 | 1.00 | 0.14 | 0.05 | 0.08 | 0.00 | 0.22 | 0.01 | 0.01 | 0.01 | 0.00 | 0.04 | 0.13 | 0.00 | 0.94 |
| IAA1-4-H.1 | 0.05 | 1.00 | 0.24 | 0.27 | 0.24 | 0.07 | 0.17 | 0.04 | 0.06 | 0.02 | 0.01 | 0.09 | 0.03 | 0.08 | 0.89 |
| IAA1-4-H.2 | 0.06 | 1.00 | 0.48 | 0.27 | 0.19 | 0.07 | 0.23 | 0.00 | 0.04 | 0.01 | 0.00 | 0.00 | 0.00 | 0.00 | 0.90 |
| TIR1/AFB1_D.2 | 0.65 | 0.48 | 0.62 | 0.67 | 0.36 | 0.45 | 0.60 | 0.87 | 1.00 | 0.51 | 0.53 | 0.00 | 0.00 | 0.00 | 0.56 |
| AFB4/5_D.1 | 0.69 | 0.46 | 0.41 | 0.62 | 0.46 | 0.25 | 0.83 | 0.59 | 0.66 | 0.34 | 0.38 | 0.38 | 1.00 | 0.23 | 0.52 |
| IAA8-9-H.2 | 0.28 | 0.52 | 0.15 | 0.31 | 0.29 | 0.10 | 0.31 | 0.66 | 0.72 | 0.35 | 0.04 | 0.27 | 1.00 | 0.09 | 0.69 |
| IAA8-9-H.4 | 0.64 | 1.00 | 0.60 | 0.55 | 0.28 | 0.09 | 0.56 | 0.87 | 0.60 | 0.47 | 0.07 | 0.00 | 0.00 | 0.00 | 0.63 |
| ARF8_C.1 | 0.72 | 0.32 | 0.88 | 1.00 | 0.26 | 0.68 | 0.65 | 0.90 | 0.50 | 0.41 | 0.11 | 0.85 | 0.29 | 0.03 | 0.49 |
| ARF8_C.3 | 0.54 | 0.08 | 0.61 | 0.75 | 0.09 | 0.00 | 0.35 | 0.77 | 1.00 | 0.39 | 0.09 | 0.00 | 0.05 | 0.00 | 0.71 |
| ARF8_C.5 | 0.41 | 0.41 | 0.26 | 0.22 | 0.18 | 0.42 | 1.00 | 0.00 | 0.35 | 0.40 | 0.05 | 0.00 | 0.00 | 0.00 | 0.79 |
| ARF6_E.1 | 0.81 | 0.52 | 0.64 | 0.84 | 0.28 | 0.42 | 0.69 | 0.63 | 1.00 | 0.57 | 0.39 | 0.41 | 0.16 | 0.13 | 0.50 |
| ARF9_B.1 | 0.12 | 0.07 | 0.08 | 0.14 | 0.05 | 0.05 | 0.03 | 0.17 | 0.11 | 0.08 | 0.02 | 0.34 | 1.00 | 0.22 | 0.89 |
| ARF9_B.2 | 0.81 | 0.58 | 0.73 | 0.76 | 0.15 | 0.15 | 0.13 | 0.85 | 1.00 | 0.68 | 0.01 | 0.15 | 0.78 | 0.06 | 0.55 |
| IAA1-4-F.1 | 0.06 | 1.00 | 0.23 | 0.09 | 0.13 | 0.00 | 0.44 | 0.02 | 0.04 | 0.03 | 0.00 | 0.06 | 0.08 | 0.00 | 0.91 |
| IAA16-H.1 | 0.16 | 0.49 | 0.17 | 0.13 | 0.25 | 0.03 | 1.00 | 0.05 | 0.10 | 0.19 | 0.00 | 0.02 | 0.04 | 0.01 | 0.87 |
| TIR1/AFB1_B.1 | 0.91 | 0.93 | 0.50 | 0.73 | 0.33 | 0.79 | 0.73 | 1.00 | 0.88 | 0.72 | 0.41 | 0.94 | 0.19 | 0.04 | 0.38 |
| IAA16-C.1 | 0.57 | 0.84 | 0.67 | 0.63 | 0.32 | 0.47 | 1.00 | 0.35 | 0.50 | 0.40 | 0.30 | 0.83 | 0.45 | 0.03 | 0.51 |
| ARF11/18_C.1 | 0.24 | 0.18 | 0.13 | 0.24 | 0.05 | 0.61 | 0.43 | 0.23 | 0.37 | 0.18 | 0.25 | 1.00 | 0.78 | 0.27 | 0.69 |
| IAA8-9-F.1 | 0.39 | 0.26 | 0.37 | 0.49 | 0.14 | 0.32 | 1.00 | 0.52 | 0.72 | 0.39 | 0.25 | 0.24 | 0.34 | 0.15 | 0.65 |
| AFB4/5_B.1 | 0.81 | 0.76 | 0.50 | 0.72 | 0.17 | 0.19 | 0.65 | 0.82 | 1.00 | 0.59 | 0.33 | 0.21 | 0.39 | 0.05 | 0.52 |
| ARF2_B.1 | 0.57 | 0.33 | 0.52 | 1.00 | 0.06 | 0.14 | 0.13 | 0.38 | 0.65 | 0.32 | 0.05 | 0.22 | 0.12 | 0.10 | 0.72 |
| ARF2_B.2 | 0.39 | 0.24 | 0.56 | 0.19 | 0.00 | 0.02 | 0.07 | 0.00 | 1.00 | 0.29 | 0.11 | 0.00 | 0.07 | 0.02 | 0.85 |
| ARF17_F.1 | 0.81 | 0.23 | 0.62 | 1.00 | 0.22 | 0.26 | 0.45 | 0.69 | 0.89 | 0.74 | 0.30 | 0.43 | 0.12 | 0.26 | 0.54 |
| ARF2_C.1 | 0.54 | 0.17 | 0.30 | 0.76 | 0.10 | 0.49 | 0.48 | 0.72 | 0.71 | 0.37 | 0.10 | 1.00 | 0.58 | 0.48 | 0.55 |
| ARF2_C.4 | 0.37 | 0.14 | 0.31 | 0.31 | 0.01 | 0.09 | 0.13 | 0.26 | 1.00 | 0.35 | 0.09 | 0.00 | 0.00 | 0.00 | 0.84 |
| ARF7/19_G.2 | 0.18 | 0.35 | 0.24 | 0.27 | 0.02 | 0.15 | 0.11 | 0.00 | 0.42 | 0.16 | 1.00 | 0.00 | 0.00 | 0.00 | 0.85 |
| IAA27-A.1 | 0.56 | 0.26 | 0.65 | 0.51 | 0.81 | 0.06 | 1.00 | 0.36 | 0.37 | 0.53 | 0.06 | 0.45 | 0.34 | 0.11 | 0.61 |
| IAA8-9-E.3 | 0.31 | 0.19 | 0.21 | 0.29 | 0.04 | 0.31 | 0.30 | 0.58 | 1.00 | 0.46 | 0.18 | 0.12 | 0.05 | 0.06 | 0.76 |
| AFB4/5_A.1 | 0.75 | 0.72 | 0.70 | 0.59 | 0.17 | 0.44 | 0.78 | 0.79 | 1.00 | 0.50 | 0.58 | 0.37 | 0.36 | 0.33 | 0.46 |
| ARF2_A.2 | 0.60 | 0.30 | 0.54 | 1.00 | 0.02 | 0.14 | 0.13 | 0.46 | 0.49 | 0.30 | 0.08 | 0.26 | 0.14 | 0.09 | 0.73 |
| ARF2_A.3 | 0.33 | 0.38 | 0.43 | 0.40 | 0.01 | 0.12 | 0.08 | 0.16 | 1.00 | 0.32 | 0.03 | 0.02 | 0.01 | 0.00 | 0.82 |
| IAA6/19-C.1 | 0.05 | 0.16 | 0.15 | 0.08 | 0.03 | 0.01 | 1.00 | 0.01 | 0.01 | 0.01 | 0.00 | 0.02 | 0.04 | 0.00 | 0.96 |
| ARF11/18_B.1 | 0.58 | 0.10 | 0.21 | 0.74 | 0.26 | 0.26 | 0.45 | 1.00 | 0.88 | 0.54 | 0.03 | 0.53 | 0.16 | 0.06 | 0.63 |
| ARF11/18_B.4 | 0.43 | 0.36 | 0.28 | 0.28 | 0.03 | 0.00 | 0.40 | 1.00 | 0.47 | 0.40 | 0.00 | 0.05 | 0.00 | 0.00 | 0.79 |
| ARF7/19_E.1 | 0.72 | 0.34 | 0.62 | 0.72 | 0.61 | 0.89 | 0.64 | 0.65 | 0.83 | 0.41 | 0.93 | 0.12 | 1.00 | 0.30 | 0.40 |
| ARF9_C.1 | 0.64 | 0.35 | 0.27 | 0.61 | 0.09 | 0.28 | 0.18 | 0.61 | 1.00 | 0.42 | 0.03 | 0.20 | 0.52 | 0.07 | 0.67 |
| AFB6_A.4 | 0.39 | 1.00 | 0.36 | 0.10 | 0.08 | 0.19 | 0.57 | 0.14 | 0.68 | 0.37 | 0.86 | 0.00 | 0.00 | 0.00 | 0.71 |
| AFB6_A.5 | 0.84 | 0.63 | 0.00 | 1.00 | 0.35 | 0.49 | 0.47 | 0.92 | 0.88 | 0.33 | 0.40 | 0.00 | 0.00 | 0.20 | 0.58 |
| ARF3_A.1 | 0.45 | 0.54 | 0.32 | 0.52 | 0.07 | 0.21 | 0.54 | 1.00 | 0.69 | 0.38 | 0.06 | 0.39 | 0.13 | 0.40 | 0.64 |
| ARF1_C.1 | 0.59 | 0.31 | 0.45 | 0.72 | 0.13 | 0.39 | 0.31 | 0.58 | 1.00 | 0.41 | 0.22 | 0.34 | 0.42 | 0.26 | 0.60 |
| ARF2_D.2 | 0.40 | 0.22 | 0.47 | 0.52 | 0.02 | 0.14 | 0.26 | 0.44 | 1.00 | 0.30 | 0.03 | 0.46 | 0.26 | 0.08 | 0.72 |
| ARF2_D.3 | 0.76 | 0.29 | 0.42 | 1.00 | 0.08 | 0.51 | 0.34 | 1.00 | 0.48 | 0.56 | 0.11 | 0.00 | 0.00 | 0.00 | 0.65 |
| ARF7/19_H.1 | 0.18 | 1.00 | 0.16 | 0.24 | 0.03 | 0.35 | 0.17 | 0.14 | 0.19 | 0.09 | 0.20 | 0.00 | 0.22 | 0.07 | 0.84 |
| IAA27-C.1 | 0.81 | 0.55 | 0.66 | 0.66 | 0.51 | 0.13 | 0.94 | 0.69 | 0.64 | 1.00 | 0.08 | 0.47 | 0.54 | 0.26 | 0.47 |
| IAA27-C.2 | 0.17 | 0.26 | 0.00 | 0.15 | 0.52 | 0.00 | 0.78 | 0.10 | 1.00 | 0.57 | 0.01 | 0.00 | 0.24 | 0.00 | 0.78 |
| AFB6_B.6 | 0.42 | 0.97 | 0.30 | 0.58 | 0.28 | 0.15 | 1.00 | 0.40 | 0.51 | 0.24 | 0.55 | 0.22 | 0.22 | 0.02 | 0.63 |
| ARF6_A.2 | 0.50 | 0.46 | 0.43 | 0.68 | 0.09 | 0.16 | 0.29 | 0.61 | 1.00 | 0.34 | 0.05 | 0.39 | 0.75 | 0.24 | 0.62 |
| ARF6_A.3 | 0.60 | 0.73 | 0.61 | 0.88 | 0.10 | 0.05 | 0.42 | 0.58 | 1.00 | 0.45 | 0.22 | 0.04 | 0.00 | 0.00 | 0.64 |
| IAA18/26/28-B.2 | 0.05 | 1.00 | 0.15 | 0.18 | 0.03 | 0.01 | 0.05 | 0.03 | 0.03 | 0.04 | 0.00 | 0.00 | 0.00 | 0.00 | 0.95 |
| IAA8-9-C.1 | 0.80 | 0.31 | 0.78 | 0.94 | 0.22 | 0.23 | 1.00 | 0.75 | 0.61 | 0.45 | 0.10 | 0.74 | 0.57 | 0.09 | 0.49 |
| IAA8-9-C.2 | 0.36 | 0.26 | 0.38 | 0.42 | 0.09 | 0.15 | 1.00 | 0.47 | 0.54 | 0.38 | 0.04 | 0.07 | 0.04 | 0.01 | 0.75 |
| ARF7/19_A.1 | 0.35 | 0.53 | 0.17 | 0.36 | 0.20 | 0.31 | 0.80 | 1.00 | 0.97 | 0.31 | 0.70 | 0.21 | 0.38 | 0.07 | 0.59 |
| IAA27-D.1 | 0.24 | 0.11 | 0.17 | 0.23 | 0.03 | 0.13 | 1.00 | 0.26 | 0.40 | 0.43 | 0.02 | 0.13 | 0.35 | 0.00 | 0.81 |
| IAA8-9-A.1 | 0.18 | 0.19 | 0.18 | 0.18 | 0.11 | 0.31 | 1.00 | 0.24 | 0.14 | 0.16 | 0.09 | 0.28 | 0.23 | 0.06 | 0.82 |
| IAA8-9-A.3 | 0.31 | 0.32 | 0.33 | 0.37 | 0.10 | 0.00 | 1.00 | 0.26 | 0.35 | 0.26 | 0.05 | 0.00 | 0.26 | 0.05 | 0.80 |
| TIR1/AFB1_C.1 | 0.50 | 0.34 | 0.24 | 0.44 | 0.21 | 0.62 | 0.60 | 1.00 | 0.74 | 0.41 | 0.13 | 0.16 | 0.07 | 0.00 | 0.66 |
| TIR1/AFB1_C.2 | 0.76 | 0.62 | 0.69 | 0.71 | 0.39 | 0.56 | 0.56 | 0.47 | 0.85 | 0.59 | 0.98 | 1.00 | 0.22 | 0.12 | 0.42 |
| TIR1/AFB1_C.3 | 0.84 | 0.31 | 0.43 | 0.33 | 0.10 | 0.14 | 0.19 | 1.00 | 0.32 | 0.46 | 0.36 | 0.73 | 0.10 | 0.04 | 0.67 |
| IAA1-4-G.3 | 0.06 | 1.00 | 0.12 | 0.08 | 0.26 | 0.11 | 0.52 | 0.06 | 0.05 | 0.08 | 0.01 | 0.05 | 0.02 | 0.01 | 0.89 |
| IAA16-E.1 | 0.05 | 1.00 | 0.22 | 0.14 | 0.15 | 0.00 | 0.16 | 0.03 | 0.05 | 0.02 | 0.00 | 0.26 | 0.87 | 0.00 | 0.85 |
| IAA12-13-D.3 | 0.20 | 1.00 | 0.26 | 0.20 | 0.12 | 0.03 | 0.55 | 0.13 | 0.19 | 0.13 | 0.06 | 0.14 | 0.32 | 0.09 | 0.81 |
| ARF17_A.1 | 0.65 | 1.00 | 0.54 | 0.57 | 0.15 | 0.17 | 0.53 | 0.23 | 0.39 | 0.46 | 0.33 | 0.38 | 0.23 | 0.06 | 0.64 |
| IAA16-A.1 | 0.51 | 0.73 | 0.49 | 0.51 | 0.33 | 0.24 | 1.00 | 0.46 | 0.66 | 0.36 | 0.02 | 0.20 | 0.55 | 0.18 | 0.60 |
| IAA16-A.2 | 0.20 | 0.24 | 0.17 | 0.19 | 0.21 | 0.06 | 1.00 | 0.20 | 0.17 | 0.24 | 0.05 | 0.03 | 0.05 | 0.00 | 0.86 |
| IAA16-A.3 | 0.28 | 0.84 | 0.40 | 0.76 | 0.37 | 1.00 | 0.84 | 0.84 | 0.42 | 0.51 | 0.14 | 0.35 | 0.04 | 0.00 | 0.56 |
| IAA1-4-A.1 | 0.10 | 0.28 | 0.07 | 0.09 | 1.00 | 0.02 | 0.40 | 0.05 | 0.04 | 0.08 | 0.00 | 0.13 | 0.22 | 0.02 | 0.88 |
| IAA1-4-A.2 | 0.23 | 1.00 | 0.08 | 0.26 | 0.76 | 0.20 | 0.76 | 0.00 | 0.00 | 0.15 | 0.00 | 0.09 | 0.14 | 0.00 | 0.79 |
| IAA7/14/17-A.1 | 0.01 | 1.00 | 0.01 | 0.01 | 0.40 | 0.06 | 0.65 | 0.00 | 0.01 | 0.01 | 0.01 | 0.04 | 0.18 | 0.01 | 0.89 |
| ARF10/16_E.1 | 0.83 | 0.37 | 0.50 | 0.62 | 1.00 | 0.09 | 0.41 | 0.79 | 0.80 | 0.54 | 0.04 | 0.16 | 0.49 | 0.41 | 0.54 |
| ARF10/16_D.1 | 0.52 | 0.56 | 0.41 | 0.41 | 0.22 | 1.00 | 0.28 | 0.31 | 0.33 | 0.34 | 0.02 | 0.13 | 0.09 | 0.02 | 0.72 |
| ARF8_A.1 | 0.91 | 1.00 | 0.00 | 0.14 | 0.15 | 0.00 | 0.27 | 0.00 | 0.00 | 0.84 | 0.00 | 0.88 | 0.17 | 0.06 | 0.74 |
| ARF8_A.2 | 0.30 | 0.00 | 0.79 | 1.00 | 0.10 | 0.23 | 0.72 | 0.73 | 0.92 | 0.00 | 0.39 | 0.00 | 0.00 | 0.00 | 0.68 |
| ARF4_A.1 | 0.66 | 0.00 | 0.48 | 0.90 | 0.00 | 0.00 | 0.21 | 0.63 | 1.00 | 0.65 | 0.00 | 0.71 | 0.00 | 0.00 | 0.67 |
| ARF4_A.2 | 0.74 | 1.00 | 0.21 | 0.53 | 0.04 | 0.38 | 0.79 | 0.02 | 0.00 | 0.18 | 0.28 | 0.00 | 0.78 | 0.31 | 0.67 |
| ARF10/16_C.1 | 1.00 | 0.44 | 0.71 | 0.77 | 0.17 | 0.14 | 0.25 | 0.34 | 0.35 | 0.65 | 0.04 | 0.18 | 0.10 | 0.07 | 0.68 |
| ARF1_A.1 | 0.58 | 0.50 | 0.42 | 0.66 | 0.24 | 0.30 | 0.43 | 0.53 | 0.74 | 0.42 | 1.00 | 0.37 | 0.43 | 0.14 | 0.56 |
| ARF4_C.9 | 0.37 | 0.18 | 0.08 | 0.39 | 0.00 | 0.34 | 0.17 | 0.91 | 1.00 | 0.44 | 0.11 | 0.00 | 0.00 | 0.00 | 0.77 |
| ARF10/16_A.1 | 0.56 | 0.66 | 0.75 | 1.00 | 0.31 | 0.83 | 0.79 | 0.30 | 0.49 | 0.36 | 0.12 | 0.22 | 0.35 | 0.10 | 0.55 |
| ARF17_D.1 | 0.69 | 0.32 | 0.65 | 1.00 | 0.23 | 0.40 | 0.53 | 0.62 | 0.58 | 0.57 | 0.48 | 0.36 | 0.21 | 0.37 | 0.54 |
| ARF7/19_D.1 | 0.16 | 1.00 | 0.09 | 0.16 | 0.05 | 0.13 | 0.50 | 0.21 | 0.21 | 0.11 | 0.24 | 0.10 | 0.10 | 0.08 | 0.83 |
| IAA12-13-C.3 | 0.34 | 1.00 | 0.18 | 0.31 | 0.17 | 0.29 | 0.78 | 0.36 | 0.36 | 0.30 | 0.00 | 0.22 | 1.00 | 0.37 | 0.64 |
| ARF17_B.2 | 0.82 | 0.41 | 0.37 | 0.52 | 0.31 | 0.35 | 0.50 | 0.58 | 1.00 | 0.45 | 0.24 | 0.28 | 0.09 | 0.00 | 0.62 |
| ARF3_B.1 | 0.44 | 0.14 | 0.45 | 0.52 | 0.03 | 0.03 | 0.60 | 0.44 | 1.00 | 0.34 | 0.02 | 0.03 | 0.03 | 0.01 | 0.76 |
| ARF6_C.2 | 0.75 | 0.39 | 0.59 | 0.94 | 0.04 | 0.03 | 0.62 | 0.48 | 1.00 | 0.44 | 0.06 | 0.43 | 0.09 | 0.03 | 0.62 |
| ARF3_C.1 | 0.79 | 0.66 | 0.60 | 1.00 | 0.09 | 0.24 | 0.66 | 0.75 | 0.88 | 0.59 | 0.13 | 0.52 | 0.19 | 0.22 | 0.51 |
| ARF10/16_B.1 | 0.71 | 1.00 | 0.76 | 0.90 | 0.15 | 0.13 | 0.17 | 0.49 | 0.93 | 0.59 | 0.08 | 0.11 | 0.21 | 0.14 | 0.59 |
| IAA18/26/28-C.11 | 0.33 | 0.59 | 0.59 | 1.00 | 0.03 | 0.03 | 0.11 | 0.90 | 0.74 | 0.68 | 0.00 | 0.00 | 0.00 | 0.12 | 0.68 |
| IAA18/26/28-C.6 | 0.34 | 0.27 | 0.43 | 0.57 | 0.12 | 0.11 | 0.18 | 0.68 | 1.00 | 0.35 | 0.13 | 0.07 | 0.00 | 0.02 | 0.75 |
| IAA18/26/28-C.8 | 0.62 | 0.35 | 0.59 | 0.97 | 0.17 | 0.30 | 0.16 | 0.77 | 0.39 | 0.20 | 0.15 | 1.00 | 0.64 | 0.00 | 0.59 |
| IAA27-E.1 | 0.52 | 0.82 | 1.00 | 0.47 | 0.96 | 0.06 | 0.73 | 0.15 | 0.21 | 0.48 | 0.00 | 0.03 | 0.91 | 0.11 | 0.58 |
| IAA6/19-A.1 | 0.08 | 0.63 | 0.09 | 0.11 | 0.44 | 0.06 | 1.00 | 0.04 | 0.05 | 0.09 | 0.00 | 0.11 | 0.15 | 0.01 | 0.86 |
| IAA15-A.2 | 0.37 | 1.00 | 0.75 | 0.67 | 0.03 | 0.01 | 0.65 | 0.05 | 0.20 | 0.24 | 0.00 | 0.03 | 0.00 | 0.00 | 0.77 |
| IAA15-A.3 | 0.11 | 0.71 | 1.00 | 0.64 | 0.00 | 0.00 | 0.70 | 0.08 | 0.12 | 0.15 | 0.01 | 0.11 | 0.00 | 0.00 | 0.80 |
| ARF6_F.1 | 0.79 | 0.44 | 0.61 | 1.00 | 0.30 | 0.45 | 0.52 | 0.36 | 0.39 | 0.29 | 0.59 | 0.34 | 0.37 | 0.13 | 0.57 |
| ARF6_F.2 | 0.67 | 0.47 | 0.55 | 0.67 | 0.23 | 0.47 | 0.71 | 0.55 | 1.00 | 0.49 | 0.39 | 0.07 | 0.11 | 0.02 | 0.58 |
| AFB4/5_C.1 | 0.95 | 0.94 | 0.70 | 0.76 | 0.49 | 0.37 | 0.77 | 1.00 | 0.86 | 0.44 | 0.55 | 0.84 | 0.63 | 0.15 | 0.35 |
| IAA8-9-G.1 | 0.57 | 1.00 | 0.54 | 0.66 | 0.41 | 0.38 | 0.88 | 0.82 | 0.88 | 0.56 | 0.04 | 0.21 | 0.87 | 0.08 | 0.47 |
| ARF8_D.1 | 0.74 | 0.41 | 0.77 | 1.00 | 0.24 | 0.11 | 0.54 | 0.71 | 0.94 | 0.45 | 0.20 | 0.27 | 0.14 | 0.02 | 0.57 |
| ARF5_A.1 | 0.27 | 0.12 | 0.20 | 0.42 | 0.02 | 0.07 | 0.05 | 0.70 | 1.00 | 0.31 | 0.37 | 0.08 | 0.09 | 0.00 | 0.79 |
| IAA27-F.1 | 0.20 | 0.33 | 0.28 | 0.16 | 0.28 | 0.09 | 1.00 | 0.11 | 0.12 | 0.15 | 0.06 | 0.06 | 0.15 | 0.03 | 0.84 |
| IAA27-F.3 | 0.54 | 1.00 | 0.22 | 0.51 | 0.00 | 0.00 | 0.00 | 0.00 | 0.37 | 0.58 | 0.00 | 0.33 | 0.40 | 0.00 | 0.77 |
| IAA18/26/28-D.1 | 0.56 | 0.26 | 0.37 | 0.63 | 0.04 | 0.40 | 0.30 | 0.60 | 1.00 | 0.41 | 0.16 | 0.24 | 0.13 | 0.00 | 0.68 |
| ARF6_D.1 | 0.77 | 0.44 | 1.00 | 0.97 | 0.03 | 0.08 | 0.32 | 0.35 | 0.78 | 0.44 | 0.09 | 0.09 | 0.12 | 0.01 | 0.65 |
| ARF6_D.4 | 0.33 | 0.40 | 0.63 | 1.00 | 0.03 | 0.11 | 0.47 | 0.47 | 0.00 | 0.27 | 0.08 | 0.00 | 0.00 | 0.00 | 0.79 |
| ARF13_A.2 | 0.75 | 0.39 | 0.65 | 0.90 | 0.19 | 0.77 | 0.45 | 0.98 | 1.00 | 0.35 | 0.56 | 0.67 | 0.00 | 0.38 | 0.46 |
| ARF7/19_B.2 | 0.66 | 0.87 | 0.35 | 0.78 | 0.31 | 0.12 | 0.63 | 0.87 | 1.00 | 0.43 | 0.76 | 0.53 | 0.44 | 0.26 | 0.46 |
| ARF1_B.1 | 0.63 | 0.49 | 0.58 | 0.78 | 0.40 | 0.40 | 0.65 | 0.82 | 1.00 | 0.50 | 0.79 | 0.52 | 0.45 | 0.18 | 0.45 |
| ARF11/18_A.2 | 0.37 | 0.16 | 0.32 | 0.38 | 0.03 | 0.10 | 0.01 | 0.89 | 1.00 | 0.38 | 0.00 | 0.41 | 0.13 | 0.02 | 0.75 |
| AFB2/3_B.1 | 0.63 | 0.40 | 0.37 | 0.67 | 0.15 | 0.32 | 0.31 | 0.93 | 1.00 | 0.44 | 0.54 | 0.37 | 0.20 | 0.10 | 0.58 |
| ARF7/19_C.1 | 0.40 | 1.00 | 0.24 | 0.58 | 0.28 | 0.33 | 0.75 | 0.78 | 0.92 | 0.38 | 0.28 | 0.20 | 0.26 | 0.02 | 0.58 |
| IAA29-D.1 | 0.34 | 1.00 | 0.14 | 0.20 | 0.06 | 0.06 | 0.99 | 0.35 | 0.72 | 0.48 | 0.00 | 0.12 | 0.00 | 0.04 | 0.73 |
| ARF5_B.1 | 0.11 | 0.04 | 0.05 | 0.26 | 0.00 | 0.02 | 0.02 | 0.41 | 0.35 | 0.10 | 1.00 | 0.04 | 0.06 | 0.01 | 0.89 |
| ARF8_B.1 | 0.71 | 0.48 | 0.61 | 0.82 | 0.20 | 0.11 | 0.63 | 0.68 | 1.00 | 0.44 | 0.34 | 0.17 | 0.22 | 0.03 | 0.58 |
| ARF9_D.1 | 0.40 | 0.54 | 0.33 | 0.69 | 0.08 | 0.02 | 0.30 | 0.65 | 1.00 | 0.45 | 0.33 | 0.00 | 0.98 | 0.00 | 0.63 |
| AFB2/3_A.1 | 0.56 | 0.57 | 0.36 | 0.57 | 0.18 | 0.17 | 0.26 | 0.81 | 1.00 | 0.42 | 0.59 | 0.36 | 0.29 | 0.10 | 0.60 |
| IAA1-4-E.1 | 0.03 | 1.00 | 0.19 | 0.11 | 0.56 | 0.13 | 0.74 | 0.01 | 0.01 | 0.00 | 0.01 | 0.04 | 0.13 | 0.03 | 0.85 |
| IAA1-4-E.3 | 0.01 | 1.00 | 0.11 | 0.04 | 0.18 | 0.10 | 0.44 | 0.01 | 0.01 | 0.01 | 0.00 | 0.02 | 0.02 | 0.00 | 0.93 |
| IAA16-G.1 | 0.02 | 0.32 | 0.09 | 0.03 | 0.13 | 0.01 | 1.00 | 0.00 | 0.00 | 0.01 | 0.00 | 0.00 | 0.10 | 0.05 | 0.94 |
| ARF17_C.2 | 0.74 | 1.00 | 0.61 | 0.68 | 0.11 | 0.45 | 0.53 | 0.32 | 0.57 | 0.53 | 0.29 | 0.21 | 0.28 | 0.21 | 0.57 |
| TIR1/AFB1_A.1 | 1.00 | 0.47 | 0.77 | 0.91 | 0.22 | 0.26 | 0.45 | 0.70 | 0.83 | 0.64 | 0.46 | 0.86 | 0.20 | 0.11 | 0.47 |
| IAA16-D.1 | 0.39 | 1.00 | 0.45 | 0.37 | 0.06 | 0.29 | 0.99 | 0.17 | 0.20 | 0.32 | 0.59 | 0.09 | 0.06 | 0.01 | 0.69 |
| ARF10/16_F.1 | 0.76 | 0.30 | 0.32 | 0.59 | 0.65 | 0.20 | 0.38 | 1.00 | 0.78 | 0.61 | 0.02 | 0.27 | 0.33 | 0.11 | 0.59 |
| IAA7/14/17-B.1 | 0.01 | 0.42 | 0.02 | 0.02 | 0.21 | 0.07 | 1.00 | 0.00 | 0.00 | 0.00 | 0.00 | 0.00 | 0.10 | 0.00 | 0.93 |
| IAA1-4-B.1 | 0.24 | 0.23 | 0.18 | 0.28 | 0.59 | 0.09 | 1.00 | 0.04 | 0.16 | 0.26 | 0.00 | 0.07 | 0.28 | 0.01 | 0.81 |
| IAA16-B.1 | 0.32 | 1.00 | 0.44 | 0.39 | 0.20 | 0.21 | 0.39 | 0.36 | 0.56 | 0.33 | 0.04 | 0.08 | 0.40 | 0.21 | 0.70 |
