# Supplementary figures and images for: Identification of potential auxin response candidate genes for soybean rapid canopy coverage through comparative evolution and expression analysis
Source: Front Plant Sci. 2024 Oct 3;15:1463438. doi: 10.3389/fpls.2024.1463438 (PMC11484095; doi:10.3389/fpls.2024.1463438)

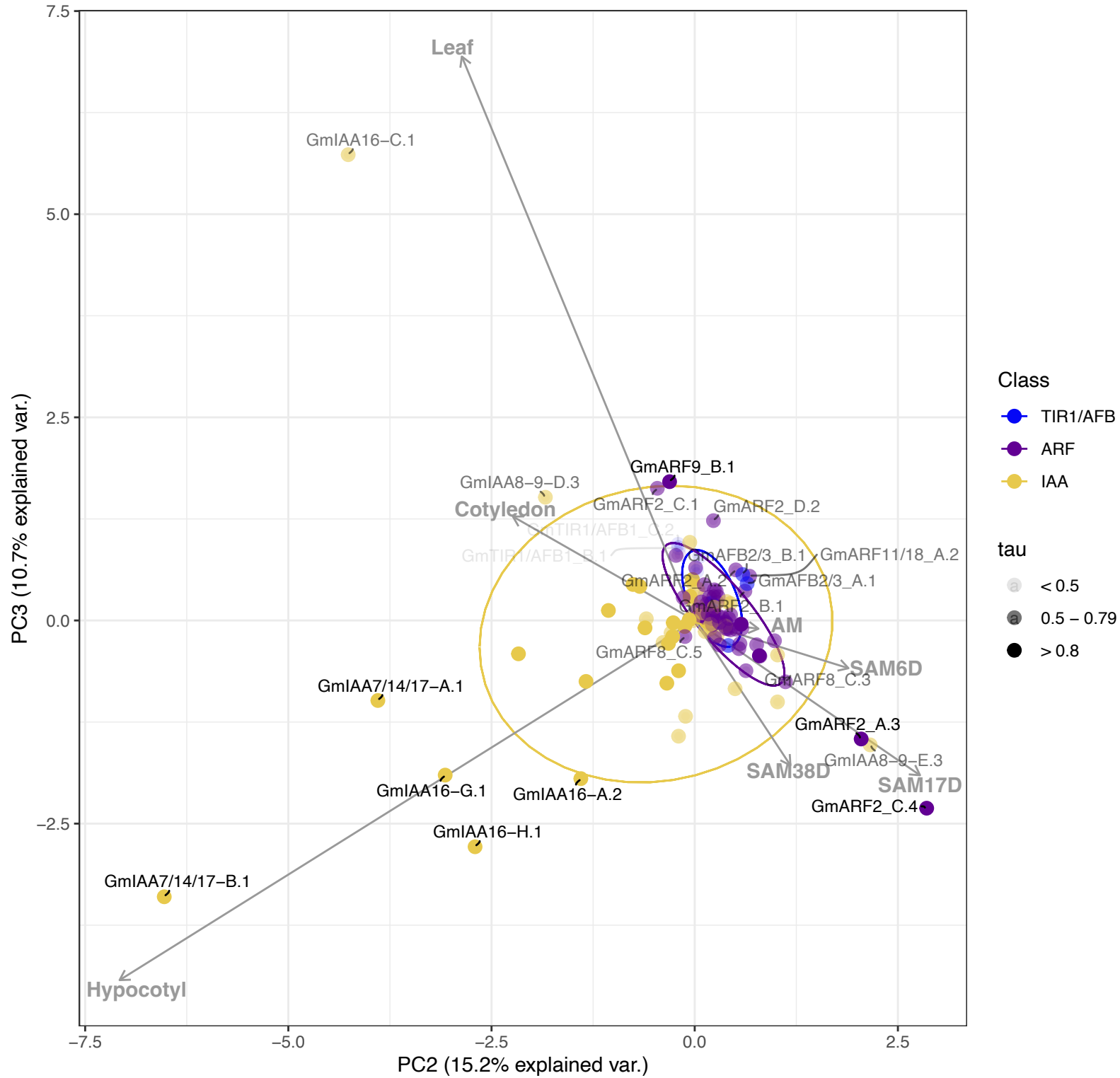

Supplement: Supplementary file 1 [file DataSheet1.zip › FigureS1_PC2_3_7Tissues_tau.pdf]

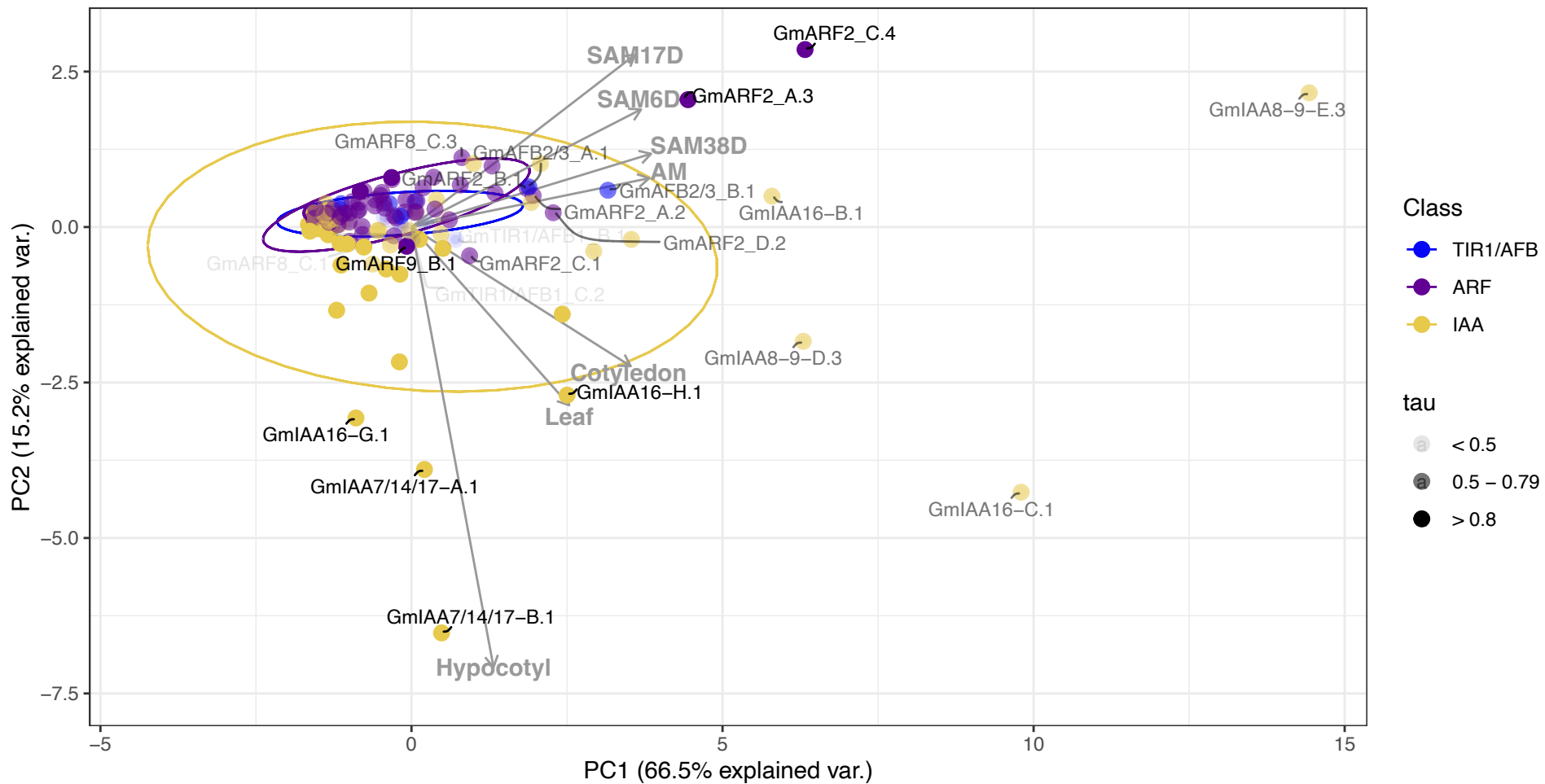

Supplement: Supplementary file 1 [file DataSheet1.zip › FigureS2_PC1_2_7Tissues_tau.pdf]

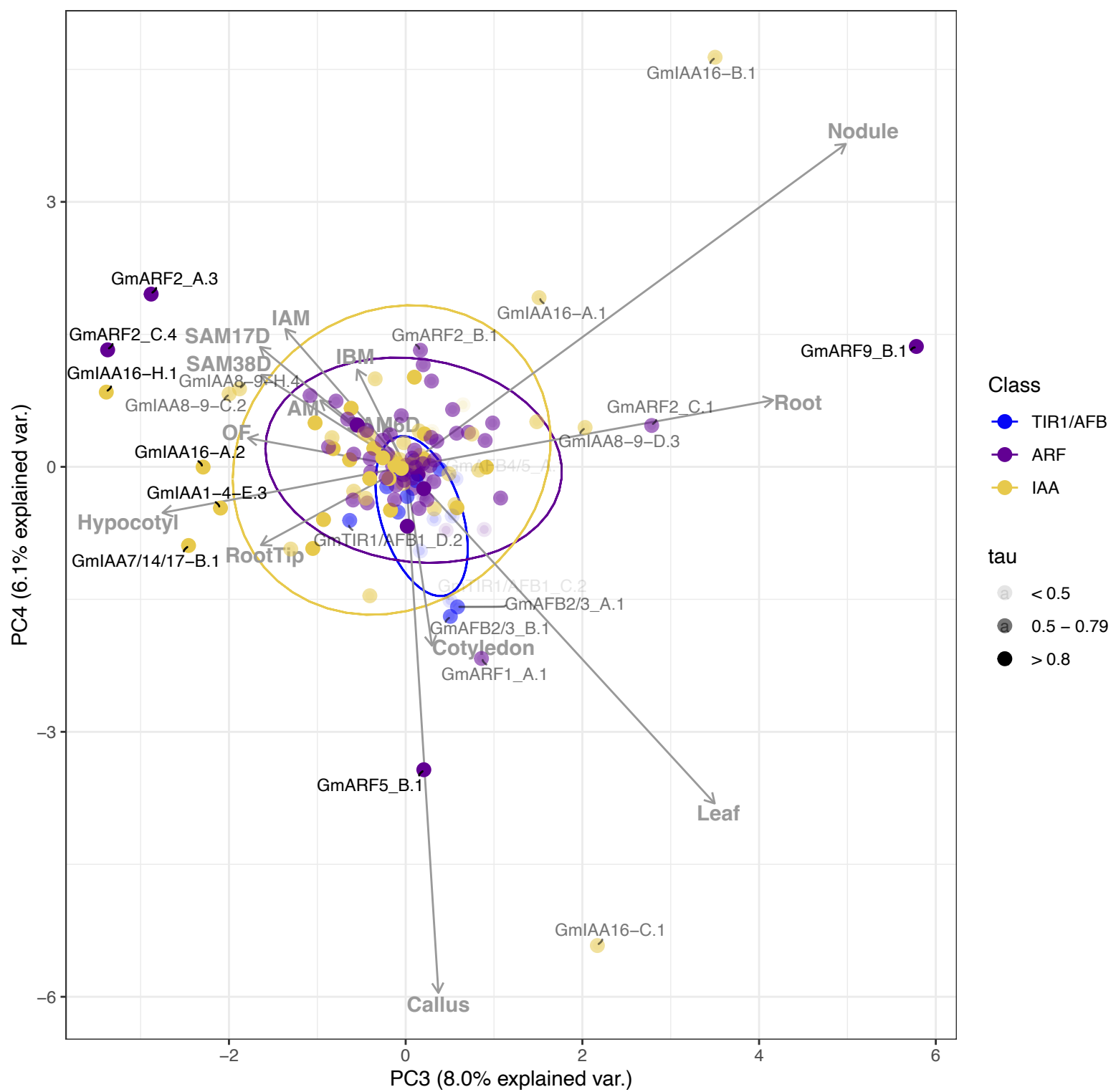

Supplement: Supplementary file 1 [file DataSheet1.zip › FigureS3_PC3_4_allTissues_tau.pdf]

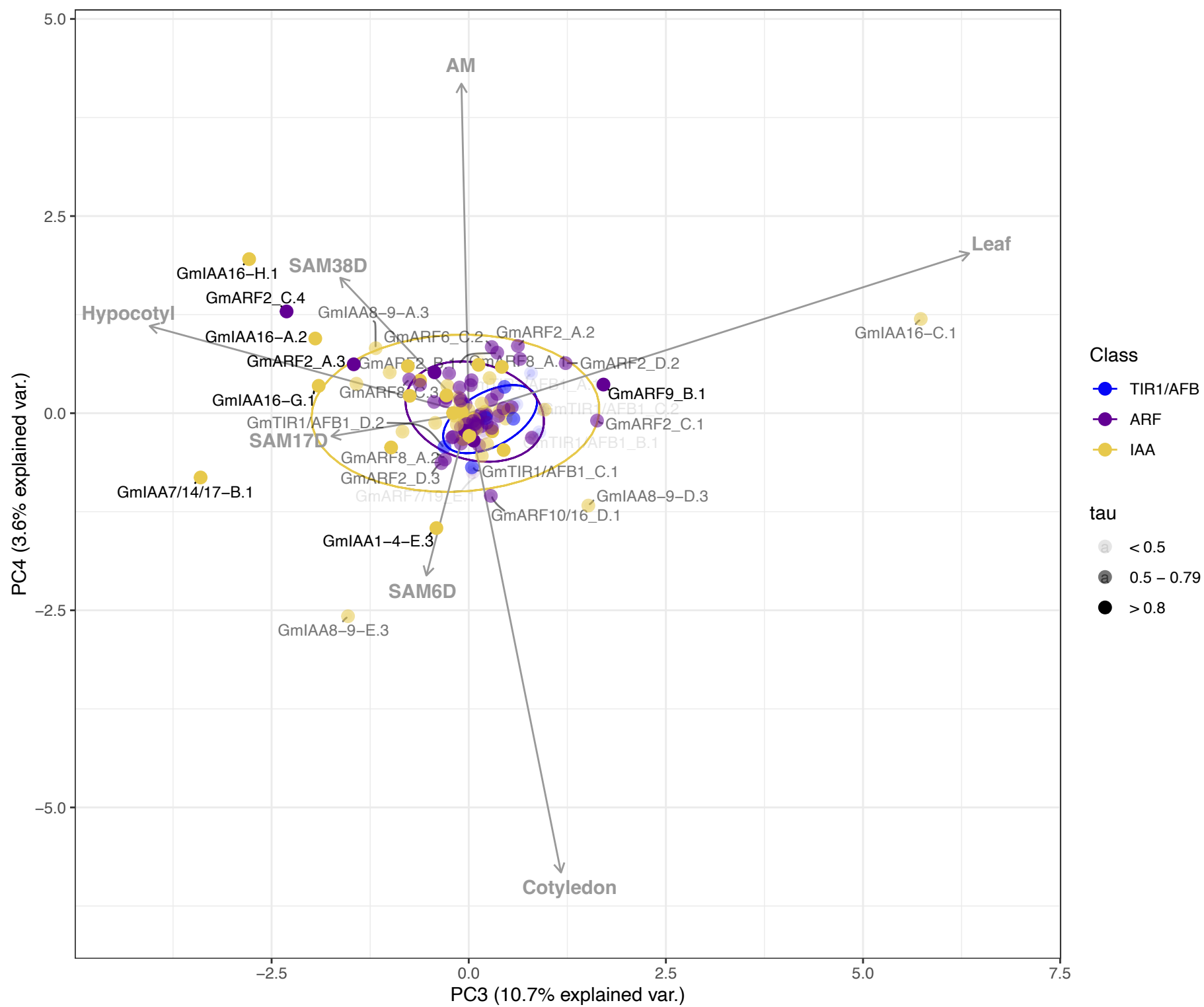

Supplement: Supplementary file 1 [file DataSheet1.zip › FigureS6_PC3_4_7Tissues_tau.pdf]
